# DNA methylation and chromatin accessibility predict age in the domestic dog

**DOI:** 10.1111/acel.14079

**Published:** 2024-01-23

**Authors:** Kelly Jin, Brianah M. McCoy, Elisabeth A. Goldman, Viktoria Usova, Victor Tkachev, Alex D. Chitsazan, Anneke Kakebeen, Unity Jeffery, Kate E. Creevy, Andrea Wills, Noah Snyder‐Mackler, Daniel E. L. Promislow

**Affiliations:** ^1^ Department of Laboratory Medicine & Pathology University of Washington Seattle Washington USA; ^2^ Center for Evolution and Medicine Arizona State University Tempe Arizona USA; ^3^ School of Life Sciences Arizona State University Tempe Arizona USA; ^4^ Department of Anthropology University of Oregon Eugene Oregon USA; ^5^ Division of Pediatric Hematology/Oncology Boston Children's Hospital Boston Massachusetts USA; ^6^ Dana Farber Cancer Institute Boston Massachusetts USA; ^7^ Harvard Medical School Boston Massachusetts USA; ^8^ Department of Biochemistry University of Washington Seattle Washington USA; ^9^ College of Veterinary Medicine Texas A & M University College Station Texas USA; ^10^ Department of Biology University of Washington Seattle Washington USA

**Keywords:** aging, ATAC‐seq, DNA methylation, dogs, epigenetic clock

## Abstract

Across mammals, the epigenome is highly predictive of chronological age. These “epigenetic clocks,” most of which have been built using DNA methylation (DNAm) profiles, have gained traction as biomarkers of aging and organismal health. While the ability of DNAm to predict chronological age has been repeatedly demonstrated, the ability of other epigenetic features to predict age remains unclear. Here, we use two types of epigenetic information—DNAm, and chromatin accessibility as measured by ATAC‐seq—to develop age predictors in peripheral blood mononuclear cells sampled from a population of domesticated dogs. We measured DNAm and ATAC‐seq profiles for 71 dogs, building separate predictive clocks from each, as well as the combined dataset. We also use fluorescence‐assisted cell sorting to quantify major lymphoid populations for each sample. We found that chromatin accessibility can accurately predict chronological age (R^2^_ATAC_ = 26%), though less accurately than the DNAm clock (R^2^_DNAm_ = 33%), and the clock built from the combined datasets was comparable to both (R^2^_combined_ = 29%). We also observed various populations of CD62L+ T cells significantly correlated with dog age. Finally, we found that all three clocks selected features that were in or near at least two protein‐coding genes: *BAIAP2* and *SCARF2*, both previously implicated in processes related to cognitive or neurological impairment. Taken together, these results highlight the potential of chromatin accessibility as a complementary epigenetic resource for modeling and investigating biologic age.

## Graphical abstract

We used two types of epigenetic information—DNA methylation (DNAm), and chromatin accessibility as measured by ATAC‐seq—to develop age predictors in peripheral blood mononuclear cells sampled from a population of 71 domesticated dogs. Chromatin accessibility can predict chronological age, though less accurately than the DNAm clock or the clock built from the combined datasets. The types of meta data features selected per clock also varied by data type. These results suggest that chromatin accessibility may act as a complementary epigenetic resource for investigating biological age.

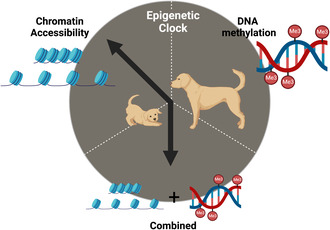

**Abbreviations**
ATAC seq: assay for transposase‐accessible chromatin using sequencing
BAIAP2: BAR/IMD domain containing adaptor protein
DN: double negative
DNAm: DNA methylation
EpiC Dog: Epigenome Catalog of the Dog
LOOCV: leave‐one‐out cross validation
PBMC: peripheral blood mononuclear cell
RMSE: root mean squared error
RRBS seq: reduced representation bisulfite sequencing
SCARF2: scavenger receptor class F member 2

## 1 INTRODUCTION

As we age, physiologic function steadily declines, while the risk of morbidity and mortality steadily rises (Kaeberlein et al., 2015). Although age is a reliable predictor of overall health across a population, there is substantial interindividual variability in how quickly we age, with some aging faster or slower than others (Christensen et al., 2004). To understand how and why we age, research has focused on developing tools to measure this variation and reveal the genetic and environmental factors that may influence it.

One promising area of study includes age‐associated changes in the epigenome. The epigenome consists of the collection of structural and biochemical changes in the cell that alter gene expression levels without changing the actual DNA sequence, including DNA methylation, histone modifications, and changes to chromatin accessibility (Sen et al., 2016). The epigenome integrates information from both genes and environment, and is rich with changes that correlate with and potentially directly influence organismal aging (Oberdoerffer & Sinclair, 2007; Sen et al., 2016). Changes in diverse epigenetic elements, including global loss of constitutive heterochromatin (Allshire & Madhani, 2018; Trojer & Reinberg, 2007), histone loss (Dang et al., 2009; O'Sullivan et al., 2010), and global and local changes in DNA methylation (Hernandez et al., 2011; Rakyan et al., 2010; Teschendorff et al., 2010) have all been associated with aging in vertebrate systems. In recent years, significant resources have been invested into using epigenetic markers to develop predictive models of age in hopes not only of predicting chronologic age, but also of estimating intrinsic measures of overall health compared to the population mean. Researchers have argued that the amount by which predicted age departs from chronological age can be taken as a measure of underlying health, referred to as age acceleration.

In addition to DNAm‐based biomarkers of age (Bocklandt et al., 2011; Hannum et al., 2013; Horvath, 2013; Levine, 2013), many other age predictors exist, including a wide range of molecular and clinical measurements, such as telomere length (Blackburn et al., 2006), gene expression transcripts (Holly et al., 2013; Peters et al., 2015), protein glycosylation and abundance (Krištić et al., 2014; Menni et al., 2015), metabolite levels (Hertel et al., 2016; Zhao et al., 2022), and composite clinical biomarkers such as systolic blood pressure and cholesterol levels (Belsky et al., 2015; Levine, 2013; Li et al., 2015). Across these different age predictors, the most accurate and well characterized are the DNA methylation (DNAm) clocks, which are also most thoroughly validated by independent studies. Two widely cited clocks are Horvath's 353 CpG multi‐tissue DNAm age estimator (Horvath, 2013), and Hannum's 71 CpG single‐tissue DNAm age estimator (Hannum et al., 2013). Both Horvath's and Hannum's DNAm clocks have been shown to be highly predictive of chronological age (Hannum et al., 2013; Horvath, 2013), predict of all‐cause mortality and life span (Marioni et al., 2015; Perna et al., 2016), and claim to measure biologic age or age acceleration in an organism or tissue (Horvath, 2013), measures that can sometimes predict age‐related phenotypes and diseases (Bell et al., 2019; Quach et al., 2017).

Applications of the DNAm clock now span many diverse areas of clinical and biological research, including clocks that are specific to human subpopulations (Horvath et al., 2016), clocks developed for nonhuman mammalian species, including but not limited to chimpanzees, mice, dogs, and humpback whales (Ake Lu et al., 2021; Ito et al., 2018; Maegawa et al., 2010; Polanowski et al., 2014; Thompson et al., 2017), and pairing of clocks with studies of life span‐extending interventions (Sziráki et al., 2018). A deeper understanding of how these DNAm sites relate to age can help further guide future applications of epigenetic clocks.

In this study, we sought to expand our understanding of these predictive clocks and the biology of aging by looking beyond DNAm measures of the epigenome and age. In particular, in addition to CpG methylation, here we also measure chromatin accessibility, building two independent age predictors, as well as one combined age predictor, from the same tissue samples. We define chromatin accessibility as regions of open chromatin that are accessible specifically to the modified transposase used in the assay for transposase‐accessible chromatin using sequencing (Buenrostro et al., 2013; ATAC‐seq). Chromatin accessibility as measured by ATAC‐seq has been used in a wide variety of basic and clinical research fields, including embryonic development (Wu et al., 2016), tumor development (Davie et al., 2015), and aging/age‐related disorders (Moskowitz et al., 2017; Wang et al., 2018).

We build and compare these age predictors across a population of individuals from a relatively new model system of aging, the companion dog (*Canis lupus familiaris*). The dog is an attractive model for aging research for a multitude of reasons. First, as the most phenotypically variable mammal on earth, dogs demonstrate considerable variation across breeds not only in morphology and behavior (Sutter et al., 2007; Boyko et al., 2010; MacLean et al., 2019), but also in life span and disease susceptibility (Fleming et al., 2011; Hayward et al., 2016). Larger breeds tend to have shorter life spans than smaller breeds (Galis et al., 2007; Patronek et al., 1997), suggesting that larger breeds may be aging faster than smaller breeds (Kraus et al., 2013). This leads us to hypothesize that for a given age, individuals of larger breeds should, on average, have an older biological age than individuals of smaller breeds, as measured by age acceleration in an epigenetic clock. Second, the unique breed‐based population structure of dogs results in high levels of genetic and phenotypic homogeneity within individual pure breeds, coupled with high levels of genetic and phenotypic *heterogeneity* between breeds (Lindblad‐Toh et al., 2005; Ostrander & Kruglyak, 2000; Parker et al., 2004). This structure affords researchers some level of control over genetics as well as increased confidence when comparing measurements of trait‐means across breeds. And lastly, dogs share our environment in a way that can never be replicated in laboratory settings. They are exposed to the same kinds of environmental factors as people are, such as second‐hand smoke, air pollution, and ambient noise. This affords researchers the opportunity to learn about the effect of these factors on human health from dog data.

Here, we measured DNA methylation and chromatin accessibility profiles of peripheral blood mononuclear cells (PBMCs) from 71 companion dogs. We used reduced representation bisulfite sequencing (Meissner et al., 2005; RRBS‐seq) to measure DNA methylation and ATAC‐seq to profile global chromatin accessibility. While other groups have built methylation age predictors from cohorts of companion dogs (Horvath et al., 2022; Thompson et al., 2017; Wang et al., 2020), our study is the first we know of to profile both methylation and chromatin accessibility from the same cohort of companion dogs. With these data, we developed a DNAm clock and, to the best of our knowledge, the first canine ATAC‐seq clock, and a combined DNAm/ATAC clock for estimating canine age. We also carried out univariate modeling to estimate the effects of age and other biologic and environmental factors on each feature. We found that (1) chromatin accessibility can accurately predict chronologic age (R^2^_ATAC_ = 26%), though less accurately than the DNAm clock (R^2^_DNAm_ = 33%), and the clock built from the combined datasets was comparable to both (R^2^_combined_ = 29%), (2) various populations of CD62L+ T cells significantly correlated with dog age, (3) all three clocks selected features that were in or near at least two protein‐coding genes: *BAIAP2* and *SCARF2*, both previously implicated in processes related to cognitive or neurologic impairment, and (4) different sets of meta data features were consistently selected in the model‐building process for the different data types. Taken together, this suggests that the biologic information captured by age‐associated changes in chromatin accessibility likely differ from those captured by DNAm changes, demonstrating that ATAC‐profiled chromatin accessibility may offer a complementary biologic perspective to that of DNAm that may help further elucidate the relationship between the epigenome and age.

## 2 RESULTS

### 2.1 Study cohort

**FIGURE 1:**
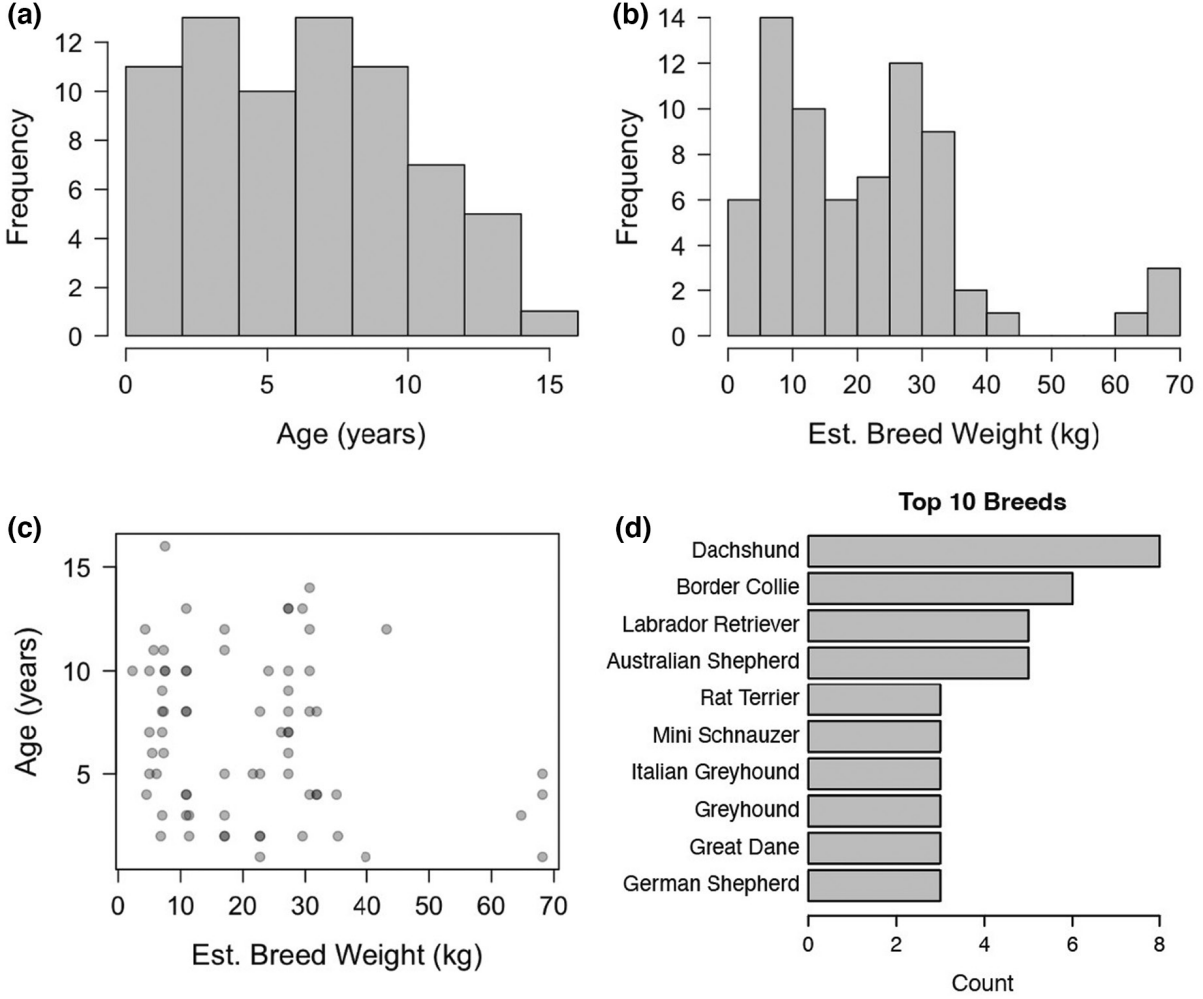
Sample cohort information. (a,b) Age and estimated breed weight distribution of 71 dogs in the cohort. (c) Correlation between age and estimated breed weight. (d) Top 10 most highly represented breeds in the cohort.

All dogs in this study were recruited at Texas A&M University, and were pets of staff and student volunteers. All animals were declared to be healthy by the owner. Age, breed, sex, and environmental survey information were reported by each owner. Ages ranged from 1 to 16 years old. Sixty‐eight out of 71 animals (96%) were sterilized, so we chose not to include sterilization status as a factor in this study. The distributions of age and breed size of the cohort are shown in Figure 1a,b, and the breakdown of age and breed size by sex are shown in Figure S1. There was no correlation between age and breed size of profiled dogs (Figure 1c). The most highly represented breeds included Dachshunds, Border Collies, Labrador Retrievers, and Australian Shepherds (Figure 1d). However, the majority of the cohort (60%) was composed of breeds represented by only one individual animal.

We isolated PBMCs from the fresh whole blood samples and split them two aliquots—one used for flow cytometry to measure relative cell type proportions, and the other used to measure chromatin accessibility and methylation using ATAC‐seq and RRBS‐seq, respectively.

### 2.2 Cell types and environmental factors correlated with age

Our goal here was to evaluate the relationship between epigenetic features and dog age. However, we first determined if environmental factors or PBMC type proportions were also correlated with age, in which case we would include them as potential covariates in our statistical models. To do this, we measured the correlations between all owner‐reported metrics about each animal's environment, as well as cell type proportions as measured by flow cytometry, with animal age. In total, this included 31 different cell types (Data S1) and 10 different categorical environmental factors, including variables such as diet and exercise type (for full list, see Data S1).

**FIGURE 2:**
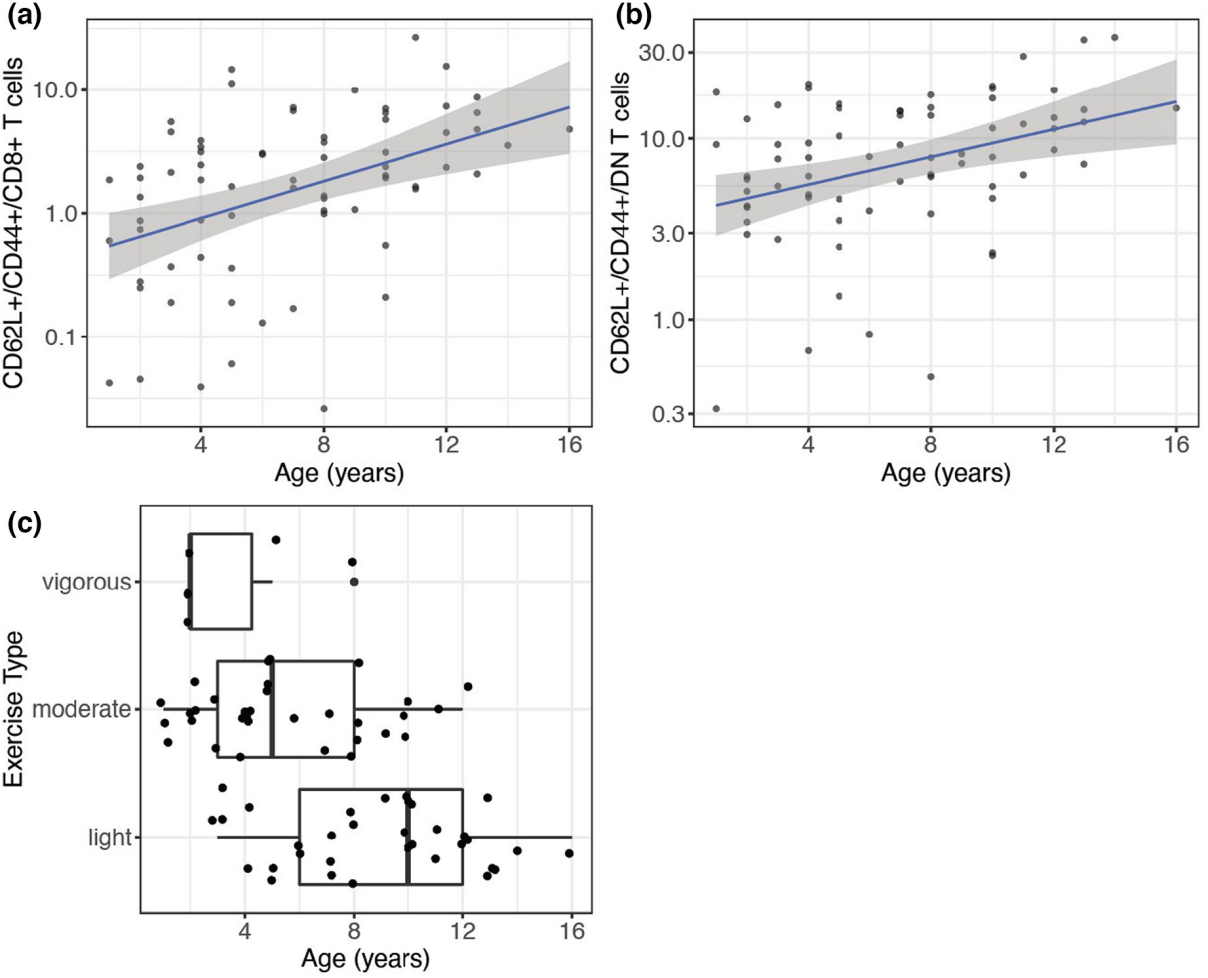
Cell types and survey questions correlated with age. (a,b) Out of 31 PBMC types measured with our flow cytometry panel, two types correlated significantly with age as tested using a linear model. Y axis units represent percentage of previous gated population as measured by FlowJo. See Data S2 for gating criteria and results. (c) Out of the lifestyle survey questions filled out by owners, responses to one question (exercise type) was significantly associated with age as tested using ANOVA.

Across all these variables, relative proportions of two cell types, CD62L+/CD44+/CD8+ T cells and CD62L+/CD44+/double negative (CD4‐/CD8‐; DN) T cells, were significantly correlated with age (Figure 2a,b), with older animals having greater proportions of these cells. CD44+/CD62L+ status is commonly used to identify populations of central memory T cells in mice, humans, and sometimes dogs (Nakajima et al., 2021; Sallusto et al., 2004; Withers et al., 2018), which have been shown to increase in number and proportion with age in humans (Saule et al., 2006). In addition to these two cell types, exercise type was also found to significantly vary with dog age, with younger animals exhibiting more vigorous exercise than older animals, as expected (Figure 2c). In order to account for variability in cell type proportions explaining epigenetic changes, all cell type proportions measured by flow cytometry were included as features for selection in the subsequent clock models.

### 2.3 Functional annotation of epigenetic features and their association with age

To assess global properties of the ATAC and methylation datasets, we performed feature level analysis on individual ATAC‐seq peaks and RRBS‐seq DNAm sites (15,417 ATAC peaks and 13,336 CpG sites after quality control filtering, see Section 4: Methods). First, we mapped each locus to the Epigenome Catalog of the Dog, a multi‐tissue canine chromatin state map (EpiC Dog; Son et al., 2023) to approximate a functional annotation for each feature. EpiC Dog is a curated repository of regulatory elements across chromatin datasets collected from 11 different tissue types in the companion dog (Figure S2a; Son et al., 2023). PBMCs were not profiled in EpiC Dog, so we chose to compare to the annotations from canine spleen tissue. Functional annotation of features from both datasets revealed that the vast majority of DNAm sites fell into inactive, quiescent regions of the genome, while the majority of ATAC features fell within more active regulatory regions, including enhancers and transcription start sites (Figure S2b).

Next, we modeled each feature as a function of age to identify age‐associated ATAC peaks and DNAm sites. We included weight category, sex, and other meta features correlated with age (Figure 2) as covariates in the model (Equation 1 in Methods). We observed that 1131 ATAC peaks and 40 DNAm sites were significantly associated with age (Figure S2c). For both gene regulatory measures, more age‐associated sites were decreasing in signal (either accessibility or methylation) with age rather than increasing (Figure S2c).To determine whether or not certain chromatin states were enriched within age‐associated features, we performed Fisher's exact tests on age‐associated ATAC features and whether or not they were significantly increasing or decreasing with age. Across the age‐associated ATAC features we observed features decreasing with age were more likely to be enriched for peaks that fell in active TSS, active TSS flanking regions, and active weak enhancers and vice versa—features increasing with age were depleted for the same elements (Figure S2d). We also observed that the opposite pattern was true of active enhancer and quiescent regions—these regions were enriched in ATAC features that were found to significantly increase with age (Figure S2d). Taken together, this suggests that typically inactive regions of chromatin may become more open and therefore active with age, consistent with the heterochromatin loss model of aging (Villeponteau, 1997).

### 2.4 The canine epigenetic clock

To evaluate the ability of our methylation and ATAC‐seq data to predict age in our cohort of dogs, we built separate predictors of age using elastic net regression (Equation 2 in Methods) performed on each dataset. In addition to evaluating each dataset separately, we also tried building a model using both combined datasets to ask whether combining information from both types of epigenetic landscapes improved age prediction. We also included certain metadata features including breed weight category and all PBMC types from flow cytometry as features available for selection in our training process, which we refer to as “meta features.”

**FIGURE 3:**
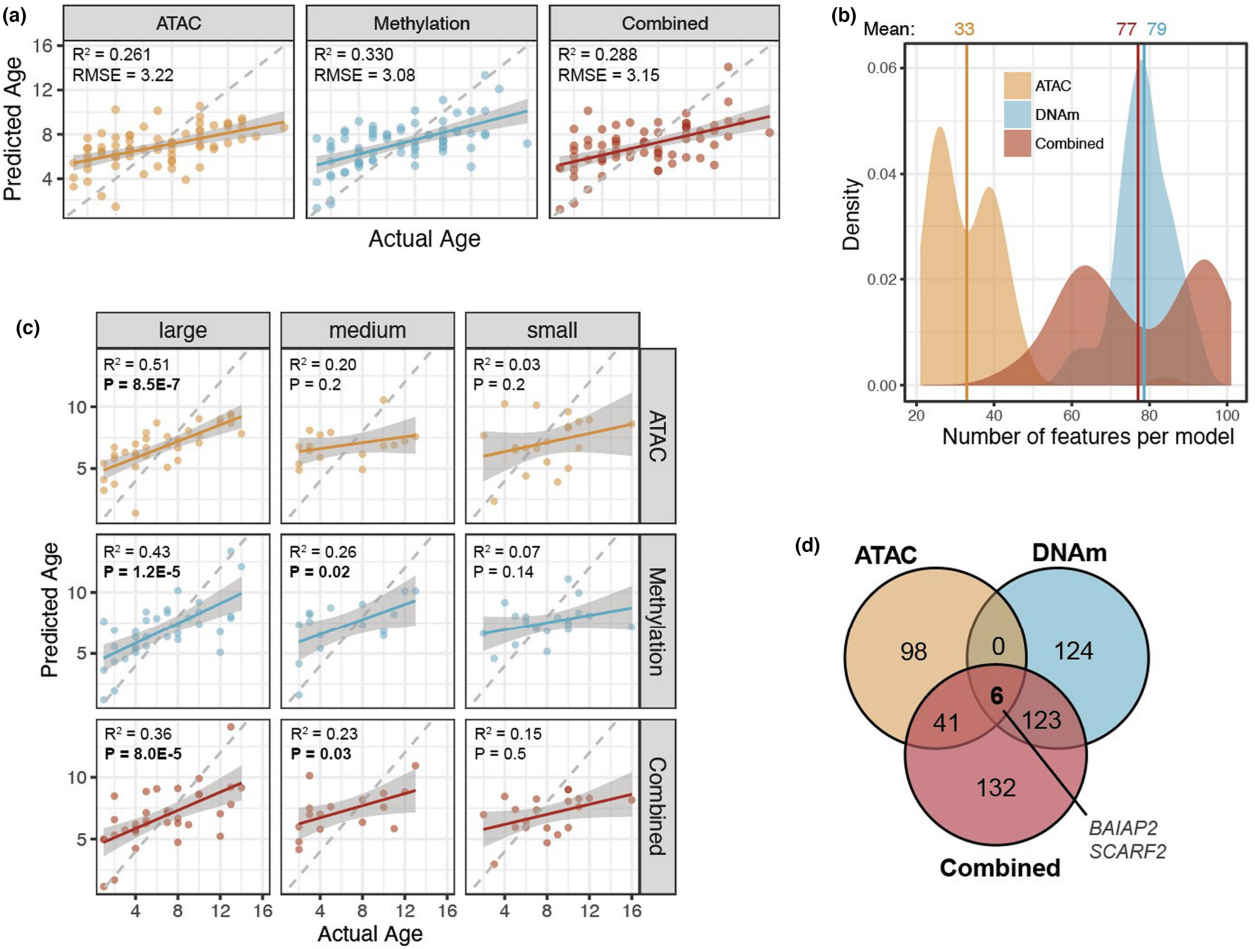
The canine epigenetic clock. (a) Comparison of age versus predicted age predicted from elastic net models from 71 dogs for three different sets of features. (b) Distribution of number of features selected per model. (c) Results from the top row of (a) are split by breed size category for all models. (d) Numbers of genes closest to features selected from each clock that overlap. Of the six genes that are found near features selected for all three clocks, two are associated with known protein‐coding genes: *BAIAP2* and *SCARF2*. All statistics generated from ordinary least squares linear regression.

Due to our limited sample size (71 dogs) and large feature set sizes, we used leave‐one‐out cross validation (LOOCV) approaches to evaluate the ability of each data type to predict age in each dataset using elastic net regression implemented from the R package glmnet (see Section 4: Methods). Briefly, we ran cv.glmnet() 71 times, each time “manually” leaving out one observed dog, and used the resultant model to predict the left out sample (Figure 3a). This results in 71 “final” models, each used to predict the left out sample. This allows us to evaluate the predictive capacity of each data type while ensuring that there is no overfitting within the model building process.

All three data types demonstrate similar accuracy when predicting age (Figure 3a), with the DNAm clock (R^2^_adj_ = 0.33, RMSE = 3.08) slightly outperforming the other two, followed by the combined clock (R^2^_adj_ = 0.29, RMSE = 3.15), and finally the ATAC clock (R^2^_adj_ = 0.26, RMSE = 3.22). While the correlation strength of predicted versus actual age from the three datasets are very similar, the nature of the models built varied between the three data types. All ATAC models showed fewer numbers of features selected than DNAm and combined clocks (Figure 3b), which is also consistent with greater observed mean values of lambda selected for each ATAC clock (Figure S3a).

To determine whether the clock was better at predicting age for certain types of breed, we partitioned the predicted ages by large, medium, and small breeds. Across all three data types, we observe the strongest and most significant correlations between predicted and actual age across the large breeds, though it is most apparent in the ATAC clock results (Figure 3c). For all models, dogs from small breeds showed the worst performance in age prediction (Figure 3c).

### 2.5 Gene related to cognitive and neuronal function are enriched near sites selected for three clocks

To determine whether or not there was any biological significance to the genes located near the features selected for each clock, we mapped each feature to the closest known gene in the canine genome. We included all features selected one or more times across all 71 models (n_ATAC_ = 147 features, n_DNAm_ = 281 features, n_Combined_ = 324 features).

Six genes were found to overlap between the three clocks (Figure 3d), two of which are protein coding genes: BAR/IMD domain containing adaptor protein (*BAIAP2*) and scavenger receptor class F member 2 (*SCARF2*). *BAIAP2* (also known as IRSp53), a brain‐specific insulin receptor tyrosine kinase substrate which has been shown to be involved in impaired memory, learning, and other cognitive deficits in mouse models of Alzheimer's (Gatta et al., 2014; Kim et al., 2009). Increased *SCARF2* expression has been detected in glioblastoma, an age‐associated neurologic disorder, compared to regular brain tissue (Kim et al., 2022).

### 2.6 Weight and certain cell types were commonly selected as predictive features in certain clocks

**FIGURE 4:**
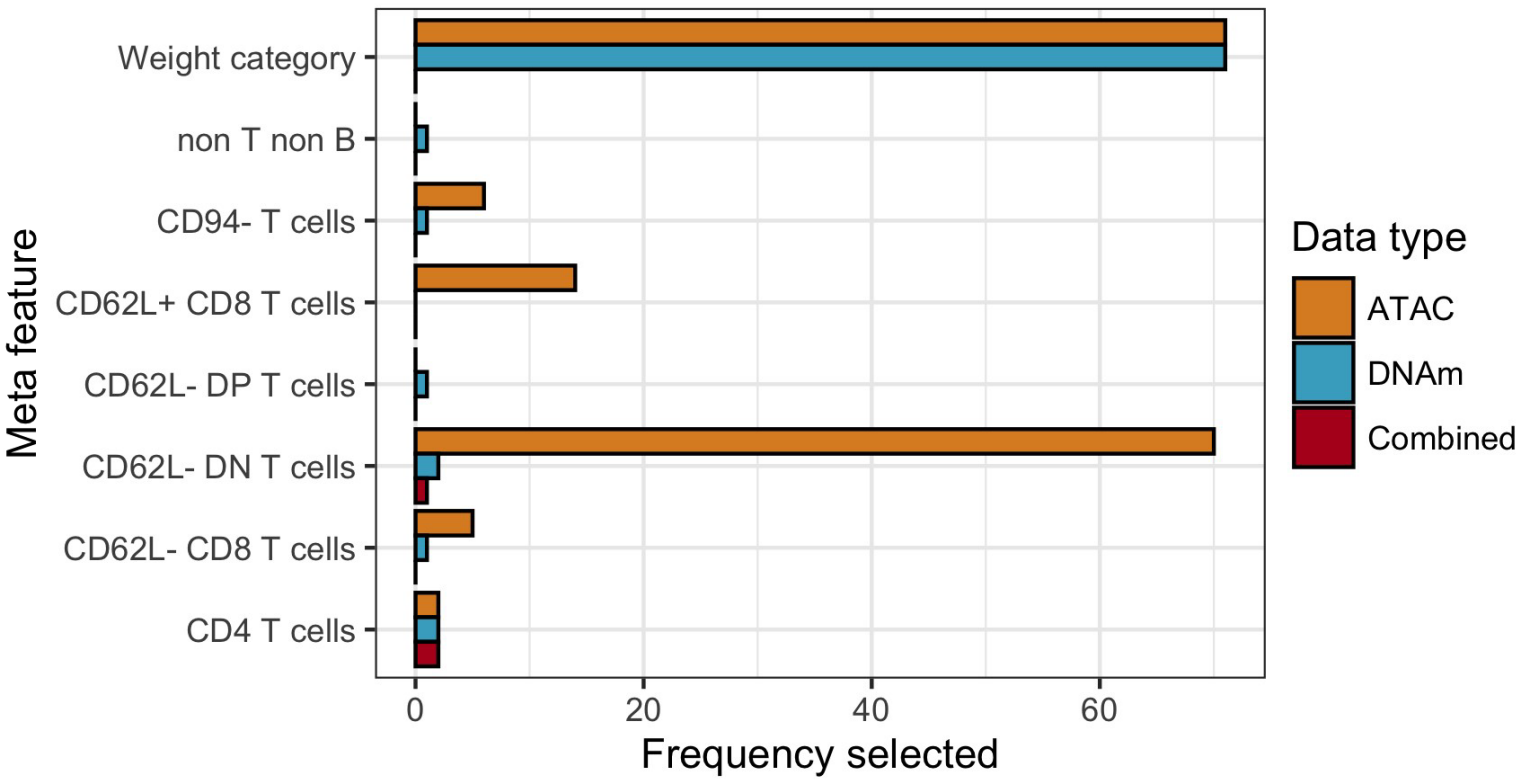
Meta features selected by epigenetic clocks. Summary of the number of instances meta features (including all PBMC types and breed weight category) were selected across all final models. The maximum number of instances each feature can be selected across each data type is 71 (one for each dog).

To get a sense of whether or not certain meta features (PBMC types and breed weight category), which were also included as features for selection in the elastic net model training process, are important for predicting age, we examined the 71 different feature sets selected for each data type. We found that across all the metadata features (PBMC types and breed weight category) that were included as options for features to be selected by the elastic net training process, only a handful of features were selected in greater than 10 models: weight category, CD62L‐ DN T cells, and CD62L+ CD8 T cells in 14 of the ATAC models (Figure 4; Table S1). Breed weight category was selected as a feature across all 71 models in both ATAC and DNAm clocks (but not the combined clock), while CD62L‐ DN T cell proportions were selected in almost all (70 out of 71) instances of ATAC clock building, but only once or twice in the DNAm or combined clocks. CD62L+ CD8 T cells were selected in 14 of the 71 ATAC models only (Figure 4; Table S1).

### 2.7 Residual age measures are not associated with breed weight

Next, we evaluated the ability of our clocks to assess biological health relative to a dog's age, that is, age acceleration, which can take on a positive (acceleration) or negative (deceleration) value. We generated an estimate of age acceleration by taking the residuals of an ordinary least squares linear regression of predicted versus observed age. From this point onward, we refer to this measure as “residual age.” Our method of measuring residual age is comparable to the measures of “age acceleration” from Horvath's clocks (Horvath, 2013; Thompson et al., 2017), which have been shown to be predictive of overall health (Bell et al., 2019; Horvath et al., 2014; Quach et al., 2017).

**FIGURE 5:**
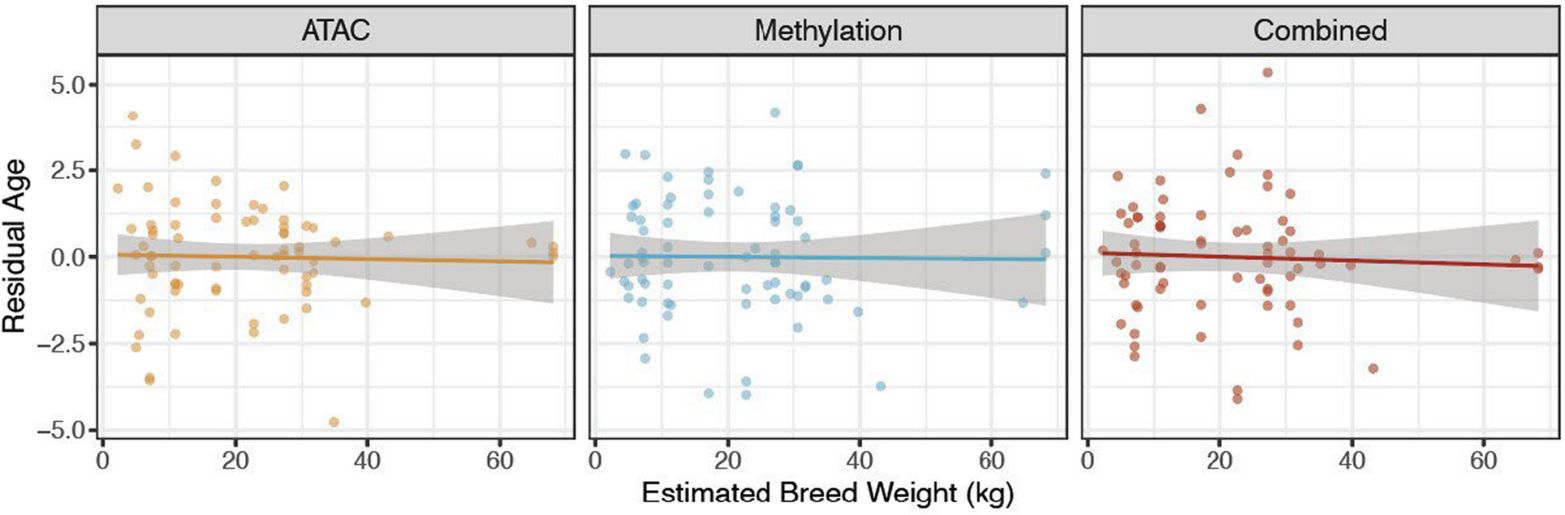
Residual age predictions. Relationship between estimated breed weight and residual age prediction from the three clocks from the final model.

If age acceleration is predictive of life span and overall health, we may expect to see a positive relationship between residual age and breed size. More specifically, we predict that larger breeds, which are shorter lived and age at a more rapid rate (Kraus et al., 2013; Patronek et al., 1997), would show a higher residual age than smaller dogs. To test this, we modeled residual age as a function of breed size (as measured by the mean weight of that breed reported by the American Kennel Club) with the three clocks. We did not find any strong correlation between residual age and breed weight across any of our three clocks (Figure 5).

Furthermore, if our epigenetic age measures both represent a shared marker of biological aging, then we would expect the residual age measures to be correlated with one another (i.e., if a given dog had a positive, or “accelerated,” DNAm residual age, then they would have a similarly positive ATAC‐seq residual age). We found no relationship between residual age from the methylation clock and residual age from any of our three clocks (Figure S3b). Collectively, our data do not provide evidence that residual age as estimated from either clock is predictive of breed size, and therefore likely life span, in companion dogs.

## 3 DISCUSSION

Here, we present what is to the best of our knowledge one of the first ATAC‐based predictors of age, coupled with DNAm and a combined predictor of age from the same set of animals. There are three notable findings from this study that we highlight here. First, this study shows that it is possible to build an accurate predictor of age using chromatin accessibility data as measured by ATAC‐seq, and performs comparably to an age predictor built from DNAm data or one build from both ATAC and DNAm when using a rigorous, LOOCV approach to evaluate age prediction (Figure 3a).

Second, while all three clocks are able to predict age to a comparable degree, other aspects of their performance and feature selection suggest that each data type captures different biologic information about the aging process, and thus, may each offer unique biologic insight into the biology of aging. This is demonstrated by the fact that the three different clocks repeatedly selected different types of meta features (PBMC types or breed weight; Figure 4). While the DNAm and combined clocks rarely selected any PBMC types as features to predict age, the ATAC clock almost always included one cell type—CD62L‐ DN T cells—in its list of features for age prediction, despite the fact that both the DNAm and chromatin accessibility datasets were collected from the same set of PBMC samples. This suggests that DNAm and chromatin accessibility might be influenced by age and other biologic and environmental factors in different ways. As such, while the majority of efforts studying epigenetic age have been heavily focused on the methylome, we could gain deeper insight into aging biology by characterizing and understanding other features of the epigenome, such as chromatin structure.

Finally, while the two types of epigenetic data can predict age, we were unable to find evidence for their ability to capture biological age, or general health, of the animals as estimated by breed size/longevity. Neither the ATAC, DNAm, nor combined residual age measures correlated with breed (Figure 5). While there is extensive evidence in human studies for the ability of age clocks to predict health and longevity metrics (Horvath, 2013; Horvath et al., 2014; Marioni et al., 2015; Perna et al., 2016), we were not able to detect it here by using breed size as an estimate for breed longevity. This could be due to many factors, including a small sample size of our study, and/or noise from the model building methods. Moreover, we lack diagnostic health information, assuming instead that for a given age, an individual from a shorter lived breed would have an older biologic age than one from a longer lived breed. The lack of association with life span might also be a unique property of epigenetic age predictors in canines, as at least two previous dog clock studies have also tried and failed to find association between biologic age as estimated by DNAm clocks and breed size/longevity (Horvath et al., 2022; Thompson et al., 2017).

At least three other studies have used DNA methylation data to build predictors of age in dogs. The first study to do so was described by (Thompson et al. (2017)), followed by (Wang et al. (2020)), and most recently, (Horvath et al., 2022). While all of these studies, ours included, successfully built DNAm clocks in companion dogs, each reveals unique aspects of the canine epigenetic clock. The Thompson study was the first to compare the DNAm clock in dogs to ones built from wolves and humans (Thompson et al., 2017). Wang et al. demonstrated that syntenic regions of the mammalian DNA methylome that change with age can be used to predict age across species, specifically dogs and mice, and that these regions occur in modules of developmental genes (Wang et al., 2020). Most recently, Horvath et al. built individual and shared DNAm clocks between a large cohort of dogs and humans. While they failed to find association between biologic age as directly estimated from their DNAm clocks, they built a novel predictor of “average time‐to‐death,” which generated estimates that were indeed predictive of breed weight and longevity (Horvath et al., 2022). In our study, given our small cohort and relatively small number of breeds with sufficient representation, we lacked the statistical power to build a rigorous time‐to‐death clock. Rather, our primary objective was to compare two different types of epigenetic information using a single population of dogs.

Several caveats should be considered here. First, the sample size (*n* = 71 dogs) is relatively small, and while we are still able to build a highly predictive age model with this group of animals, the lack of correlation of our residual age measures with breed or life expectancy might be due to lack of statistical power. We also acknowledge that the distribution of dog breeds included in this dataset is skewed toward larger breeds, which may impact the models shown in Figure 3c. However, due to the fact that other studies have reported similar observations of more accelerated aging in larger dogs (Rubbi et al., 2022), we feel this result is still important to highlight. In the future, the Dog Aging Project (Creevy et al., 2022), will build epigenetic clocks in a set of over 1000 dogs followed longitudinally over the course of their lives. Our results establish the feasibility, and provide us with a lower bound on efficacy for such measures. These future studies will include efforts to build not only a global biologic clock for all dogs, but breed‐specific ones as well.

Second, the demographic data for the dogs in this study, including age and breed, were reported by the owners and have not been verified through objective measures (e.g., veterinary electronic medical records, registration records). While we have no reason to believe any of the self‐reported responses are inaccurate, we acknowledge that information about pets, particularly age and breed, are not always well documented and might be subject to error.

Despite these caveats, our results point to the exciting new landscape of studies of health and aging now being pursued in companion dogs. The unique breed structure and highly variable longevity patterns of the domestic dog offer straightforward aging‐related hypotheses to generate and test. Dogs suffer from many of the same diseases as humans do (Hoffman et al., 2018), with a concomitantly sophisticated health‐care system, and are exposed to many of the same environmental risk factors as humans. Furthermore, canine health itself, independent from modeling human health, is an important area of study, motivated by the fact that owners care a great deal about their canine companions. Thus, there is tremendous potential for canine biologic and chronological age clocks to be applied in diverse contexts. These clocks have the potential not only to inform us about the health of pets, but also to generate very accurate estimates of chronological age, as the majority of adopted or rescued animals have no veterinary records with which to inform owners about age. We hope that studies such as ours will generate more enthusiasm and excitement about using companion dogs to learn about human health.

## 4 METHODS

### 4.1 Study cohort

We measured chromatin accessibility and methylation status of PBMCs in 71 healthy companion dogs using ATAC‐seq and RRBS‐seq, respectively. All dogs were recruited at Texas A&M University and comprised of pets of staff and student volunteers. All animals were declared to be healthy by the owner, although no formal veterinary exams were performed. Age, breed, and environmental survey information were reported by each owner. Sixty‐eight out of 71 animals were sterilized, so we chose not to include sterilization status as a factor in this study. Individual animal weights were not recorded. Average adult breed weight as reported by the American Kennel Club in 2012 was used throughout the analysis. All procedures for this study were reviewed and approved by the TAMU Institutional Animal Care and Use Committee (IACUC 2016–0224 CA). Because dog owners provided information about their dogs in the home environment, the study was also reviewed and approved by the TAMU Institutional Review Board (IRB2016‐0532D). Informed consent was obtained from all owners at the time of enrollment.

The distributions of age and breed size of the cohort are shown in Figure 1a,b. There was no correlation between age and breed size of profiled dogs (Figure 1c). The most highly represented breeds included Dachshunds, Border Collies, Labrador Retrievers, and Australian Shepherds (Figure 1d). However, the cohort was composed primarily of breeds represented by only one individual animal.

Whole blood was drawn and PBMCs were isolated in Texas, cryopreserved (detailed below), and then shipped to Seattle, Washington where the remaining epigenetic profiling and analyses were performed.

### 4.2 Sample collection and PBMC isolation

Using a needle and syringe, blood (5 mL) was collected from a peripheral vein by routine venipuncture and immediately transferred to K_2_EDTA vacutainers. Blood was mixed with an equal volume of 2% fetal bovine serum (HyClone) in phosphate buffered saline (HyClone), and transferred to a barrier tube (SepMate‐15, StemCell technologies) prefilled with 4.5 mL of density gradient medium (Lymphoprep 1.077, StemCell technologies). After centrifugation at 1200 *g* for 15 min at room temperature, the supernatant was collected and washed three times with 10 mL of 2% fetal bovine serum in phosphate buffered saline by centrifugation at 300 *g* for 10 min at room temperature. Based on a hemocytometer count, cells were resuspended at a concentration of 1 × 10^6^ per mL in fetal bovine serum with 10% DMSO. After 25 min incubation at room temperature, the cells were transferred to a −80°C freezer within a Styrofoam container. Samples were held at −80°C for a maximum of 4 days before shipping on dry ice. Once arriving in Seattle, samples were rapidly thawed at 37°C for 60 s, a small volume was stained with Trypan Blue, and then counted using a hemocytometer to obtain cell concentration and viability estimates. Samples were then immediately distributed into aliquots for downstream analyses, including ATAC‐seq, RRBS‐seq, and flow cytometry analysis.

### 4.3 ATAC‐seq library preparation

ATAC‐seq was performed on canine PBMCs largely following the original protocol from (Buenrostro et al. (2013)), with some modifications (Kakebeen et al., 2020). Briefly, 250,000 cells were washed 3x in 1x PBS by spinning for 2 min at 2000 g. In contrast to the original published methods, we skipped the cell lysis step and moved immediately to the transposition reaction by adding the transposition buffer and transposase directly to the washed cell pellet. Transposition was carried out at 37°C for 1 h. DNA from the transposed sample was then purified using a Qiagen Minelute kit as per manufacturer's instructions. PCR amplification of purified DNA was then conducted using Nextera PCR primers and NEB Next High‐Fidelity 2x PCR Master Mix (cat no. M0541s) using the recipe and cycling program as previously described (Buenrostro et al., 2013). Amplification was monitored in parallel using qPCR in order to reduce GC and size bias. The amplified reaction was then purified using a Qiagen PCR Cleanup kit. The final library was eluted in Qiagen Elution Buffer (10 mM Tris Buffer, pH 8) and stored at −20°C until ready for sequencing.

Samples were prepared as described above in batch sizes ranging from 6 to 12 samples. After all the samples were processed, all libraries were pooled for sequencing using the Illumina Nextseq 500 High Output Kit at the Brotman Baty Institute at the University of Washington.

### 4.4 ATAC‐seq data analysis

Software and parameters used for adaptor trimming, read alignment, and peak calling parameters were followed as described in (Kakebeen et al., 2020). Briefly, adapters were trimmed from reads and low‐quality sequences (Phred <33) were removed using Trim Galore! (https://github.com/FelixKrueger/TrimGalore). Reads were aligned to CanFam 3.1 using Bowtie2 (option:–very‐sensitive) (Langmead & Salzberg, 2012). Duplicate reads were marked using Picard “MarkDuplicates” (http://broadinstitute.github.io/picard/). Duplicate reads were removed using SAMtools (Danecek et al., 2021).

A consensus peak set was used to determine feature signal for all samples. The consensus peaks were called on a merged BAM file composed of equally subsampled reads from all donors in the experiment. Peaks with summits that were closer than 500 bp to one another were merged and considered as a single feature. Peaks were filtered to include peaks with a median coverage of >20 reads across all samples. Peaks that mapped to mitochondrial or DNA scaffolds were also removed. After filtering, 15,417 features remained in the dataset.

Count values were then converted to reads per kilobases mapped (RPKM) by dividing the number of reads at each peak region by the peak width (estimated from Macs2 peak‐calling software) and total reads mapped for each sample. These values were then log transformed, centered, and scaled prior to model building.

### 4.5 RRBS seq library preparation

RRBS libraries were generated from ~300 ng of DNA extracted from canine PBMCs following a modified version of Boyle et al. (2012). A detailed protocol can be found at https://doi.org/10.17504/protocols.io.e6nvwkxb9vmk/v1

### 4.6 RRBS seq data analysis

Samples were sequenced on the Illumina NovaSeq 6000 platform at the Northwest Genomics Center. Sequenced reads were trimmed with software Trim Galore!, and trimmed reads were mapped to the dog genome (CanFam 3.1). Total methylated and unmethylated CpG sites were counted from mapped reads. CpG sites were filtered to include sites with a mean depth of 5X and median methylation level between 0.1 and 0.9 to exclude constitutively hyper‐ or hypo‐methylated sites. Sites that mapped to mitochondrial or scaffold DNA were also removed. After filtering, 14,336 sites remained in the dataset. These values were centered and scaled prior to model building.

### 4.7 Flow cytometry

Cryopreserved canine PBMC samples were thawed in a 37°C water bath, half of the amount of each sample was used for flow cytometrical staining, and half was refrozen for future analysis. Samples for flow cytometry were transferred into 50 mL conical tubes and diluted in RPMI‐1640 culture media. Cells were washed twice with RPMI‐1640 by spinning at 300 g for 8 min, the resulting cellular pellets were resuspended in 50 μL of FACS staining buffer (2% fetal bovine serum in PBS) and stained with 18 μL of antibody cocktail, which includes FITC‐conjugated anti‐canine CD3 clone CA17.2A12 (Bio‐Rad MCA1774F), PE‐Cyanine 7‐conjugated anti‐canine CD4, clone YKIX302.9 (eBioscience 25–5040‐42), Pacific Blue‐conjugated anti‐canine CD8, clone YCATE55.9 (Bio‐Rad MCA1039PB), APC‐AlexaFluor 750‐conjugated anti‐human CD11b clone Bear1 (Beckman Coulter A97052), Brilliant Violet 605‐conjugated anti‐human CD14, clone M5E2 (Becton Dickenson 564,054), Alexa Fluor 647‐conjugated anti‐canine CD21, clone CA2.1D6 (Bio‐Rad MCA1781A647), Brilliant Violet 785‐conjugated anti‐mouse/human CD44, clone IM7 (Biolegend 103,059), PE‐conjugated anti‐human CD62L clone FMC46 (Bio‐Rad MCA1076PE), and Brilliant UltraViolet 395‐conjugated anti‐human CD94, clone HP‐3D9 (BD OptiBuild 743954). Cells were stained for 20 min at 4°C and washed twice with FACS staining buffer. After the last wash, stained cells were resuspended in FACS buffer containing 7‐AAD (1:500 dilution) and immediately run on an LSR Fortessa flow cytometer (BD Biosciences). Data were analyzed using FlowJo 10. Doublets were excluded based on FSC‐A/FSC‐H and SSC‐A/SSC‐H gating. Lymphocytes, monocytes, and granulocytes were gated based on FSC‐A and SSC‐A parameters, confirmed by lineage‐restricted expression of CD11b and CD14. T cells were defined as CD3+/CD21‐ lymphocytes, B cells were defined as CD3‐/CD21+ lymphocytes, NK cells were defined as CD3‐/CD21‐/CD94+ lymphocytes; within T cells we identified the following populations: CD94+ T cells defined as CD3+/CD21‐/CD94+ lymphocytes and conventional CD94‐ T cells defined as CD3+/CD21‐/CD94‐ lymphocytes. CD4 and CD8 T cells were defined within CD94‐ T cells as CD4+/CD8‐/CD3+/CD21‐/CD94‐ lymphocytes and CD4‐/CD8+/CD3+/CD21‐/CD94lymphocytes, respectively. Double‐positive and double‐negative T cells were defined within CD94‐ T cells as CD4+/CD8+/CD3+/CD21‐/CD94‐ lymphocytes and CD4‐/CD8‐/CD3+/CD21‐/CD94‐ lymphocytes, respectively. Within CD4 and CD8 T cells, we defined CD62L‐ and CD62L+ subsets as well as CD44Low and CD44High subsets.

### 4.8 Statistical analysis

All data analysis and visualization were performed using the statistical analysis software package R version 4.1+ (R Core Team, 2018). *P*‐values were adjusted for multiple comparisons using the Benjamini–Hochberg–Yekutieli procedure (Benjamini & Hochberg, 1995).

#### 4.8.1 Age‐associated features

We used ordinary least squares linear models to identify age‐associated peaks, modeling each feature as a function of age and other covariates, which included estimated breed weight, sex, exercise level, CD62L+/CD44+/CD8+ T cell proportion, and CD62L+/CD44+/DN T cell proportion. The latter three covariates were included because all are associated with age (Figure 2):

(1) Feature~age+weight+sex+exercise+cell.CD8+cdll.DN

#### 4.8.2 Chromatin state annotation

We performed the annotation of age‐associated ATAC peaks and CpG sites by utilizing genomic feature annotations sourced from multiple references. Specifically, chromatin state information was obtained from the Epigenome Catalog of the Dog. CpG islands were extracted from the UCSC Genome Browser, specifically for the CanFam3.1 genome assembly. Additionally, information pertaining to gene promoters and genes was also obtained from the UCSC repository utilizing the CanFam3.1 reference genome. All annotations were carried out using a combination of Bedtools Intersect (bedtools v2.31.0) and FindOverlaps() function from the GenomicRanges package in R (package).

#### 4.8.3 Chromatin state enrichment analysis

We conducted a chromatin state enrichment analysis using a Fisher's exact test in R using fisher.test () to investigate the relationship between chromatin states (designated as 1–13) and age‐associated ATAC peaks categorized as increasing or decreasing.

#### 4.8.4 Epigenetic clocks

We use the R package glmnet (version 4.1–4) to build epigenetic clocks using either ATAC‐seq or RRBS‐seq data. We used an elastic net model using the loss function

(2) $$\begin{array}{c} \min\\ \left(\beta_{0,}\beta \right)\in R^{p+1} \end{array}\frac{1}{2N}\;\sum_{i=1}^{N}{\left(y_{i}-\beta_{0}-x_{i}^{T}\beta \right)}^{2}+\lambda \left[\left(1-\alpha \right)∥\beta {∥}_{2}^{2}/2+\alpha ∥\beta {∥}_{1}\right]$$

where *N* is the number of samples, *y*_*i*_ is the age of dog *i*, and *x* is the epigenetic profile. The model is built with two parameters, including a mixing parameter alpha (*α*) and a regularization parameter lambda (*λ*). Briefly, *α* determines whether or not the model will use Ridge regression (*α* = 0), Lasso regression (*α* = 1), or a mixture of both (0 < *α* < 1). The role of the regularization parameter is to minimize mean‐squared error. The greater the value of *λ*, the greater the penalty and the smaller the overall coefficient size of the models. We trained our models by setting *α* to 0.5 (elastic net, or an equal balance between Ridge and Lasso) and optimizing *λ*. We used a leave‐one‐out‐cross validation (LOOCV) approach. Specifically, we used the function cv.glmnet, but “manually” excluded a single observation each time, resulting in one model per dog per data type. The predicted ages from this method are shown in Figure 3a. The distributions of the number of features and optimal lambda values from each of these models are shown in Figure 3b and Figure S3a, respectively.

## AUTHOR CONTRIBUTIONS

D.E.L.P. and N.S‐M. conceived of the presented idea, supervised analysis, and are co‐corresponding authors. K.E.C. and U.J. collected and processed tissue samples from animals. V.T. collected and analyzed flow cytometry data. A.W. supervised ATAC library generation. K.J., A.D.C., A.K., and V.U. prepared and processed ATAC libraries. N.S‐M., V.U., and E.A.G. prepared and processed RRBS libraries. K.J., B.M.M., and E.A.G. analyzed the data. K.J. made all figures. K.J., D.E.L.P., and N.S‐M. wrote the article text. All authors reviewed and contributed to the final article.

## CONFLICT OF INTEREST STATEMENT

The authors declare that they have no conflicts of interests.

## Supporting information


Data S1.

Data S2.

Table S1.

Figures S1–S3.

## Data Availability

All code used for analysis of this dataset can be found at https://github.com/kleejin/canine_epigenetic_clock. All raw data from sequencing libraries are accessible on NCBI SRA. RRBS data are available as BioProject accession PRJNA1049514 and the ATAC data are available as BioProject accession PRJNA1048909.

## References

[acel14079-bib-0001] Ake Lu, V. P. , Fei, Z. , Raj, K. , & Horvath, S. (2021). Universal DNA methylation age across mammalian tissues. Innovation in Aging, 5, 410.

[acel14079-bib-0002] Allshire, R. C. , & Madhani, H. D. (2018). Ten principles of heterochromatin formation and function. Nature Reviews Molecular Cell Biology, 19, 229–244. 29235574 10.1038/nrm.2017.119 PMC6822695

[acel14079-bib-0003] Bell, C. G. , Lowe, R. , Adams, P. D. , Baccarelli, A. A. , Beck, S. , Bell, J. T. , Christensen, B. C. , Gladyshev, V. N. , Heijmans, B. T. , Horvath, S. , Ideker, T. , Issa, J.‐P. J. , Kelsey, K. T. , Marioni, R. E. , Reik, W. , Relton, C. L. , Schalkwyk, L. C. , Teschendorff, A. E. , Wagner, W. , … Rakyan, V. K. (2019). DNA methylation aging clocks: Challenges and recommendations. Genome Biology, 20, 249. 31767039 10.1186/s13059-019-1824-y PMC6876109

[acel14079-bib-0004] Belsky, D. W. , Caspi, A. , Houts, R. , Cohen, H. J. , Corcoran, D. L. , Danese, A. , Harrington, H. , Israel, S. , Levine, M. E. , Schaefer, J. D. , Sugden, K. , Williams, B. , Yashin, A. I. , Poulton, R. , & Moffitt, T. E. (2015). Quantification of biological aging in young adults. Proceedings of the National Academy of Sciences of the United States of America, 112, E4104–E4110. 26150497 10.1073/pnas.1506264112 PMC4522793

[acel14079-bib-0005] Benjamini, Y. , & Hochberg, Y. (1995). Controlling the false discovery rate: A practical and powerful approach to multiple testing. Journal of the Royal Statistical Society: Series B: Methodological, 57, 289–300.

[acel14079-bib-0006] Blackburn, E. H. , Greider, C. W. , & Szostak, J. W. (2006). Telomeres and telomerase: The path from maize, tetrahymena and yeast to human cancer and aging. Nature Medicine, 12, 1133–1138. 10.1038/nm1006-1133 17024208

[acel14079-bib-0007] Bocklandt, S. , Lin, W. , Sehl, M. E. , Sánchez, F. J. , Sinsheimer, J. S. , Horvath, S. , & Vilain, E. (2011). Epigenetic predictor of age. PLoS One, 6, e14821. 21731603 10.1371/journal.pone.0014821 PMC3120753

[acel14079-bib-0008] Boyko, A. R. , Quignon, P. , Li, L. , Schoenebeck, J. J. , Degenhardt, J. D. , Lohmueller, K. E. , Zhao, K. , Brisbin, A. , Parker, H. G. , vonHoldt, B. M. , Cargill, M. , Auton, A. , Reynolds, A. , Elkahloun, A. G. , Castelhano, M. , Mosher, D. S. , Sutter, N. B. , Johnson, G. S. , Novembre, J. , … Ostrander, E. A. (2010). A simple genetic architecture underlies morphological variation in dogs. PLoS Biology, 8, e1000451. 20711490 10.1371/journal.pbio.1000451 PMC2919785

[acel14079-bib-0009] Boyle, P. , Clement, K. , Gu, H. , Smith, Z. D. , Ziller, M. , Fostel, J. L. , Holmes, L. , Meldrim, J. , Kelley, F. , Gnirke, A. , & Meissner, A. (2012). Gel‐free multiplexed reduced representation bisulfite sequencing for large‐scale DNA methylation profiling. Genome Biology, 13, R92. 23034176 10.1186/gb-2012-13-10-r92 PMC3491420

[acel14079-bib-0010] Buenrostro, J. D. , Giresi, P. G. , Zaba, L. C. , Chang, H. Y. , & Greenleaf, W. J. (2013). Transposition of native chromatin for fast and sensitive epigenomic profiling of open chromatin, DNA‐binding proteins and nucleosome position. Nature Methods, 10, 1213–1218. 24097267 10.1038/nmeth.2688 PMC3959825

[acel14079-bib-0011] Christensen, K. , Iachina, M. , Rexbye, H. , Tomassini, C. , Frederiksen, H. , McGue, M. , & Vaupel, J. W. (2004). “Looking old for your age”: Genetics and mortality. Epidemiology, 15, 251–252. 15127920 10.1097/01.ede.0000112211.11416.a6

[acel14079-bib-0012] Creevy, K. E. , Akey, J. M. , Kaeberlein, M. , Promislow, D. E. L. , & Dog Aging Project Consortium . (2022). An open science study of ageing in companion dogs. Nature, 602, 51–57. 35110758 10.1038/s41586-021-04282-9 PMC8940555

[acel14079-bib-0013] Danecek, P. , Bonfield, J. K. , Liddle, J. , Marshall, J. , Ohan, V. , Pollard, M. O. , Whitwham, A. , Keane, T. , McCarthy, S. A. , Davies, R. M. , & Li, H. (2021). Twelve years of SAMtools and BCFtools. GigaScience, 10, giab008. 33590861 10.1093/gigascience/giab008 PMC7931819

[acel14079-bib-0014] Dang, W. , Steffen, K. K. , Perry, R. , Dorsey, J. A. , Johnson, F. B. , Shilatifard, A. , Kaeberlein, M. , Kennedy, B. K. , & Berger, S. L. (2009). Histone H4 lysine 16 acetylation regulates cellular lifespan. Nature, 459, 802–807. 19516333 10.1038/nature08085 PMC2702157

[acel14079-bib-0015] Davie, K. , Jacobs, J. , Atkins, M. , Potier, D. , Christiaens, V. , Halder, G. , & Aerts, S. (2015). Discovery of transcription factors and regulatory regions driving in vivo tumor development by ATAC‐seq and FAIRE‐seq open chromatin profiling. PLoS Genetics, 11, e1004994. 25679813 10.1371/journal.pgen.1004994 PMC4334524

[acel14079-bib-0016] Fleming, J. M. , Creevy, K. E. , & Promislow, D. E. L. (2011). Mortality in north american dogs from 1984 to 2004: An investigation into age‐, size‐, and breed‐related causes of death. Journal of Veterinary Internal Medicine, 25, 187–198. 21352376 10.1111/j.1939-1676.2011.0695.x

[acel14079-bib-0017] Galis, F. , van der Sluijs, I. , van Dooren, T. J. M. , Metz, J. A. J. , & Nussbaumer, M. (2007). Do large dogs die young? Journal of Experimental Zoology. Part B, Molecular and Developmental Evolution, 308B, 119–126. 10.1002/jez.b.21116 16788896

[acel14079-bib-0018] Gatta, V. , D'Aurora, M. , Granzotto, A. , Stuppia, L. , & Sensi, S. L. (2014). Early and sustained altered expression of aging‐related genes in young 3xTg‐AD mice. Cell Death & Disease, 5, e1054. 24525730 10.1038/cddis.2014.11 PMC3944230

[acel14079-bib-0019] Hannum, G. , Guinney, J. , Zhao, L. , Zhang, L. , Hughes, G. , Sadda, S. , Klotzle, B. , Bibikova, M. , Fan, J.‐B. , Gao, Y. , Deconde, R. , Chen, M. , Rajapakse, I. , Friend, S. , Ideker, T. , & Zhang, K. (2013). Genome‐wide methylation profiles reveal quantitative views of human aging rates. Molecular Cell, 49, 359–367. 23177740 10.1016/j.molcel.2012.10.016 PMC3780611

[acel14079-bib-0020] Hayward, J. J. , Castelhano, M. G. , Oliveira, K. C. , Corey, E. , Balkman, C. , Baxter, T. L. , Casal, M. L. , Center, S. A. , Fang, M. , Garrison, S. J. , Kalla, S. E. , Korniliev, P. , Kotlikoff, M. I. , Moise, N. S. , Shannon, L. M. , Simpson, K. W. , Sutter, N. B. , Todhunter, R. J. , & Boyko, A. R. (2016). Complex disease and phenotype mapping in the domestic dog. Nature Communications, 7, 10460. 10.1038/ncomms10460 PMC4735900 26795439

[acel14079-bib-0021] Hernandez, D. G. , Nalls, M. A. , Gibbs, J. R. , Arepalli, S. , van der Brug, M. , Chong, S. , Moore, M. , Longo, D. L. , Cookson, M. R. , Traynor, B. J. , & Singleton, A. B. (2011). Distinct DNA methylation changes highly correlated with chronological age in the human brain. Human Molecular Genetics, 20, 1164–1172. 21216877 10.1093/hmg/ddq561 PMC3043665

[acel14079-bib-0022] Hertel, J. , Friedrich, N. , Wittfeld, K. , Pietzner, M. , Budde, K. , Van der Auwera, S. , Lohmann, T. , Teumer, A. , Völzke, H. , Nauck, M. , & Grabe, H. J. (2016). Measuring biological age via Metabonomics: The metabolic age score. Journal of Proteome Research, 15, 400–410. 26652958 10.1021/acs.jproteome.5b00561

[acel14079-bib-0023] Hoffman, J. M. , Creevy, K. E. , Franks, A. , O'Neill, D. G. , & Promislow, D. E. L. (2018). The companion dog as a model for human aging and mortality. Aging Cell, 17, e12737. 29457329 10.1111/acel.12737 PMC5946068

[acel14079-bib-0024] Holly, A. C. , Melzer, D. , Pilling, L. C. , Henley, W. , Hernandez, D. G. , Singleton, A. B. , Bandinelli, S. , Guralnik, J. M. , Ferrucci, L. , & Harries, L. W. (2013). Towards a gene expression biomarker set for human biological age. Aging Cell, 12, 324–326. 23311345 10.1111/acel.12044 PMC4623317

[acel14079-bib-0025] Horvath, S. (2013). DNA methylation age of human tissues and cell types. Genome Biology, 14, 3156. 10.1186/gb-2013-14-10-r115 PMC4015143 24138928

[acel14079-bib-0026] Horvath, S. , Erhart, W. , Brosch, M. , Ammerpohl, O. , von Schönfels, W. , Ahrens, M. , Heits, N. , Bell, J. T. , Tsai, P.‐C. , Spector, T. D. , Deloukas, P. , Siebert, R. , Sipos, B. , Becker, T. , Röcken, C. , Schafmayer, C. , & Hampe, J. (2014). Obesity accelerates epigenetic aging of human liver. Proceedings of the National Academy of Sciences of the United States of America, 111, 15538–15543. 25313081 10.1073/pnas.1412759111 PMC4217403

[acel14079-bib-0027] Horvath, S. , Gurven, M. , Levine, M. E. , Trumble, B. C. , Kaplan, H. , Allayee, H. , Ritz, B. R. , Chen, B. , Lu, A. T. , Rickabaugh, T. M. , Jamieson, B. D. , Sun, D. , Li, S. , Chen, W. , Quintana‐Murci, L. , Fagny, M. , Kobor, M. S. , Tsao, P. S. , Reiner, A. P. , … Assimes, T. L. (2016). An epigenetic clock analysis of race/ethnicity, sex, and coronary heart disease. Genome Biology, 17, 171. 27511193 10.1186/s13059-016-1030-0 PMC4980791

[acel14079-bib-0028] Horvath, S. , Lu, A. T. , Haghani, A. , Zoller, J. A. , Li, C. Z. , Lim, A. R. , Brooke, R. T. , Raj, K. , Serres‐Armero, A. , Dreger, D. L. , Hogan, A. N. , Plassais, J. , & Ostrander, E. A. (2022). DNA methylation clocks for dogs and humans. Proceedings of the National Academy of Sciences of the United States of America, 119, e2120887119. 35580182 10.1073/pnas.2120887119 PMC9173771

[acel14079-bib-0029] Ito, H. , Udono, T. , Hirata, S. , & Inoue‐Murayama, M. (2018). Estimation of chimpanzee age based on DNA methylation. Scientific Reports, 8, 9998. 29968770 10.1038/s41598-018-28318-9 PMC6030051

[acel14079-bib-0030] Kaeberlein, M. , Rabinovitch, P. S. , & Martin, G. M. (2015). Healthy aging: The ultimate preventative medicine. Science, 350, 1191–1193. 26785476 10.1126/science.aad3267 PMC4793924

[acel14079-bib-0031] Kakebeen, A. D. , Chitsazan, A. D. , Williams, M. C. , Saunders, L. M. , & Wills, A. E. (2020). In M. E. Bronner , J. R. Monaghan , & A. Sater (Eds.), Chromatin accessibility dynamics and single cell RNA‐seq reveal new regulators of regeneration in neural progenitors (Vol. 9, e52648). eLife. 10.7554/eLife.52648 PMC7250574 32338593

[acel14079-bib-0032] Kim, C. , Kong, G. , Lee, H. , Tran, Q. , Vo, T.‐T. T. , Kwon, S. H. , Park, J. , Kim, S.‐H. , & Park, J. (2022). Scavenger receptor class F member 2 (SCARF2) as a novel therapeutic target in glioblastoma. Toxicology Research, 38, 249–256. 10.1007/s43188-022-00125-5 PMC8960497 35419275

[acel14079-bib-0033] Kim, M.‐H. , Choi, J. , Yang, J. , Chung, W. , Kim, J.‐H. , Paik, S. K. , Kim, K. , Han, S. , Won, H. , Bae, Y.‐S. , Cho, S.‐H. , Seo, J. , Bae, Y. C. , Choi, S.‐Y. , & Kim, E. (2009). Enhanced NMDA receptor‐mediated synaptic transmission, enhanced long‐term potentiation, and impaired learning and memory in mice lacking IRSp53. The Journal of Neuroscience, 29, 1586–1595. 19193906 10.1523/JNEUROSCI.4306-08.2009 PMC6666077

[acel14079-bib-0034] Kraus, C. , Pavard, S. , & Promislow, D. E. L. (2013). The size‐life span trade‐off decomposed: Why large dogs die young. The American Naturalist, 181, 492–505. 10.1086/669665 23535614

[acel14079-bib-0035] Krištić, J. , Vučković, F. , Menni, C. , Klarić, L. , Keser, T. , Beceheli, I. , Pučić‐Baković, M. , Novokmet, M. , Mangino, M. , Thaqi, K. , Rudan, P. , Novokmet, N. , Sarac, J. , Missoni, S. , Kolčić, I. , Polašek, O. , Rudan, I. , Campbell, H. , Hayward, C. , … Lauc, G. (2014). Glycans are a novel biomarker of chronological and biological ages. The Journals of Gerontology. Series A, Biological Sciences and Medical Sciences, 69, 779–789. 24325898 10.1093/gerona/glt190 PMC4049143

[acel14079-bib-0036] Langmead, B. , & Salzberg, S. L. (2012). Fast gapped‐read alignment with bowtie 2. Nature Methods, 9, 357–359. 22388286 10.1038/nmeth.1923 PMC3322381

[acel14079-bib-0037] Levine, M. E. (2013). Modeling the rate of senescence: Can estimated biological age predict mortality more accurately than chronological age? The Journals of Gerontology. Series A, Biological Sciences and Medical Sciences, 68, 667–674. 23213031 10.1093/gerona/gls233 PMC3660119

[acel14079-bib-0038] Li, Q. , Wang, S. , Milot, E. , Bergeron, P. , Ferrucci, L. , Fried, L. P. , & Cohen, A. A. (2015). Homeostatic dysregulation proceeds in parallel in multiple physiological systems. Aging Cell, 14, 1103–1112. 26416593 10.1111/acel.12402 PMC4693454

[acel14079-bib-0039] Lindblad‐Toh, K. , Wade, C. M. , Mikkelsen, T. S. , Karlsson, E. K. , Jaffe, D. B. , Kamal, M. , Clamp, M. , Chang, J. L. , Kulbokas, E. J. , Zody, M. C. , Mauceli, E. , Xie, X. , Breen, M. , Wayne, R. K. , Ostrander, E. A. , Ponting, C. P. , Galibert, F. , Smith, D. R. , DeJong, P. J. , … Lander, E. S. (2005). Genome sequence, comparative analysis and haplotype structure of the domestic dog. Nature, 438, 803–819. 16341006 10.1038/nature04338

[acel14079-bib-0040] MacLean, E. L. , Snyder‐Mackler, N. , vonHoldt, B. M. , & Serpell, J. A. (2019). Highly heritable and functionally relevant breed differences in dog behaviour. Proceedings of the Biological Sciences, 286, 20190716. 31575369 10.1098/rspb.2019.0716 PMC6790757

[acel14079-bib-0041] Maegawa, S. , Hinkal, G. , Kim, H. S. , Shen, L. , Zhang, L. , Zhang, J. , Zhang, N. , Liang, S. , Donehower, L. A. , & Issa, J.‐P. J. (2010). Widespread and tissue specific age‐related DNA methylation changes in mice. Genome Research, 20, 332–340. 20107151 10.1101/gr.096826.109 PMC2840983

[acel14079-bib-0042] Marioni, R. E. , Shah, S. , McRae, A. F. , Chen, B. H. , Colicino, E. , Harris, S. E. , Gibson, J. , Henders, A. K. , Redmond, P. , Cox, S. R. , Pattie, A. , Corley, J. , Murphy, L. , Martin, N. G. , Montgomery, G. W. , Feinberg, A. P. , Fallin, M. D. , Multhaup, M. L. , Jaffe, A. E. , … Deary, I. J. (2015). DNA methylation age of blood predicts all‐cause mortality in later life. Genome Biology, 16, 25. 25633388 10.1186/s13059-015-0584-6 PMC4350614

[acel14079-bib-0043] Meissner, A. , Gnirke, A. , Bell, G. W. , Ramsahoye, B. , Lander, E. S. , & Jaenisch, R. (2005). Reduced representation bisulfite sequencing for comparative high‐resolution DNA methylation analysis. Nucleic Acids Research, 33, 5868–5877. 16224102 10.1093/nar/gki901 PMC1258174

[acel14079-bib-0044] Menni, C. , Kiddle, S. J. , Mangino, M. , Viñuela, A. , Psatha, M. , Steves, C. , Sattlecker, M. , Buil, A. , Newhouse, S. , Nelson, S. , Williams, S. , Voyle, N. , Soininen, H. , Kloszewska, I. , Mecocci, P. , Tsolaki, M. , Vellas, B. , Lovestone, S. , Spector, T. D. , … Valdes, A. M. (2015). Circulating proteomic signatures of chronological age. The Journals of Gerontology. Series A, Biological Sciences and Medical Sciences, 70, 809–816. 25123647 10.1093/gerona/glu121 PMC4469006

[acel14079-bib-0045] Moskowitz, D. M. , Zhang, D. W. , Hu, B. , Le Saux, S. , Yanes, R. E. , Ye, Z. , Buenrostro, J. D. , Weyand, C. M. , Greenleaf, W. J. , & Goronzy, J. J. (2017). Epigenomics of human CD8 T cell differentiation and aging. Science Immunology, 2, eaag0192. 28439570 10.1126/sciimmunol.aag0192 PMC5399889

[acel14079-bib-0046] Nakajima, Y. , Chamoto, K. , Oura, T. , & Honjo, T. (2021). Critical role of the CD44lowCD62Llow CD8+ T cell subset in restoring antitumor immunity in aged mice. Proceedings of the National Academy of Sciences of the United States of America, 118, e2103730118. 34088845 10.1073/pnas.2103730118 PMC8201912

[acel14079-bib-0047] Oberdoerffer, P. , & Sinclair, D. A. (2007). The role of nuclear architecture in genomic instability and ageing. Nature Reviews. Molecular Cell Biology, 8, 692–702. 17700626 10.1038/nrm2238

[acel14079-bib-0048] Ostrander, E. A. , & Kruglyak, L. (2000). Unleashing the canine genome. Genome Research, 10, 1271–1274. 10984444 10.1101/gr.155900

[acel14079-bib-0049] O'Sullivan, R. J. , Kubicek, S. , Schreiber, S. L. , & Karlseder, J. (2010). Reduced histone biosynthesis and chromatin changes arising from a damage signal at telomeres. Nature Structural & Molecular Biology, 17, 1218–1225. 10.1038/nsmb.1897 PMC2951278 20890289

[acel14079-bib-0050] Parker, H. G. , Kim, L. V. , Sutter, N. B. , Carlson, S. , Lorentzen, T. D. , Malek, T. B. , Johnson, G. S. , DeFrance, H. B. , Ostrander, E. A. , & Kruglyak, L. (2004). Genetic structure of the purebred domestic dog. Science, 304, 1160–1164. 15155949 10.1126/science.1097406

[acel14079-bib-0051] Patronek, G. J. , Waters, D. J. , & Glickman, L. T. (1997). Comparative longevity of pet dogs and humans: Implications for gerontology research. The Journals of Gerontology. Series A, Biological Sciences and Medical Sciences, 52, B171–B178. 9158552 10.1093/gerona/52a.3.b171

[acel14079-bib-0052] Perna, L. , Zhang, Y. , Mons, U. , Holleczek, B. , Saum, K.‐U. , & Brenner, H. (2016). Epigenetic age acceleration predicts cancer, cardiovascular, and all‐cause mortality in a German case cohort. Clinical Epigenetics, 8, 64. 27274774 10.1186/s13148-016-0228-z PMC4891876

[acel14079-bib-0053] Peters, M. J. , Joehanes, R. , Pilling, L. C. , Schurmann, C. , Conneely, K. N. , Powell, J. , Reinmaa, E. , Sutphin, G. L. , Zhernakova, A. , Schramm, K. , Wilson, Y. A. , Kobes, S. , Tukiainen, T. , Ramos, Y. F. , Göring, H. H. H. , Fornage, M. , Liu, Y. , Gharib, S. A. , Stranger, B. E. , … Johnson, A. D. (2015). The transcriptional landscape of age in human peripheral blood. Nature Communications, 6, 8570. 10.1038/ncomms9570 PMC4639797 26490707

[acel14079-bib-0054] Polanowski, A. M. , Robbins, J. , Chandler, D. , & Jarman, S. N. (2014). Epigenetic estimation of age in humpback whales. Molecular Ecology Resources, 14, 976–987. 24606053 10.1111/1755-0998.12247 PMC4314680

[acel14079-bib-0055] Quach, A. , Levine, M. E. , Tanaka, T. , Lu, A. T. , Chen, B. H. , Ferrucci, L. , Ritz, B. , Bandinelli, S. , Neuhouser, M. L. , Beasley, J. M. , Snetselaar, L. , Wallace, R. B. , Tsao, P. S. , Absher, D. , Assimes, T. L. , Stewart, J. D. , Li, Y. , Hou, L. , Baccarelli, A. A. , … Horvath, S. (2017). Epigenetic clock analysis of diet, exercise, education, and lifestyle factors. Aging, 9, 419–446. 28198702 10.18632/aging.101168 PMC5361673

[acel14079-bib-0056] R Core Team . (2018). R: A language and environment for statistical computing. R Foundation for Statistical Computing.

[acel14079-bib-0057] Rakyan, V. K. , Down, T. A. , Maslau, S. , Andrew, T. , Yang, T.‐P. , Beyan, H. , Whittaker, P. , McCann, O. T. , Finer, S. , Valdes, A. M. , Leslie, R. D. , Deloukas, P. , & Spector, T. D. (2010). Human aging‐associated DNA hypermethylation occurs preferentially at bivalent chromatin domains. Genome Research, 20, 434–439. 20219945 10.1101/gr.103101.109 PMC2847746

[acel14079-bib-0058] Rubbi, L. , Zhang, H. , Feng, J. , He, C. , Kurnia, P. , Ratan, P. , Tammana, A. , House, S. , Thompson, M. , Farrell, C. , Snir, S. , Stahler, D. , Ostrander, E. A. , vonHoldt, B. M. , & Pellegrini, M. (2022). The effects of age, sex, weight, and breed on canid methylomes. Epigenetics, 17, 1497–1512. 35502722 10.1080/15592294.2022.2069385 PMC9586589

[acel14079-bib-0059] Sallusto, F. , Geginat, J. , & Lanzavecchia, A. (2004). Central memory and effector memory T cell subsets: Function, generation, and maintenance. Annual Review of Immunology, 22, 745–763. 10.1146/annurev.immunol.22.012703.104702 15032595

[acel14079-bib-0060] Saule, P. , Trauet, J. , Dutriez, V. , Lekeux, V. , Dessaint, J.‐P. , & Labalette, M. (2006). Accumulation of memory T cells from childhood to old age: Central and effector memory cells in CD4(+) versus effector memory and terminally differentiated memory cells in CD8(+) compartment. Mechanisms of Ageing and Development, 127, 274–281. 16352331 10.1016/j.mad.2005.11.001

[acel14079-bib-0061] Sen, P. , Shah, P. P. , Nativio, R. , & Berger, S. L. (2016). Epigenetic mechanisms of longevity and aging. Cell, 166, 822–839. 27518561 10.1016/j.cell.2016.07.050 PMC5821249

[acel14079-bib-0062] Son, K. H. , Aldonza, M. B. D. , Nam, A.‐R. , Lee, K.‐H. , Lee, J.‐W. , Shin, K.‐J. , Kang, K. , & Cho, J.‐Y. (2023). Integrative mapping of the dog epigenome: Reference annotation for comparative intertissue and cross‐species studies. Science Advances, 9, eade3399. 37406108 10.1126/sciadv.ade3399 PMC10321747

[acel14079-bib-0063] Sutter, N. B. , Bustamante, C. D. , Chase, K. , Gray, M. M. , Zhao, K. , Zhu, L. , Padhukasahasram, B. , Karlins, E. , Davis, S. , Jones, P. G. , Quignon, P. , Johnson, G. S. , Parker, H. G. , Fretwell, N. , Mosher, D. S. , Lawler, D. F. , Satyaraj, E. , Nordborg, M. , Lark, K. G. , … Ostrander, E. A. (2007). A single IGF1 allele is a major determinant of small size in dogs. Science, 316, 112–115. 17412960 10.1126/science.1137045 PMC2789551

[acel14079-bib-0064] Sziráki, A. , Tyshkovskiy, A. , & Gladyshev, V. N. (2018). Global remodeling of the mouse DNA methylome during aging and in response to calorie restriction. Aging Cell, 17, e12738. 29575528 10.1111/acel.12738 PMC5946071

[acel14079-bib-0065] Teschendorff, A. E. , Menon, U. , Gentry‐Maharaj, A. , Ramus, S. J. , Weisenberger, D. J. , Shen, H. , Campan, M. , Noushmehr, H. , Bell, C. G. , Maxwell, A. P. , Savage, D. A. , Mueller‐Holzner, E. , Marth, C. , Kocjan, G. , Gayther, S. A. , Jones, A. , Beck, S. , Wagner, W. , Laird, P. W. , … Widschwendter, M. (2010). Age‐dependent DNA methylation of genes that are suppressed in stem cells is a hallmark of cancer. Genome Research, 20, 440–446. 20219944 10.1101/gr.103606.109 PMC2847747

[acel14079-bib-0066] Thompson, M. J. , vonHoldt, B. , Horvath, S. , & Pellegrini, M. (2017). An epigenetic aging clock for dogs and wolves. Aging, 9, 1055–1068. 28373601 10.18632/aging.101211 PMC5391218

[acel14079-bib-0067] Trojer, P. , & Reinberg, D. (2007). Facultative heterochromatin: Is there a distinctive molecular signature? Molecular Cell, 28, 1–13. 17936700 10.1016/j.molcel.2007.09.011

[acel14079-bib-0068] Villeponteau, B. (1997). The heterochromatin loss model of aging. Experimental Gerontology, 32, 383–394. 9315443 10.1016/s0531-5565(96)00155-6

[acel14079-bib-0069] Wang, J. , Zibetti, C. , Shang, P. , Sripathi, S. R. , Zhang, P. , Cano, M. , Hoang, T. , Xia, S. , Ji, H. , Merbs, S. L. , Zack, D. J. , Handa, J. T. , Sinha, D. , Blackshaw, S. , & Qian, J. (2018). ATAC‐seq analysis reveals a widespread decrease of chromatin accessibility in age‐related macular degeneration. Nature Communications, 9, 1364. 10.1038/s41467-018-03856-y PMC5893535 29636475

[acel14079-bib-0070] Wang, T. , Ma, J. , Hogan, A. N. , Fong, S. , Licon, K. , Tsui, B. , Kreisberg, J. F. , Adams, P. D. , Carvunis, A.‐R. , Bannasch, D. L. , Ostrander, E. A. , & Ideker, T. (2020). Quantitative translation of dog‐to‐human aging by conserved remodeling of the DNA methylome. Cell Systems, 11, 176–185. 32619550 10.1016/j.cels.2020.06.006 PMC7484147

[acel14079-bib-0071] Withers, S. S. , Moore, P. F. , Chang, H. , Choi, J. W. , McSorley, S. J. , Kent, M. S. , Monjazeb, A. M. , Canter, R. J. , Murphy, W. J. , Sparger, E. E. , & Rebhun, R. B. (2018). Multi‐color flow cytometry for evaluating age‐related changes in memory lymphocyte subsets in dogs. Developmental and Comparative Immunology, 87, 64–74. 29859828 10.1016/j.dci.2018.05.022 PMC6197816

[acel14079-bib-0072] Wu, J. , Huang, B. , Chen, H. , Yin, Q. , Liu, Y. , Xiang, Y. , Zhang, B. , Liu, B. , Wang, Q. , Xia, W. , Li, W. , Li, Y. , Ma, J. , Peng, X. , Zheng, H. , Ming, J. , Zhang, W. , Zhang, J. , Tian, G. , … Xie, W. (2016). The landscape of accessible chromatin in mammalian preimplantation embryos. Nature, 534, 652–657. 27309802 10.1038/nature18606

[acel14079-bib-0073] Zhao, X. , Golic, F. T. , Harrison, B. R. , Manoj, M. , Hoffman, E. V. , Simon, N. , Johnson, R. , MacCoss, M. J. , McIntyre, L. M. , & Promislow, D. E. L. (2022). The metabolome as a biomarker of aging in Drosophila melanogaster. Aging Cell, 21, e13548. 35019203 10.1111/acel.13548 PMC8844127

