# Supplementary material for: DNA methylation and chromatin accessibility predict age in the domestic dog
Source: Aging Cell. 2024 Jan 23;23(4):e14079. doi: 10.1111/acel.14079 (PMC11019125; doi:10.1111/acel.14079)
Supplement: Supplementary file 2 — Data S2. [file ACEL-23-e14079-s004.pdf]

504
24-JUL-2018

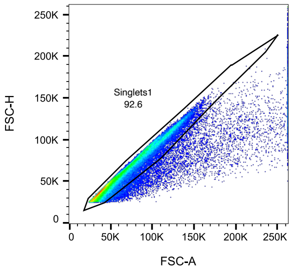

[20180724 Batch1_504_012.fcs]
Ungated
88958

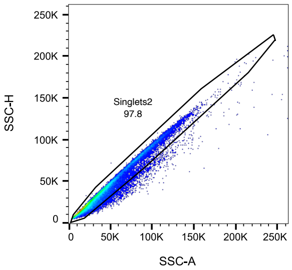

[20180724 Batch1_504_012.fcs]
Singlets1
82369

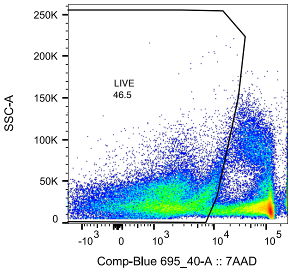

[20180724 Batch1_504_012.fcs]
Singlets2
80528

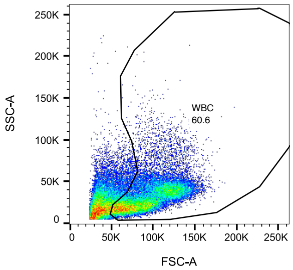

[20180724 Batch1_504_012.fcs]
LIVE
37412

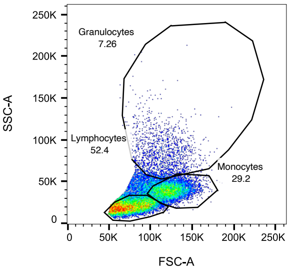

[20180724 Batch1_504_012.fcs]
WBC
22655

Granulocytes

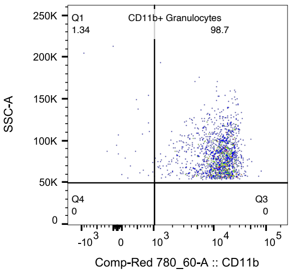

[20180724 Batch1_504_012.fcs]
Granulocytes
1645

Monocytes

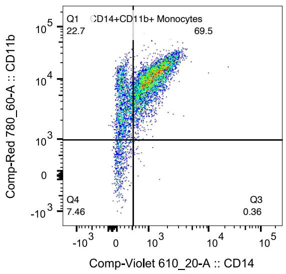

[20180724 Batch1_504_012.fcs]
Monocytes
6623

Lymphocytes

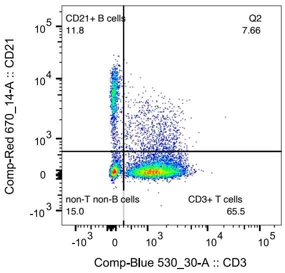

[20180724 Batch1_504_012.fcs]
Lymphocytes
11862

non-T non-B

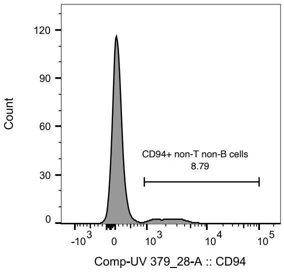

[20180724 Batch1_504_012.fcs]
non-T non-B cells
1775

504
24-JUL-2018

T cells

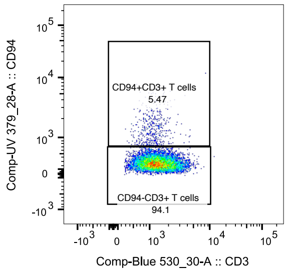

[20180724 Batch1_504_012.fcs]
CD3+ T cells
7775

CD94- T cells

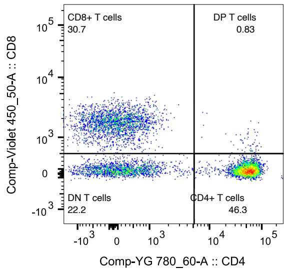

[20180724 Batch1_504_012.fcs]
CD94-CD3+ T cells
7314

CD4 T cells

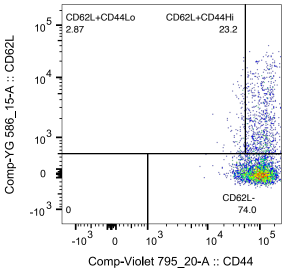

[20180724 Batch1_504_012.fcs]
CD4+ T cells
3385

CD4 T cells

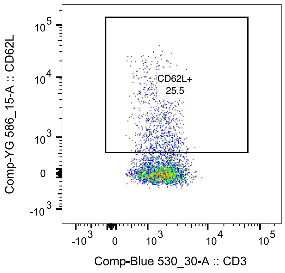

[20180724 Batch1_504_012.fcs]
CD4+ T cells
3385

CD8 T cells

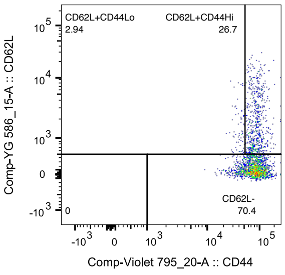

[20180724 Batch1_504_012.fcs]
CD8+ T cells
2242

CD8 T cells

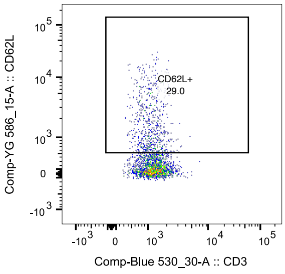

[20180724 Batch1_504_012.fcs]
CD8+ T cells
2242

DN T cells

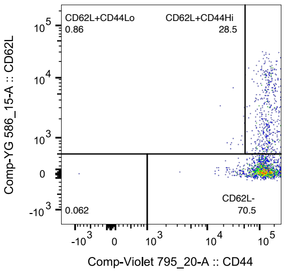

[20180724 Batch1_504_012.fcs]
DN T cells
1626

DN T cells

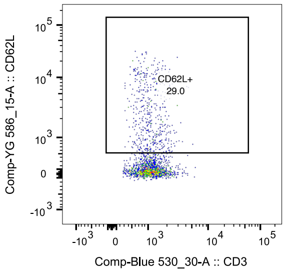

[20180724 Batch1_504_012.fcs]
DN T cells
1626

DP T cells

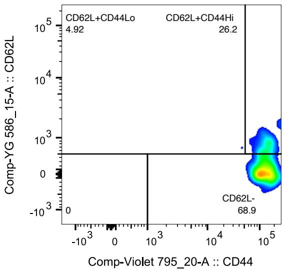

[20180724 Batch1_504_012.fcs]
DP T cells
61.0

DP T cells

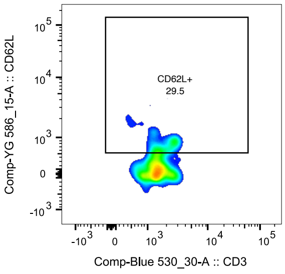

[20180724 Batch1_504_012.fcs]
DP T cells
61.0

505
24-JUL-2018

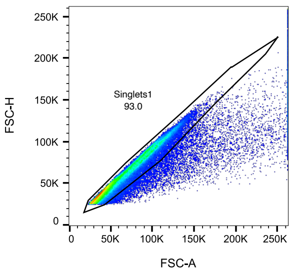

[20180724 Batch1_505_013.fcs]
Ungated
124982

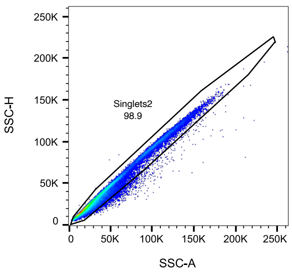

[20180724 Batch1_505_013.fcs]
Singlets1
116256

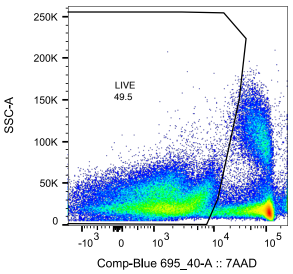

[20180724 Batch1_505_013.fcs]
Singlets2
114984

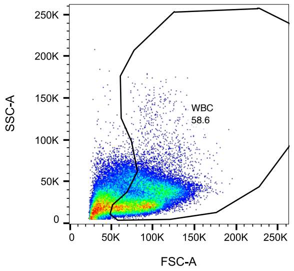

[20180724 Batch1_505_013.fcs]
LIVE
56932

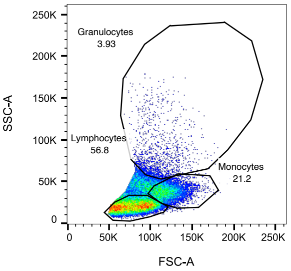

[20180724 Batch1_505_013.fcs]
WBC
33388

Granulocytes

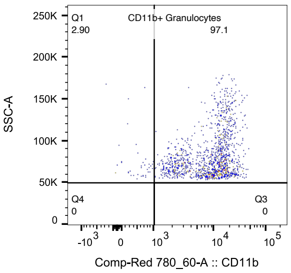

[20180724 Batch1_505_013.fcs]
Granulocytes
1311

Monocytes

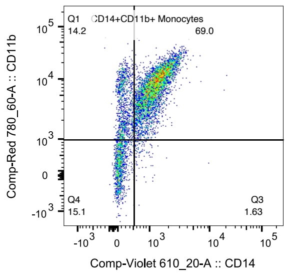

[20180724 Batch1_505_013.fcs]
Monocytes
7065

Lymphocytes

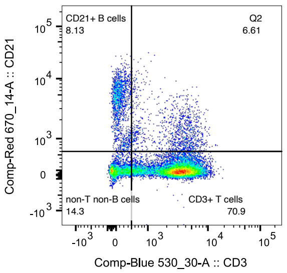

[20180724 Batch1_505_013.fcs]
Lymphocytes
18972

non-T non-B

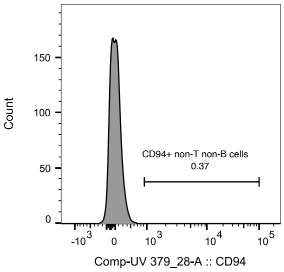

[20180724 Batch1_505_013.fcs]
non-T non-B cells
2721

505
24-JUL-2018

T cells

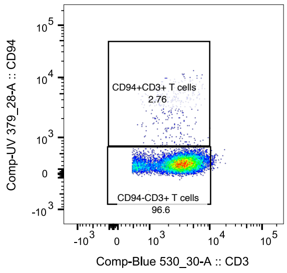

[20180724 Batch1_505_013.fcs]
CD3+ T cells
13454

CD94- T cells

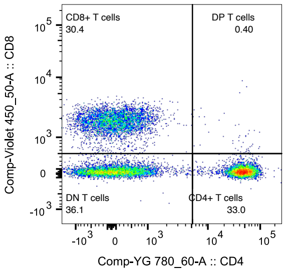

[20180724 Batch1_505_013.fcs]
CD94-CD3+ T cells
12991

CD4 T cells

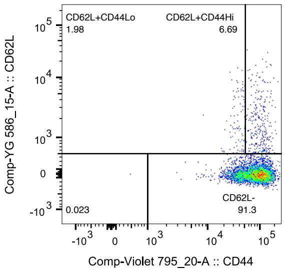

[20180724 Batch1_505_013.fcs]
CD4+ T cells
4289

CD4 T cells

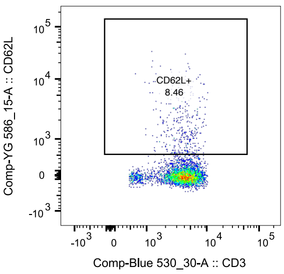

[20180724 Batch1_505_013.fcs]
CD4+ T cells
4289

CD8 T cells

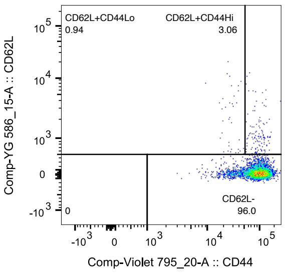

[20180724 Batch1_505_013.fcs]
CD8+ T cells
3955

CD8 T cells

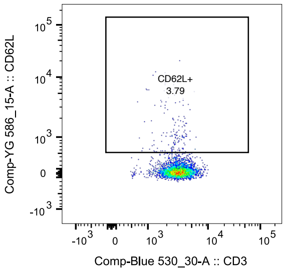

[20180724 Batch1_505_013.fcs]
CD8+ T cells
3955

DN T cells

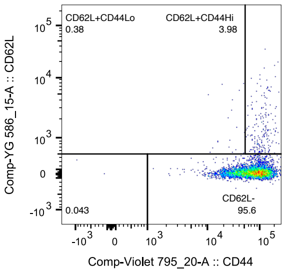

[20180724 Batch1_505_013.fcs]
DN T cells
4695

DN T cells

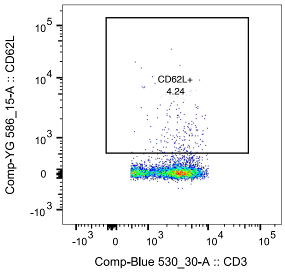

[20180724 Batch1_505_013.fcs]
DN T cells
4695

DP T cells

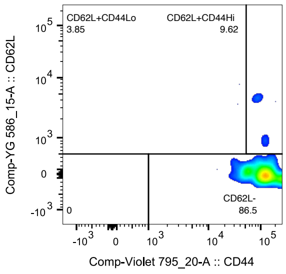

[20180724 Batch1_505_013.fcs]
DP T cells
52.0

DP T cells

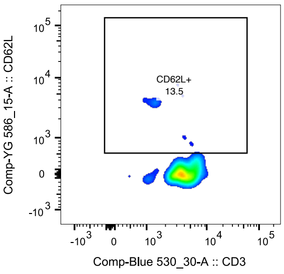

[20180724 Batch1_505_013.fcs]
DP T cells
52.0

544
24-JUL-2018

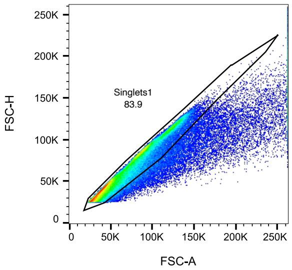

[20180724 Batch1_544_014.fcs]
Ungated
112860

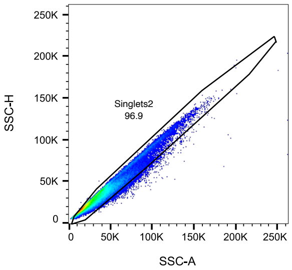

[20180724 Batch1_544_014.fcs]
Singlets1
94742

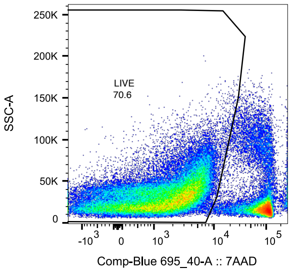

[20180724 Batch1_544_014.fcs]
Singlets2
91816

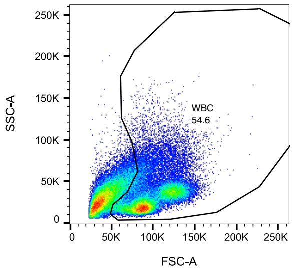

[20180724 Batch1_544_014.fcs]
LIVE
64868

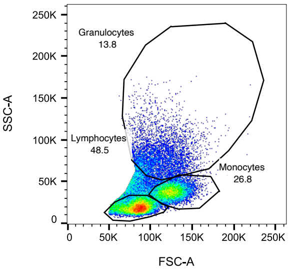

[20180724 Batch1_544_014.fcs]
WBC
35446

Granulocytes

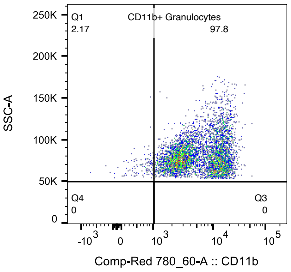

[20180724 Batch1_544_014.fcs]
Granulocytes
4877

Monocytes

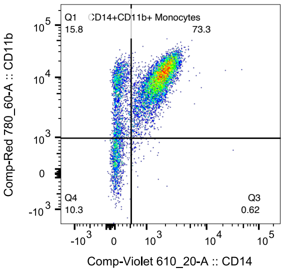

[20180724 Batch1_544_014.fcs]
Monocytes
9503

Lymphocytes

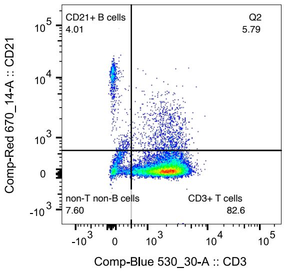

[20180724 Batch1_544_014.fcs]
Lymphocytes
17188

non-T non-B

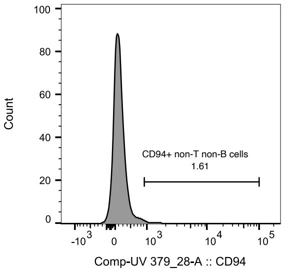

[20180724 Batch1_544_014.fcs]
non-T non-B cells
1306

544
24-JUL-2018

T cells

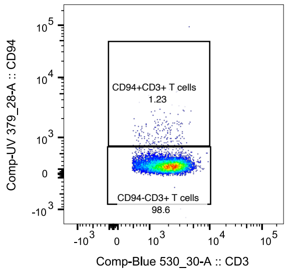

[20180724 Batch1_544_014.fcs]
CD3+ T cells
14196

CD94- T cells

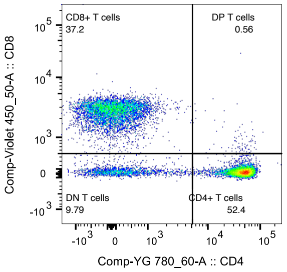

[20180724 Batch1_544_014.fcs]
CD94-CD3+ T cells
13998

CD4 T cells

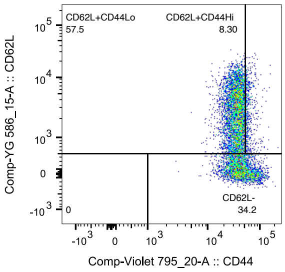

[20180724 Batch1_544_014.fcs]
CD4+ T cells
7338

CD4 T cells

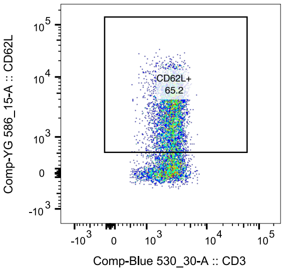

[20180724 Batch1_544_014.fcs]
CD4+ T cells
7338

CD8 T cells

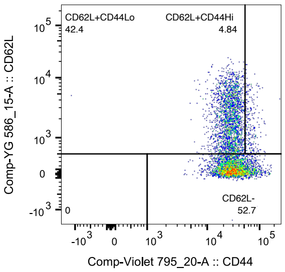

[20180724 Batch1_544_014.fcs]
CD8+ T cells
5211

CD8 T cells

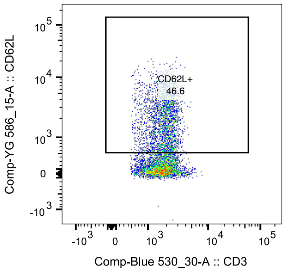

[20180724 Batch1_544_014.fcs]
CD8+ T cells
5211

DN T cells

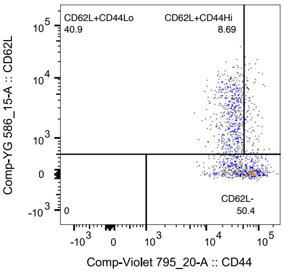

[20180724 Batch1_544_014.fcs]
DN T cells
1370

DN T cells

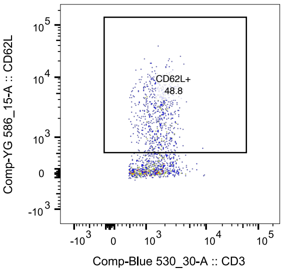

[20180724 Batch1_544_014.fcs]
DN T cells
1370

DP T cells

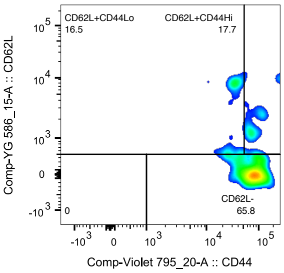

[20180724 Batch1_544_014.fcs]
DP T cells
79.0

DP T cells

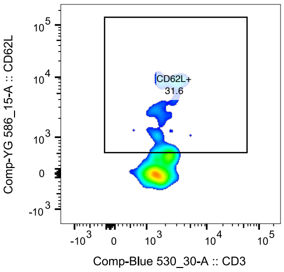

[20180724 Batch1_544_014.fcs]
DP T cells
79.0

546
24-JUL-2018

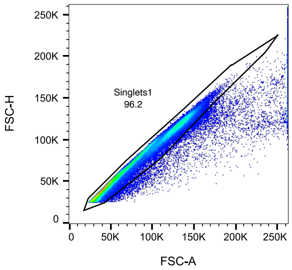

[20180724 Batch1_546_015.fcs]
Ungated
168321

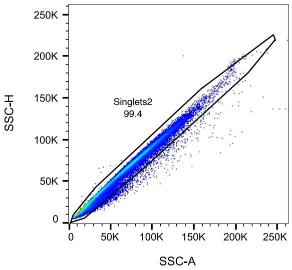

[20180724 Batch1_546_015.fcs]
Singlets1
162002

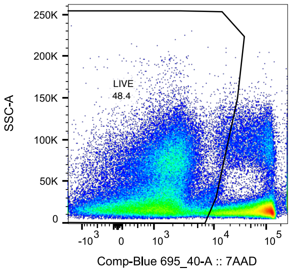

[20180724 Batch1_546_015.fcs]
Singlets2
160989

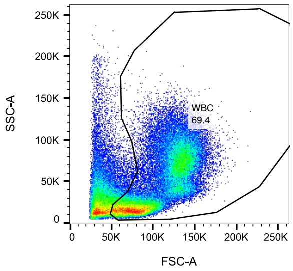

[20180724 Batch1_546_015.fcs]
LIVE
77869

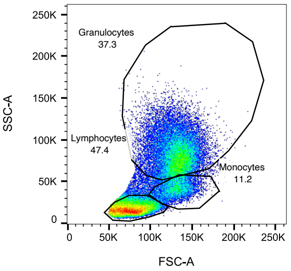

[20180724 Batch1_546_015.fcs]
WBC
54063

Granulocytes

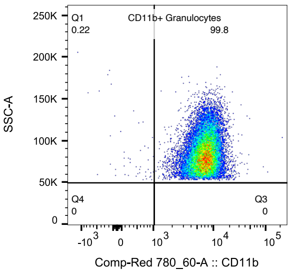

[20180724 Batch1_546_015.fcs]
Granulocytes
20144

Monocytes

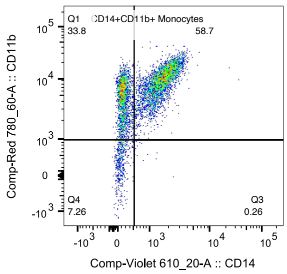

[20180724 Batch1_546_015.fcs]
Monocytes
6060

Lymphocytes

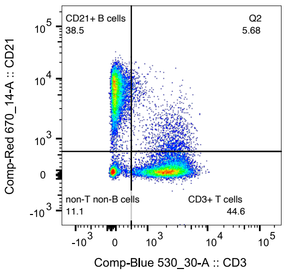

[20180724 Batch1_546_015.fcs]
Lymphocytes
25602

non-T non-B

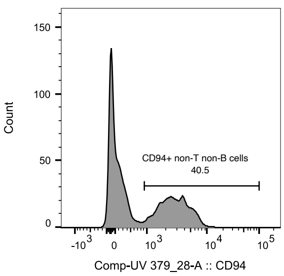

[20180724 Batch1_546_015.fcs]
non-T non-B cells
2854

546
24-JUL-2018

T cells

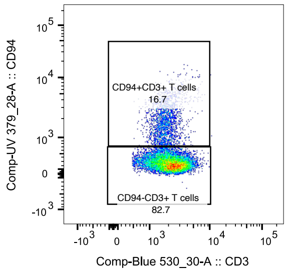

[20180724 Batch1_546_015.fcs]
CD3+ T cells
11427

CD94- T cells

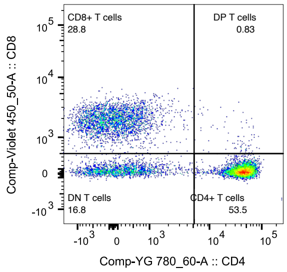

[20180724 Batch1_546_015.fcs]
CD94-CD3+ T cells
9453

CD4 T cells

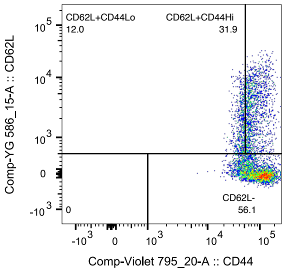

[20180724 Batch1_546_015.fcs]
CD4+ T cells
5062

CD4 T cells

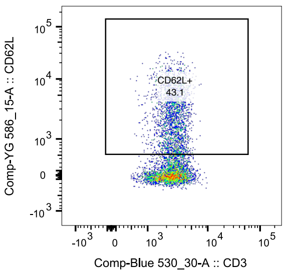

[20180724 Batch1_546_015.fcs]
CD4+ T cells
5062

CD8 T cells

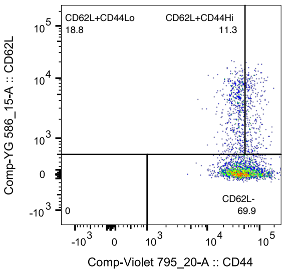

[20180724 Batch1_546_015.fcs]
CD8+ T cells
2722

CD8 T cells

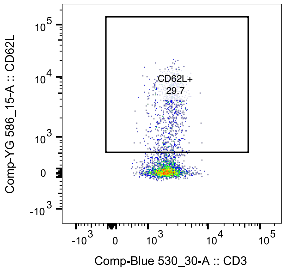

[20180724 Batch1_546_015.fcs]
CD8+ T cells
2722

DN T cells

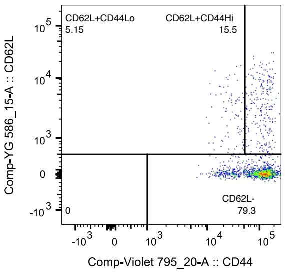

[20180724 Batch1_546_015.fcs]
DN T cells
1591

DN T cells

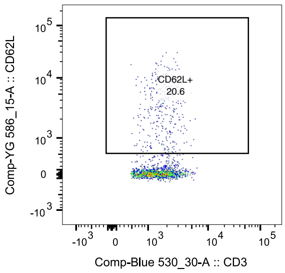

[20180724 Batch1_546_015.fcs]
DN T cells
1591

DP T cells

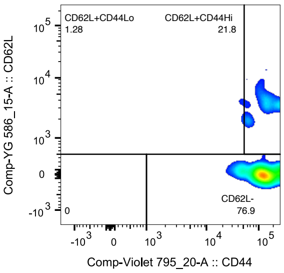

[20180724 Batch1_546_015.fcs]
DP T cells
78.0

DP T cells

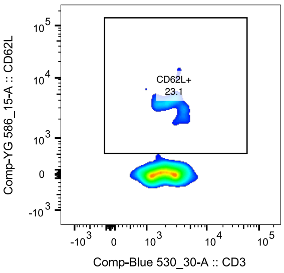

[20180724 Batch1_546_015.fcs]
DP T cells
78.0

553
24-JUL-2018

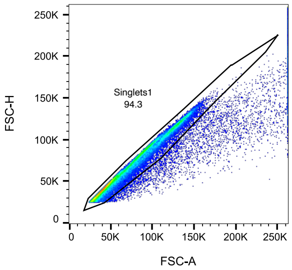

[20180724 Batch1_553_016.fcs]
Ungated
75463

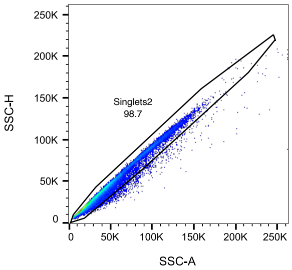

[20180724 Batch1_553_016.fcs]
Singlets1
71176

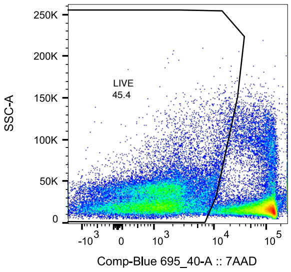

[20180724 Batch1_553_016.fcs]
Singlets2
70273

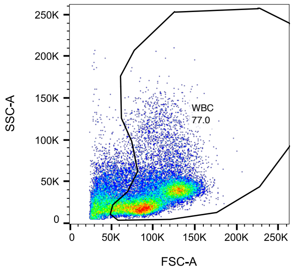

[20180724 Batch1_553_016.fcs]
LIVE
31915

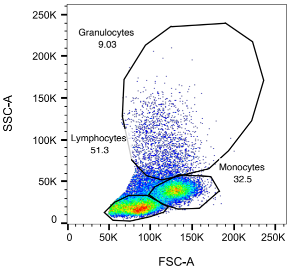

[20180724 Batch1_553_016.fcs]
WBC
24583

Granulocytes

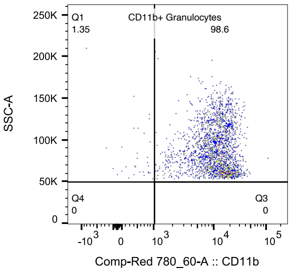

[20180724 Batch1_553_016.fcs]
Granulocytes
2220

Monocytes

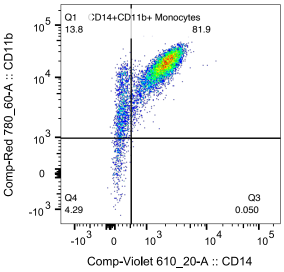

[20180724 Batch1_553_016.fcs]
Monocytes
7980

Lymphocytes

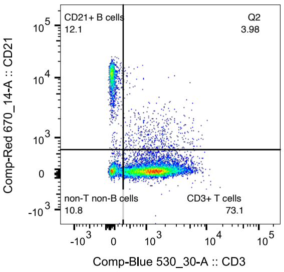

[20180724 Batch1_553_016.fcs]
Lymphocytes
12619

non-T non-B

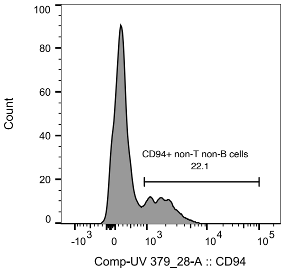

[20180724 Batch1_553_016.fcs]
non-T non-B cells
1360

553
24-JUL-2018

T cells

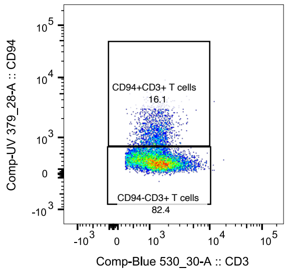

[20180724 Batch1_553_016.fcs]
CD3+ T cells
9229

CD94- T cells

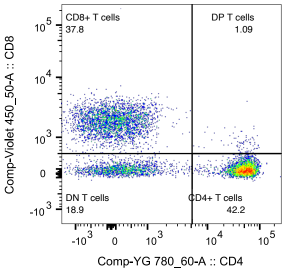

[20180724 Batch1_553_016.fcs]
CD94-CD3+ T cells
7607

CD4 T cells

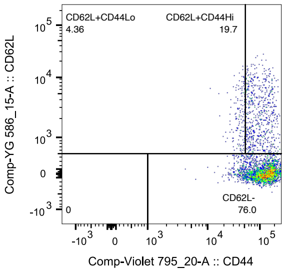

[20180724 Batch1_553_016.fcs]
CD4+ T cells
3208

CD4 T cells

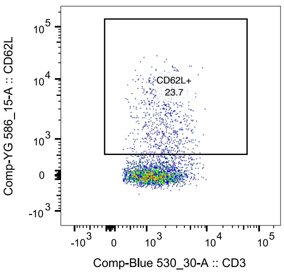

[20180724 Batch1_553_016.fcs]
CD4+ T cells
3208

CD8 T cells

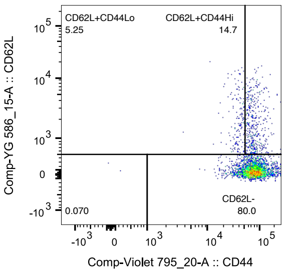

[20180724 Batch1_553_016.fcs]
CD8+ T cells
2877

CD8 T cells

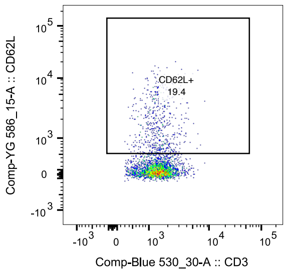

[20180724 Batch1_553_016.fcs]
CD8+ T cells
2877

DN T cells

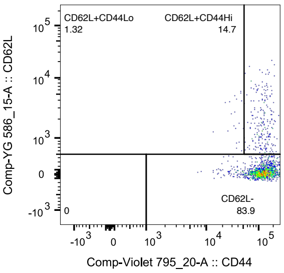

[20180724 Batch1_553_016.fcs]
DN T cells
1439

DN T cells

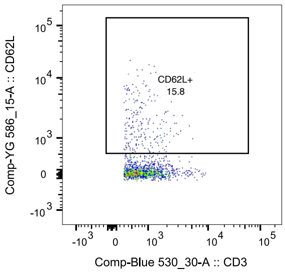

[20180724 Batch1_553_016.fcs]
DN T cells
1439

DP T cells

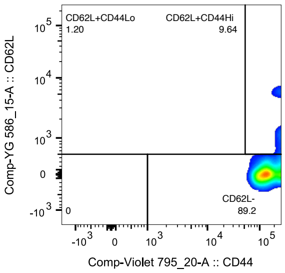

[20180724 Batch1_553_016.fcs]
DP T cells
83.0

DP T cells

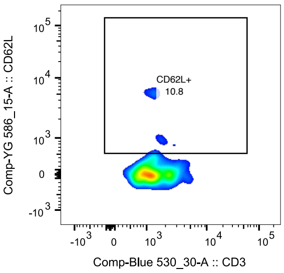

[20180724 Batch1_553_016.fcs]
DP T cells
83.0

564
24-JUL-2018

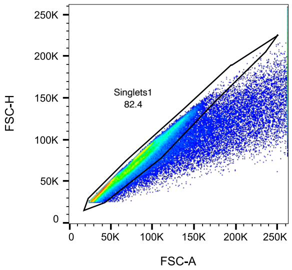

[20180724 Batch1_564_017.fcs]
Ungated
145816

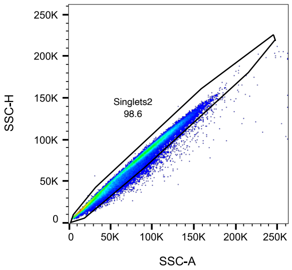

[20180724 Batch1_564_017.fcs]
Singlets1
120175

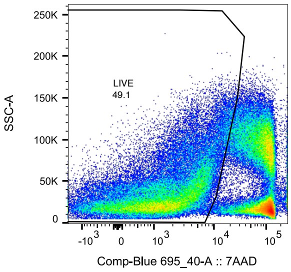

[20180724 Batch1_564_017.fcs]
Singlets2
118471

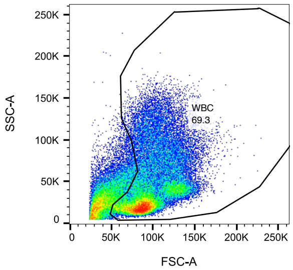

[20180724 Batch1_564_017.fcs]
LIVE
58123

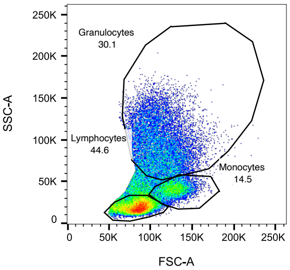

[20180724 Batch1_564_017.fcs]
WBC
40289

Granulocytes

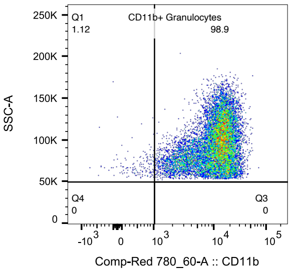

[20180724 Batch1_564_017.fcs]
Granulocytes
12141

Monocytes

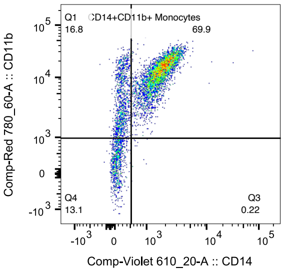

[20180724 Batch1_564_017.fcs]
Monocytes
5861

Lymphocytes

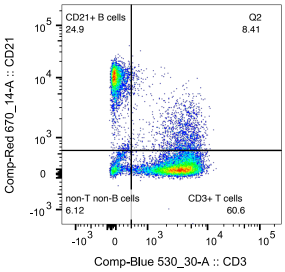

[20180724 Batch1_564_017.fcs]
Lymphocytes
17987

non-T non-B

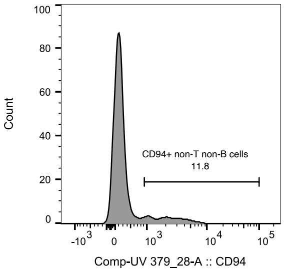

[20180724 Batch1_564_017.fcs]
non-T non-B cells
1101

564
24-JUL-2018

T cells

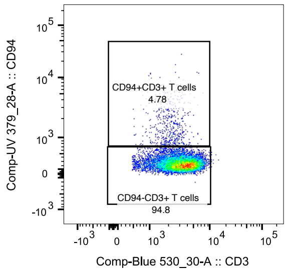

[20180724 Batch1_564_017.fcs]
CD3+ T cells
10896

CD94- T cells

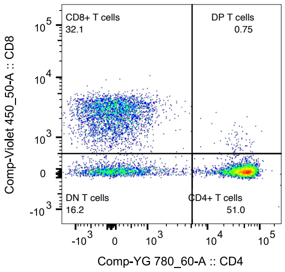

[20180724 Batch1_564_017.fcs]
CD94-CD3+ T cells
10333

CD4 T cells

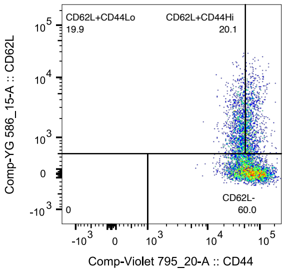

[20180724 Batch1_564_017.fcs]
CD4+ T cells
5270

CD4 T cells

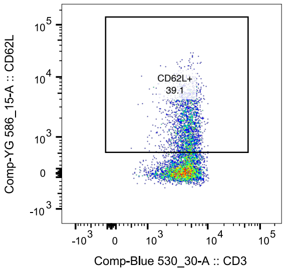

[20180724 Batch1_564_017.fcs]
CD4+ T cells
5270

CD8 T cells

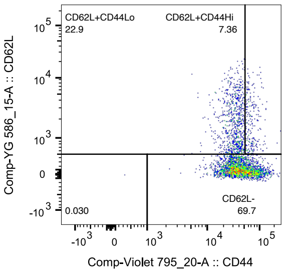

[20180724 Batch1_564_017.fcs]
CD8+ T cells
3314

CD8 T cells

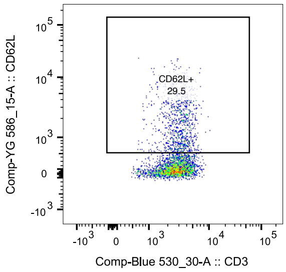

[20180724 Batch1_564_017.fcs]
CD8+ T cells
3314

DN T cells

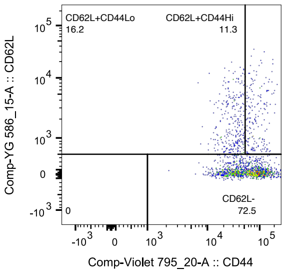

[20180724 Batch1_564_017.fcs]
DN T cells
1671

DN T cells

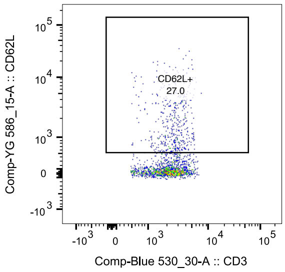

[20180724 Batch1_564_017.fcs]
DN T cells
1671

DP T cells

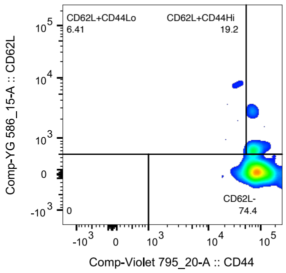

[20180724 Batch1_564_017.fcs]
DP T cells
78.0

DP T cells

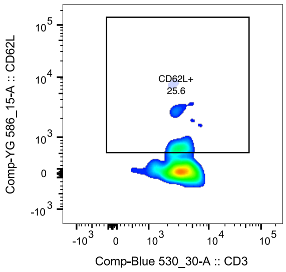

[20180724 Batch1_564_017.fcs]
DP T cells
78.0

565
24-JUL-2018

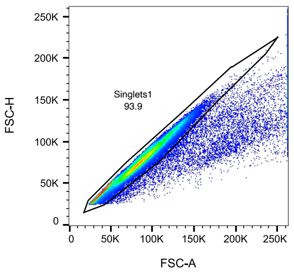

[20180724 Batch1_565_018.fcs]
Ungated
130844

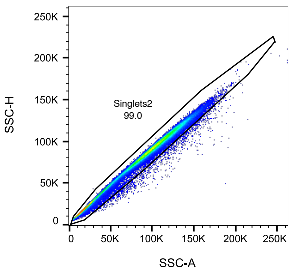

[20180724 Batch1_565_018.fcs]
Singlets1
122856

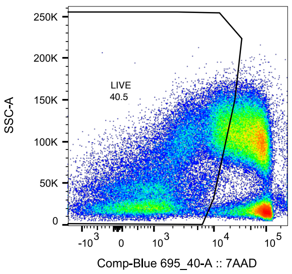

[20180724 Batch1_565_018.fcs]
Singlets2
121678

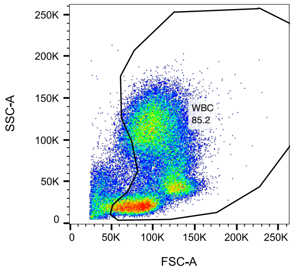

[20180724 Batch1_565_018.fcs]
LIVE
49223

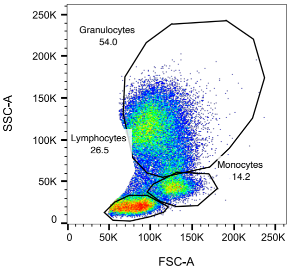

[20180724 Batch1_565_018.fcs]
WBC
41960

Granulocytes

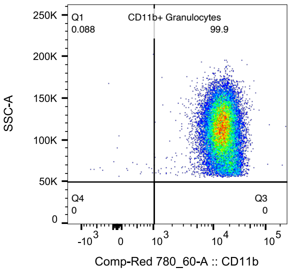

[20180724 Batch1_565_018.fcs]
Granulocytes
22644

Monocytes

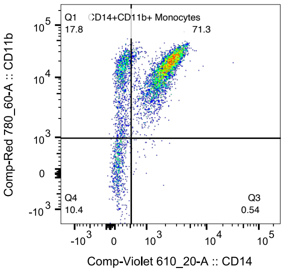

[20180724 Batch1_565_018.fcs]
Monocytes
5947

Lymphocytes

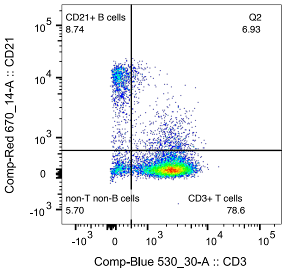

[20180724 Batch1_565_018.fcs]
Lymphocytes
11132

non-T non-B

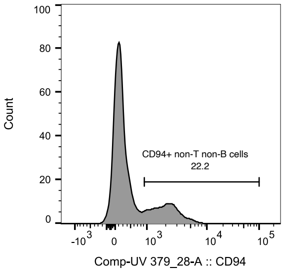

[20180724 Batch1_565_018.fcs]
non-T non-B cells
634

565
24-JUL-2018

T cells

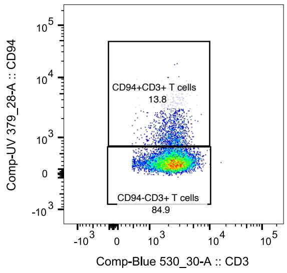

[20180724 Batch1_565_018.fcs]
CD3+ T cells
8754

CD94- T cells

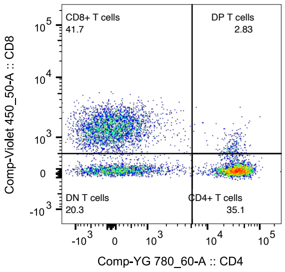

[20180724 Batch1_565_018.fcs]
CD94-CD3+ T cells
7431

CD4 T cells

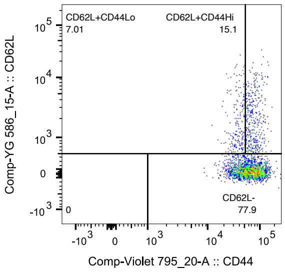

[20180724 Batch1_565_018.fcs]
CD4+ T cells
2610

CD4 T cells

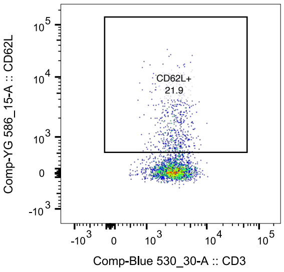

[20180724 Batch1_565_018.fcs]
CD4+ T cells
2610

CD8 T cells

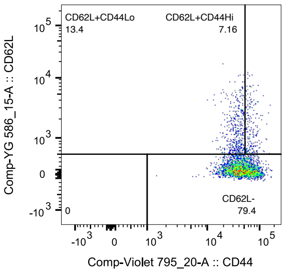

[20180724 Batch1_565_018.fcs]
CD8+ T cells
3099

CD8 T cells

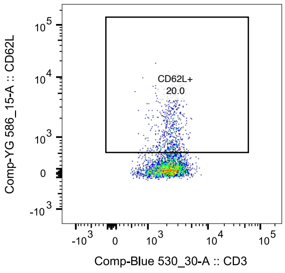

[20180724 Batch1_565_018.fcs]
CD8+ T cells
3099

DN T cells

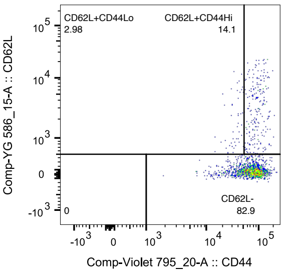

[20180724 Batch1_565_018.fcs]
DN T cells
1512

DN T cells

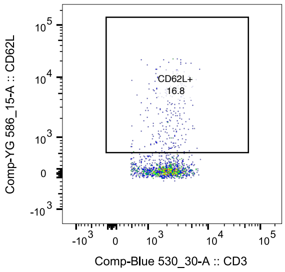

[20180724 Batch1_565_018.fcs]
DN T cells
1512

DP T cells

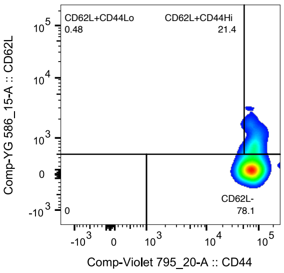

[20180724 Batch1_565_018.fcs]
DP T cells
210

DP T cells

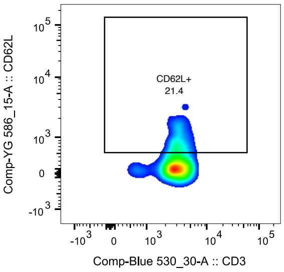

[20180724 Batch1_565_018.fcs]
DP T cells
210

583
24-JUL-2018

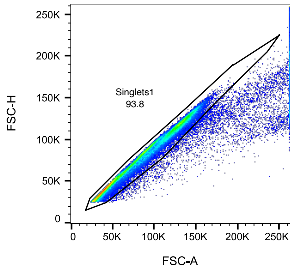

[20180724 Batch1_583_019.fcs]
Ungated
100845

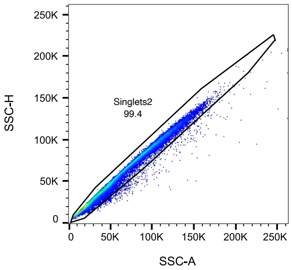

[20180724 Batch1_583_019.fcs]
Singlets1
94571

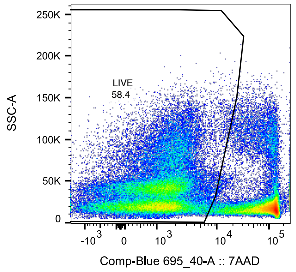

[20180724 Batch1_583_019.fcs]
Singlets2
94022

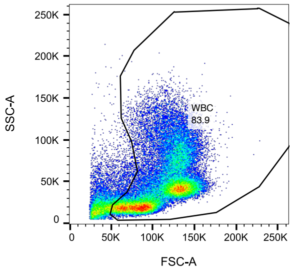

[20180724 Batch1_583_019.fcs]
LIVE
54942

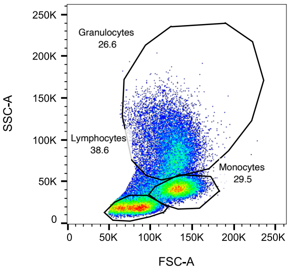

[20180724 Batch1_583_019.fcs]
WBC
46119

Granulocytes

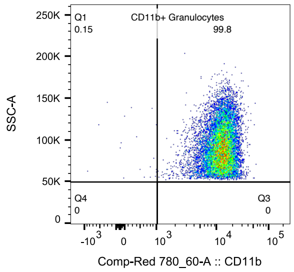

[20180724 Batch1_583_019.fcs]
Granulocytes
12273

Monocytes

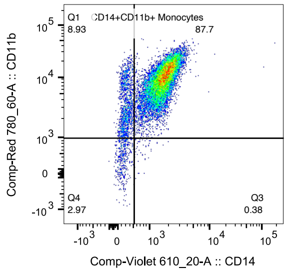

[20180724 Batch1_583_019.fcs]
Monocytes
13594

Lymphocytes

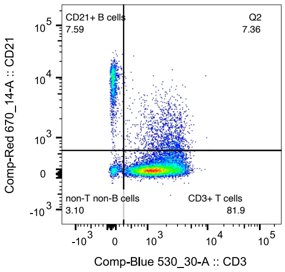

[20180724 Batch1_583_019.fcs]
Lymphocytes
17817

non-T non-B

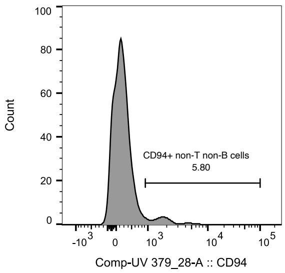

[20180724 Batch1_583_019.fcs]
non-T non-B cells
552

583
24-JUL-2018

T cells

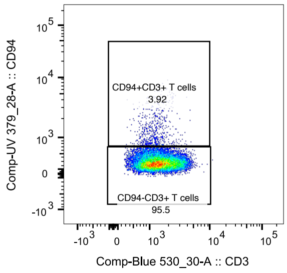

[20180724 Batch1_583_019.fcs]
CD3+ T cells
14601

CD94- T cells

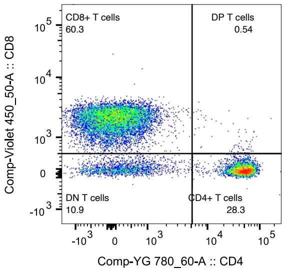

[20180724 Batch1_583_019.fcs]
CD94-CD3+ T cells
13950

CD4 T cells

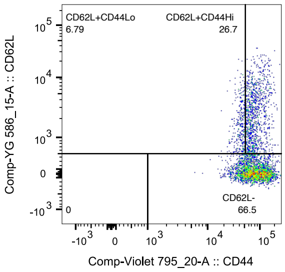

[20180724 Batch1_583_019.fcs]
CD4+ T cells
3949

CD4 T cells

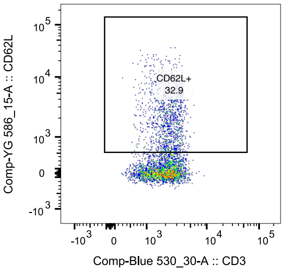

[20180724 Batch1_583_019.fcs]
CD4+ T cells
3949

CD8 T cells

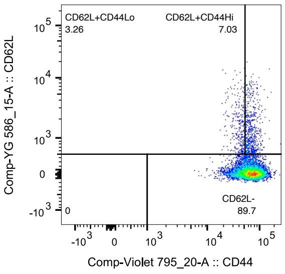

[20180724 Batch1_583_019.fcs]
CD8+ T cells
8411

CD8 T cells

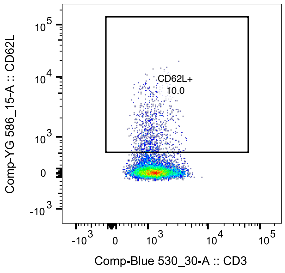

[20180724 Batch1_583_019.fcs]
CD8+ T cells
8411

DN T cells

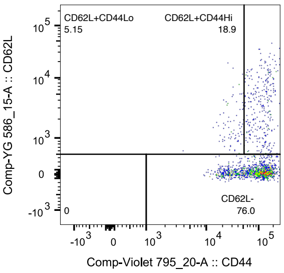

[20180724 Batch1_583_019.fcs]
DN T cells
1514

DN T cells

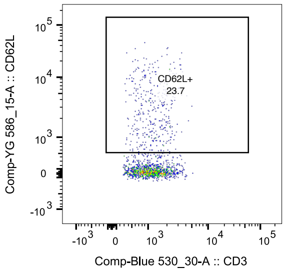

[20180724 Batch1_583_019.fcs]
DN T cells
1514

DP T cells

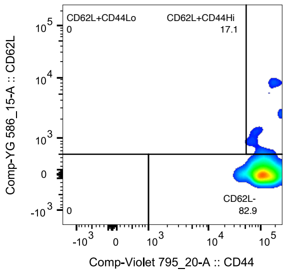

[20180724 Batch1_583_019.fcs]
DP T cells
76.0

DP T cells

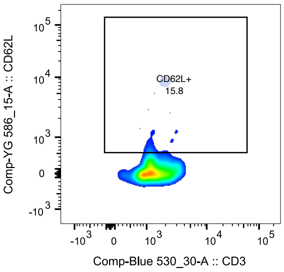

[20180724 Batch1_583_019.fcs]
DP T cells
76.0

587
24-JUL-2018

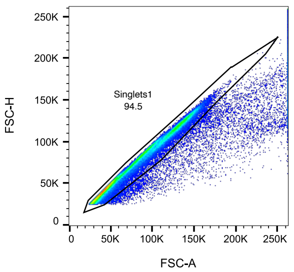

[20180724 Batch1_587_020.fcs]
Ungated
122507

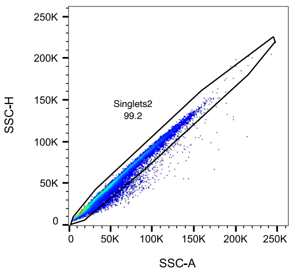

[20180724 Batch1_587_020.fcs]
Singlets1
115780

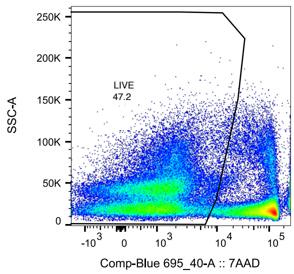

[20180724 Batch1_587_020.fcs]
Singlets2
114876

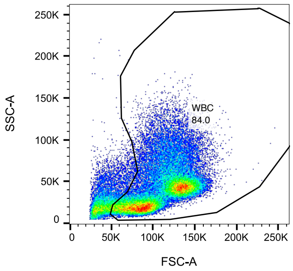

[20180724 Batch1_587_020.fcs]
LIVE
54168

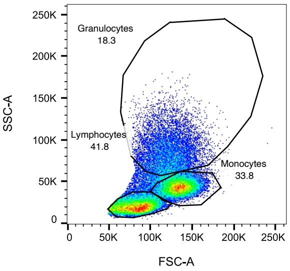

[20180724 Batch1_587_020.fcs]
WBC
45481

Granulocytes

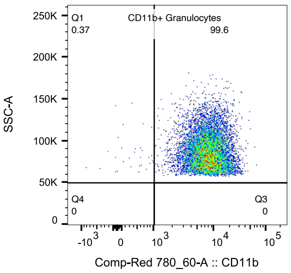

[20180724 Batch1_587_020.fcs]
Granulocytes
8326

Monocytes

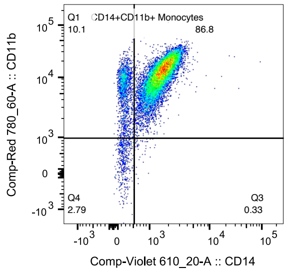

[20180724 Batch1_587_020.fcs]
Monocytes
15363

Lymphocytes

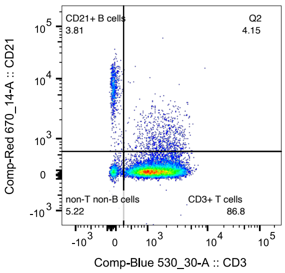

[20180724 Batch1_587_020.fcs]
Lymphocytes
19026

non-T non-B

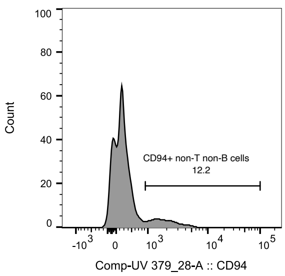

[20180724 Batch1_587_020.fcs]
non-T non-B cells
994

587
24-JUL-2018

T cells

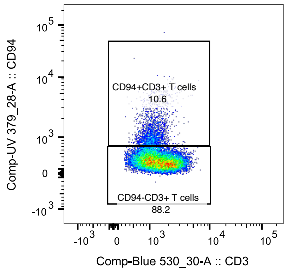

[20180724 Batch1_587_020.fcs]
CD3+ T cells
16519

CD94- T cells

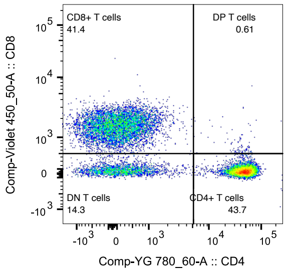

[20180724 Batch1_587_020.fcs]
CD94-CD3+ T cells
14574

CD4 T cells

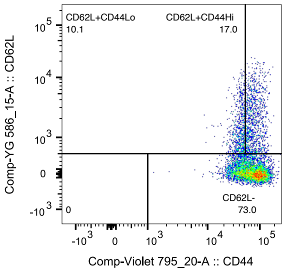

[20180724 Batch1_587_020.fcs]
CD4+ T cells
6375

CD4 T cells

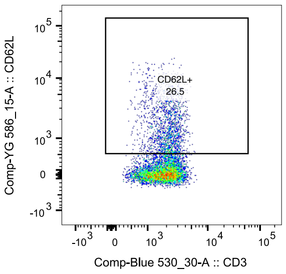

[20180724 Batch1_587_020.fcs]
CD4+ T cells
6375

CD8 T cells

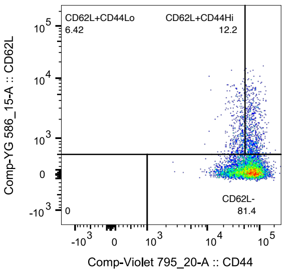

[20180724 Batch1_587_020.fcs]
CD8+ T cells
6027

CD8 T cells

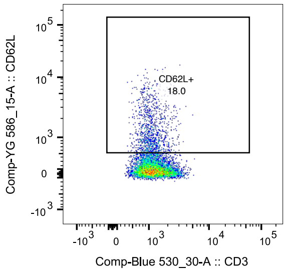

[20180724 Batch1_587_020.fcs]
CD8+ T cells
6027

DN T cells

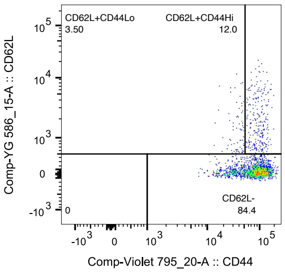

[20180724 Batch1_587_020.fcs]
DN T cells
2083

DN T cells

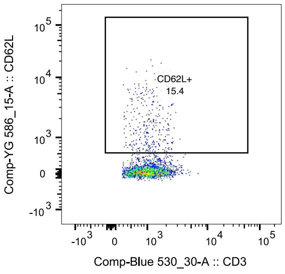

[20180724 Batch1_587_020.fcs]
DN T cells
2083

DP T cells

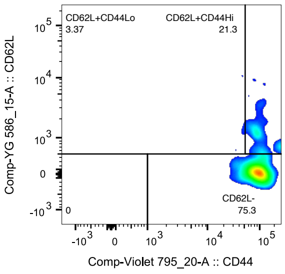

[20180724 Batch1_587_020.fcs]
DP T cells
89.0

DP T cells

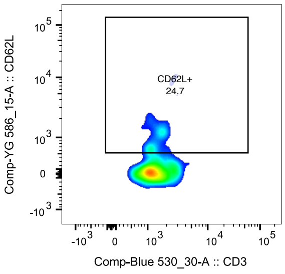

[20180724 Batch1_587_020.fcs]
DP T cells
89.0

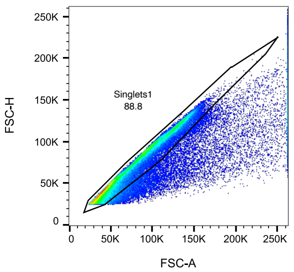

[20180724 Batch1_588_021.fcs]
Ungated
125892

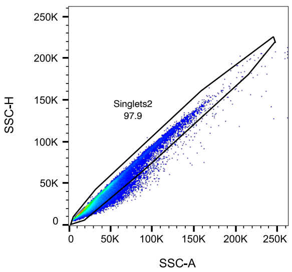

[20180724 Batch1_588_021.fcs]
Singlets1
111774

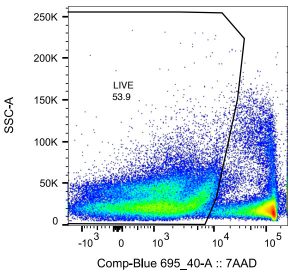

[20180724 Batch1_588_021.fcs]
Singlets2
109389

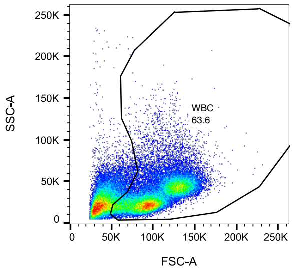

[20180724 Batch1_588_021.fcs]
LIVE
58994

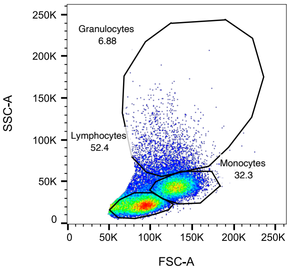

[20180724 Batch1_588_021.fcs]
WBC
37523

Granulocytes

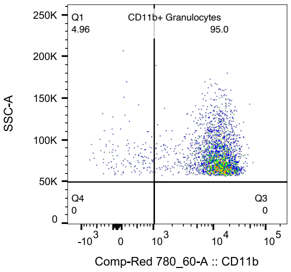

[20180724 Batch1_588_021.fcs]
Granulocytes
2580

Monocytes

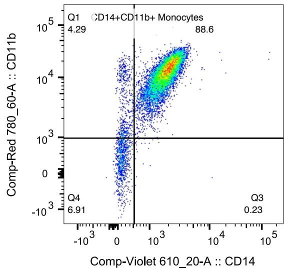

[20180724 Batch1_588_021.fcs]
Monocytes
12106

Lymphocytes

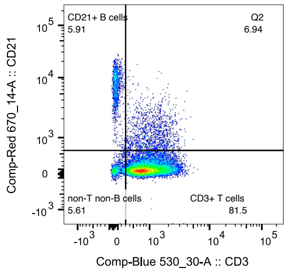

[20180724 Batch1_588_021.fcs]
Lymphocytes
19675

non-T non-B

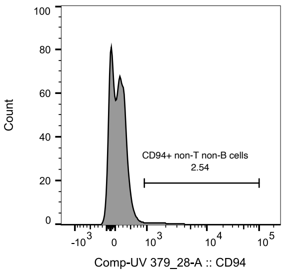

[20180724 Batch1_588_021.fcs]
non-T non-B cells
1104

588
24-JUL-2018

T cells

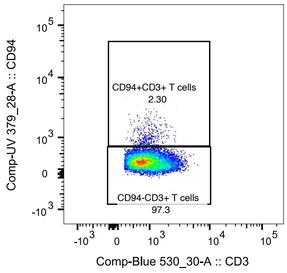

[20180724 Batch1_588_021.fcs]
CD3+ T cells
16042

CD94- T cells

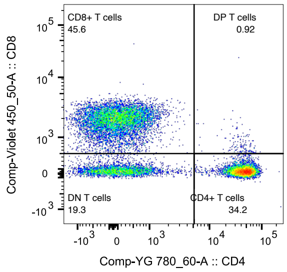

[20180724 Batch1_588_021.fcs]
CD94-CD3+ T cells
15613

CD4 T cells

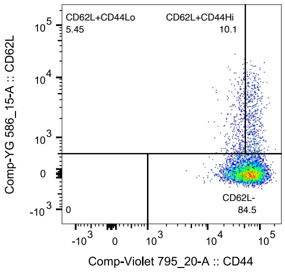

[20180724 Batch1_588_021.fcs]
CD4+ T cells
5337

CD4 T cells

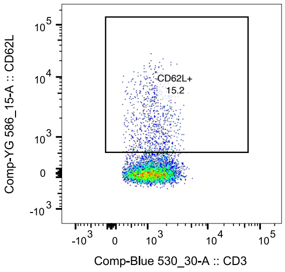

[20180724 Batch1_588_021.fcs]
CD4+ T cells
5337

CD8 T cells

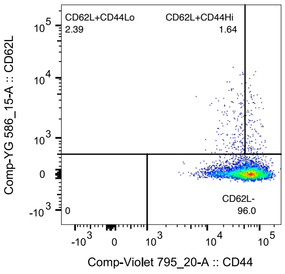

[20180724 Batch1_588_021.fcs]
CD8+ T cells
7118

CD8 T cells

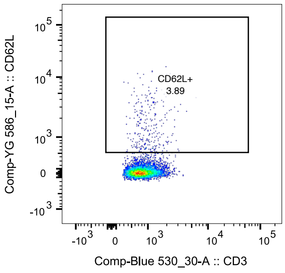

[20180724 Batch1_588_021.fcs]
CD8+ T cells
7118

DN T cells

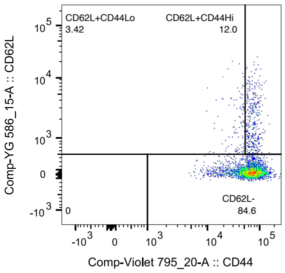

[20180724 Batch1_588_021.fcs]
DN T cells
3014

DN T cells

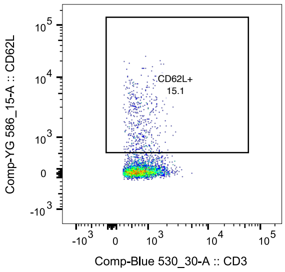

[20180724 Batch1_588_021.fcs]
DN T cells
3014

DP T cells

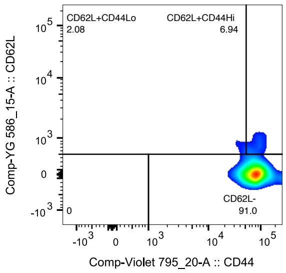

[20180724 Batch1_588_021.fcs]
DP T cells
144

DP T cells

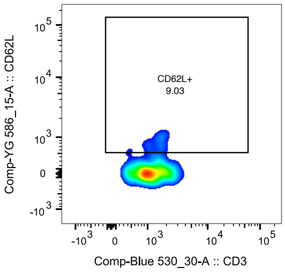

[20180724 Batch1_588_021.fcs]
DP T cells
144

589
31-JUL-2018

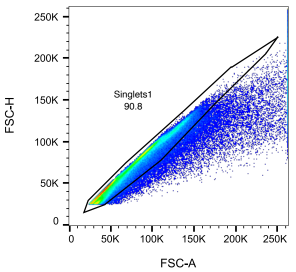

[20180731 Batch2_589_019.fcs]
Ungated
184935

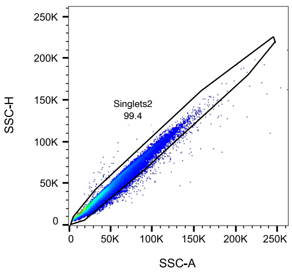

[20180731 Batch2_589_019.fcs]
Singlets1
167973

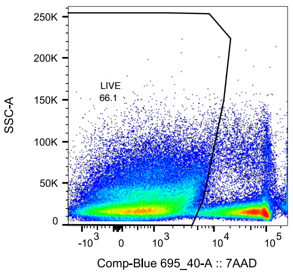

[20180731 Batch2_589_019.fcs]
Singlets2
166981

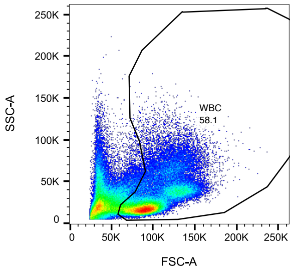

[20180731 Batch2_589_019.fcs]
LIVE
110425

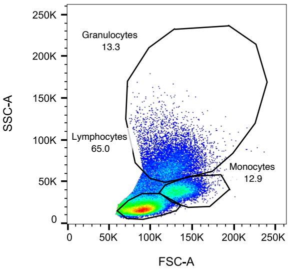

[20180731 Batch2_589_019.fcs]
WBC
64163

Granulocytes

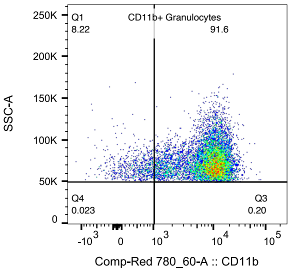

[20180731 Batch2_589_019.fcs]
Granulocytes
8519

Monocytes

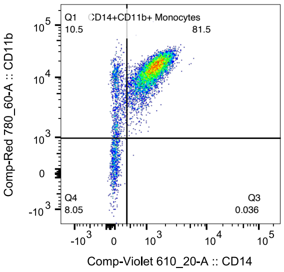

[20180731 Batch2_589_019.fcs]
Monocytes
8261

Lymphocytes

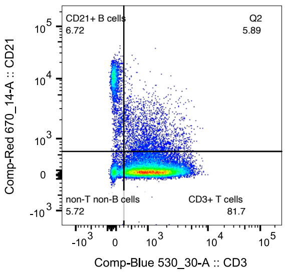

[20180731 Batch2_589_019.fcs]
Lymphocytes
41679

non-T non-B

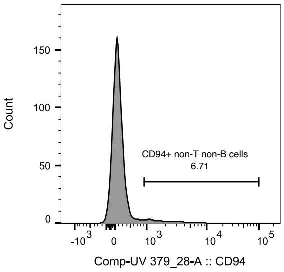

[20180731 Batch2_589_019.fcs]
non-T non-B cells
2383

589
31-JUL-2018

T cells

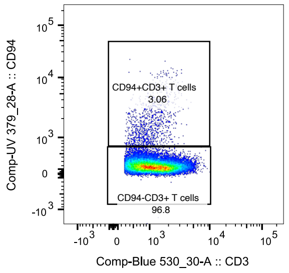

[20180731 Batch2_589_019.fcs]
CD3+ T cells
34041

CD94- T cells

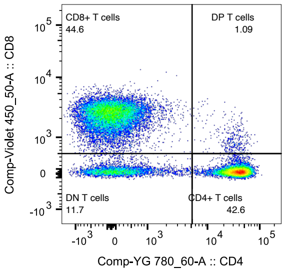

[20180731 Batch2_589_019.fcs]
CD94-CD3+ T cells
32948

CD4 T cells

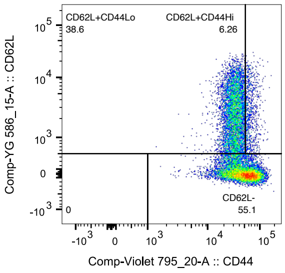

[20180731 Batch2_589_019.fcs]
CD4+ T cells
14027

CD4 T cells

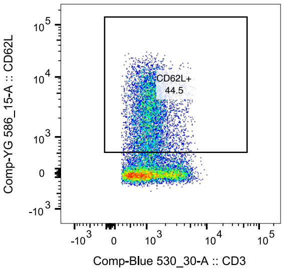

[20180731 Batch2_589_019.fcs]
CD4+ T cells
14027

CD8 T cells

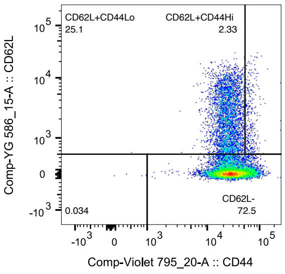

[20180731 Batch2_589_019.fcs]
CD8+ T cells
14700

CD8 T cells

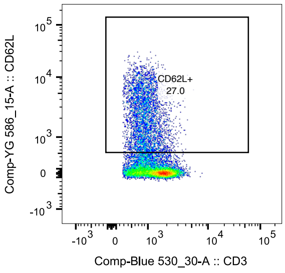

[20180731 Batch2_589_019.fcs]
CD8+ T cells
14700

DN T cells

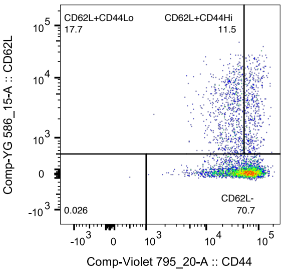

[20180731 Batch2_589_019.fcs]
DN T cells
3862

DN T cells

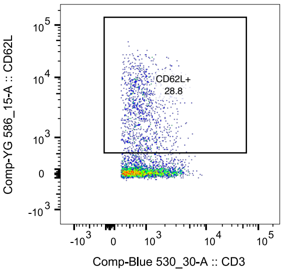

[20180731 Batch2_589_019.fcs]
DN T cells
3862

DP T cells

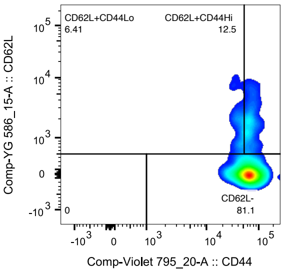

[20180731 Batch2_589_019.fcs]
DP T cells
359

DP T cells

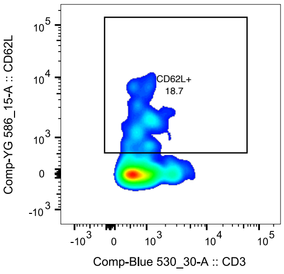

[20180731 Batch2_589_019.fcs]
DP T cells
359

551
31-JUL-2018

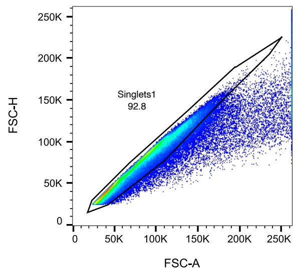

[20180731 Batch2_551_013.fcs]
Ungated
195492

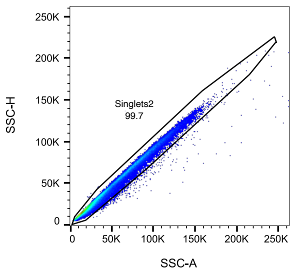

[20180731 Batch2_551_013.fcs]
Singlets1
181333

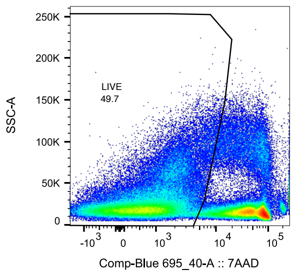

[20180731 Batch2_551_013.fcs]
Singlets2
180847

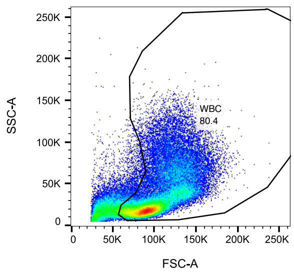

[20180731 Batch2_551_013.fcs]
LIVE
89914

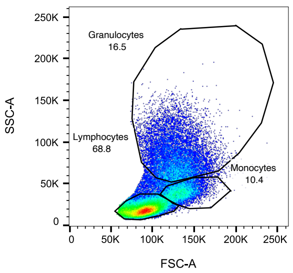

[20180731 Batch2_551_013.fcs]
WBC
72279

Granulocytes

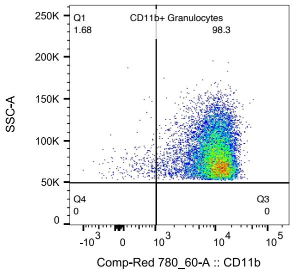

[20180731 Batch2_551_013.fcs]
Granulocytes
11911

Monocytes

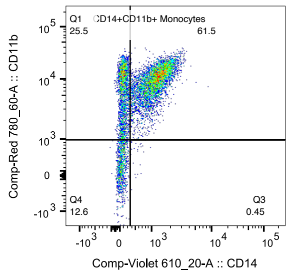

[20180731 Batch2_551_013.fcs]
Monocytes
7551

Lymphocytes

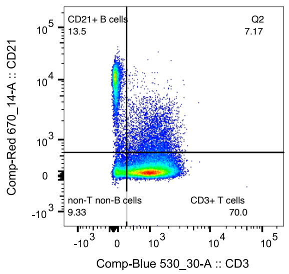

[20180731 Batch2_551_013.fcs]
Lymphocytes
49704

non-T non-B

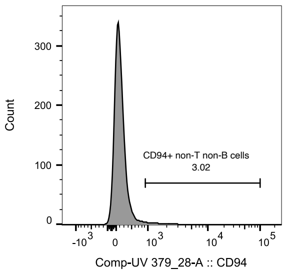

[20180731 Batch2_551_013.fcs]
non-T non-B cells
4638

551
31-JUL-2018

T cells

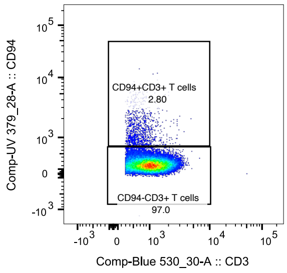

[20180731 Batch2_551_013.fcs]
CD3+ T cells
34801

CD94- T cells

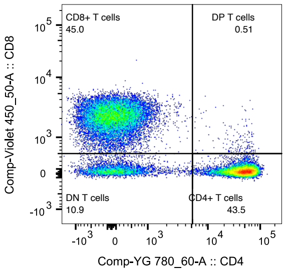

[20180731 Batch2_551_013.fcs]
CD94-CD3+ T cells
33745

CD4 T cells

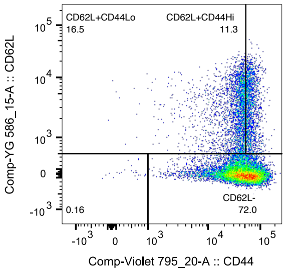

[20180731 Batch2_551_013.fcs]
CD4+ T cells
14682

CD4 T cells

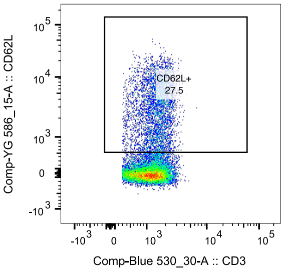

[20180731 Batch2_551_013.fcs]
CD4+ T cells
14682

CD8 T cells

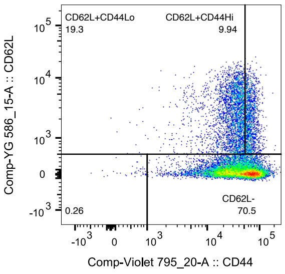

[20180731 Batch2_551_013.fcs]
CD8+ T cells
15197

CD8 T cells

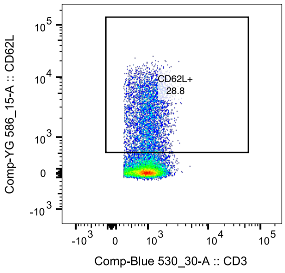

[20180731 Batch2_551_013.fcs]
CD8+ T cells
15197

DN T cells

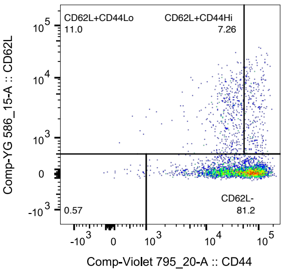

[20180731 Batch2_551_013.fcs]
DN T cells
3694

DN T cells

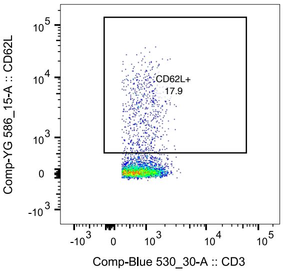

[20180731 Batch2_551_013.fcs]
DN T cells
3694

DP T cells

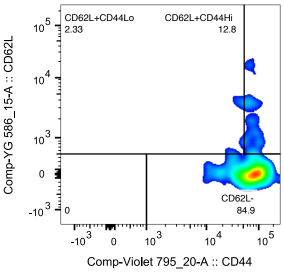

[20180731 Batch2_551_013.fcs]
DP T cells
172

DP T cells

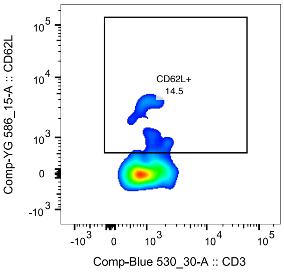

[20180731 Batch2_551_013.fcs]
DP T cells
172

554
31-JUL-2018

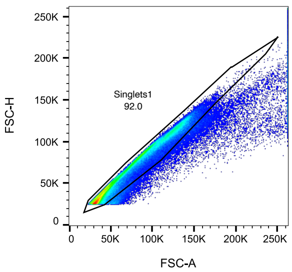

[20180731 Batch2_554_014.fcs]
Ungated
227354

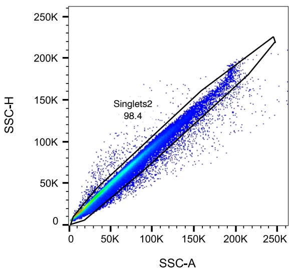

[20180731 Batch2_554_014.fcs]
Singlets1
209168

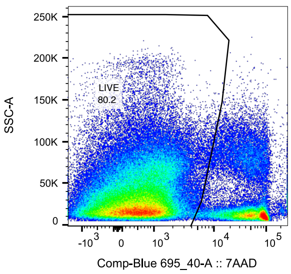

[20180731 Batch2_554_014.fcs]
Singlets2
205887

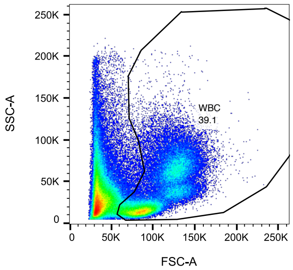

[20180731 Batch2_554_014.fcs]
LIVE
165093

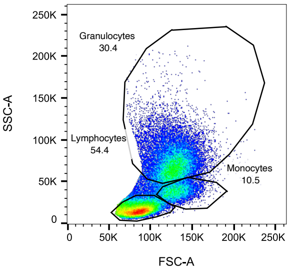

[20180731 Batch2_554_014.fcs]
WBC
64545

Granulocytes

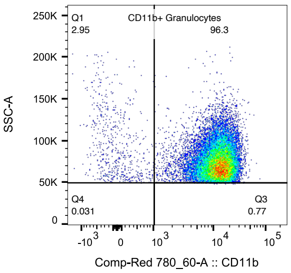

[20180731 Batch2_554_014.fcs]
Granulocytes
19591

Monocytes

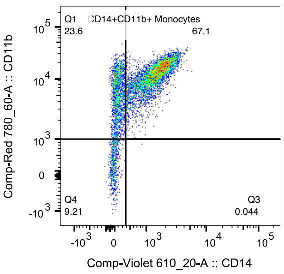

[20180731 Batch2_554_014.fcs]
Monocytes
6783

Lymphocytes

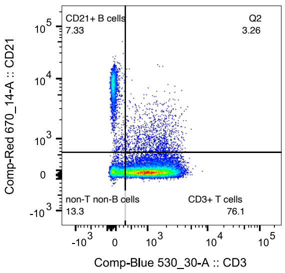

[20180731 Batch2_554_014.fcs]
Lymphocytes
35087

non-T non-B

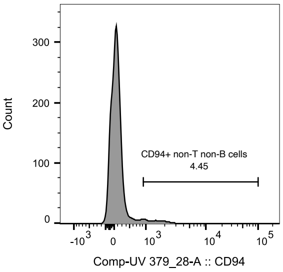

[20180731 Batch2_554_014.fcs]
non-T non-B cells
4676

554
31-JUL-2018

T cells

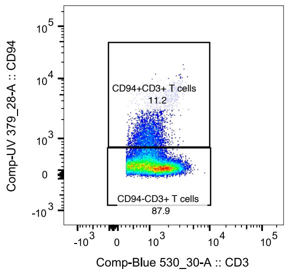

[20180731 Batch2_554_014.fcs]
CD3+ T cells
26694

CD94- T cells

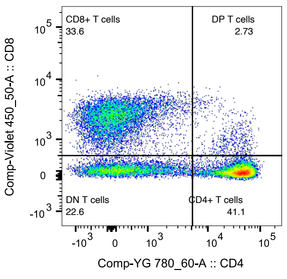

[20180731 Batch2_554_014.fcs]
CD94-CD3+ T cells
23470

CD4 T cells

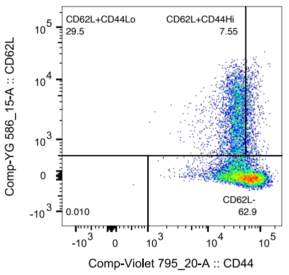

[20180731 Batch2_554_014.fcs]
CD4+ T cells
9657

CD4 T cells

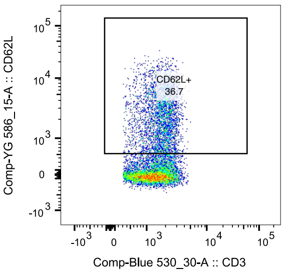

[20180731 Batch2_554_014.fcs]
CD4+ T cells
9657

CD8 T cells

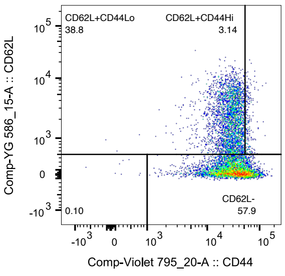

[20180731 Batch2_554_014.fcs]
CD8+ T cells
7877

CD8 T cells

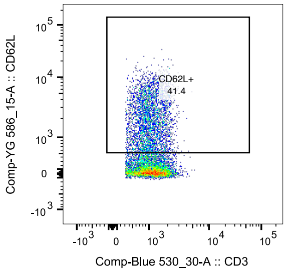

[20180731 Batch2_554_014.fcs]
CD8+ T cells
7877

DN T cells

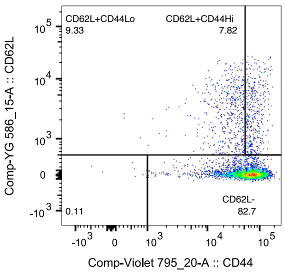

[20180731 Batch2_554_014.fcs]
DN T cells
5296

DN T cells

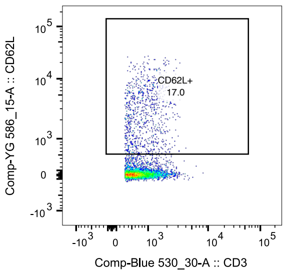

[20180731 Batch2_554_014.fcs]
DN T cells
5296

DP T cells

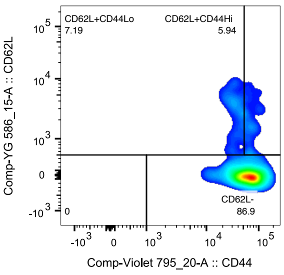

[20180731 Batch2_554_014.fcs]
DP T cells
640

DP T cells

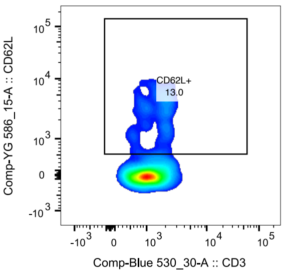

[20180731 Batch2_554_014.fcs]
DP T cells
640

582
31-JUL-2018

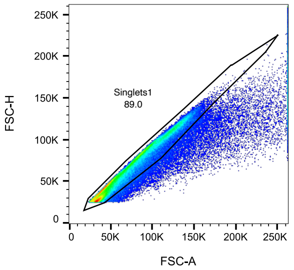

[20180731 Batch2_582_018.fcs]
Ungated
137820

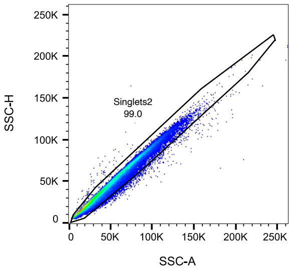

[20180731 Batch2_582_018.fcs]
Singlets1
122667

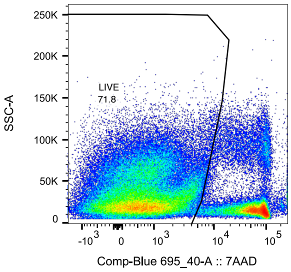

[20180731 Batch2_582_018.fcs]
Singlets2
121480

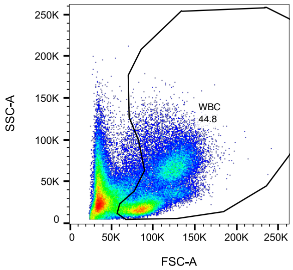

[20180731 Batch2_582_018.fcs]
LIVE
87195

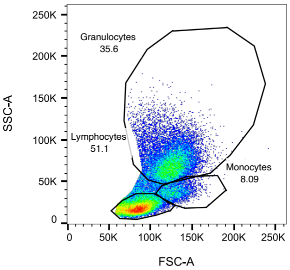

[20180731 Batch2_582_018.fcs]
WBC
39093

Granulocytes

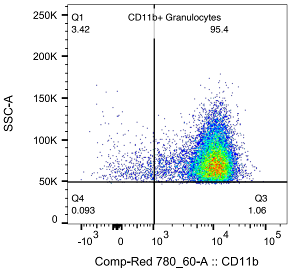

[20180731 Batch2_582_018.fcs]
Granulocytes
13924

Monocytes

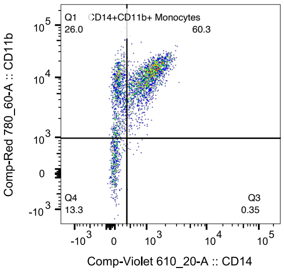

[20180731 Batch2_582_018.fcs]
Monocytes
3164

Lymphocytes

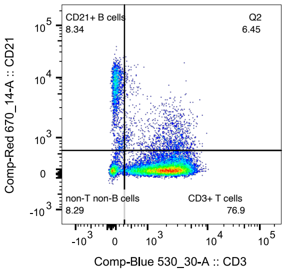

[20180731 Batch2_582_018.fcs]
Lymphocytes
19992

non-T non-B

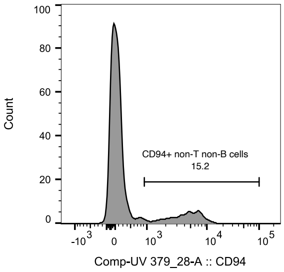

[20180731 Batch2_582_018.fcs]
non-T non-B cells
1658

582
31-JUL-2018

T cells

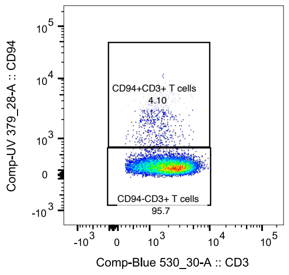

[20180731 Batch2_582_018.fcs]
CD3+ T cells
15377

CD94- T cells

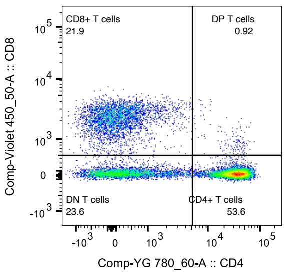

[20180731 Batch2_582_018.fcs]
CD94-CD3+ T cells
14719

CD4 T cells

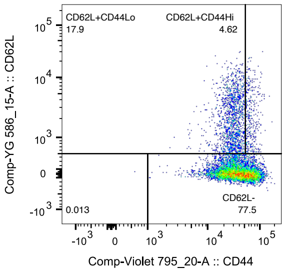

[20180731 Batch2_582_018.fcs]
CD4+ T cells
7883

CD4 T cells

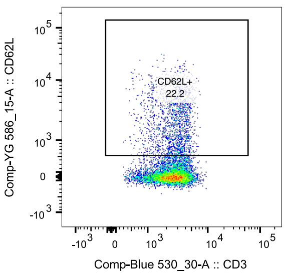

[20180731 Batch2_582_018.fcs]
CD4+ T cells
7883

CD8 T cells

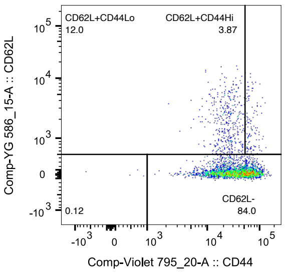

[20180731 Batch2_582_018.fcs]
CD8+ T cells
3228

CD8 T cells

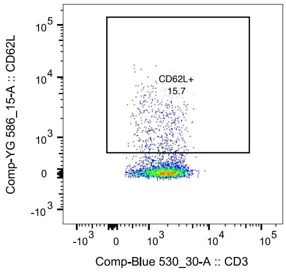

[20180731 Batch2_582_018.fcs]
CD8+ T cells
3228

DN T cells

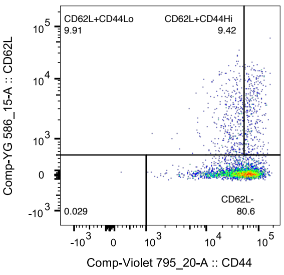

[20180731 Batch2_582_018.fcs]
DN T cells
3472

DN T cells

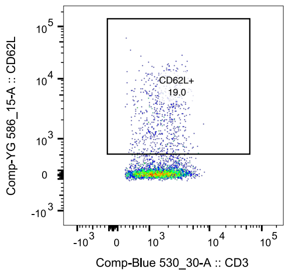

[20180731 Batch2_582_018.fcs]
DN T cells
3472

DP T cells

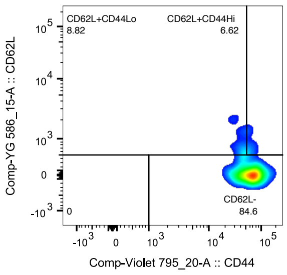

[20180731 Batch2_582_018.fcs]
DP T cells
136

DP T cells

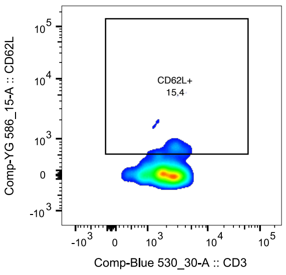

[20180731 Batch2_582_018.fcs]
DP T cells
136

529
31-JUL-2018

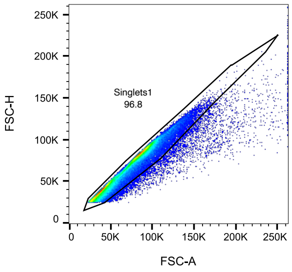

[20180731 Batch2_529_009.fcs]
Ungated
107927

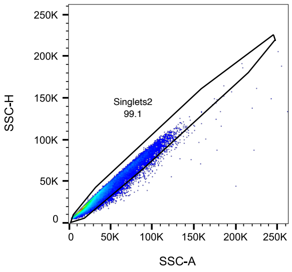

[20180731 Batch2_529_009.fcs]
Singlets1
104464

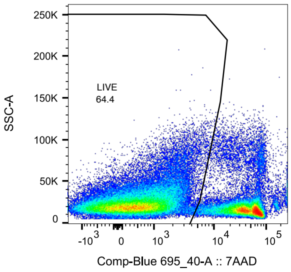

[20180731 Batch2_529_009.fcs]
Singlets2
103523

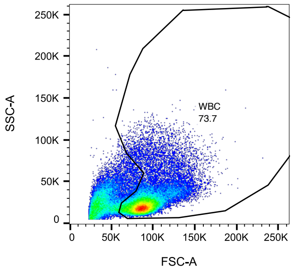

[20180731 Batch2_529_009.fcs]
LIVE
66706

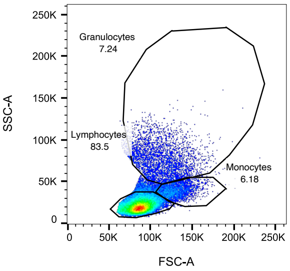

[20180731 Batch2_529_009.fcs]
WBC
49141

Granulocytes

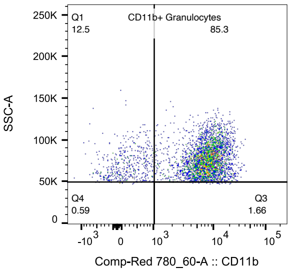

[20180731 Batch2_529_009.fcs]
Granulocytes
3560

Monocytes

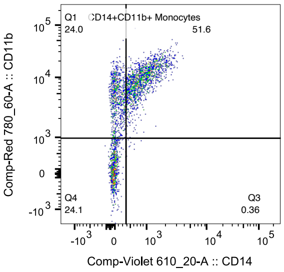

[20180731 Batch2_529_009.fcs]
Monocytes
3035

Lymphocytes

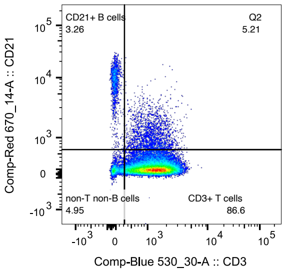

[20180731 Batch2_529_009.fcs]
Lymphocytes
41044

non-T non-B

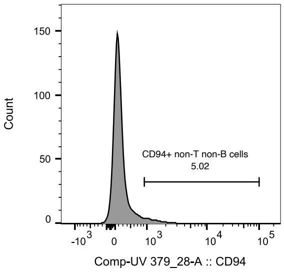

[20180731 Batch2_529_009.fcs]
non-T non-B cells
2031

529
31-JUL-2018

T cells

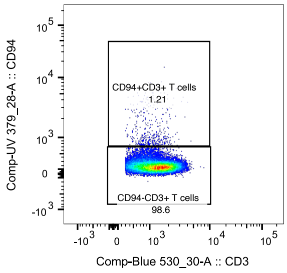

[20180731 Batch2_529_009.fcs]
CD3+ T cells
35535

CD94- T cells

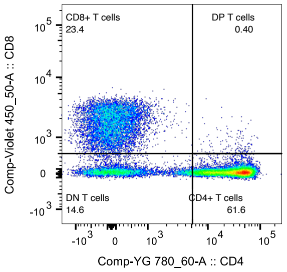

[20180731 Batch2_529_009.fcs]
CD94-CD3+ T cells
35027

CD4 T cells

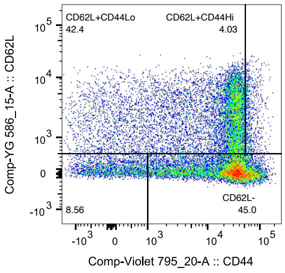

[20180731 Batch2_529_009.fcs]
CD4+ T cells
21582

CD4 T cells

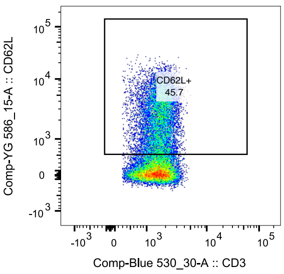

[20180731 Batch2_529_009.fcs]
CD4+ T cells
21582

CD8 T cells

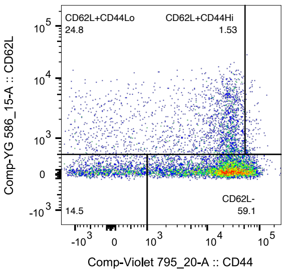

[20180731 Batch2_529_009.fcs]
CD8+ T cells
8186

CD8 T cells

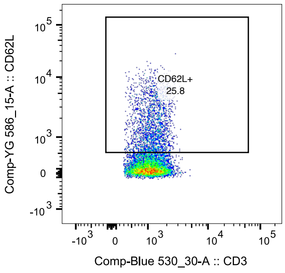

[20180731 Batch2_529_009.fcs]
CD8+ T cells
8186

DN T cells

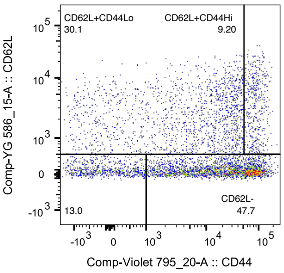

[20180731 Batch2_529_009.fcs]
DN T cells
5119

DN T cells

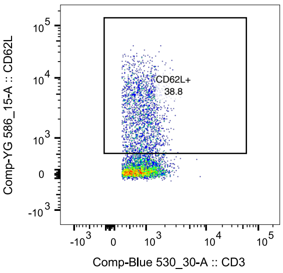

[20180731 Batch2_529_009.fcs]
DN T cells
5119

DP T cells

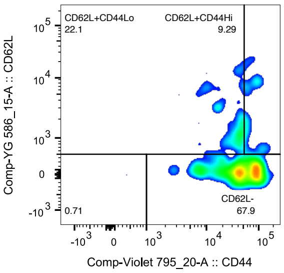

[20180731 Batch2_529_009.fcs]
DP T cells
140

DP T cells

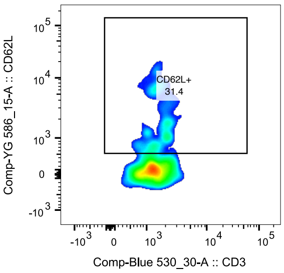

[20180731 Batch2_529_009.fcs]
DP T cells
140

516
31-JUL-2018

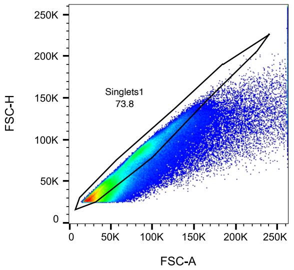

[20180731 Batch2_516_006.fcs]
Ungated
298042

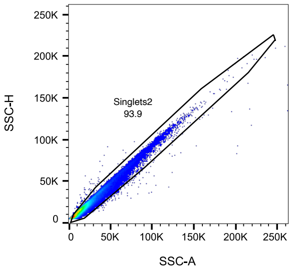

[20180731 Batch2_516_006.fcs]
Singlets1
219901

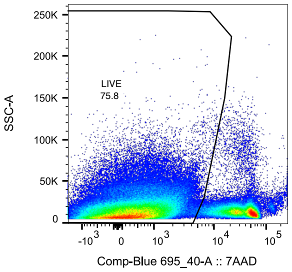

[20180731 Batch2_516_006.fcs]
Singlets2
206491

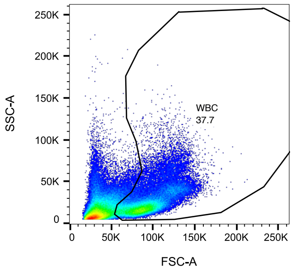

[20180731 Batch2_516_006.fcs]
LIVE
156575

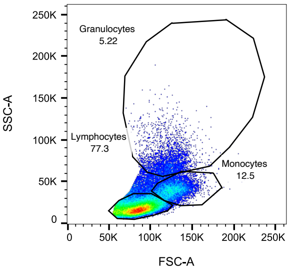

[20180731 Batch2_516_006.fcs]
WBC
58978

Granulocytes

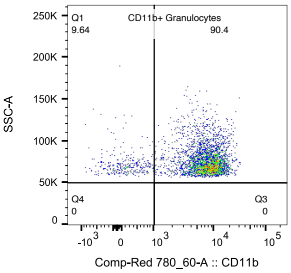

[20180731 Batch2_516_006.fcs]
Granulocytes
3081

Monocytes

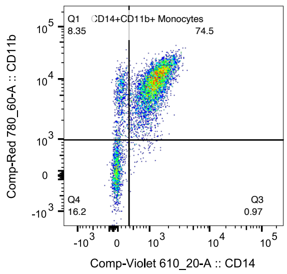

[20180731 Batch2_516_006.fcs]
Monocytes
7392

Lymphocytes

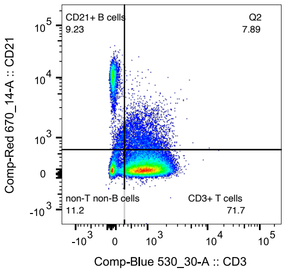

[20180731 Batch2_516_006.fcs]
Lymphocytes
45570

non-T non-B

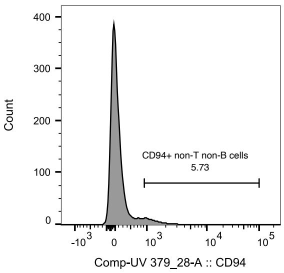

[20180731 Batch2_516_006.fcs]
non-T non-B cells
5114

516
31-JUL-2018

T cells

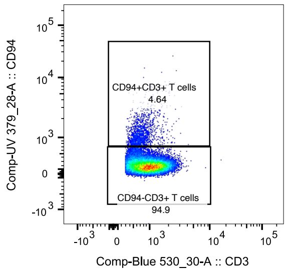

[20180731 Batch2_516_006.fcs]
CD3+ T cells
32654

CD94- T cells

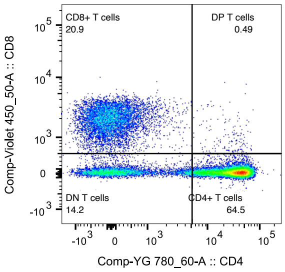

[20180731 Batch2_516_006.fcs]
CD94-CD3+ T cells
31002

CD4 T cells

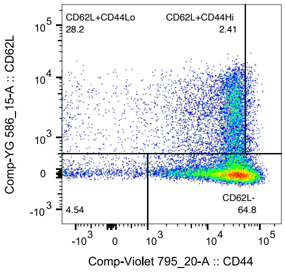

[20180731 Batch2_516_006.fcs]
CD4+ T cells
19985

CD4 T cells

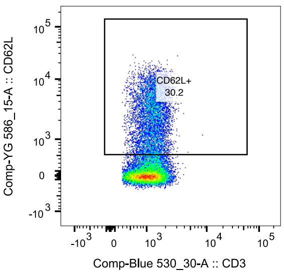

[20180731 Batch2_516_006.fcs]
CD4+ T cells
19985

CD8 T cells

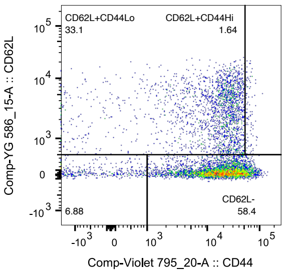

[20180731 Batch2_516_006.fcs]
CD8+ T cells
6465

CD8 T cells

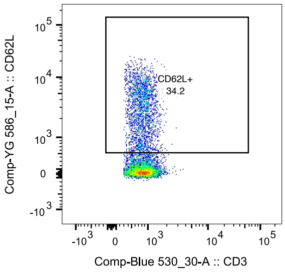

[20180731 Batch2_516_006.fcs]
CD8+ T cells
6465

DN T cells

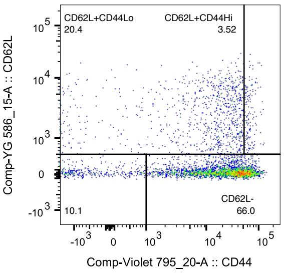

[20180731 Batch2_516_006.fcs]
DN T cells
4400

DN T cells

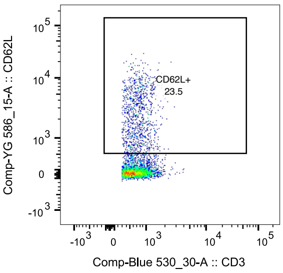

[20180731 Batch2_516_006.fcs]
DN T cells
4400

DP T cells

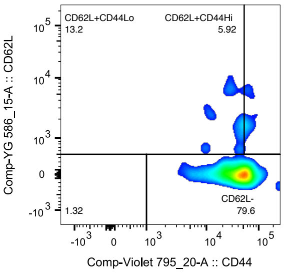

[20180731 Batch2_516_006.fcs]
DP T cells
152

DP T cells

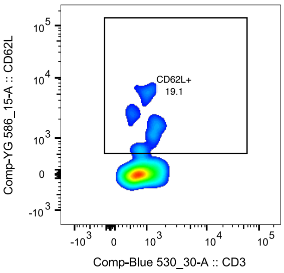

[20180731 Batch2_516_006.fcs]
DP T cells
152

507
31-JUL-2018

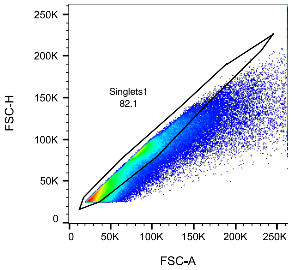

[20180731 Batch2_507_002.fcs]
Ungated
188396

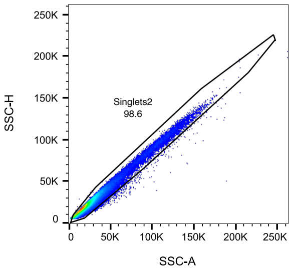

[20180731 Batch2_507_002.fcs]
Singlets1
154718

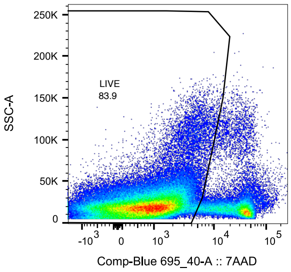

[20180731 Batch2_507_002.fcs]
Singlets2
152495

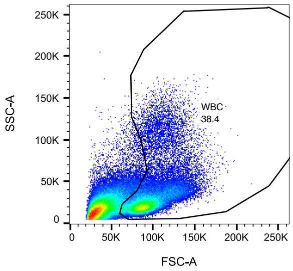

[20180731 Batch2_507_002.fcs]
LIVE
127984

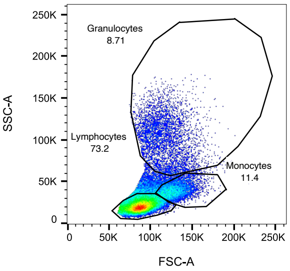

[20180731 Batch2_507_002.fcs]
WBC
49121

Granulocytes

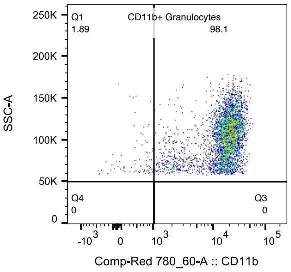

[20180731 Batch2_507_002.fcs]
Granulocytes
4280

Monocytes

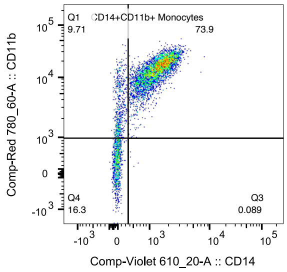

[20180731 Batch2_507_002.fcs]
Monocytes
5601

Lymphocytes

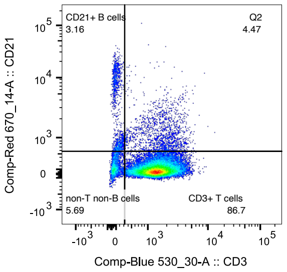

[20180731 Batch2_507_002.fcs]
Lymphocytes
35935

non-T non-B

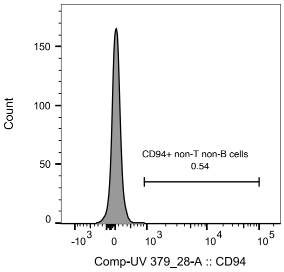

[20180731 Batch2_507_002.fcs]
non-T non-B cells
2044

507
31-JUL-2018

T cells

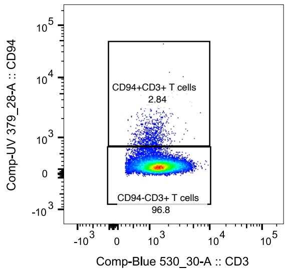

[20180731 Batch2_507_002.fcs]
CD3+ T cells
31150

CD94- T cells

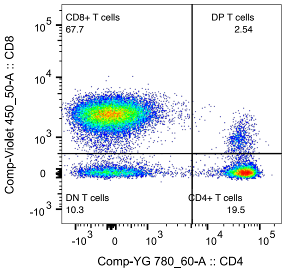

[20180731 Batch2_507_002.fcs]
CD94-CD3+ T cells
30168

CD4 T cells

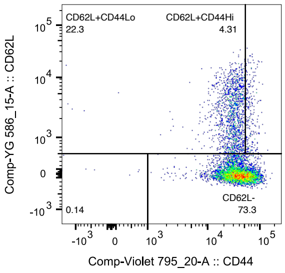

[20180731 Batch2_507_002.fcs]
CD4+ T cells
5874

CD4 T cells

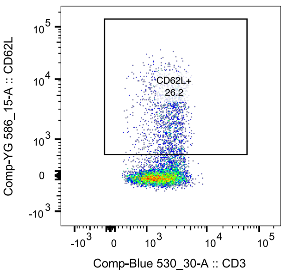

[20180731 Batch2_507_002.fcs]
CD4+ T cells
5874

CD8 T cells

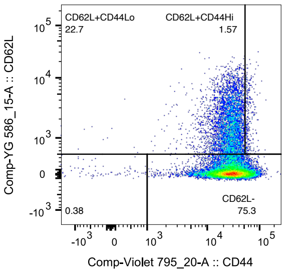

[20180731 Batch2_507_002.fcs]
CD8+ T cells
20422

CD8 T cells

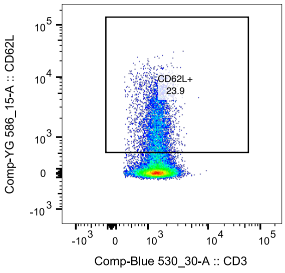

[20180731 Batch2_507_002.fcs]
CD8+ T cells
20422

DN T cells

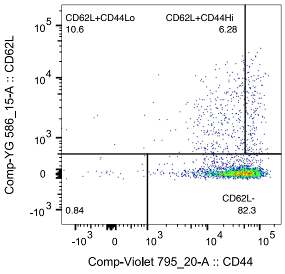

[20180731 Batch2_507_002.fcs]
DN T cells
3107

DN T cells

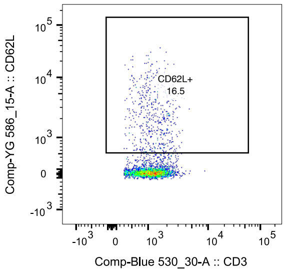

[20180731 Batch2_507_002.fcs]
DN T cells
3107

DP T cells

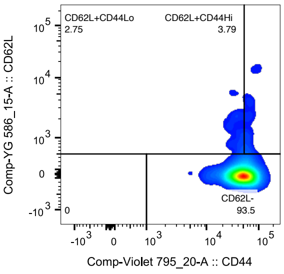

[20180731 Batch2_507_002.fcs]
DP T cells
765

DP T cells

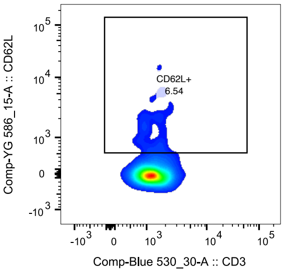

[20180731 Batch2_507_002.fcs]
DP T cells
765

530
31-JUL-2018

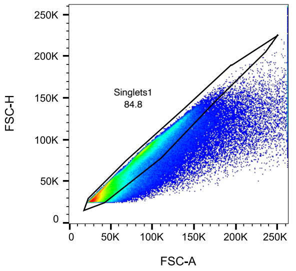

[20180731 Batch2_530_010.fcs]
Ungated
274781

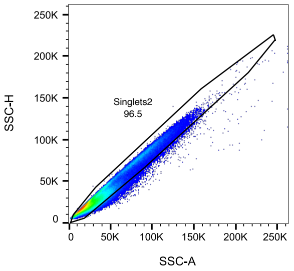

[20180731 Batch2_530_010.fcs]
Singlets1
232915

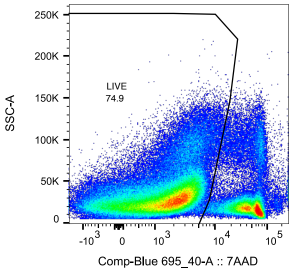

[20180731 Batch2_530_010.fcs]
Singlets2
224650

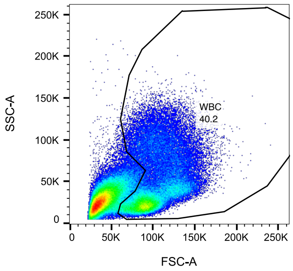

[20180731 Batch2_530_010.fcs]
LIVE
168328

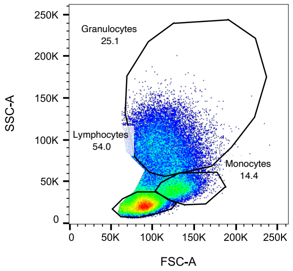

[20180731 Batch2_530_010.fcs]
WBC
67601

Granulocytes

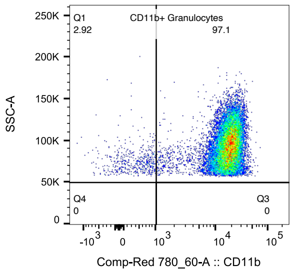

[20180731 Batch2_530_010.fcs]
Granulocytes
16970

Monocytes

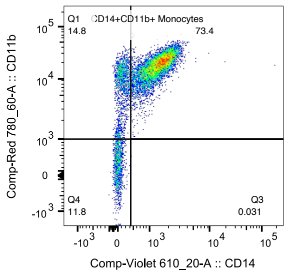

[20180731 Batch2_530_010.fcs]
Monocytes
9715

Lymphocytes

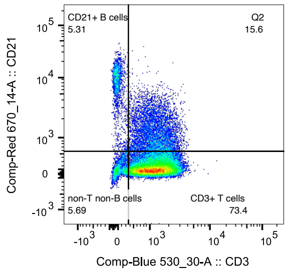

[20180731 Batch2_530_010.fcs]
Lymphocytes
36487

non-T non-B

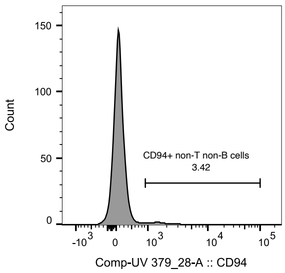

[20180731 Batch2_530_010.fcs]
non-T non-B cells
2077

530
31-JUL-2018

T cells

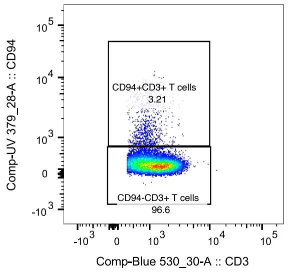

[20180731 Batch2_530_010.fcs]
CD3+ T cells
26777

CD94- T cells

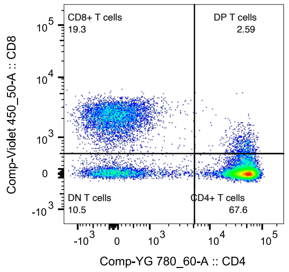

[20180731 Batch2_530_010.fcs]
CD94-CD3+ T cells
25861

CD4 T cells

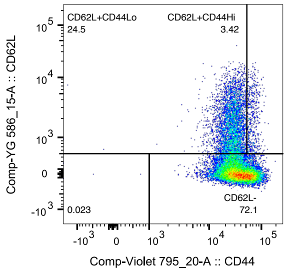

[20180731 Batch2_530_010.fcs]
CD4+ T cells
17490

CD4 T cells

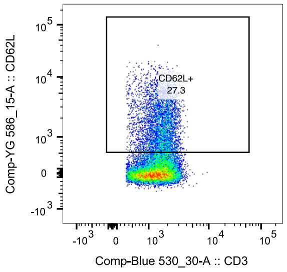

[20180731 Batch2_530_010.fcs]
CD4+ T cells
17490

CD8 T cells

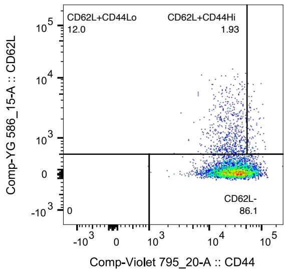

[20180731 Batch2_530_010.fcs]
CD8+ T cells
4981

CD8 T cells

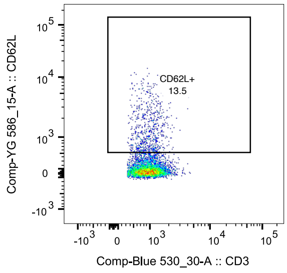

[20180731 Batch2_530_010.fcs]
CD8+ T cells
4981

DN T cells

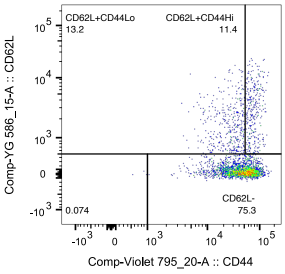

[20180731 Batch2_530_010.fcs]
DN T cells
2721

DN T cells

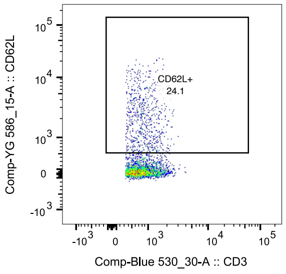

[20180731 Batch2_530_010.fcs]
DN T cells
2721

DP T cells

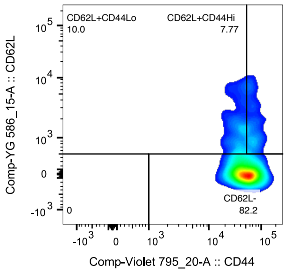

[20180731 Batch2_530_010.fcs]
DP T cells
669

DP T cells

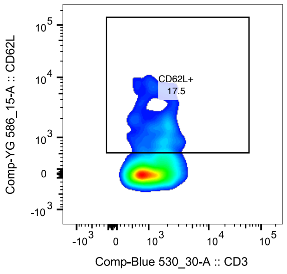

[20180731 Batch2_530_010.fcs]
DP T cells
669

525
31-JUL-2018

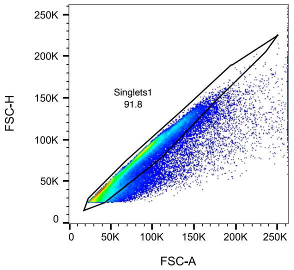

[20180731 Batch2_525_008.fcs]
Ungated
135024

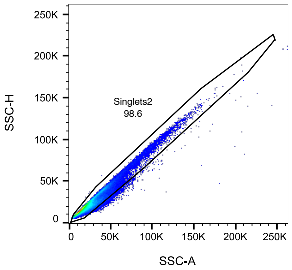

[20180731 Batch2_525_008.fcs]
Singlets1
123947

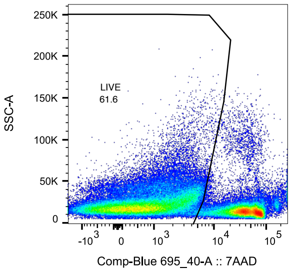

[20180731 Batch2_525_008.fcs]
Singlets2
122244

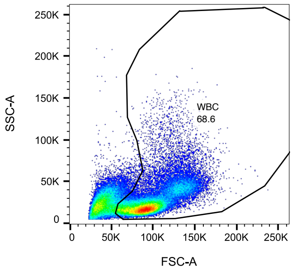

[20180731 Batch2_525_008.fcs]
LIVE
75352

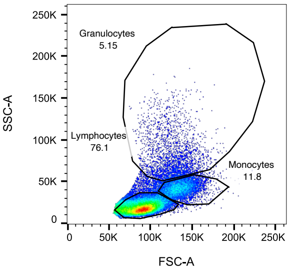

[20180731 Batch2_525_008.fcs]
WBC
51667

Granulocytes

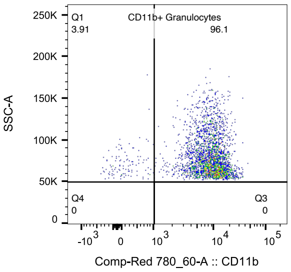

[20180731 Batch2_525_008.fcs]
Granulocytes
2663

Monocytes

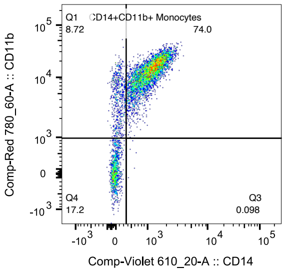

[20180731 Batch2_525_008.fcs]
Monocytes
6098

Lymphocytes

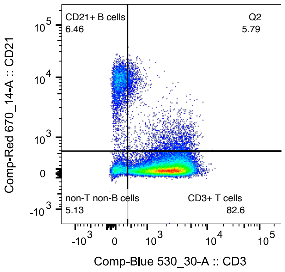

[20180731 Batch2_525_008.fcs]
Lymphocytes
39338

non-T non-B

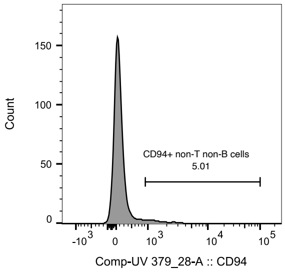

[20180731 Batch2_525_008.fcs]
non-T non-B cells
2017

525
31-JUL-2018

T cells

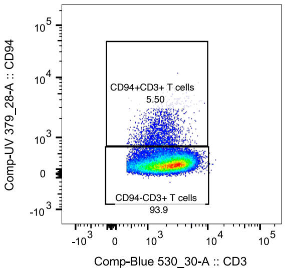

[20180731 Batch2_525_008.fcs]
CD3+ T cells
32503

CD94- T cells

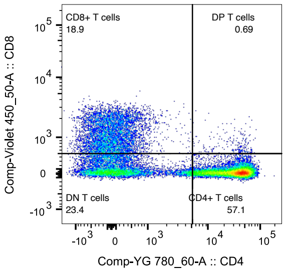

[20180731 Batch2_525_008.fcs]
CD94-CD3+ T cells
30513

CD4 T cells

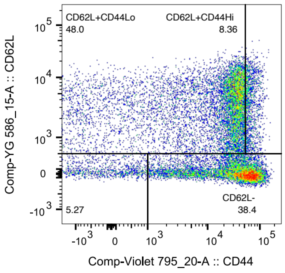

[20180731 Batch2_525_008.fcs]
CD4+ T cells
17414

CD4 T cells

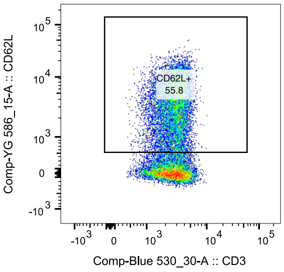

[20180731 Batch2_525_008.fcs]
CD4+ T cells
17414

CD8 T cells

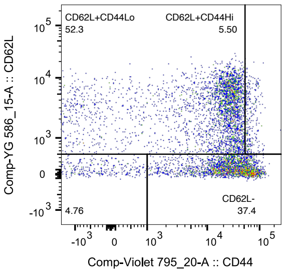

[20180731 Batch2_525_008.fcs]
CD8+ T cells
5762

CD8 T cells

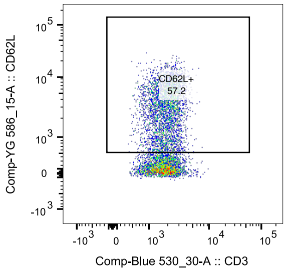

[20180731 Batch2_525_008.fcs]
CD8+ T cells
5762

DN T cells

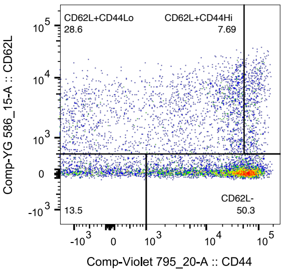

[20180731 Batch2_525_008.fcs]
DN T cells
7126

DN T cells

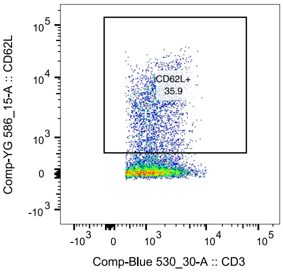

[20180731 Batch2_525_008.fcs]
DN T cells
7126

DP T cells

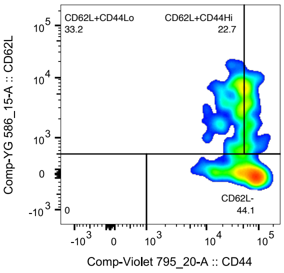

[20180731 Batch2_525_008.fcs]
DP T cells
211

DP T cells

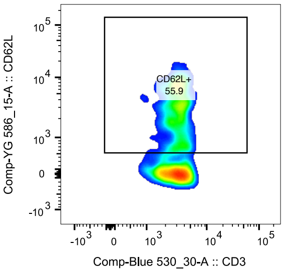

[20180731 Batch2_525_008.fcs]
DP T cells
211

536
31-JUL-2018

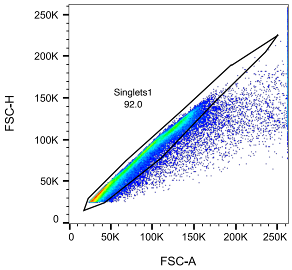

[20180731 Batch2_536_011.fcs]
Ungated
72284

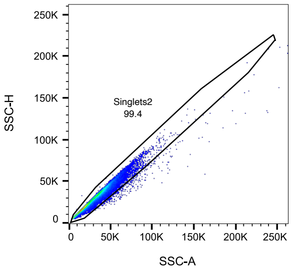

[20180731 Batch2_536_011.fcs]
Singlets1
66484

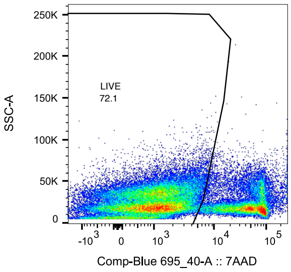

[20180731 Batch2_536_011.fcs]
Singlets2
66118

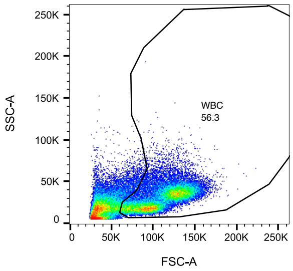

[20180731 Batch2_536_011.fcs]
LIVE
47677

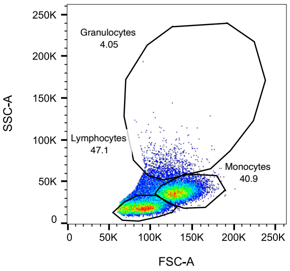

[20180731 Batch2_536_011.fcs]
WBC
26820

Granulocytes

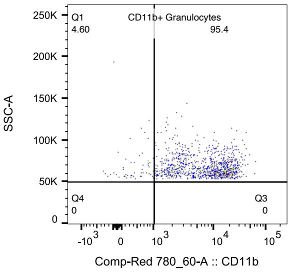

[20180731 Batch2_536_011.fcs]
Granulocytes
1087

Monocytes

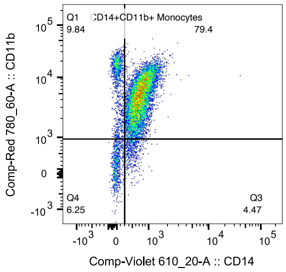

[20180731 Batch2_536_011.fcs]
Monocytes
10973

Lymphocytes

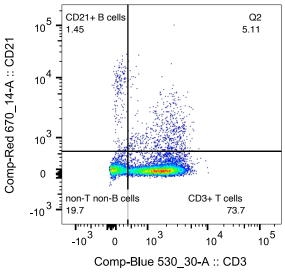

[20180731 Batch2_536_011.fcs]
Lymphocytes
12645

non-T non-B

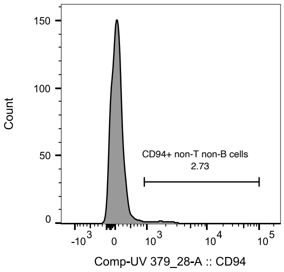

[20180731 Batch2_536_011.fcs]
non-T non-B cells
2495

536
31-JUL-2018

T cells

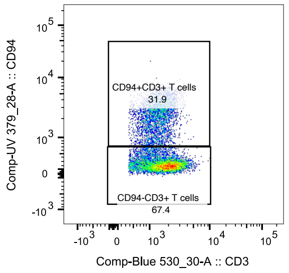

[20180731 Batch2_536_011.fcs]
CD3+ T cells
9321

CD94- T cells

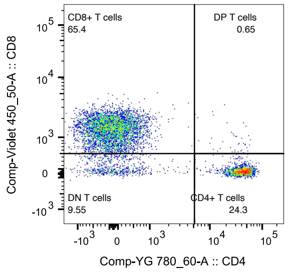

[20180731 Batch2_536_011.fcs]
CD94-CD3+ T cells
6280

CD4 T cells

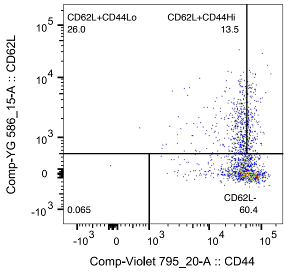

[20180731 Batch2_536_011.fcs]
CD4+ T cells
1529

CD4 T cells

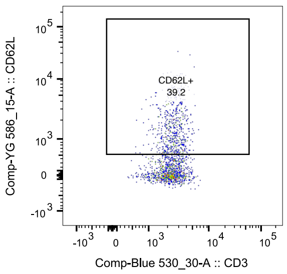

[20180731 Batch2_536_011.fcs]
CD4+ T cells
1529

CD8 T cells

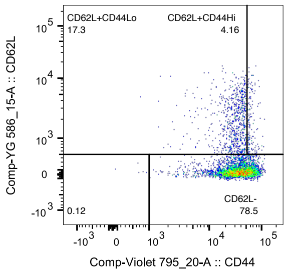

[20180731 Batch2_536_011.fcs]
CD8+ T cells
4110

CD8 T cells

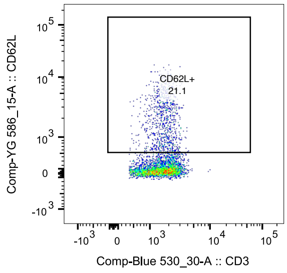

[20180731 Batch2_536_011.fcs]
CD8+ T cells
4110

DN T cells

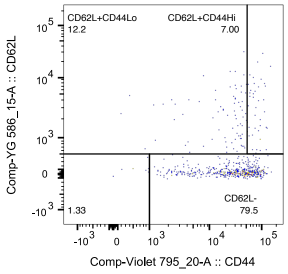

[20180731 Batch2_536_011.fcs]
DN T cells
600

DN T cells

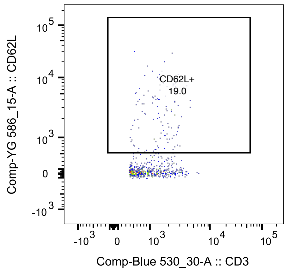

[20180731 Batch2_536_011.fcs]
DN T cells
600

DP T cells

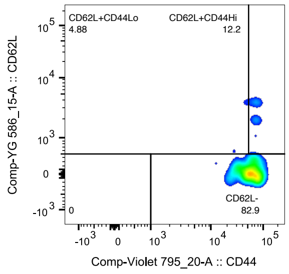

[20180731 Batch2_536_011.fcs]
DP T cells
41.0

DP T cells

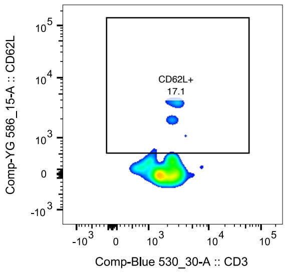

[20180731 Batch2_536_011.fcs]
DP T cells
41.0

571
31-JUL-2018

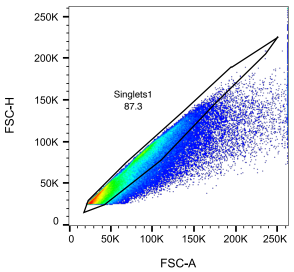

[20180731 Batch2_571_016.fcs]
Ungated
145954

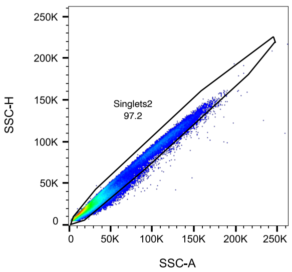

[20180731 Batch2_571_016.fcs]
Singlets1
127457

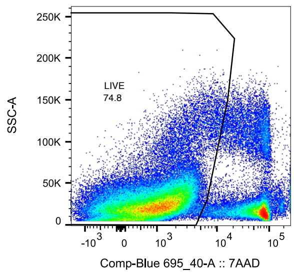

[20180731 Batch2_571_016.fcs]
Singlets2
123949

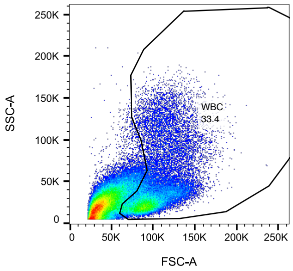

[20180731 Batch2_571_016.fcs]
LIVE
92766

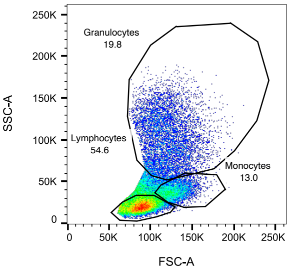

[20180731 Batch2_571_016.fcs]
WBC
30951

Granulocytes

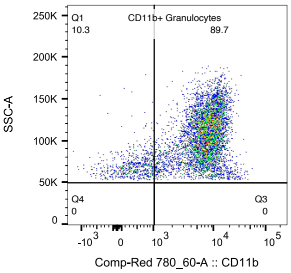

[20180731 Batch2_571_016.fcs]
Granulocytes
6124

Monocytes

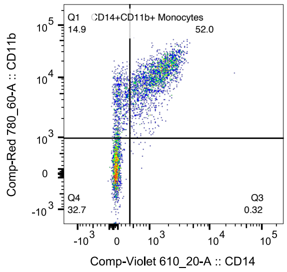

[20180731 Batch2_571_016.fcs]
Monocytes
4029

Lymphocytes

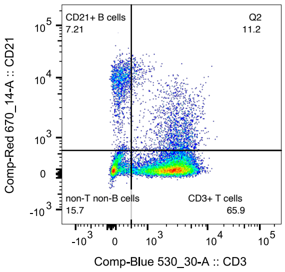

[20180731 Batch2_571_016.fcs]
Lymphocytes
16910

non-T non-B

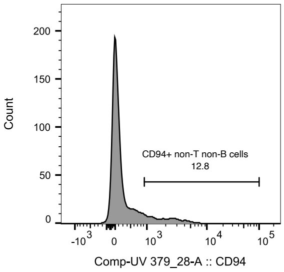

[20180731 Batch2_571_016.fcs]
non-T non-B cells
2647

571
31-JUL-2018

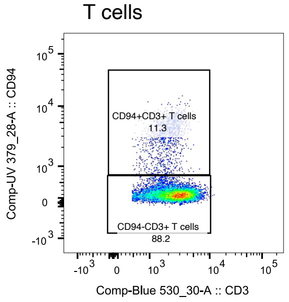

[20180731 Batch2_571_016.fcs]
CD3+ T cells
11147

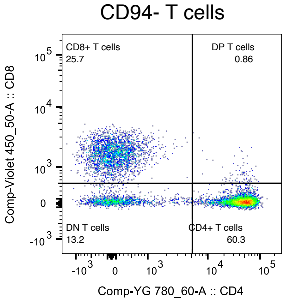

[20180731 Batch2_571_016.fcs]
CD94-CD3+ T cells
9837

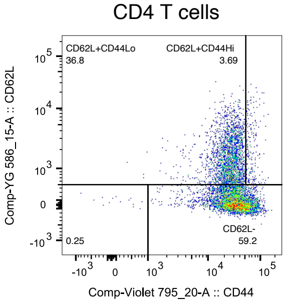

[20180731 Batch2_571_016.fcs]
CD4+ T cells
5932

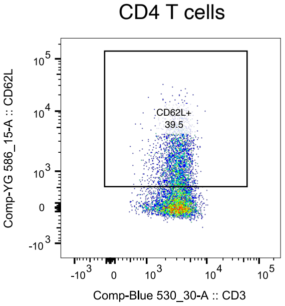

[20180731 Batch2_571_016.fcs]
CD4+ T cells
5932

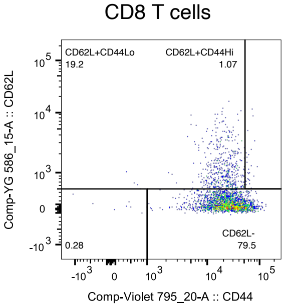

[20180731 Batch2_571_016.fcs]
CD8+ T cells
2526

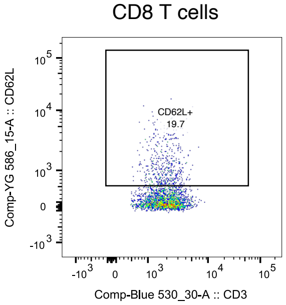

[20180731 Batch2_571_016.fcs]
CD8+ T cells
2526

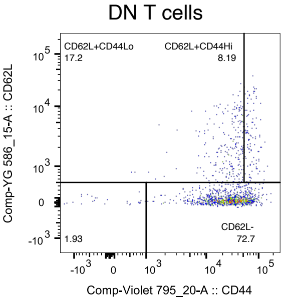

[20180731 Batch2_571_016.fcs]
DN T cells
1294

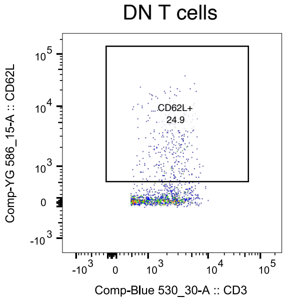

[20180731 Batch2_571_016.fcs]
DN T cells
1294

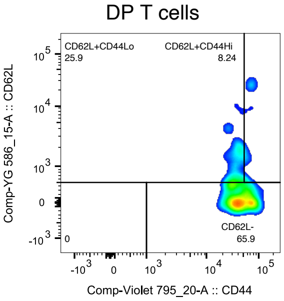

[20180731 Batch2_571_016.fcs]
DP T cells
85.0

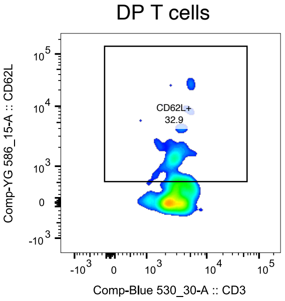

[20180731 Batch2_571_016.fcs]
DP T cells
85.0

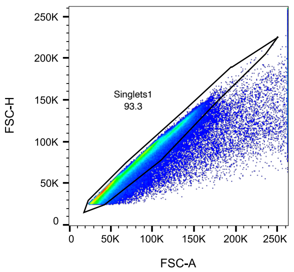

[20180731 Batch2_628_023.fcs]
Ungated
244554

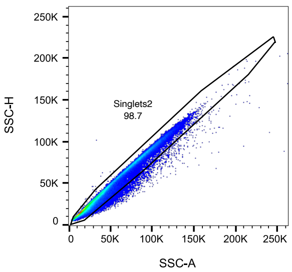

[20180731 Batch2_628_023.fcs]
Singlets1
228192

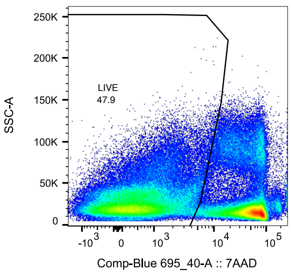

[20180731 Batch2_628_023.fcs]
Singlets2
225233

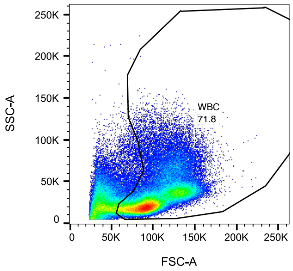

[20180731 Batch2_628_023.fcs]
LIVE
107965

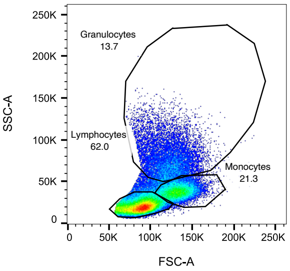

[20180731 Batch2_628_023.fcs]
WBC
77525

Granulocytes

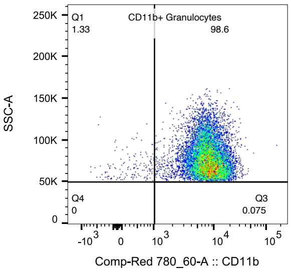

[20180731 Batch2_628_023.fcs]
Granulocytes
10656

Monocytes

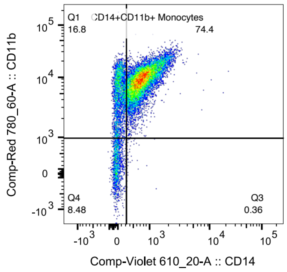

[20180731 Batch2_628_023.fcs]
Monocytes
16479

Lymphocytes

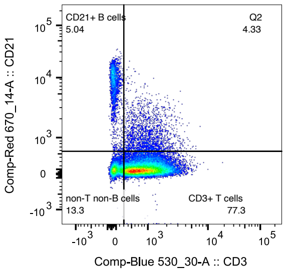

[20180731 Batch2_628_023.fcs]
Lymphocytes
48072

non-T non-B

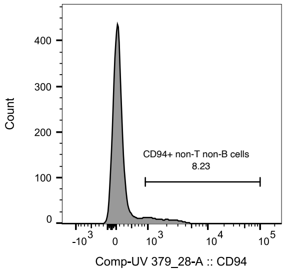

[20180731 Batch2_628_023.fcs]
non-T non-B cells
6406

628
31-JUL-2018

T cells

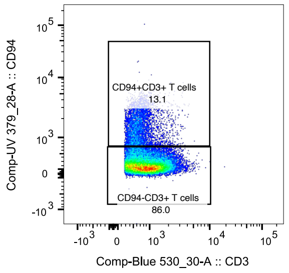

[20180731 Batch2_628_023.fcs]
CD3+ T cells
37164

CD94- T cells

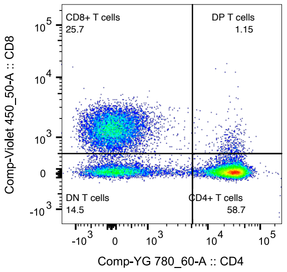

[20180731 Batch2_628_023.fcs]
CD94-CD3+ T cells
31963

CD4 T cells

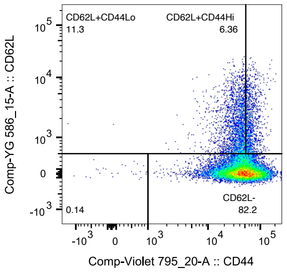

[20180731 Batch2_628_023.fcs]
CD4+ T cells
18763

CD4 T cells

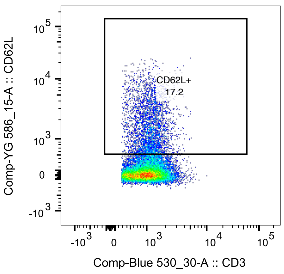

[20180731 Batch2_628_023.fcs]
CD4+ T cells
18763

CD8 T cells

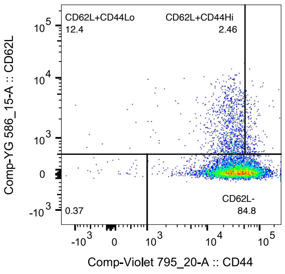

[20180731 Batch2_628_023.fcs]
CD8+ T cells
8211

CD8 T cells

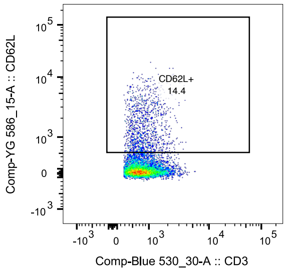

[20180731 Batch2_628_023.fcs]
CD8+ T cells
8211

DN T cells

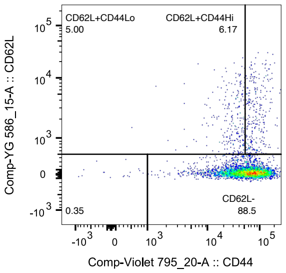

[20180731 Batch2_628_023.fcs]
DN T cells
4621

DN T cells

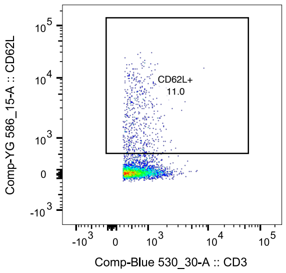

[20180731 Batch2_628_023.fcs]
DN T cells
4621

DP T cells

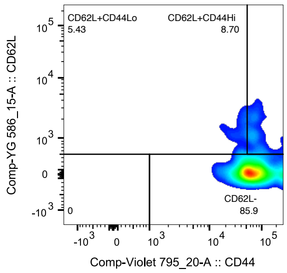

[20180731 Batch2_628_023.fcs]
DP T cells
368

DP T cells

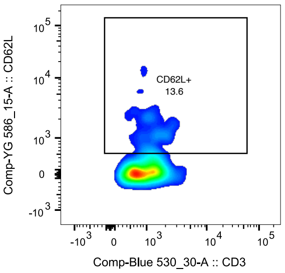

[20180731 Batch2_628_023.fcs]
DP T cells
368

508
31-JUL-2018

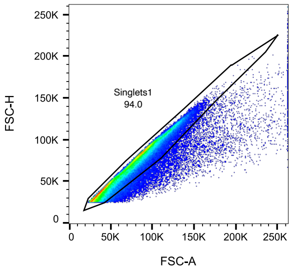

[20180731 Batch2_508_003.fcs]
Ungated
154881

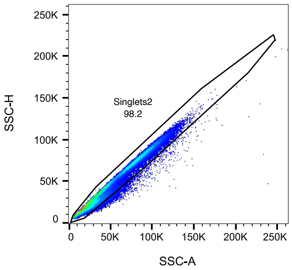

[20180731 Batch2_508_003.fcs]
Singlets1
145608

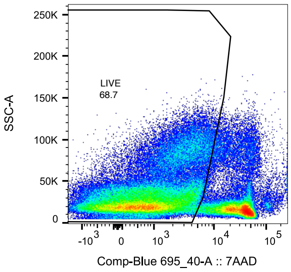

[20180731 Batch2_508_003.fcs]
Singlets2
142996

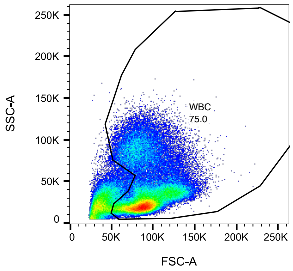

[20180731 Batch2_508_003.fcs]
LIVE
98264

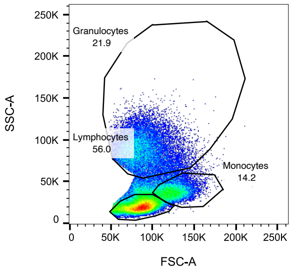

[20180731 Batch2_508_003.fcs]
WBC
73725

Granulocytes

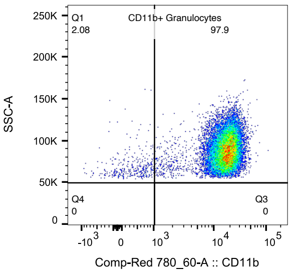

[20180731 Batch2_508_003.fcs]
Granulocytes
16139

Monocytes

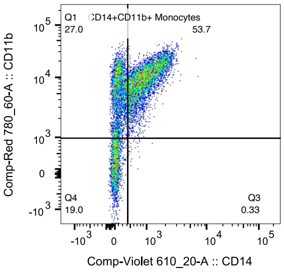

[20180731 Batch2_508_003.fcs]
Monocytes
10452

Lymphocytes

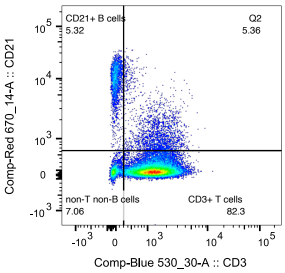

[20180731 Batch2_508_003.fcs]
Lymphocytes
41310

non-T non-B

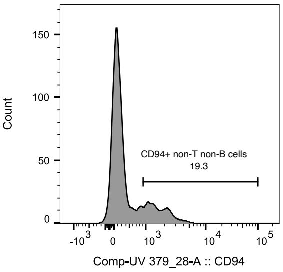

[20180731 Batch2_508_003.fcs]
non-T non-B cells
2916

508
31-JUL-2018

T cells

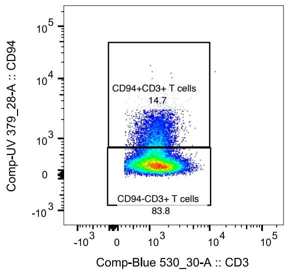

[20180731 Batch2_508_003.fcs]
CD3+ T cells
33984

CD94- T cells

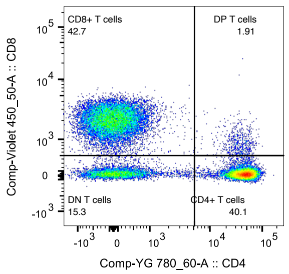

[20180731 Batch2_508_003.fcs]
CD94-CD3+ T cells
28492

CD4 T cells

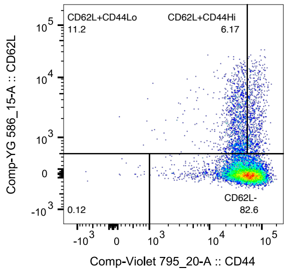

[20180731 Batch2_508_003.fcs]
CD4+ T cells
11416

CD4 T cells

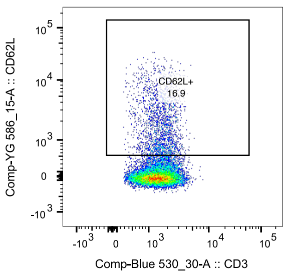

[20180731 Batch2_508_003.fcs]
CD4+ T cells
11416

CD8 T cells

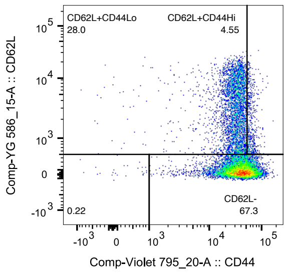

[20180731 Batch2_508_003.fcs]
CD8+ T cells
12166

CD8 T cells

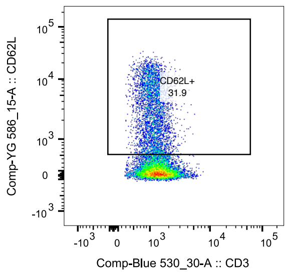

[20180731 Batch2_508_003.fcs]
CD8+ T cells
12166

DN T cells

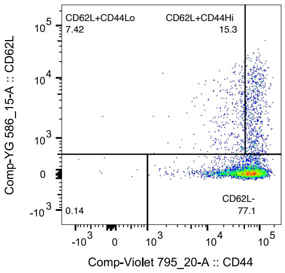

[20180731 Batch2_508_003.fcs]
DN T cells
4365

DN T cells

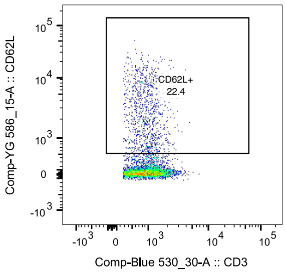

[20180731 Batch2_508_003.fcs]
DN T cells
4365

DP T cells

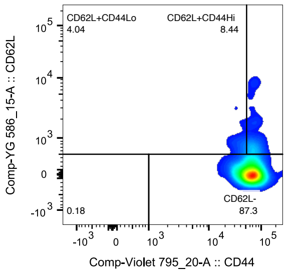

[20180731 Batch2_508_003.fcs]
DP T cells
545

DP T cells

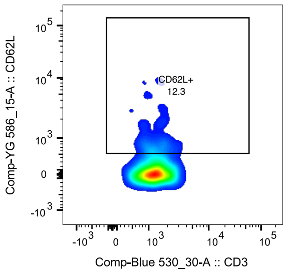

[20180731 Batch2_508_003.fcs]
DP T cells
545

511
31-JUL-2018

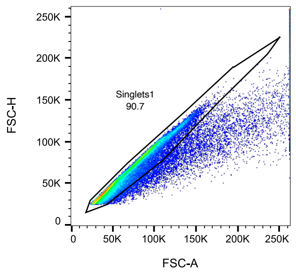

[20180731 Batch2_511_004.fcs]
Ungated
85435

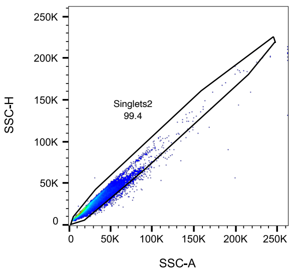

[20180731 Batch2_511_004.fcs]
Singlets1
77457

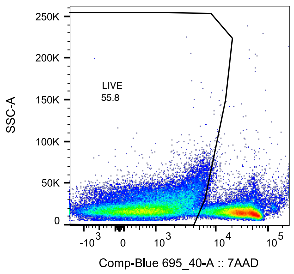

[20180731 Batch2_511_004.fcs]
Singlets2
76990

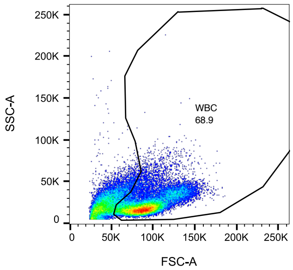

[20180731 Batch2_511_004.fcs]
LIVE
42954

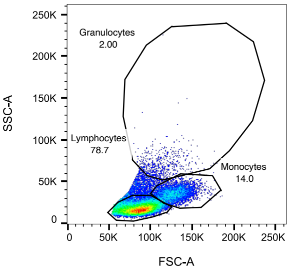

[20180731 Batch2_511_004.fcs]
WBC
29579

Granulocytes

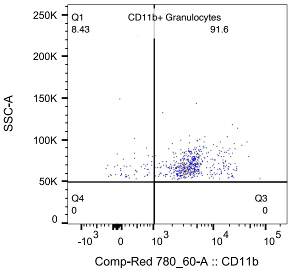

[20180731 Batch2_511_004.fcs]
Granulocytes
593

Monocytes

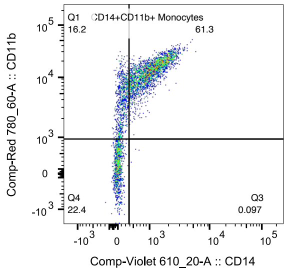

[20180731 Batch2_511_004.fcs]
Monocytes
4130

Lymphocytes

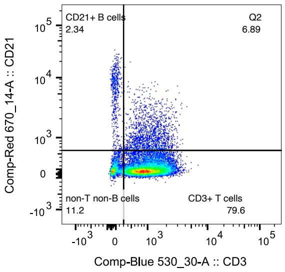

[20180731 Batch2_511_004.fcs]
Lymphocytes
23280

non-T non-B

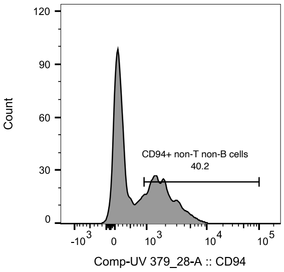

[20180731 Batch2_511_004.fcs]
non-T non-B cells
2600

511
31-JUL-2018

T cells

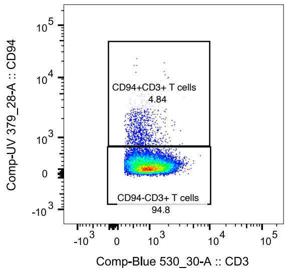

[20180731 Batch2_511_004.fcs]
CD3+ T cells
18530

CD94- T cells

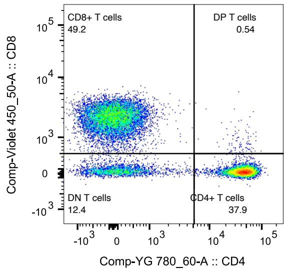

[20180731 Batch2_511_004.fcs]
CD94-CD3+ T cells
17563

CD4 T cells

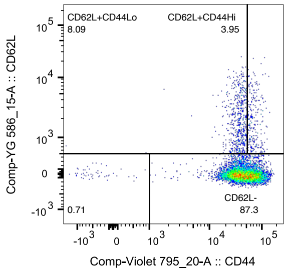

[20180731 Batch2_511_004.fcs]
CD4+ T cells
6653

CD4 T cells

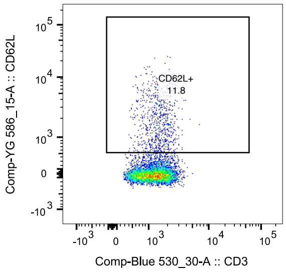

[20180731 Batch2_511_004.fcs]
CD4+ T cells
6653

CD8 T cells

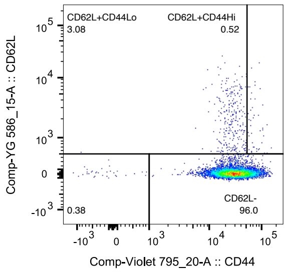

[20180731 Batch2_511_004.fcs]
CD8+ T cells
8633

CD8 T cells

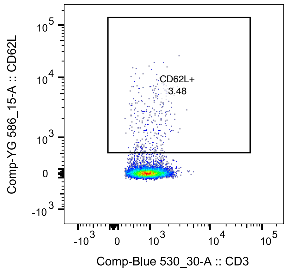

[20180731 Batch2_511_004.fcs]
CD8+ T cells
8633

DN T cells

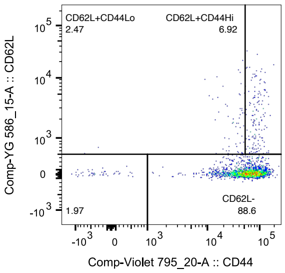

[20180731 Batch2_511_004.fcs]
DN T cells
2182

DN T cells

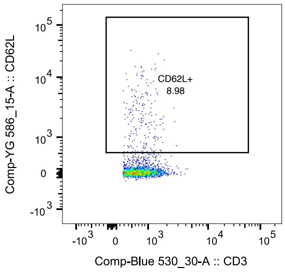

[20180731 Batch2_511_004.fcs]
DN T cells
2182

DP T cells

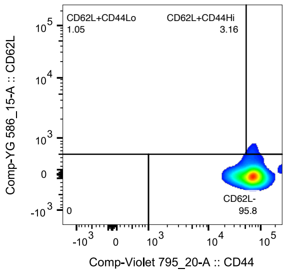

[20180731 Batch2_511_004.fcs]
DP T cells
95.0

DP T cells

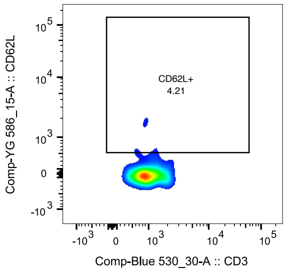

[20180731 Batch2_511_004.fcs]
DP T cells
95.0

501
31-JUL-2018

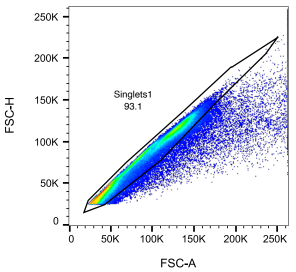

[20180731 Batch2_501_001.fcs]
Ungated
181655

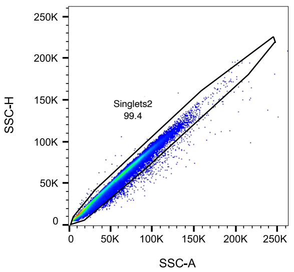

[20180731 Batch2_501_001.fcs]
Singlets1
169037

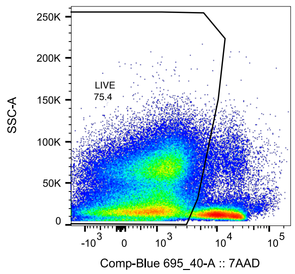

[20180731 Batch2_501_001.fcs]
Singlets2
168089

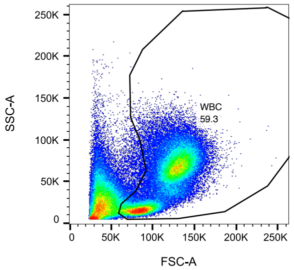

[20180731 Batch2_501_001.fcs]
LIVE
126813

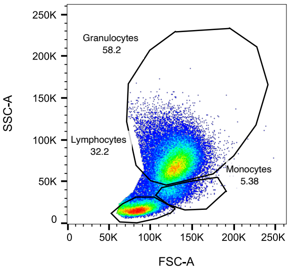

[20180731 Batch2_501_001.fcs]
WBC
75246

Granulocytes

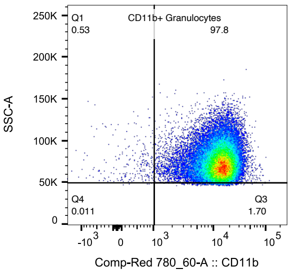

[20180731 Batch2_501_001.fcs]
Granulocytes
43789

Monocytes

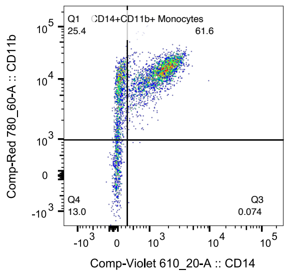

[20180731 Batch2_501_001.fcs]
Monocytes
4047

Lymphocytes

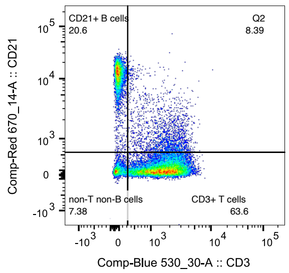

[20180731 Batch2_501_001.fcs]
Lymphocytes
24260

non-T non-B

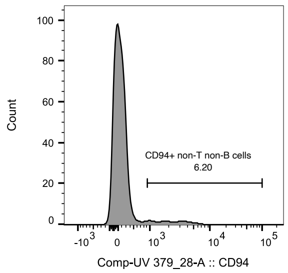

[20180731 Batch2_501_001.fcs]
non-T non-B cells
1790

501
31-JUL-2018

T cells

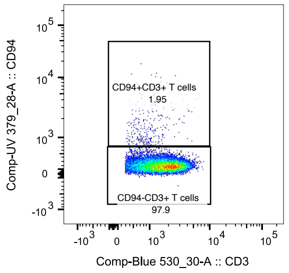

[20180731 Batch2_501_001.fcs]
CD3+ T cells
15440

CD94- T cells

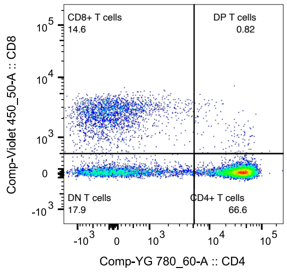

[20180731 Batch2_501_001.fcs]
CD94-CD3+ T cells
15114

CD4 T cells

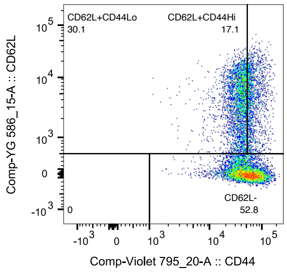

[20180731 Batch2_501_001.fcs]
CD4+ T cells
10066

CD4 T cells

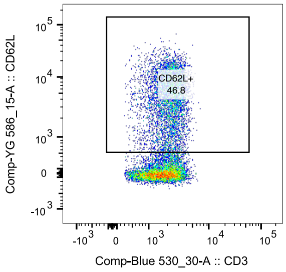

[20180731 Batch2_501_001.fcs]
CD4+ T cells
10066

CD8 T cells

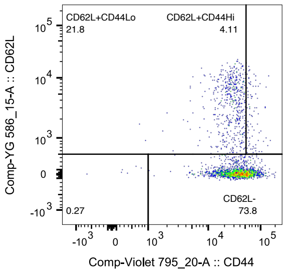

[20180731 Batch2_501_001.fcs]
CD8+ T cells
2212

CD8 T cells

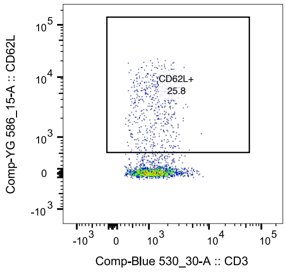

[20180731 Batch2_501_001.fcs]
CD8+ T cells
2212

DN T cells

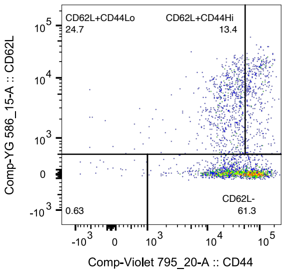

[20180731 Batch2_501_001.fcs]
DN T cells
2712

DN T cells

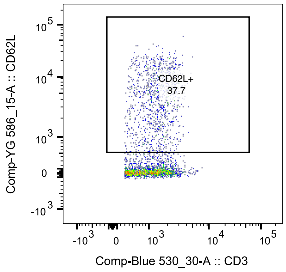

[20180731 Batch2_501_001.fcs]
DN T cells
2712

DP T cells

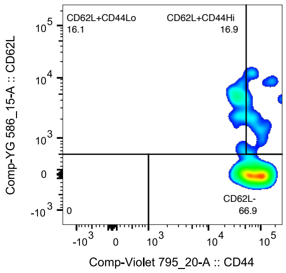

[20180731 Batch2_501_001.fcs]
DP T cells
124

DP T cells

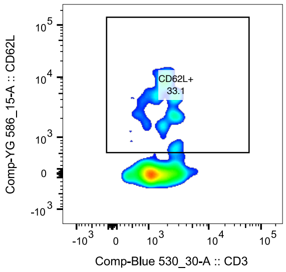

[20180731 Batch2_501_001.fcs]
DP T cells
124

560
31-JUL-2018

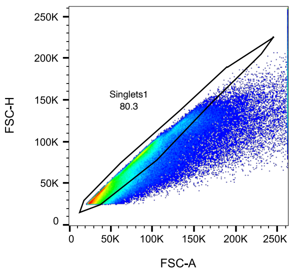

[20180731 Batch2_560_015.fcs]
Ungated
238621

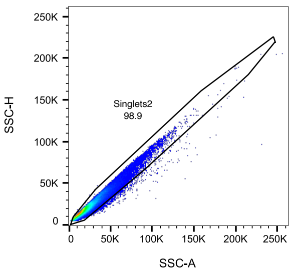

[20180731 Batch2_560_015.fcs]
Singlets1
191606

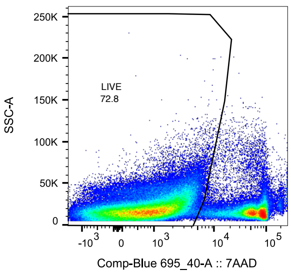

[20180731 Batch2_560_015.fcs]
Singlets2
189469

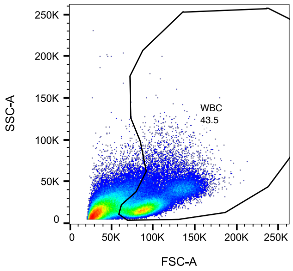

[20180731 Batch2_560_015.fcs]
LIVE
137982

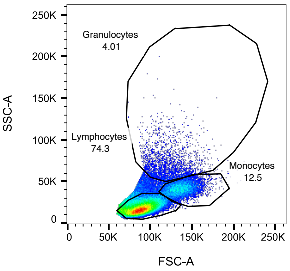

[20180731 Batch2_560_015.fcs]
WBC
59958

Granulocytes

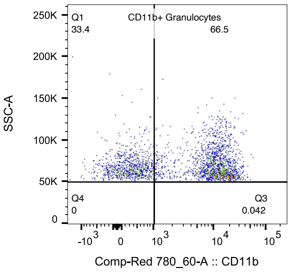

[20180731 Batch2_560_015.fcs]
Granulocytes
2407

Monocytes

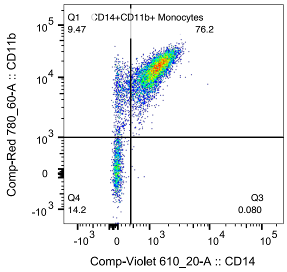

[20180731 Batch2_560_015.fcs]
Monocytes
7504

Lymphocytes

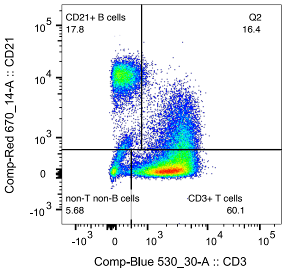

[20180731 Batch2_560_015.fcs]
Lymphocytes
44547

non-T non-B

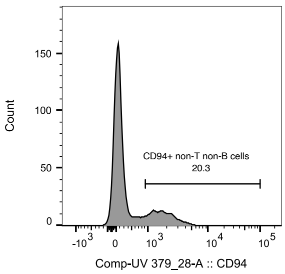

[20180731 Batch2_560_015.fcs]
non-T non-B cells
2530

560
31-JUL-2018

T cells

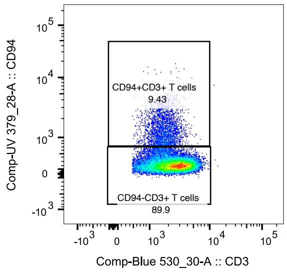

[20180731 Batch2_560_015.fcs]
CD3+ T cells
26769

CD94- T cells

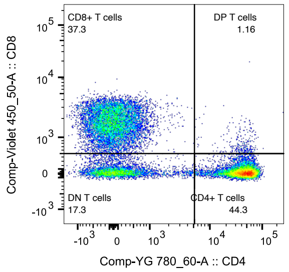

[20180731 Batch2_560_015.fcs]
CD94-CD3+ T cells
24064

CD4 T cells

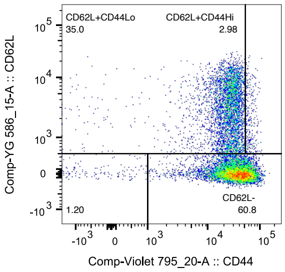

[20180731 Batch2_560_015.fcs]
CD4+ T cells
10657

CD4 T cells

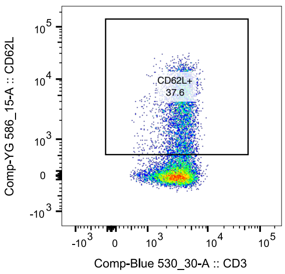

[20180731 Batch2_560_015.fcs]
CD4+ T cells
10657

CD8 T cells

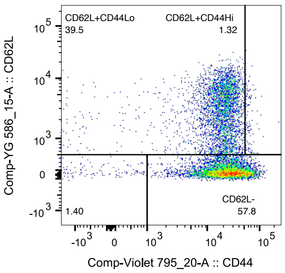

[20180731 Batch2_560_015.fcs]
CD8+ T cells
8972

CD8 T cells

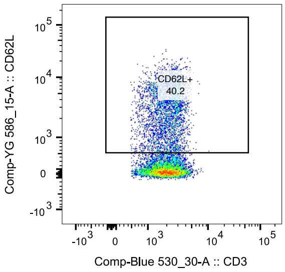

[20180731 Batch2_560_015.fcs]
CD8+ T cells
8972

DN T cells

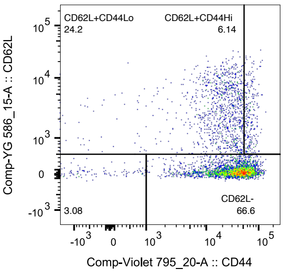

[20180731 Batch2_560_015.fcs]
DN T cells
4155

DN T cells

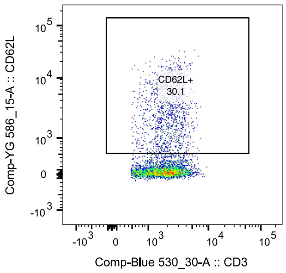

[20180731 Batch2_560_015.fcs]
DN T cells
4155

DP T cells

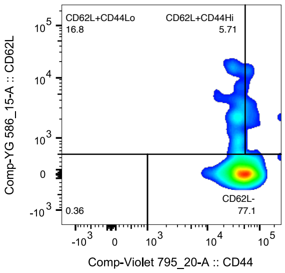

[20180731 Batch2_560_015.fcs]
DP T cells
280

DP T cells

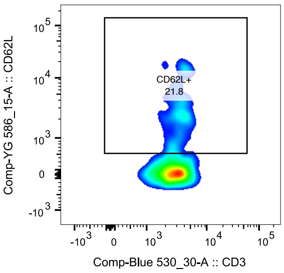

[20180731 Batch2_560_015.fcs]
DP T cells
280

577
31-JUL-2018

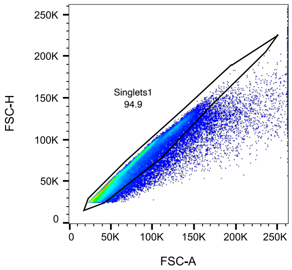

[20180731 Batch2_577_017.fcs]
Ungated
155563

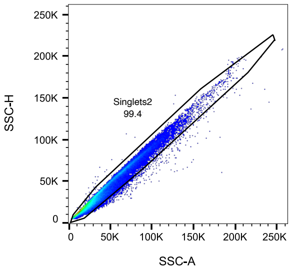

[20180731 Batch2_577_017.fcs]
Singlets1
147704

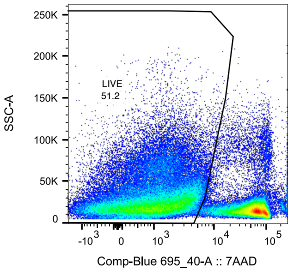

[20180731 Batch2_577_017.fcs]
Singlets2
146744

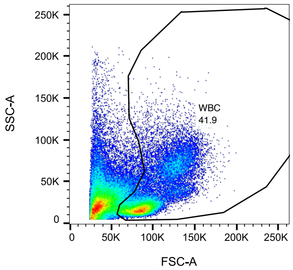

[20180731 Batch2_577_017.fcs]
LIVE
75202

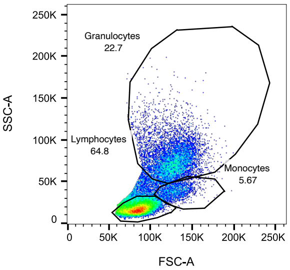

[20180731 Batch2_577_017.fcs]
WBC
31547

Granulocytes

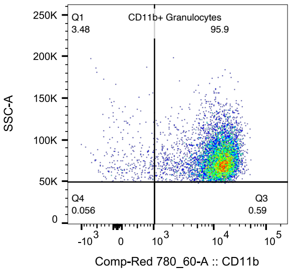

[20180731 Batch2_577_017.fcs]
Granulocytes
7176

Monocytes

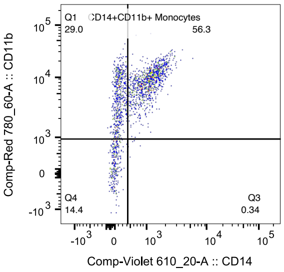

[20180731 Batch2_577_017.fcs]
Monocytes
1789

Lymphocytes

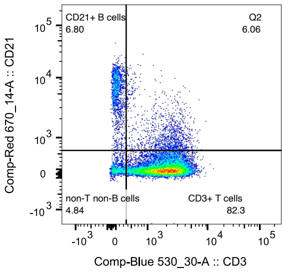

[20180731 Batch2_577_017.fcs]
Lymphocytes
20441

non-T non-B

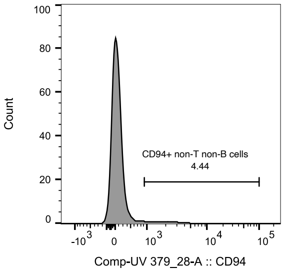

[20180731 Batch2_577_017.fcs]
non-T non-B cells
990

577
31-JUL-2018

T cells

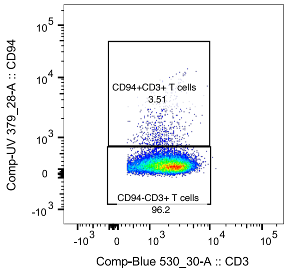

[20180731 Batch2_577_017.fcs]
CD3+ T cells
16824

CD94- T cells

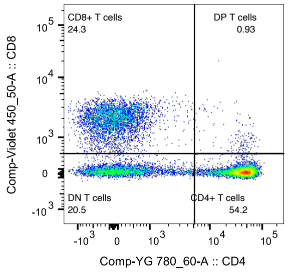

[20180731 Batch2_577_017.fcs]
CD94-CD3+ T cells
16188

CD4 T cells

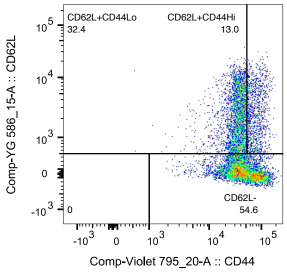

[20180731 Batch2_577_017.fcs]
CD4+ T cells
8781

CD4 T cells

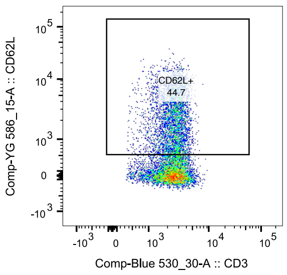

[20180731 Batch2_577_017.fcs]
CD4+ T cells
8781

CD8 T cells

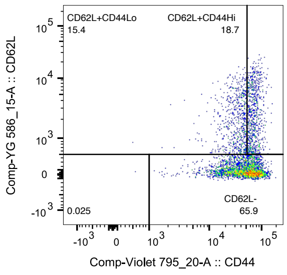

[20180731 Batch2_577_017.fcs]
CD8+ T cells
3935

CD8 T cells

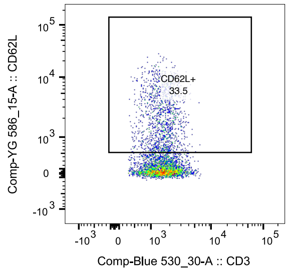

[20180731 Batch2_577_017.fcs]
CD8+ T cells
3935

DN T cells

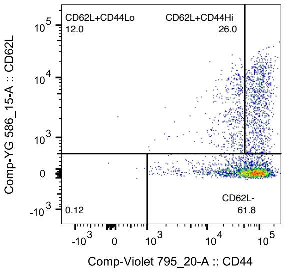

[20180731 Batch2_577_017.fcs]
DN T cells
3321

DN T cells

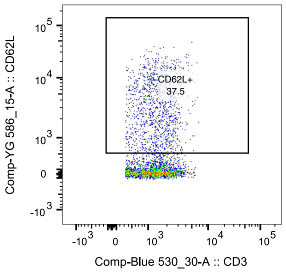

[20180731 Batch2_577_017.fcs]
DN T cells
3321

DP T cells

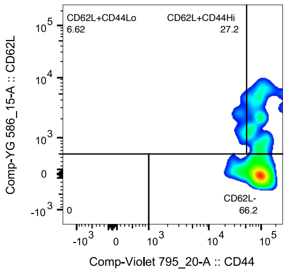

[20180731 Batch2_577_017.fcs]
DP T cells
151

DP T cells

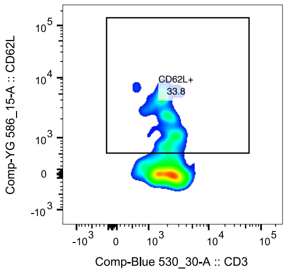

[20180731 Batch2_577_017.fcs]
DP T cells
151

629
31-JUL-2018

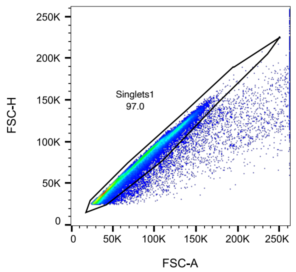

[20180731 Batch2_629_024.fcs]
Ungated
129771

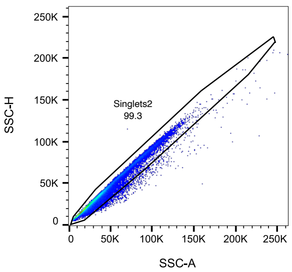

[20180731 Batch2_629_024.fcs]
Singlets1
125940

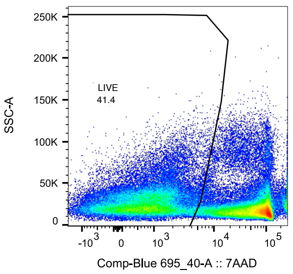

[20180731 Batch2_629_024.fcs]
Singlets2
125107

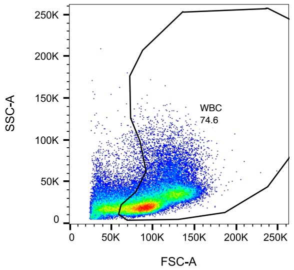

[20180731 Batch2_629_024.fcs]
LIVE
51837

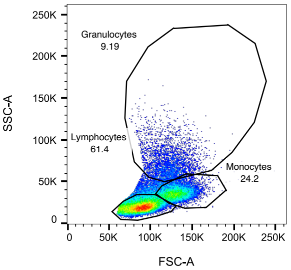

[20180731 Batch2_629_024.fcs]
WBC
38696

Granulocytes

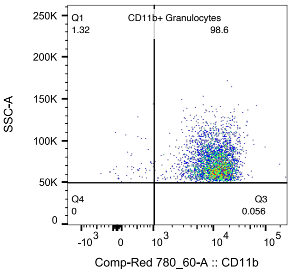

[20180731 Batch2_629_024.fcs]
Granulocytes
3555

Monocytes

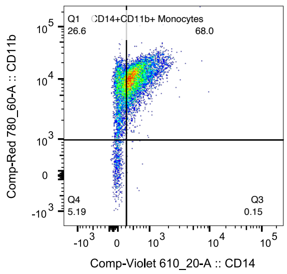

[20180731 Batch2_629_024.fcs]
Monocytes
9363

Lymphocytes

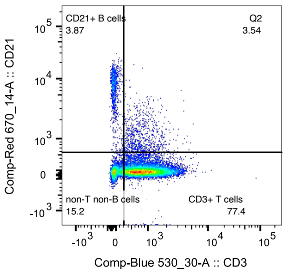

[20180731 Batch2_629_024.fcs]
Lymphocytes
23776

non-T non-B

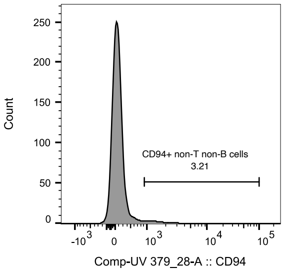

[20180731 Batch2_629_024.fcs]
non-T non-B cells
3616

629
31-JUL-2018

T cells

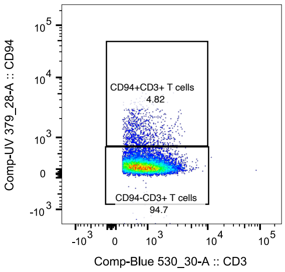

[20180731 Batch2_629_024.fcs]
CD3+ T cells
18397

CD94- T cells

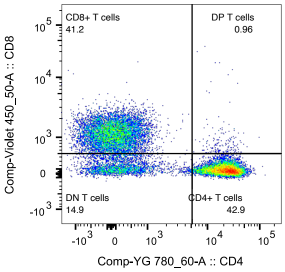

[20180731 Batch2_629_024.fcs]
CD94-CD3+ T cells
17428

CD4 T cells

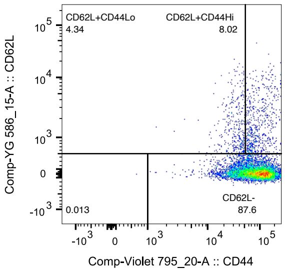

[20180731 Batch2_629_024.fcs]
CD4+ T cells
7483

CD4 T cells

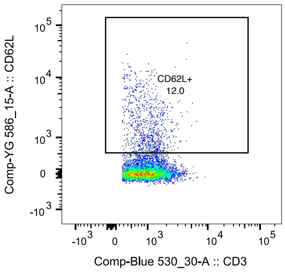

[20180731 Batch2_629_024.fcs]
CD4+ T cells
7483

CD8 T cells

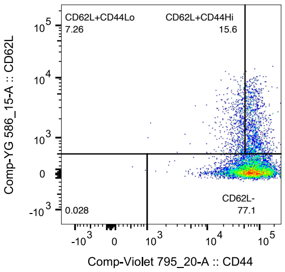

[20180731 Batch2_629_024.fcs]
CD8+ T cells
7181

CD8 T cells

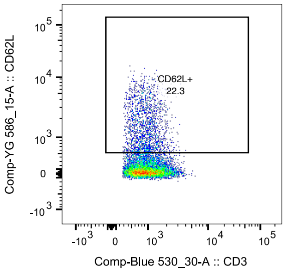

[20180731 Batch2_629_024.fcs]
CD8+ T cells
7181

DN T cells

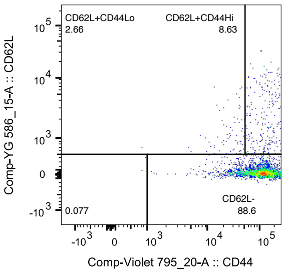

[20180731 Batch2_629_024.fcs]
DN T cells
2597

DN T cells

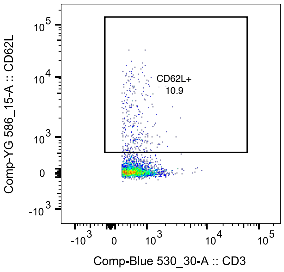

[20180731 Batch2_629_024.fcs]
DN T cells
2597

DP T cells

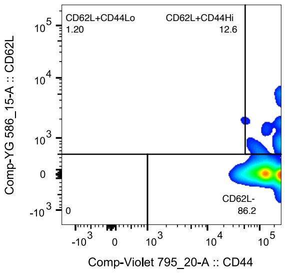

[20180731 Batch2_629_024.fcs]
DP T cells
167

DP T cells

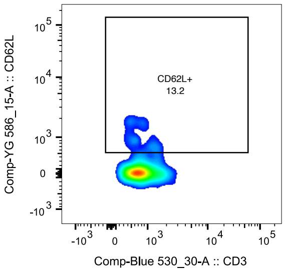

[20180731 Batch2_629_024.fcs]
DP T cells
167

513
31-JUL-2018

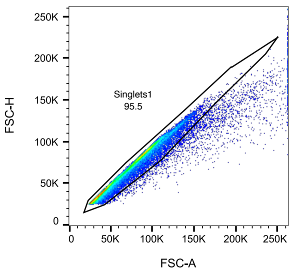

[20180731 Batch2_513_005.fcs]
Ungated
47009

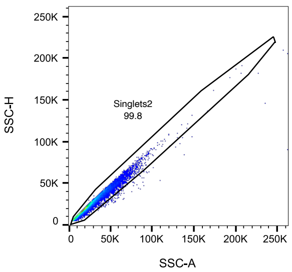

[20180731 Batch2_513_005.fcs]
Singlets1
44881

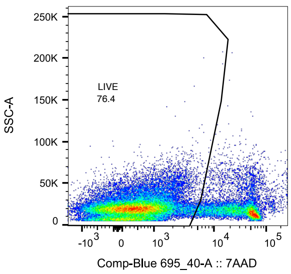

[20180731 Batch2_513_005.fcs]
Singlets2
44774

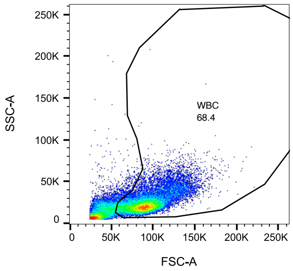

[20180731 Batch2_513_005.fcs]
LIVE
34192

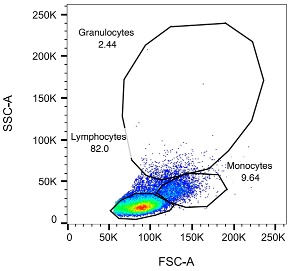

[20180731 Batch2_513_005.fcs]
WBC
23381

Granulocytes

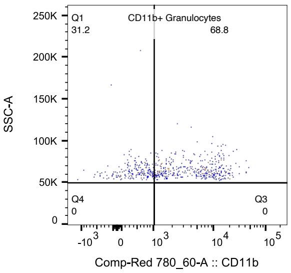

[20180731 Batch2_513_005.fcs]
Granulocytes
571

Monocytes

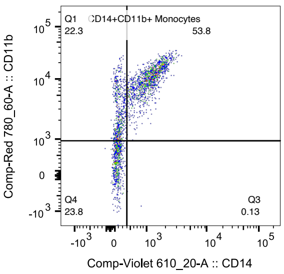

[20180731 Batch2_513_005.fcs]
Monocytes
2253

Lymphocytes

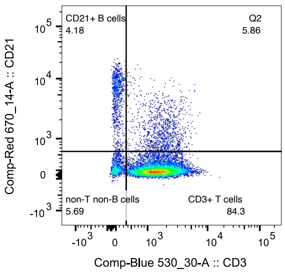

[20180731 Batch2_513_005.fcs]
Lymphocytes
19171

non-T non-B

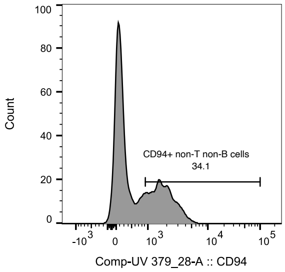

[20180731 Batch2_513_005.fcs]
non-T non-B cells
1090

513
31-JUL-2018

T cells

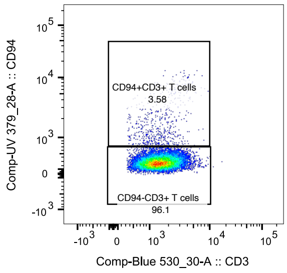

[20180731 Batch2_513_005.fcs]
CD3+ T cells
16157

CD94- T cells

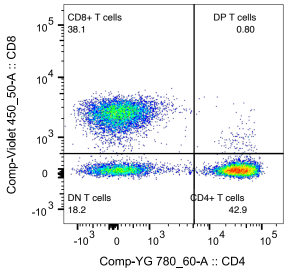

[20180731 Batch2_513_005.fcs]
CD94-CD3+ T cells
15525

CD4 T cells

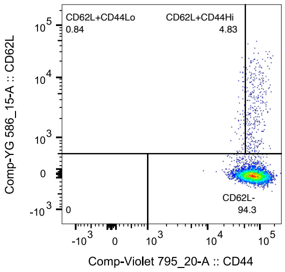

[20180731 Batch2_513_005.fcs]
CD4+ T cells
6661

CD4 T cells

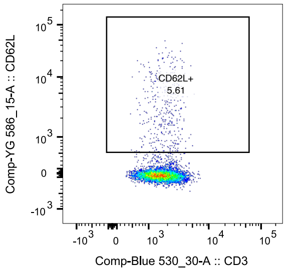

[20180731 Batch2_513_005.fcs]
CD4+ T cells
6661

CD8 T cells

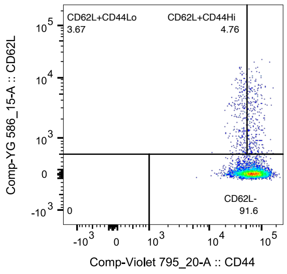

[20180731 Batch2_513_005.fcs]
CD8+ T cells
5919

CD8 T cells

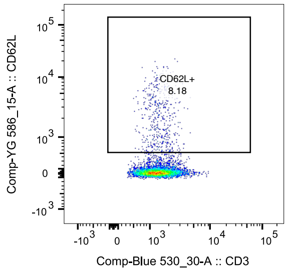

[20180731 Batch2_513_005.fcs]
CD8+ T cells
5919

DN T cells

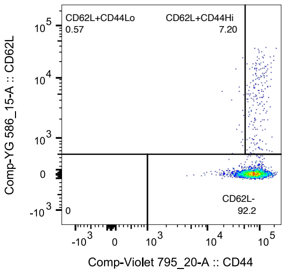

[20180731 Batch2_513_005.fcs]
DN T cells
2821

DN T cells

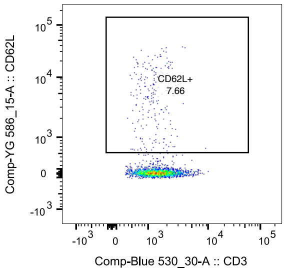

[20180731 Batch2_513_005.fcs]
DN T cells
2821

DP T cells

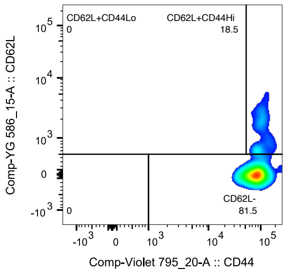

[20180731 Batch2_513_005.fcs]
DP T cells
124

DP T cells

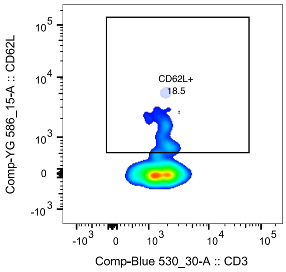

[20180731 Batch2_513_005.fcs]
DP T cells
124

604
31-JUL-2018

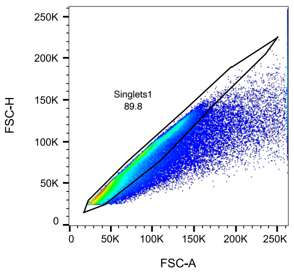

[20180731 Batch2_604_021.fcs]
Ungated
195121

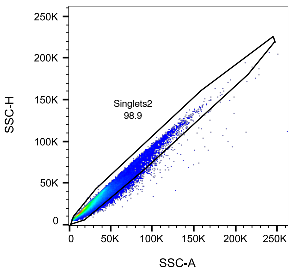

[20180731 Batch2_604_021.fcs]
Singlets1
175268

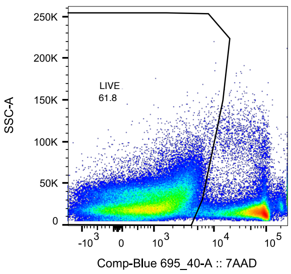

[20180731 Batch2_604_021.fcs]
Singlets2
173349

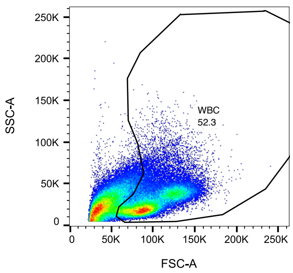

[20180731 Batch2_604_021.fcs]
LIVE
107114

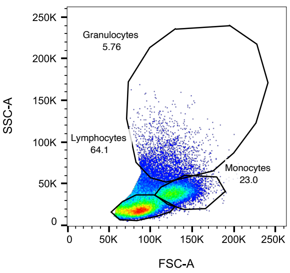

[20180731 Batch2_604_021.fcs]
WBC
56023

Granulocytes

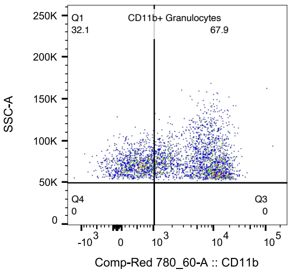

[20180731 Batch2_604_021.fcs]
Granulocytes
3227

Monocytes

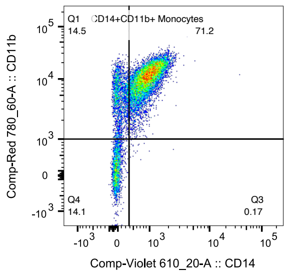

[20180731 Batch2_604_021.fcs]
Monocytes
12908

Lymphocytes

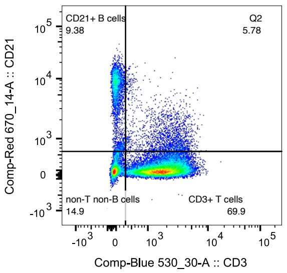

[20180731 Batch2_604_021.fcs]
Lymphocytes
35888

non-T non-B

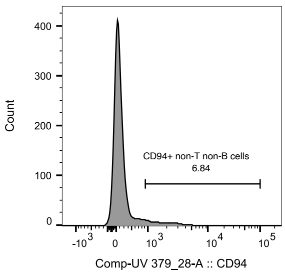

[20180731 Batch2_604_021.fcs]
non-T non-B cells
5365

604
31-JUL-2018

T cells

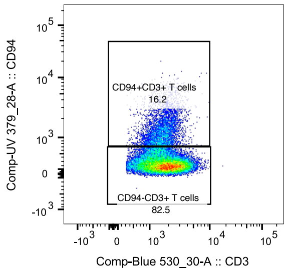

[20180731 Batch2_604_021.fcs]
CD3+ T cells
25079

CD94- T cells

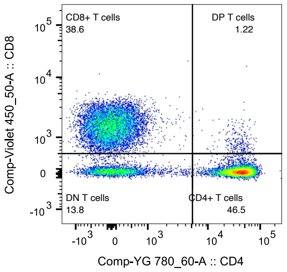

[20180731 Batch2_604_021.fcs]
CD94-CD3+ T cells
20700

CD4 T cells

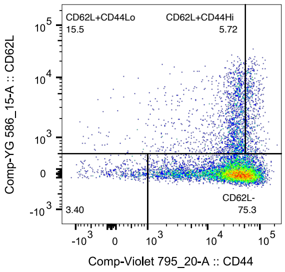

[20180731 Batch2_604_021.fcs]
CD4+ T cells
9618

CD4 T cells

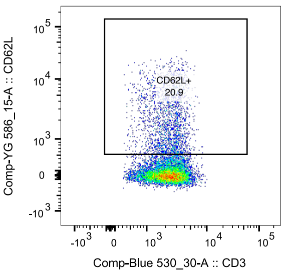

[20180731 Batch2_604_021.fcs]
CD4+ T cells
9618

CD8 T cells

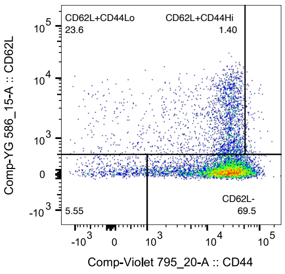

[20180731 Batch2_604_021.fcs]
CD8+ T cells
7983

CD8 T cells

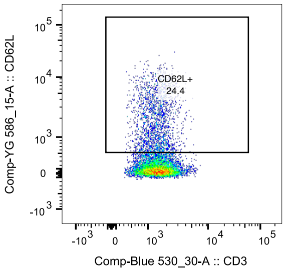

[20180731 Batch2_604_021.fcs]
CD8+ T cells
7983

DN T cells

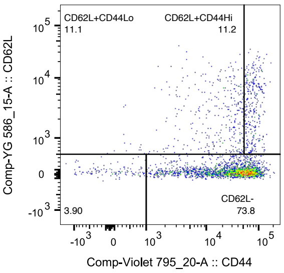

[20180731 Batch2_604_021.fcs]
DN T cells
2847

DN T cells

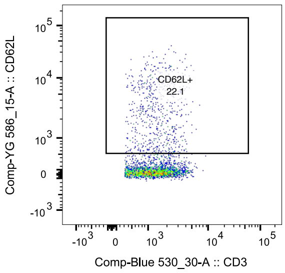

[20180731 Batch2_604_021.fcs]
DN T cells
2847

DP T cells

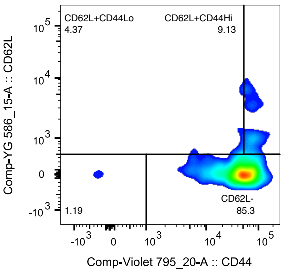

[20180731 Batch2_604_021.fcs]
DP T cells
252

DP T cells

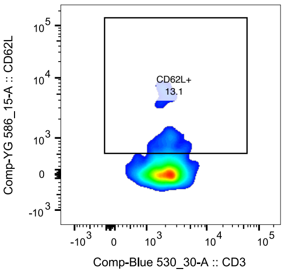

[20180731 Batch2_604_021.fcs]
DP T cells
252

550
31-JUL-2018

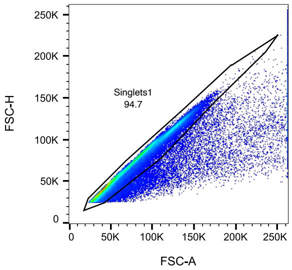

[20180731 Batch2_550_012.fcs]
Ungated
220082

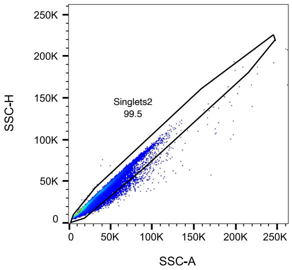

[20180731 Batch2_550_012.fcs]
Singlets1
208510

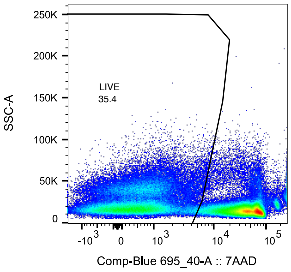

[20180731 Batch2_550_012.fcs]
Singlets2
207447

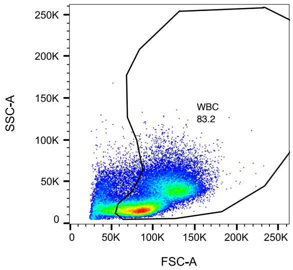

[20180731 Batch2_550_012.fcs]
LIVE
73357

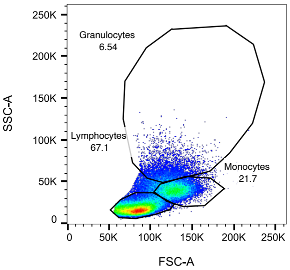

[20180731 Batch2_550_012.fcs]
WBC
60999

Granulocytes

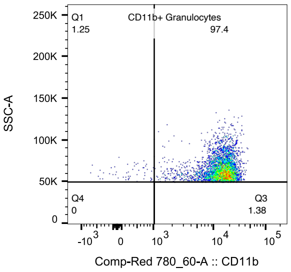

[20180731 Batch2_550_012.fcs]
Granulocytes
3987

Monocytes

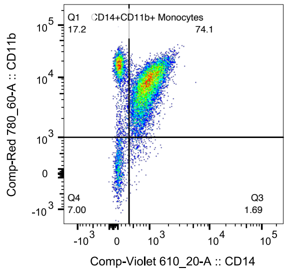

[20180731 Batch2_550_012.fcs]
Monocytes
13210

Lymphocytes

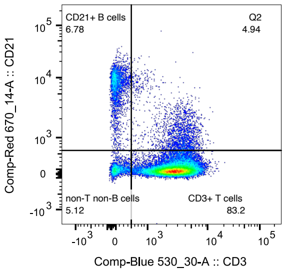

[20180731 Batch2_550_012.fcs]
Lymphocytes
40950

non-T non-B

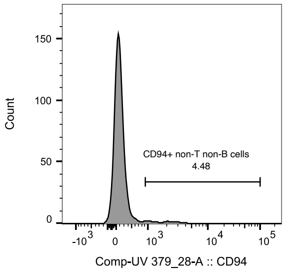

[20180731 Batch2_550_012.fcs]
non-T non-B cells
2096

550
31-JUL-2018

T cells

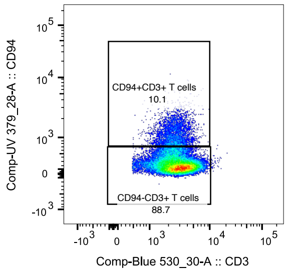

[20180731 Batch2_550_012.fcs]
CD3+ T cells
34054

CD94- T cells

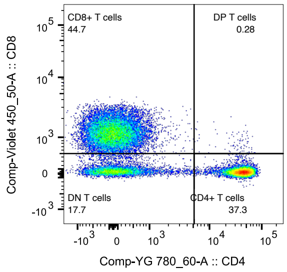

[20180731 Batch2_550_012.fcs]
CD94-CD3+ T cells
30215

CD4 T cells

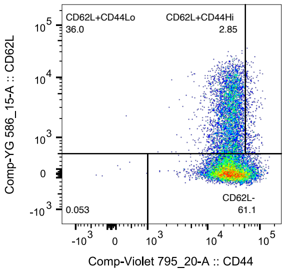

[20180731 Batch2_550_012.fcs]
CD4+ T cells
11262

CD4 T cells

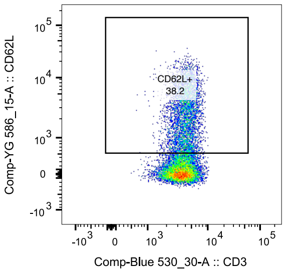

[20180731 Batch2_550_012.fcs]
CD4+ T cells
11262

CD8 T cells

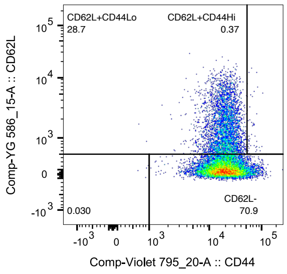

[20180731 Batch2_550_012.fcs]
CD8+ T cells
13507

CD8 T cells

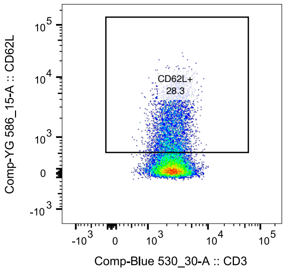

[20180731 Batch2_550_012.fcs]
CD8+ T cells
13507

DN T cells

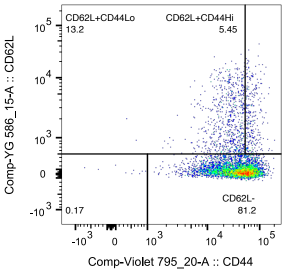

[20180731 Batch2_550_012.fcs]
DN T cells
5362

DN T cells

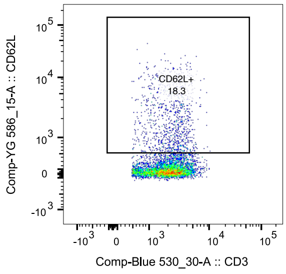

[20180731 Batch2_550_012.fcs]
DN T cells
5362

DP T cells

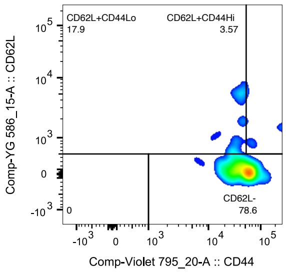

[20180731 Batch2_550_012.fcs]
DP T cells
84.0

DP T cells

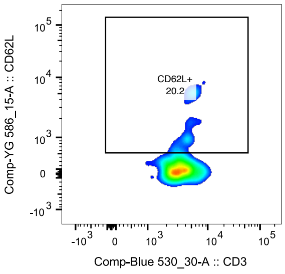

[20180731 Batch2_550_012.fcs]
DP T cells
84.0

609
31-JUL-2018

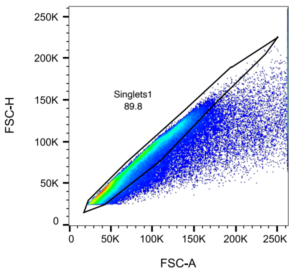

[20180731 Batch2_609_022.fcs]
Ungated
209992

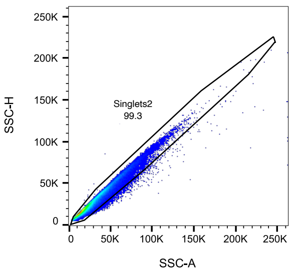

[20180731 Batch2_609_022.fcs]
Singlets1
188613

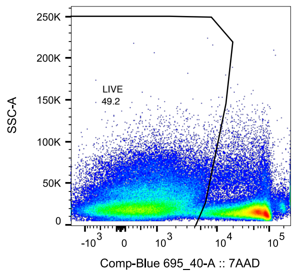

[20180731 Batch2_609_022.fcs]
Singlets2
187287

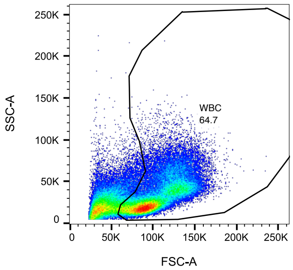

[20180731 Batch2_609_022.fcs]
LIVE
92198

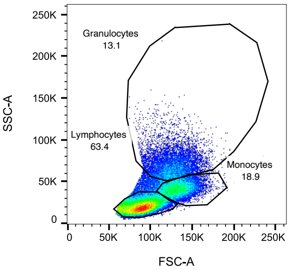

[20180731 Batch2_609_022.fcs]
WBC
59646

Granulocytes

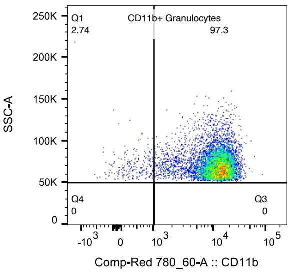

[20180731 Batch2_609_022.fcs]
Granulocytes
7807

Monocytes

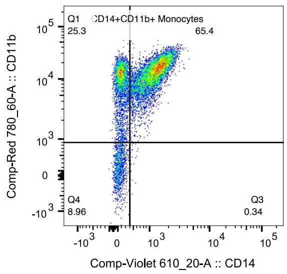

[20180731 Batch2_609_022.fcs]
Monocytes
11277

Lymphocytes

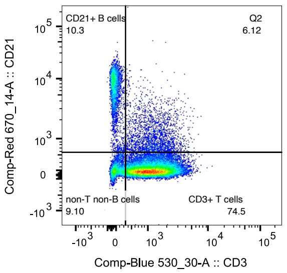

[20180731 Batch2_609_022.fcs]
Lymphocytes
37805

non-T non-B

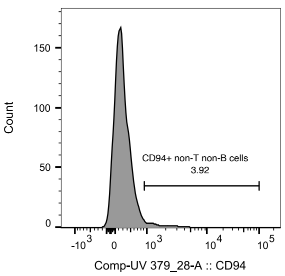

[20180731 Batch2_609_022.fcs]
non-T non-B cells
3442

609
31-JUL-2018

T cells

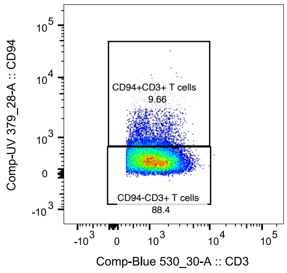

[20180731 Batch2_609_022.fcs]
CD3+ T cells
28158

CD94- T cells

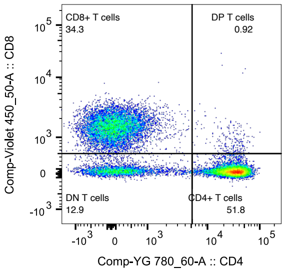

[20180731 Batch2_609_022.fcs]
CD94-CD3+ T cells
24883

CD4 T cells

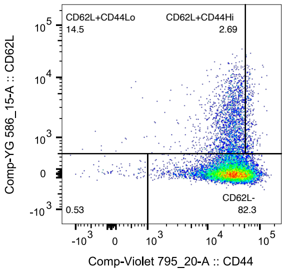

[20180731 Batch2_609_022.fcs]
CD4+ T cells
12897

CD4 T cells

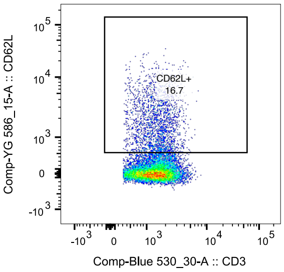

[20180731 Batch2_609_022.fcs]
CD4+ T cells
12897

CD8 T cells

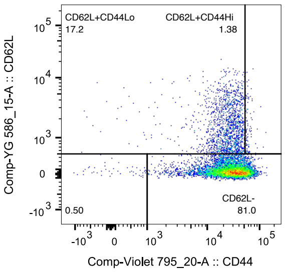

[20180731 Batch2_609_022.fcs]
CD8+ T cells
8541

CD8 T cells

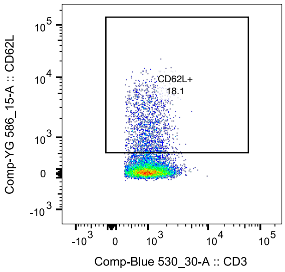

[20180731 Batch2_609_022.fcs]
CD8+ T cells
8541

DN T cells

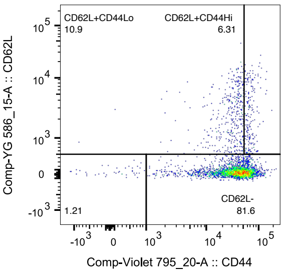

[20180731 Batch2_609_022.fcs]
DN T cells
3216

DN T cells

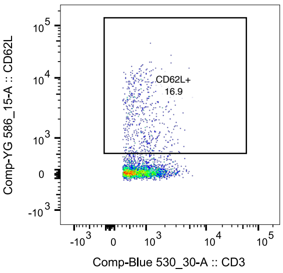

[20180731 Batch2_609_022.fcs]
DN T cells
3216

DP T cells

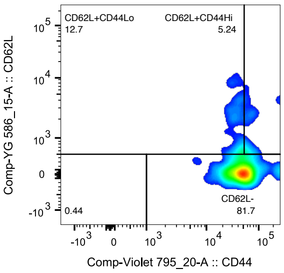

[20180731 Batch2_609_022.fcs]
DP T cells
229

DP T cells

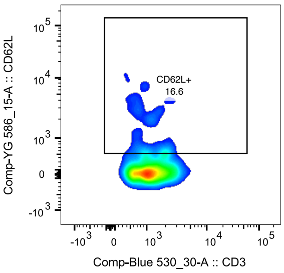

[20180731 Batch2_609_022.fcs]
DP T cells
229

591
31-JUL-2018

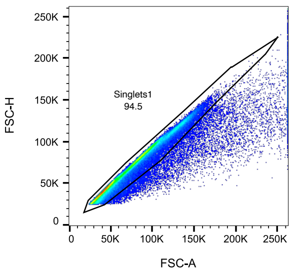

[20180731 Batch2_591_020.fcs]
Ungated
151204

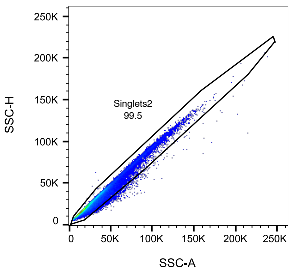

[20180731 Batch2_591_020.fcs]
Singlets1
142885

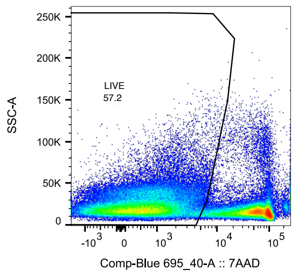

[20180731 Batch2_591_020.fcs]
Singlets2
142149

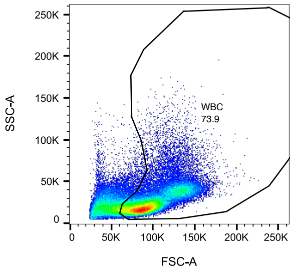

[20180731 Batch2_591_020.fcs]
LIVE
81350

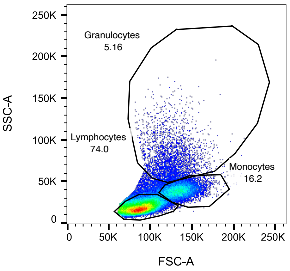

[20180731 Batch2_591_020.fcs]
WBC
60100

Granulocytes

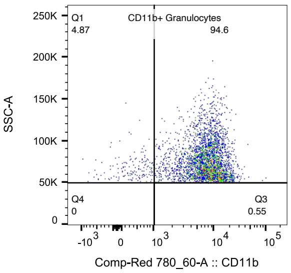

[20180731 Batch2_591_020.fcs]
Granulocytes
3102

Monocytes

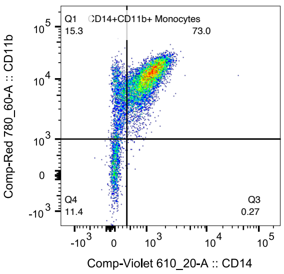

[20180731 Batch2_591_020.fcs]
Monocytes
9752

Lymphocytes

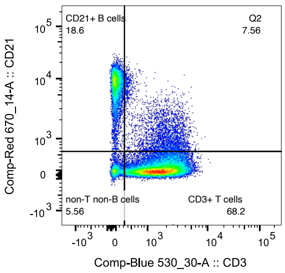

[20180731 Batch2_591_020.fcs]
Lymphocytes
44445

non-T non-B

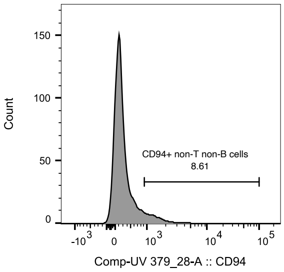

[20180731 Batch2_591_020.fcs]
non-T non-B cells
2473

591
31-JUL-2018

T cells

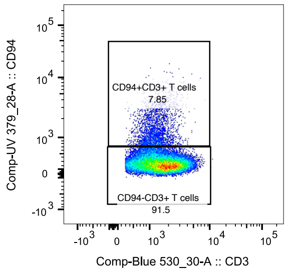

[20180731 Batch2_591_020.fcs]
CD3+ T cells
30330

CD94- T cells

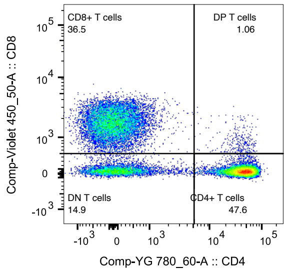

[20180731 Batch2_591_020.fcs]
CD94-CD3+ T cells
27755

CD4 T cells

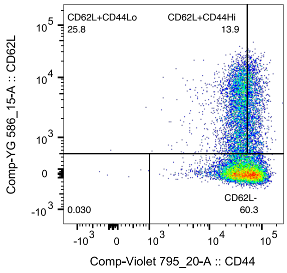

[20180731 Batch2_591_020.fcs]
CD4+ T cells
13212

CD4 T cells

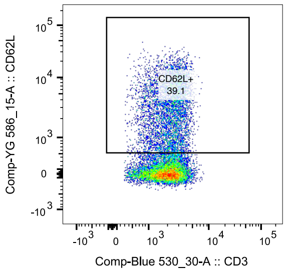

[20180731 Batch2_591_020.fcs]
CD4+ T cells
13212

CD8 T cells

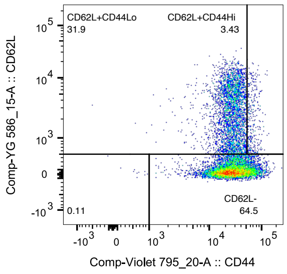

[20180731 Batch2_591_020.fcs]
CD8+ T cells
10117

CD8 T cells

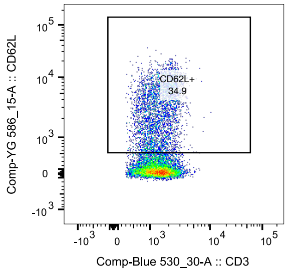

[20180731 Batch2_591_020.fcs]
CD8+ T cells
10117

DN T cells

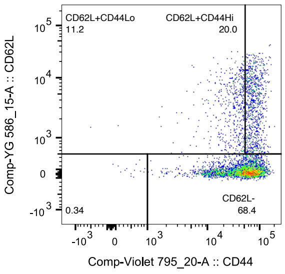

[20180731 Batch2_591_020.fcs]
DN T cells
4133

DN T cells

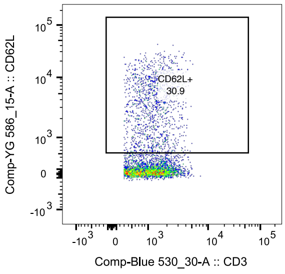

[20180731 Batch2_591_020.fcs]
DN T cells
4133

DP T cells

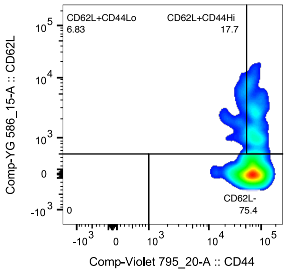

[20180731 Batch2_591_020.fcs]
DP T cells
293

DP T cells

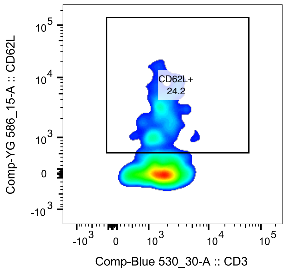

[20180731 Batch2_591_020.fcs]
DP T cells
293

522
31-JUL-2018

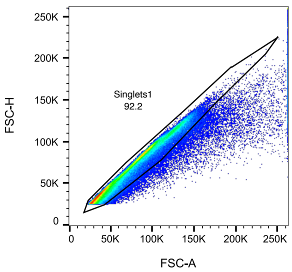

[20180731 Batch2_522_007.fcs]
Ungated
134070

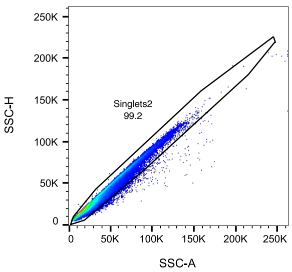

[20180731 Batch2_522_007.fcs]
Singlets1
123554

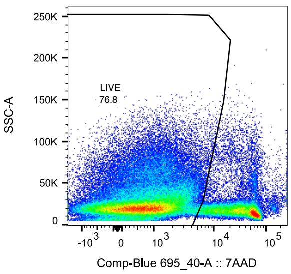

[20180731 Batch2_522_007.fcs]
Singlets2
122532

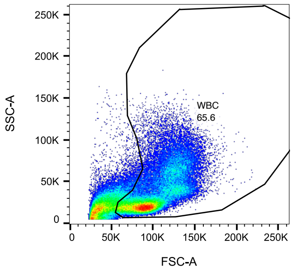

[20180731 Batch2_522_007.fcs]
LIVE
94059

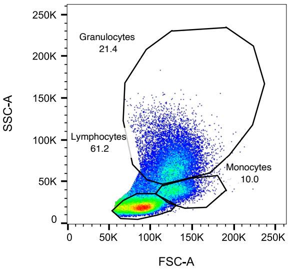

[20180731 Batch2_522_007.fcs]
WBC
61743

Granulocytes

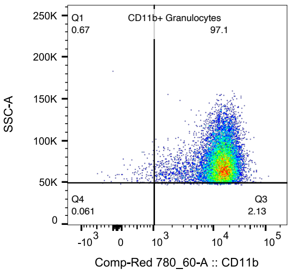

[20180731 Batch2_522_007.fcs]
Granulocytes
13185

Monocytes

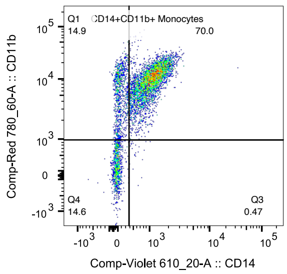

[20180731 Batch2_522_007.fcs]
Monocytes
6190

Lymphocytes

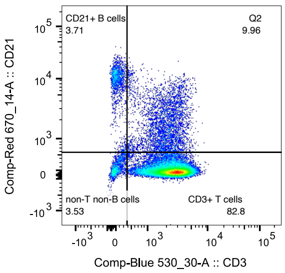

[20180731 Batch2_522_007.fcs]
Lymphocytes
37785

non-T non-B

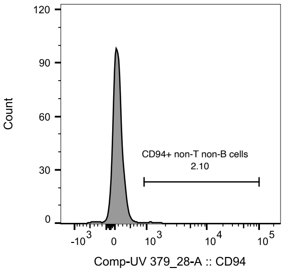

[20180731 Batch2_522_007.fcs]
non-T non-B cells
1334

522
31-JUL-2018

T cells

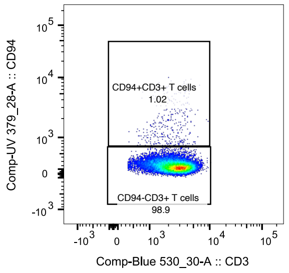

[20180731 Batch2_522_007.fcs]
CD3+ T cells
31284

CD94- T cells

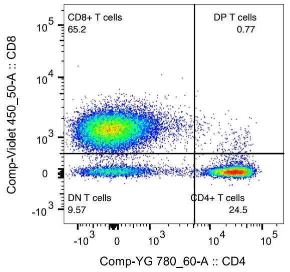

[20180731 Batch2_522_007.fcs]
CD94-CD3+ T cells
30940

CD4 T cells

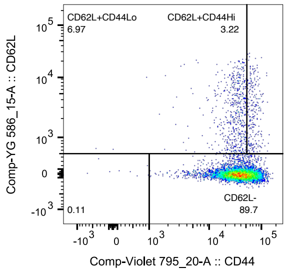

[20180731 Batch2_522_007.fcs]
CD4+ T cells
7573

CD4 T cells

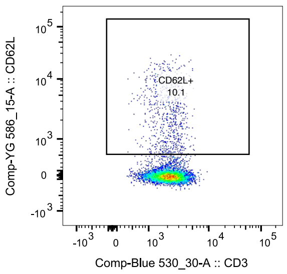

[20180731 Batch2_522_007.fcs]
CD4+ T cells
7573

CD8 T cells

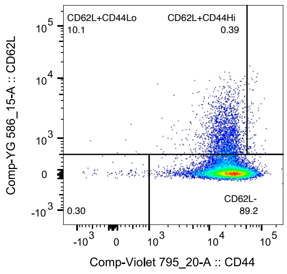

[20180731 Batch2_522_007.fcs]
CD8+ T cells
20170

CD8 T cells

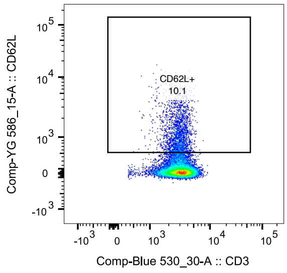

[20180731 Batch2_522_007.fcs]
CD8+ T cells
20170

DN T cells

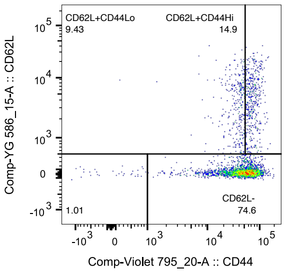

[20180731 Batch2_522_007.fcs]
DN T cells
2960

DN T cells

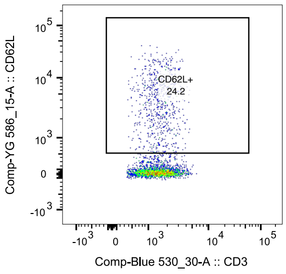

[20180731 Batch2_522_007.fcs]
DN T cells
2960

DP T cells

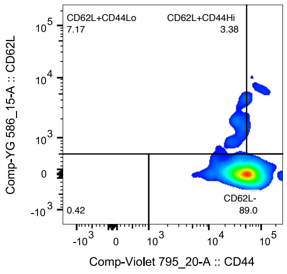

[20180731 Batch2_522_007.fcs]
DP T cells
237

DP T cells

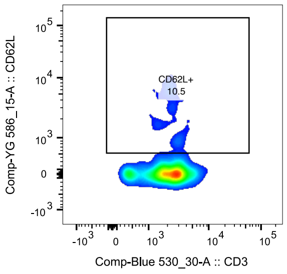

[20180731 Batch2_522_007.fcs]
DP T cells
237

502
01-AUG-2018

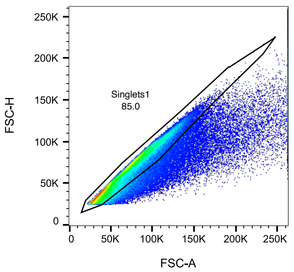

[20180801 Batch3_502_001.fcs]
Ungated
179975

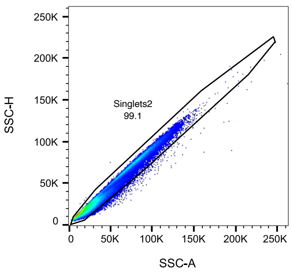

[20180801 Batch3_502_001.fcs]
Singlets1
152946

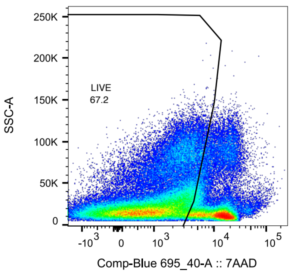

[20180801 Batch3_502_001.fcs]
Singlets2
151640

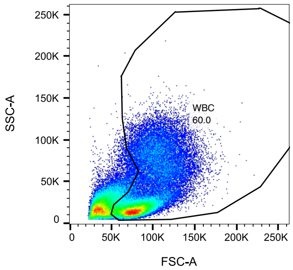

[20180801 Batch3_502_001.fcs]
LIVE
101967

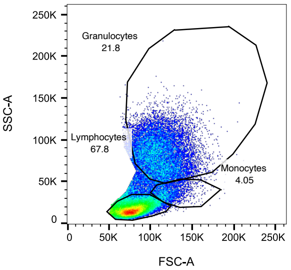

[20180801 Batch3_502_001.fcs]
WBC
61211

Granulocytes

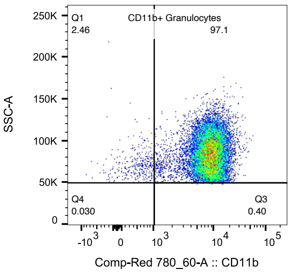

[20180801 Batch3_502_001.fcs]
Granulocytes
13345

Monocytes

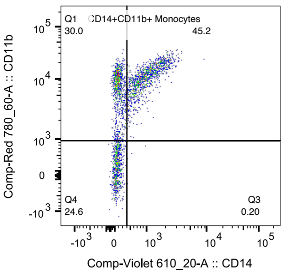

[20180801 Batch3_502_001.fcs]
Monocytes
2481

Lymphocytes

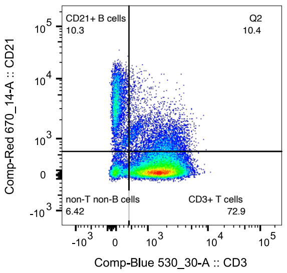

[20180801 Batch3_502_001.fcs]
Lymphocytes
41475

non-T non-B

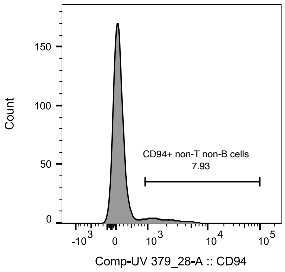

[20180801 Batch3_502_001.fcs]
non-T non-B cells
2662

502
01-AUG-2018

T cells

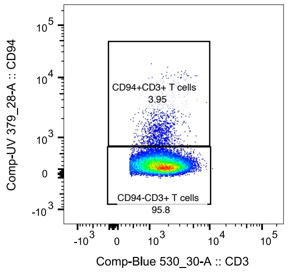

[20180801 Batch3_502_001.fcs]
CD3+ T cells
30228

CD94- T cells

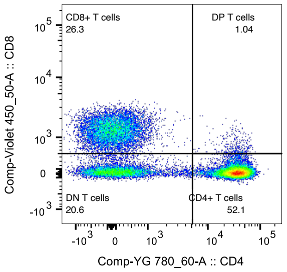

[20180801 Batch3_502_001.fcs]
CD94-CD3+ T cells
28964

CD4 T cells

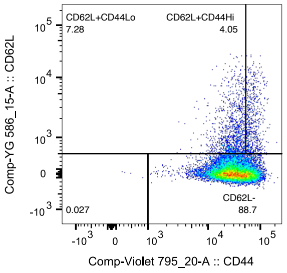

[20180801 Batch3_502_001.fcs]
CD4+ T cells
15076

CD4 T cells

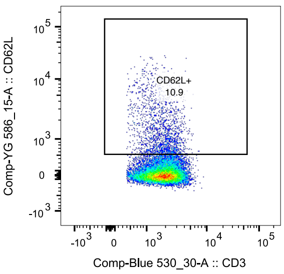

[20180801 Batch3_502_001.fcs]
CD4+ T cells
15076

CD8 T cells

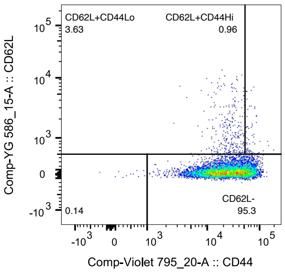

[20180801 Batch3_502_001.fcs]
CD8+ T cells
7613

CD8 T cells

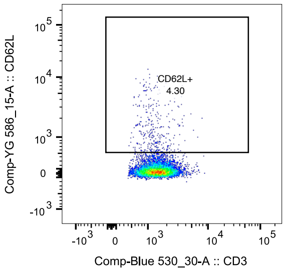

[20180801 Batch3_502_001.fcs]
CD8+ T cells
7613

DN T cells

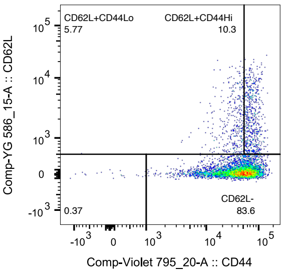

[20180801 Batch3_502_001.fcs]
DN T cells
5975

DN T cells

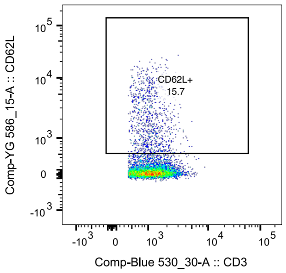

[20180801 Batch3_502_001.fcs]
DN T cells
5975

DP T cells

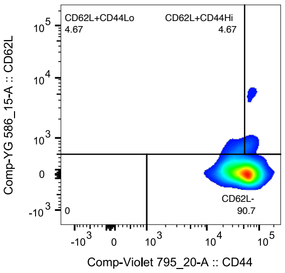

[20180801 Batch3_502_001.fcs]
DP T cells
300

DP T cells

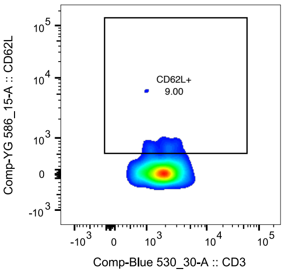

[20180801 Batch3_502_001.fcs]
DP T cells
300

503
01-AUG-2018

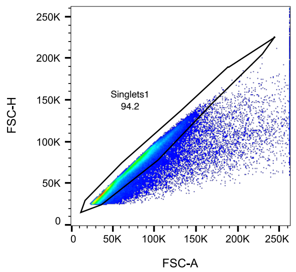

[20180801 Batch3_503_002.fcs]
Ungated
216524

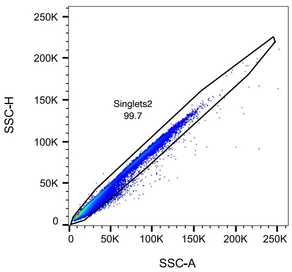

[20180801 Batch3_503_002.fcs]
Singlets1
203890

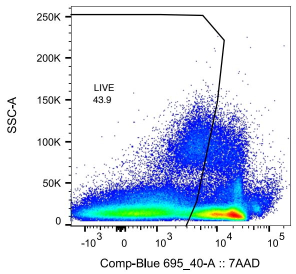

[20180801 Batch3_503_002.fcs]
Singlets2
203307

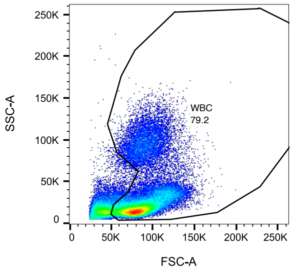

[20180801 Batch3_503_002.fcs]
LIVE
89174

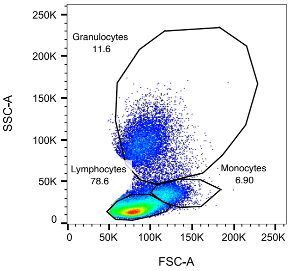

[20180801 Batch3_503_002.fcs]
WBC
70619

Granulocytes

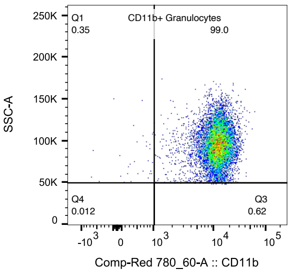

[20180801 Batch3_503_002.fcs]
Granulocytes
8203

Monocytes

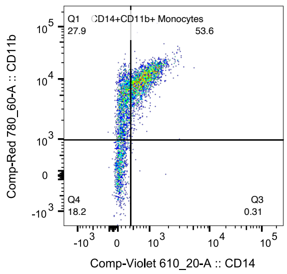

[20180801 Batch3_503_002.fcs]
Monocytes
4876

Lymphocytes

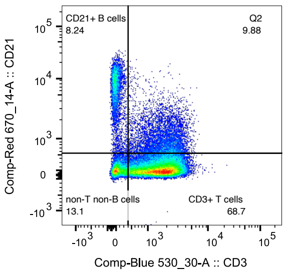

[20180801 Batch3_503_002.fcs]
Lymphocytes
55485

non-T non-B

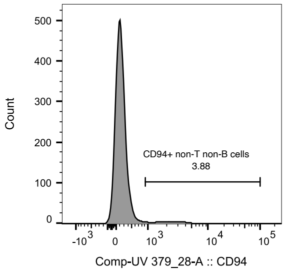

[20180801 Batch3_503_002.fcs]
non-T non-B cells
7285

503
01-AUG-2018

T cells

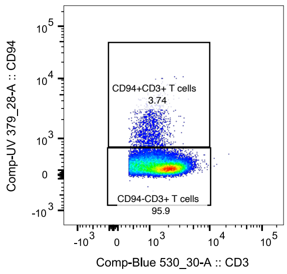

[20180801 Batch3_503_002.fcs]
CD3+ T cells
38144

CD94- T cells

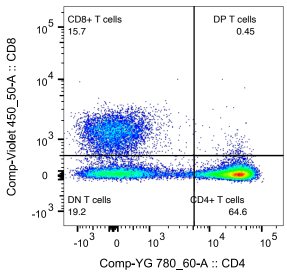

[20180801 Batch3_503_002.fcs]
CD94-CD3+ T cells
36595

CD4 T cells

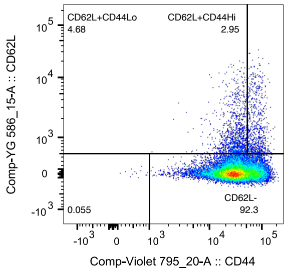

[20180801 Batch3_503_002.fcs]
CD4+ T cells
23655

CD4 T cells

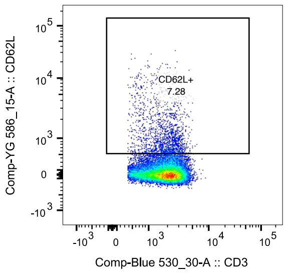

[20180801 Batch3_503_002.fcs]
CD4+ T cells
23655

CD8 T cells

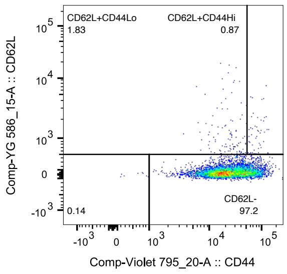

[20180801 Batch3_503_002.fcs]
CD8+ T cells
5746

CD8 T cells

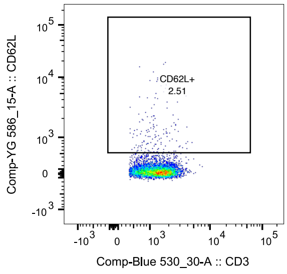

[20180801 Batch3_503_002.fcs]
CD8+ T cells
5746

DN T cells

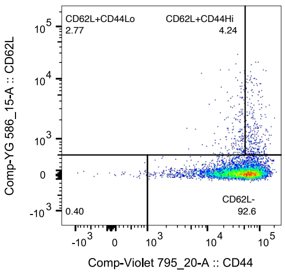

[20180801 Batch3_503_002.fcs]
DN T cells
7029

DN T cells

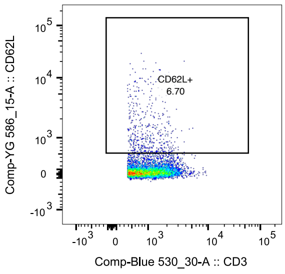

[20180801 Batch3_503_002.fcs]
DN T cells
7029

DP T cells

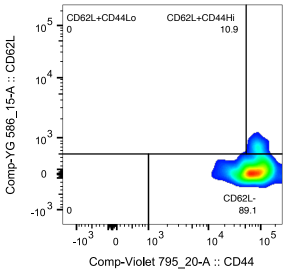

[20180801 Batch3_503_002.fcs]
DP T cells
165

DP T cells

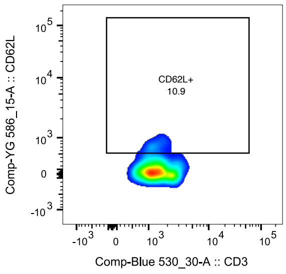

[20180801 Batch3_503_002.fcs]
DP T cells
165

510
01-AUG-2018

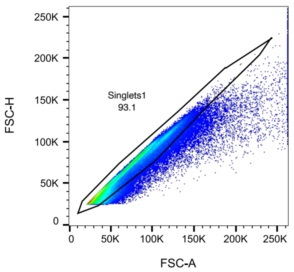

[20180801 Batch3_510_003.fcs]
Ungated
249242

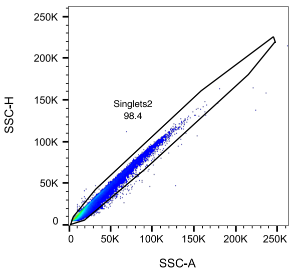

[20180801 Batch3_510_003.fcs]
Singlets1
231974

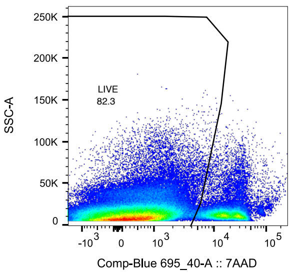

[20180801 Batch3_510_003.fcs]
Singlets2
228215

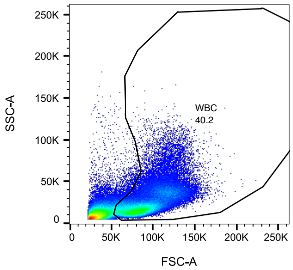

[20180801 Batch3_510_003.fcs]
LIVE
187822

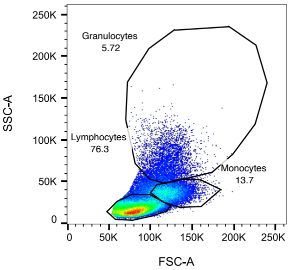

[20180801 Batch3_510_003.fcs]
WBC
75568

Granulocytes

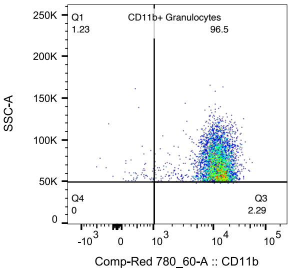

[20180801 Batch3_510_003.fcs]
Granulocytes
4324

Monocytes

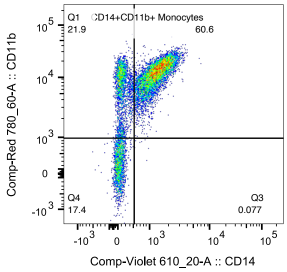

[20180801 Batch3_510_003.fcs]
Monocytes
10379

Lymphocytes

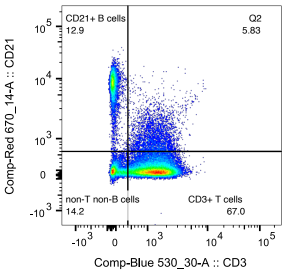

[20180801 Batch3_510_003.fcs]
Lymphocytes
57631

non-T non-B

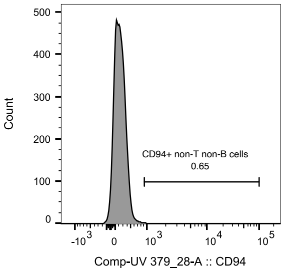

[20180801 Batch3_510_003.fcs]
non-T non-B cells
8193

510
01-AUG-2018

T cells

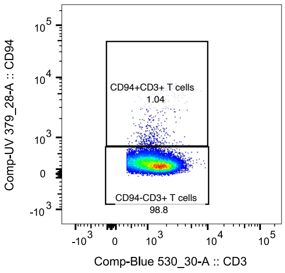

[20180801 Batch3_510_003.fcs]
CD3+ T cells
38634

CD94- T cells

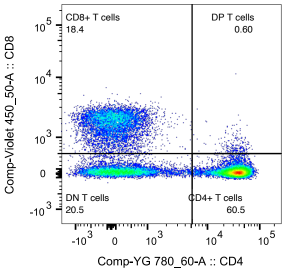

[20180801 Batch3_510_003.fcs]
CD94-CD3+ T cells
38173

CD4 T cells

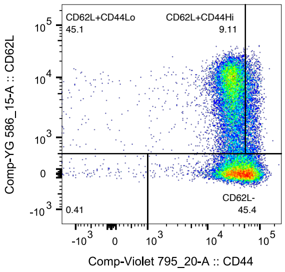

[20180801 Batch3_510_003.fcs]
CD4+ T cells
23094

CD4 T cells

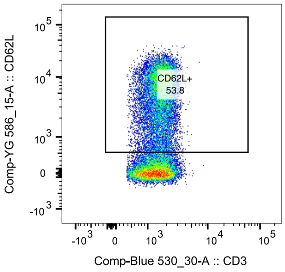

[20180801 Batch3_510_003.fcs]
CD4+ T cells
23094

CD8 T cells

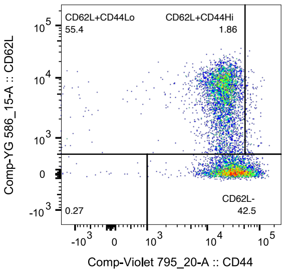

[20180801 Batch3_510_003.fcs]
CD8+ T cells
7042

CD8 T cells

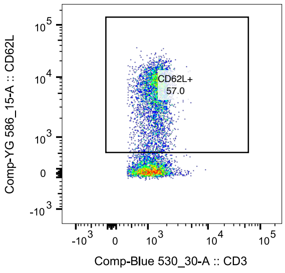

[20180801 Batch3_510_003.fcs]
CD8+ T cells
7042

DN T cells

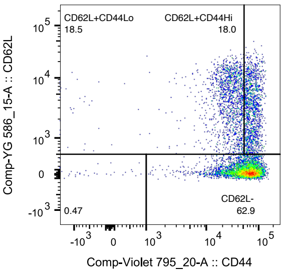

[20180801 Batch3_510_003.fcs]
DN T cells
7807

DN T cells

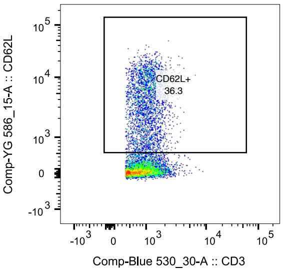

[20180801 Batch3_510_003.fcs]
DN T cells
7807

DP T cells

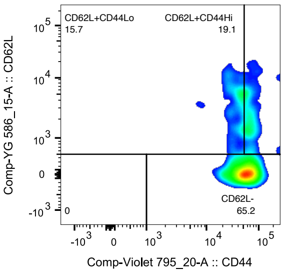

[20180801 Batch3_510_003.fcs]
DP T cells
230

DP T cells

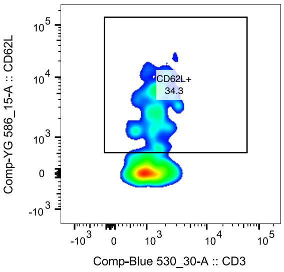

[20180801 Batch3_510_003.fcs]
DP T cells
230

519
01-AUG-2018

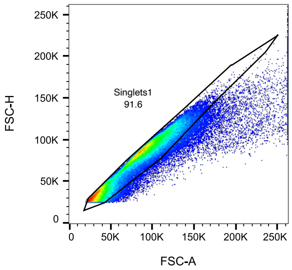

[20180801 Batch3_519_004.fcs]
Ungated
127319

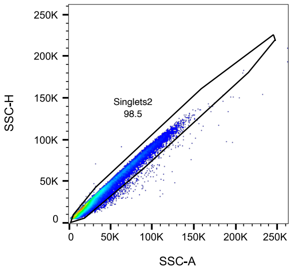

[20180801 Batch3_519_004.fcs]
Singlets1
116604

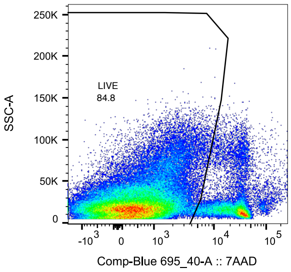

[20180801 Batch3_519_004.fcs]
Singlets2
114801

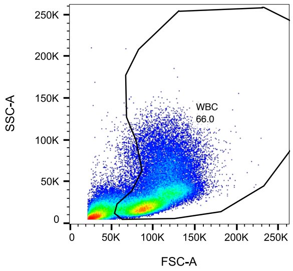

[20180801 Batch3_519_004.fcs]
LIVE
97389

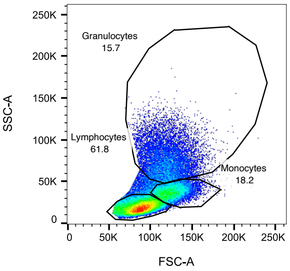

[20180801 Batch3_519_004.fcs]
WBC
64279

Granulocytes

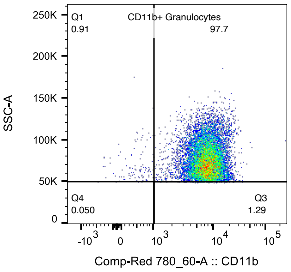

[20180801 Batch3_519_004.fcs]
Granulocytes
10082

Monocytes

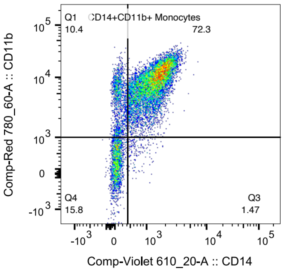

[20180801 Batch3_519_004.fcs]
Monocytes
11682

Lymphocytes

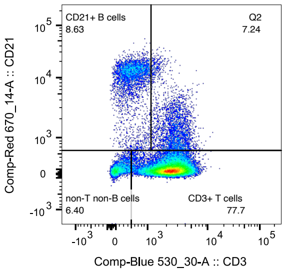

[20180801 Batch3_519_004.fcs]
Lymphocytes
39731

non-T non-B

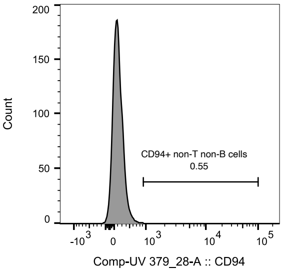

[20180801 Batch3_519_004.fcs]
non-T non-B cells
2541

519
01-AUG-2018

T cells

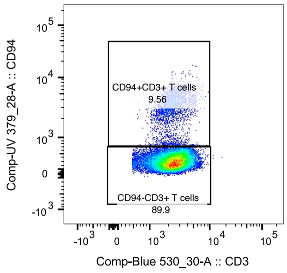

[20180801 Batch3_519_004.fcs]
CD3+ T cells
30884

CD94- T cells

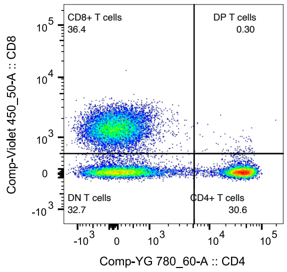

[20180801 Batch3_519_004.fcs]
CD94-CD3+ T cells
27758

CD4 T cells

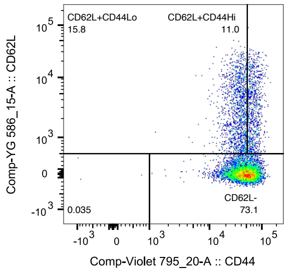

[20180801 Batch3_519_004.fcs]
CD4+ T cells
8501

CD4 T cells

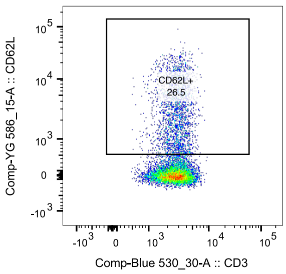

[20180801 Batch3_519_004.fcs]
CD4+ T cells
8501

CD8 T cells

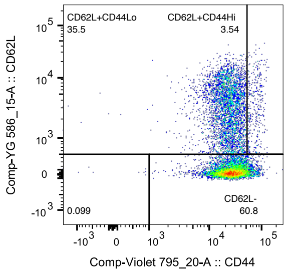

[20180801 Batch3_519_004.fcs]
CD8+ T cells
10103

CD8 T cells

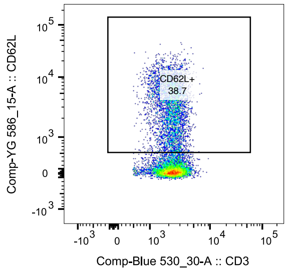

[20180801 Batch3_519_004.fcs]
CD8+ T cells
10103

DN T cells

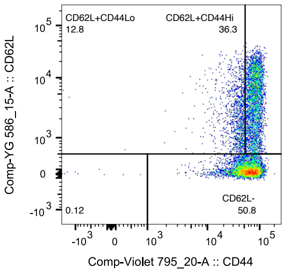

[20180801 Batch3_519_004.fcs]
DN T cells
9070

DN T cells

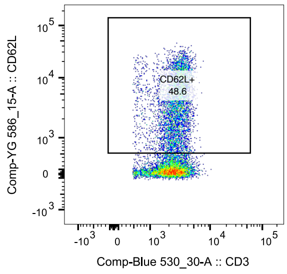

[20180801 Batch3_519_004.fcs]
DN T cells
9070

DP T cells

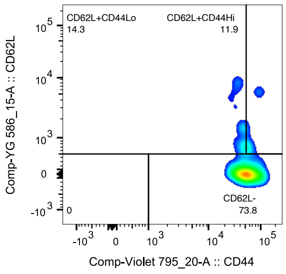

[20180801 Batch3_519_004.fcs]
DP T cells
84.0

DP T cells

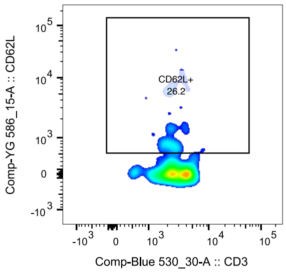

[20180801 Batch3_519_004.fcs]
DP T cells
84.0

521
01-AUG-2018

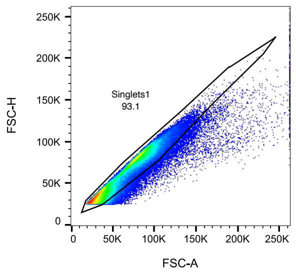

[20180801 Batch3_521_005.fcs]
Ungated
158370

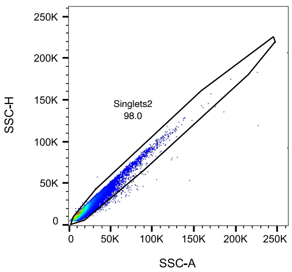

[20180801 Batch3_521_005.fcs]
Singlets1
147521

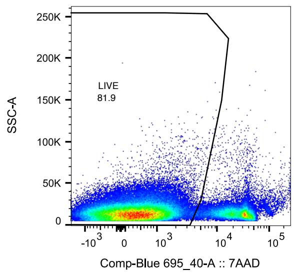

[20180801 Batch3_521_005.fcs]
Singlets2
144622

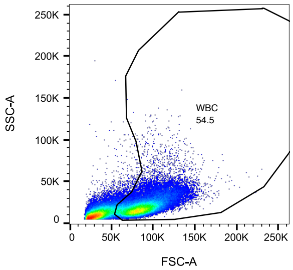

[20180801 Batch3_521_005.fcs]
LIVE
118490

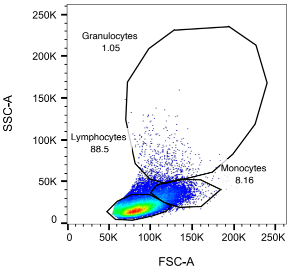

[20180801 Batch3_521_005.fcs]
WBC
64600

Granulocytes

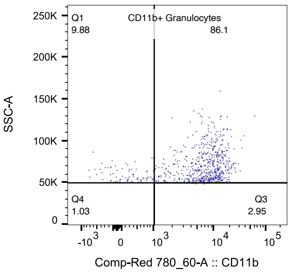

[20180801 Batch3_521_005.fcs]
Granulocytes
678

Monocytes

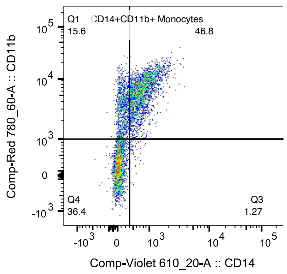

[20180801 Batch3_521_005.fcs]
Monocytes
5272

Lymphocytes

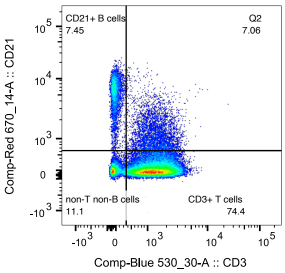

[20180801 Batch3_521_005.fcs]
Lymphocytes
57200

non-T non-B

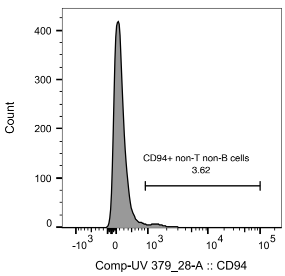

[20180801 Batch3_521_005.fcs]
non-T non-B cells
6349

521
01-AUG-2018

T cells

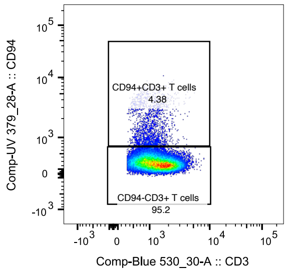

[20180801 Batch3_521_005.fcs]
CD3+ T cells
42546

CD94- T cells

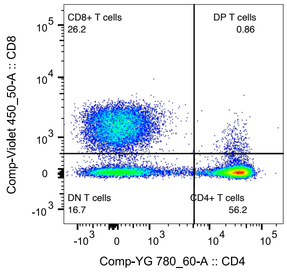

[20180801 Batch3_521_005.fcs]
CD94-CD3+ T cells
40490

CD4 T cells

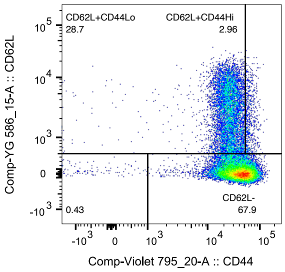

[20180801 Batch3_521_005.fcs]
CD4+ T cells
22771

CD4 T cells

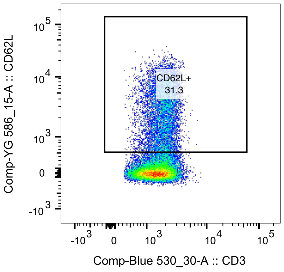

[20180801 Batch3_521_005.fcs]
CD4+ T cells
22771

CD8 T cells

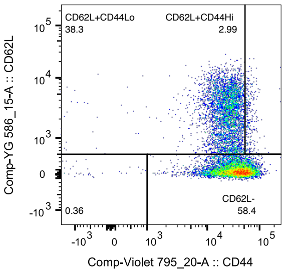

[20180801 Batch3_521_005.fcs]
CD8+ T cells
10616

CD8 T cells

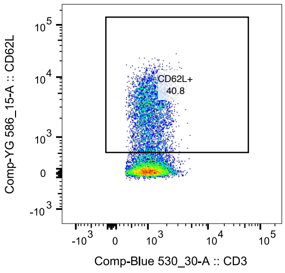

[20180801 Batch3_521_005.fcs]
CD8+ T cells
10616

DN T cells

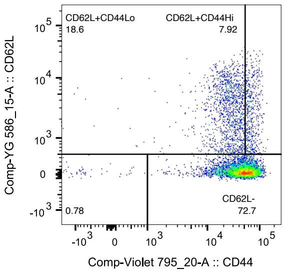

[20180801 Batch3_521_005.fcs]
DN T cells
6756

DN T cells

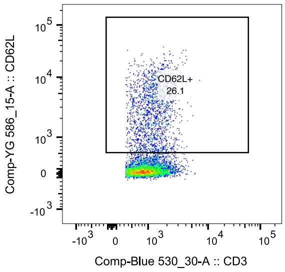

[20180801 Batch3_521_005.fcs]
DN T cells
6756

DP T cells

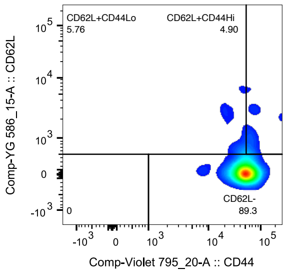

[20180801 Batch3_521_005.fcs]
DP T cells
347

DP T cells

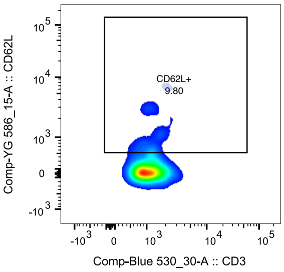

[20180801 Batch3_521_005.fcs]
DP T cells
347

526
01-AUG-2018

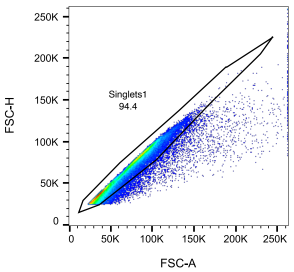

[20180801 Batch3_526_006.fcs]
Ungated
72941

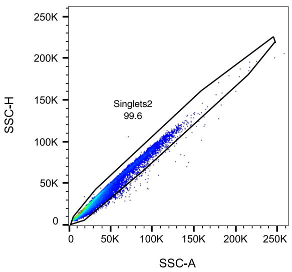

[20180801 Batch3_526_006.fcs]
Singlets1
68846

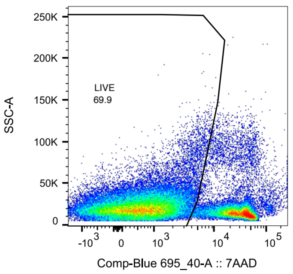

[20180801 Batch3_526_006.fcs]
Singlets2
68575

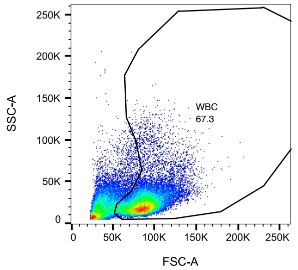

[20180801 Batch3_526_006.fcs]
LIVE
47952

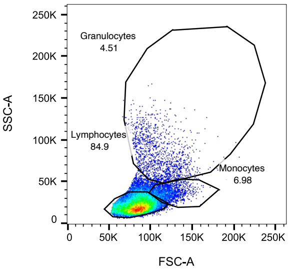

[20180801 Batch3_526_006.fcs]
WBC
32250

Granulocytes

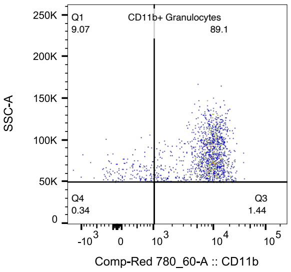

[20180801 Batch3_526_006.fcs]
Granulocytes
1455

Monocytes

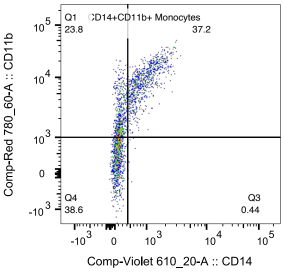

[20180801 Batch3_526_006.fcs]
Monocytes
2252

Lymphocytes

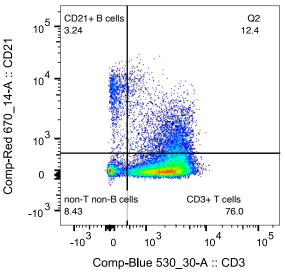

[20180801 Batch3_526_006.fcs]
Lymphocytes
27393

non-T non-B

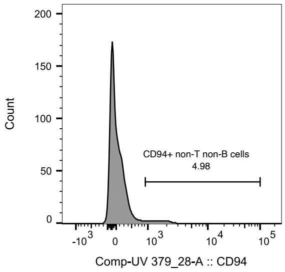

[20180801 Batch3_526_006.fcs]
non-T non-B cells
2310

526
01-AUG-2018

T cells

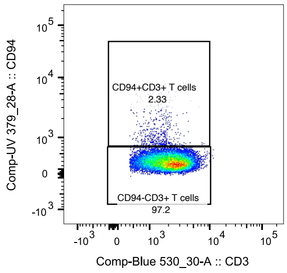

[20180801 Batch3_526_006.fcs]
CD3+ T cells
20809

CD94- T cells

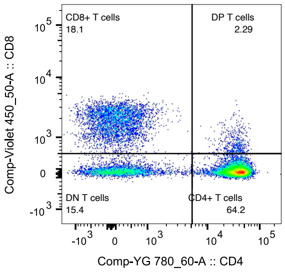

[20180801 Batch3_526_006.fcs]
CD94-CD3+ T cells
20236

CD4 T cells

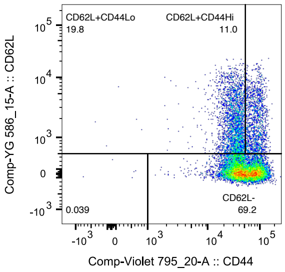

[20180801 Batch3_526_006.fcs]
CD4+ T cells
12986

CD4 T cells

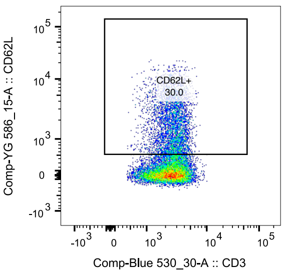

[20180801 Batch3_526_006.fcs]
CD4+ T cells
12986

CD8 T cells

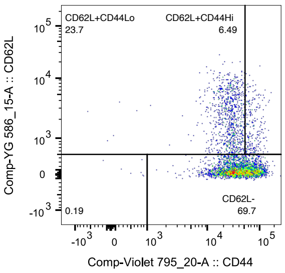

[20180801 Batch3_526_006.fcs]
CD8+ T cells
3668

CD8 T cells

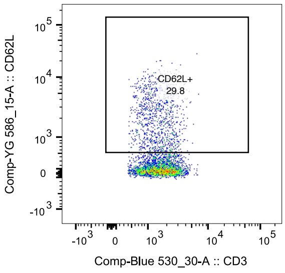

[20180801 Batch3_526_006.fcs]
CD8+ T cells
3668

DN T cells

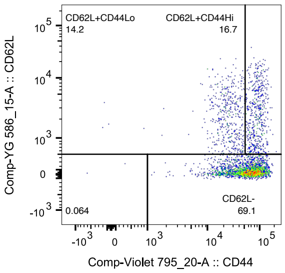

[20180801 Batch3_526_006.fcs]
DN T cells
3118

DN T cells

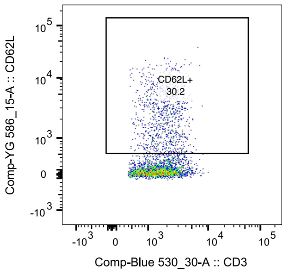

[20180801 Batch3_526_006.fcs]
DN T cells
3118

DP T cells

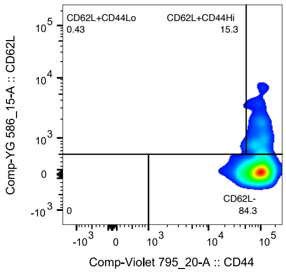

[20180801 Batch3_526_006.fcs]
DP T cells
464

DP T cells

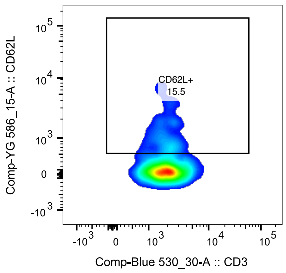

[20180801 Batch3_526_006.fcs]
DP T cells
464

532
01-AUG-2018

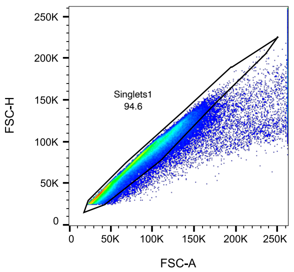

[20180801 Batch3_532_007.fcs]
Ungated
182537

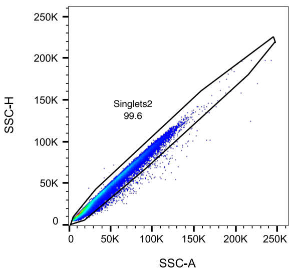

[20180801 Batch3_532_007.fcs]
Singlets1
172673

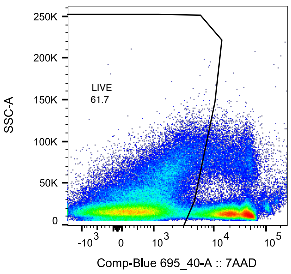

[20180801 Batch3_532_007.fcs]
Singlets2
171995

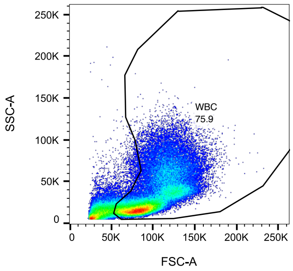

[20180801 Batch3_532_007.fcs]
LIVE
106035

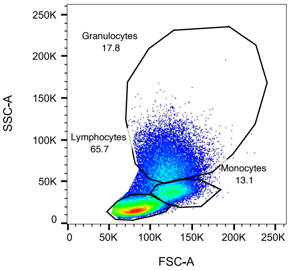

[20180801 Batch3_532_007.fcs]
WBC
80481

Granulocytes

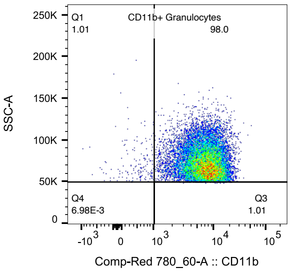

[20180801 Batch3_532_007.fcs]
Granulocytes
14324

Monocytes

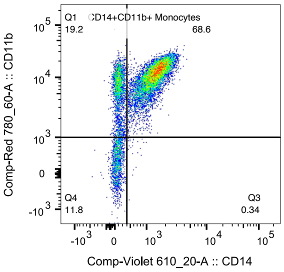

[20180801 Batch3_532_007.fcs]
Monocytes
10509

Lymphocytes

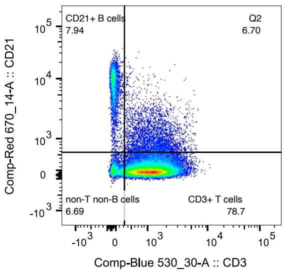

[20180801 Batch3_532_007.fcs]
Lymphocytes
52892

non-T non-B

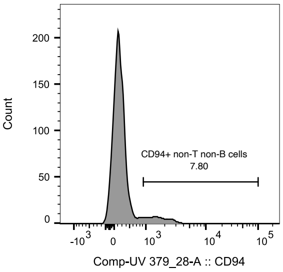

[20180801 Batch3_532_007.fcs]
non-T non-B cells
3538

532
01-AUG-2018

T cells

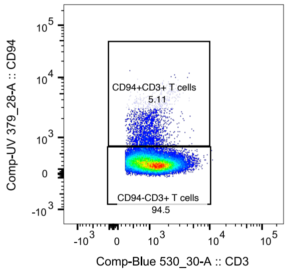

[20180801 Batch3_532_007.fcs]
CD3+ T cells
41609

CD94- T cells

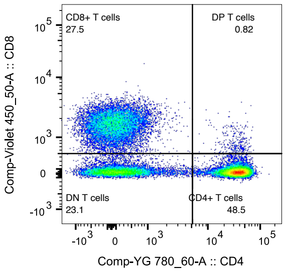

[20180801 Batch3_532_007.fcs]
CD94-CD3+ T cells
39314

CD4 T cells

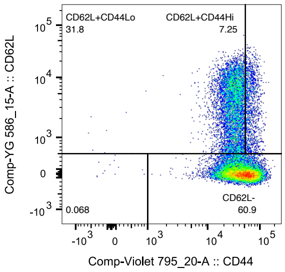

[20180801 Batch3_532_007.fcs]
CD4+ T cells
19082

CD4 T cells

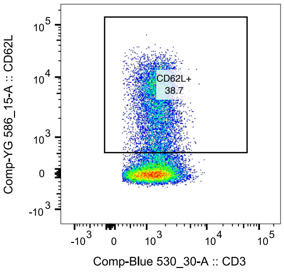

[20180801 Batch3_532_007.fcs]
CD4+ T cells
19082

CD8 T cells

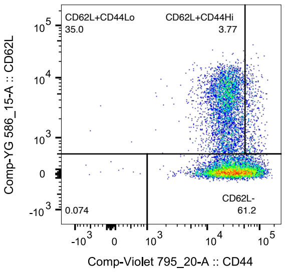

[20180801 Batch3_532_007.fcs]
CD8+ T cells
10808

CD8 T cells

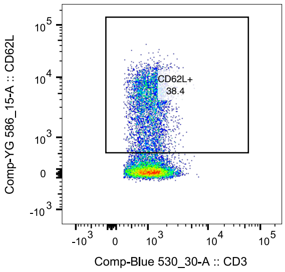

[20180801 Batch3_532_007.fcs]
CD8+ T cells
10808

DN T cells

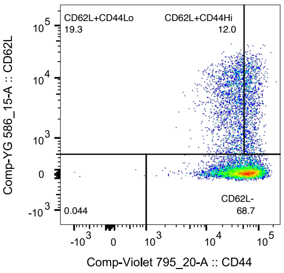

[20180801 Batch3_532_007.fcs]
DN T cells
9100

DN T cells

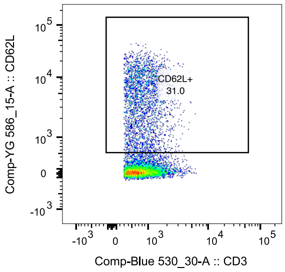

[20180801 Batch3_532_007.fcs]
DN T cells
9100

DP T cells

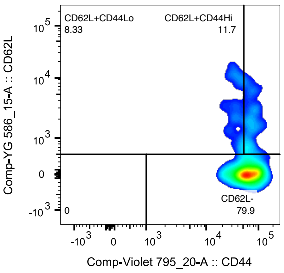

[20180801 Batch3_532_007.fcs]
DP T cells
324

DP T cells

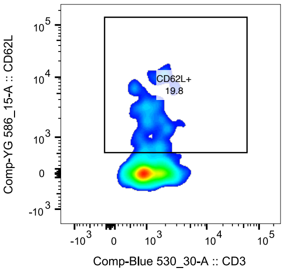

[20180801 Batch3_532_007.fcs]
DP T cells
324

533
01-AUG-2018

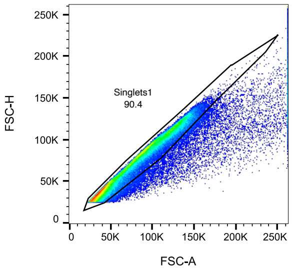

[20180801 Batch3_533_008.fcs]
Ungated
110938

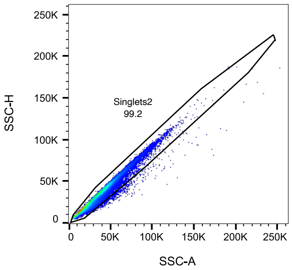

[20180801 Batch3_533_008.fcs]
Singlets1
100259

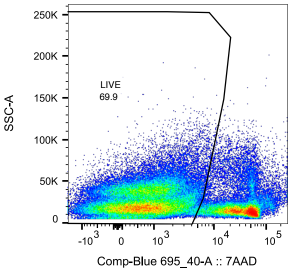

[20180801 Batch3_533_008.fcs]
Singlets2
99477

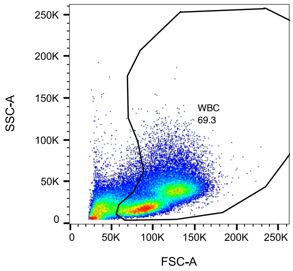

[20180801 Batch3_533_008.fcs]
LIVE
69548

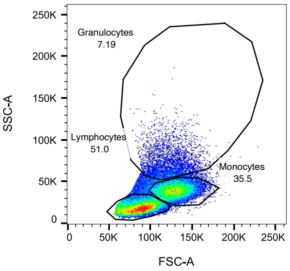

[20180801 Batch3_533_008.fcs]
WBC
48162

Granulocytes

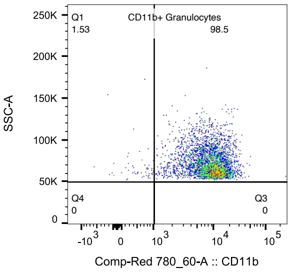

[20180801 Batch3_533_008.fcs]
Granulocytes
3464

Monocytes

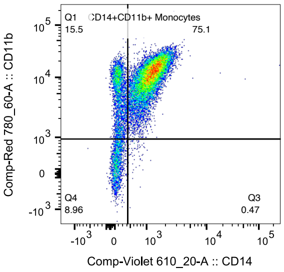

[20180801 Batch3_533_008.fcs]
Monocytes
17096

Lymphocytes

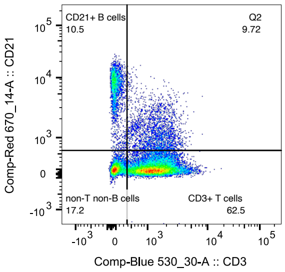

[20180801 Batch3_533_008.fcs]
Lymphocytes
24552

non-T non-B

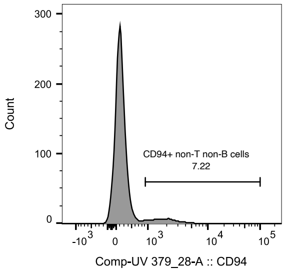

[20180801 Batch3_533_008.fcs]
non-T non-B cells
4227

533
01-AUG-2018

T cells

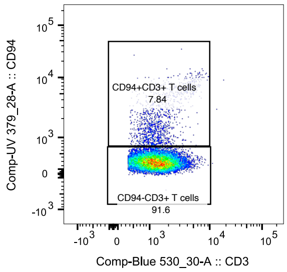

[20180801 Batch3_533_008.fcs]
CD3+ T cells
15351

CD94- T cells

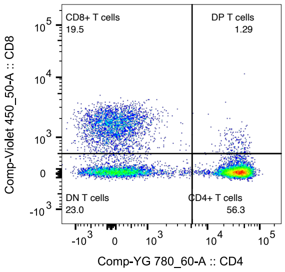

[20180801 Batch3_533_008.fcs]
CD94-CD3+ T cells
14060

CD4 T cells

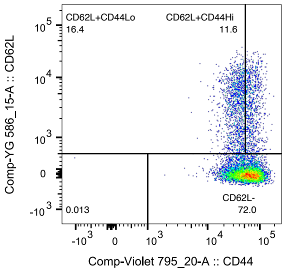

[20180801 Batch3_533_008.fcs]
CD4+ T cells
7913

CD4 T cells

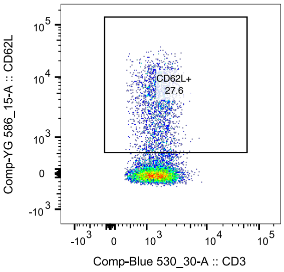

[20180801 Batch3_533_008.fcs]
CD4+ T cells
7913

CD8 T cells

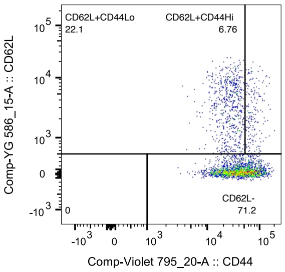

[20180801 Batch3_533_008.fcs]
CD8+ T cells
2735

CD8 T cells

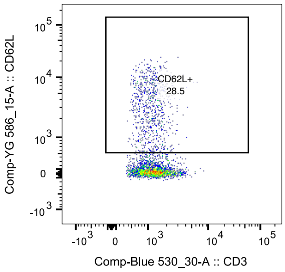

[20180801 Batch3_533_008.fcs]
CD8+ T cells
2735

DN T cells

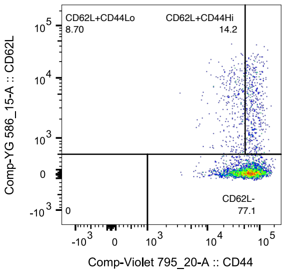

[20180801 Batch3_533_008.fcs]
DN T cells
3230

DN T cells

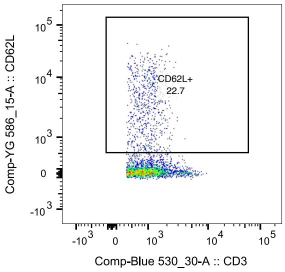

[20180801 Batch3_533_008.fcs]
DN T cells
3230

DP T cells

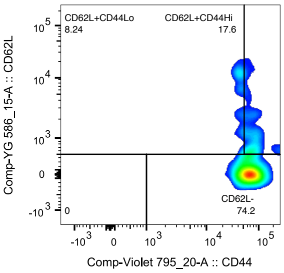

[20180801 Batch3_533_008.fcs]
DP T cells
182

DP T cells

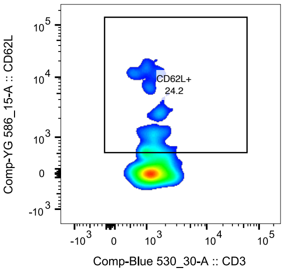

[20180801 Batch3_533_008.fcs]
DP T cells
182

552
01-AUG-2018

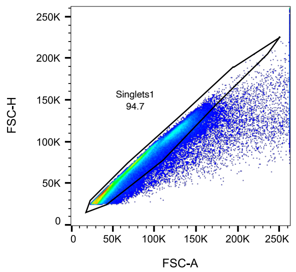

[20180801 Batch3_552_009.fcs]
Ungated
225978

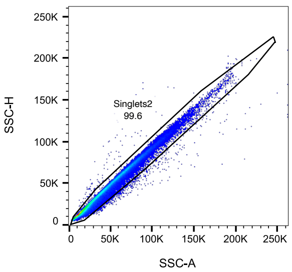

[20180801 Batch3_552_009.fcs]
Singlets1
213905

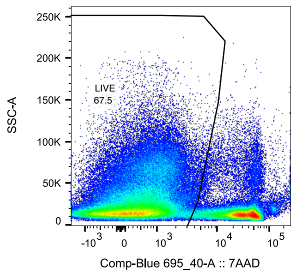

[20180801 Batch3_552_009.fcs]
Singlets2
213049

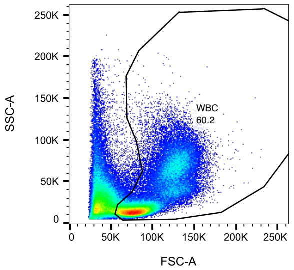

[20180801 Batch3_552_009.fcs]
LIVE
143795

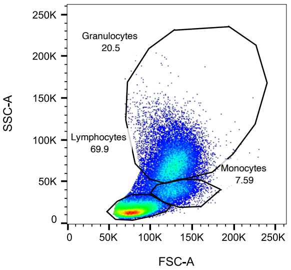

[20180801 Batch3_552_009.fcs]
WBC
86564

Granulocytes

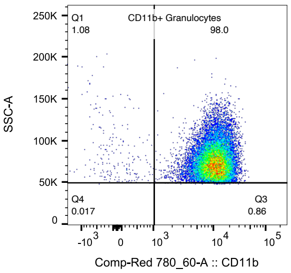

[20180801 Batch3_552_009.fcs]
Granulocytes
17744

Monocytes

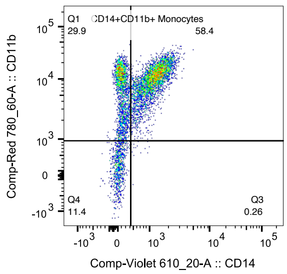

[20180801 Batch3_552_009.fcs]
Monocytes
6572

Lymphocytes

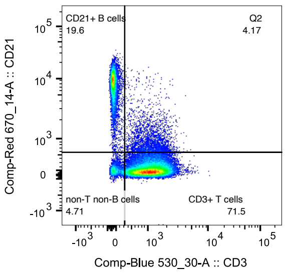

[20180801 Batch3_552_009.fcs]
Lymphocytes
60528

non-T non-B

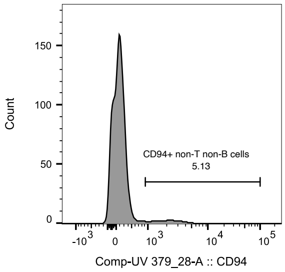

[20180801 Batch3_552_009.fcs]
non-T non-B cells
2848

552
01-AUG-2018

T cells

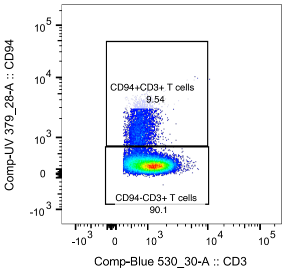

[20180801 Batch3_552_009.fcs]
CD3+ T cells
43275

CD94- T cells

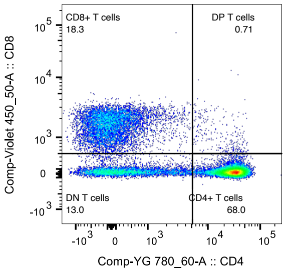

[20180801 Batch3_552_009.fcs]
CD94-CD3+ T cells
39005

CD4 T cells

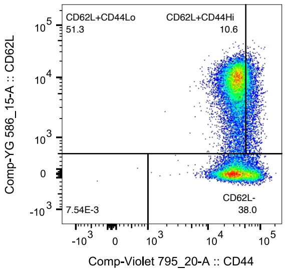

[20180801 Batch3_552_009.fcs]
CD4+ T cells
26528

CD4 T cells

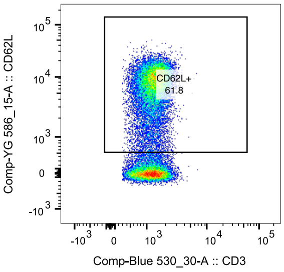

[20180801 Batch3_552_009.fcs]
CD4+ T cells
26528

CD8 T cells

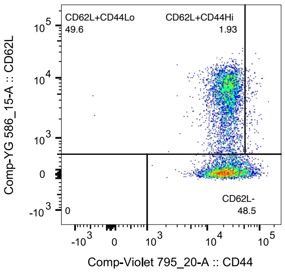

[20180801 Batch3_552_009.fcs]
CD8+ T cells
7133

CD8 T cells

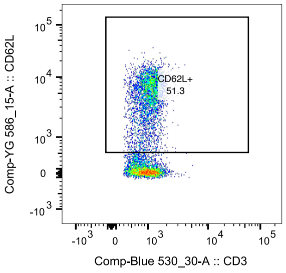

[20180801 Batch3_552_009.fcs]
CD8+ T cells
7133

DN T cells

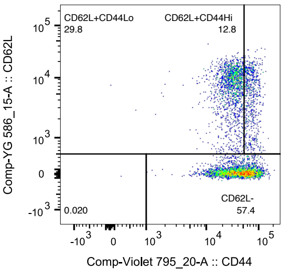

[20180801 Batch3_552_009.fcs]
DN T cells
5066

DN T cells

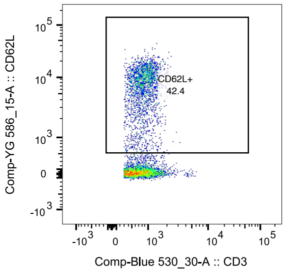

[20180801 Batch3_552_009.fcs]
DN T cells
5066

DP T cells

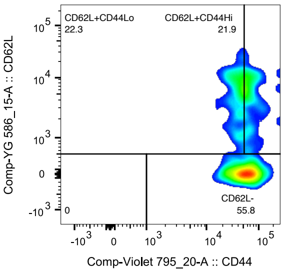

[20180801 Batch3_552_009.fcs]
DP T cells
278

DP T cells

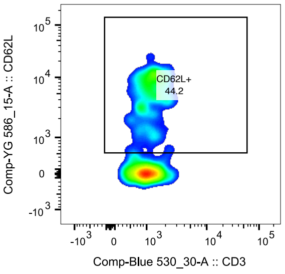

[20180801 Batch3_552_009.fcs]
DP T cells
278

555
01-AUG-2018

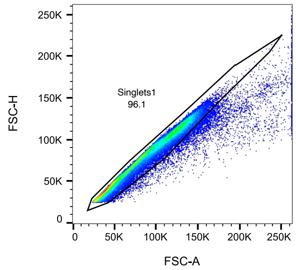

[20180801 Batch3_555_010.fcs]
Ungated
113023

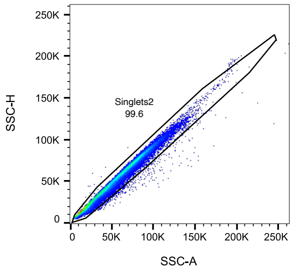

[20180801 Batch3_555_010.fcs]
Singlets1
108615

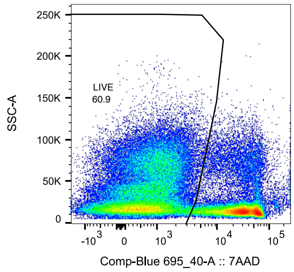

[20180801 Batch3_555_010.fcs]
Singlets2
108172

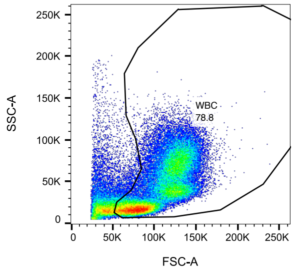

[20180801 Batch3_555_010.fcs]
LIVE
65865

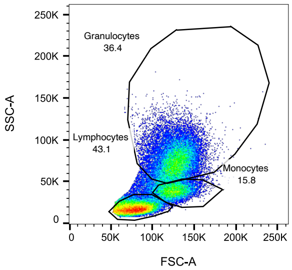

[20180801 Batch3_555_010.fcs]
WBC
51869

Granulocytes

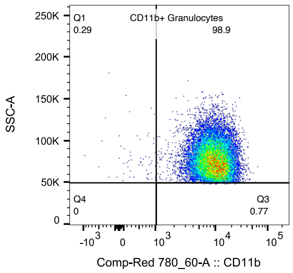

[20180801 Batch3_555_010.fcs]
Granulocytes
18869

Monocytes

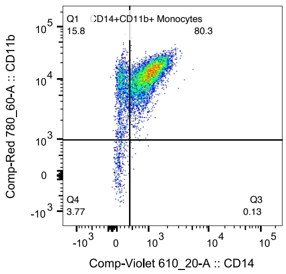

[20180801 Batch3_555_010.fcs]
Monocytes
8202

Lymphocytes

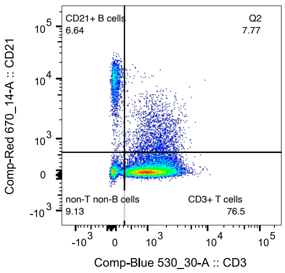

[20180801 Batch3_555_010.fcs]
Lymphocytes
22359

non-T non-B

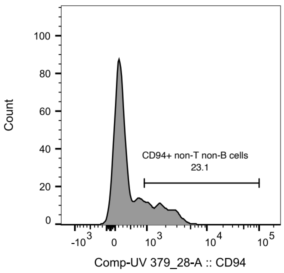

[20180801 Batch3_555_010.fcs]
non-T non-B cells
2041

555
01-AUG-2018

T cells

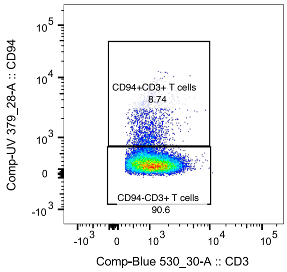

[20180801 Batch3_555_010.fcs]
CD3+ T cells
17095

CD94- T cells

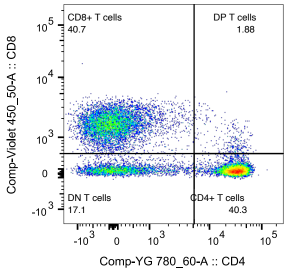

[20180801 Batch3_555_010.fcs]
CD94-CD3+ T cells
15492

CD4 T cells

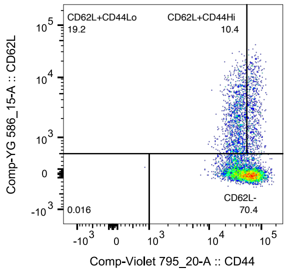

[20180801 Batch3_555_010.fcs]
CD4+ T cells
6245

CD4 T cells

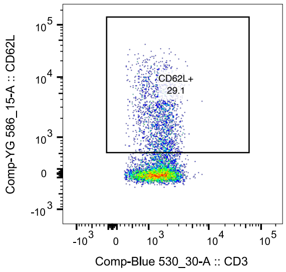

[20180801 Batch3_555_010.fcs]
CD4+ T cells
6245

CD8 T cells

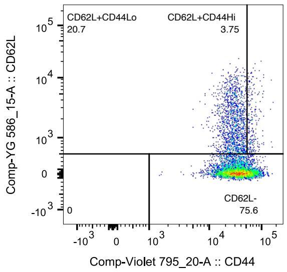

[20180801 Batch3_555_010.fcs]
CD8+ T cells
6300

CD8 T cells

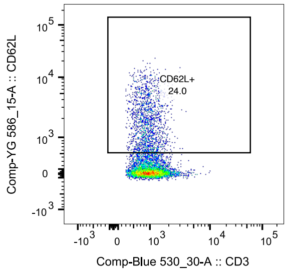

[20180801 Batch3_555_010.fcs]
CD8+ T cells
6300

DN T cells

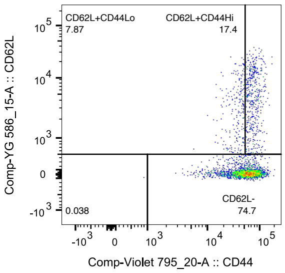

[20180801 Batch3_555_010.fcs]
DN T cells
2655

DN T cells

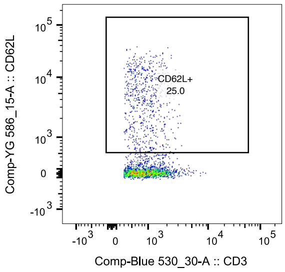

[20180801 Batch3_555_010.fcs]
DN T cells
2655

DP T cells

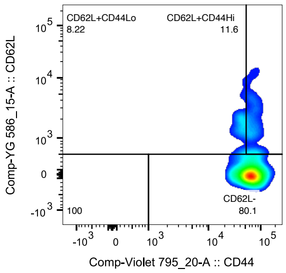

[20180801 Batch3_555_010.fcs]
DP T cells
292

DP T cells

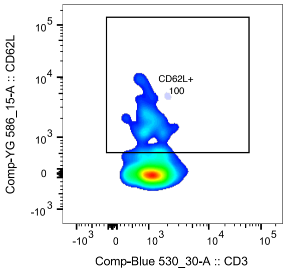

[20180801 Batch3_555_010.fcs]
DP T cells
292

556
01-AUG-2018

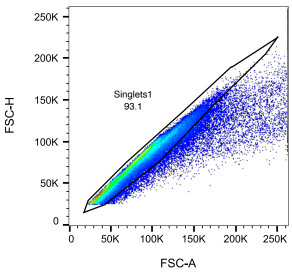

[20180801 Batch3_556_011.fcs]
Ungated
164271

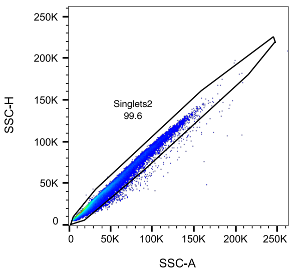

[20180801 Batch3_556_011.fcs]
Singlets1
152969

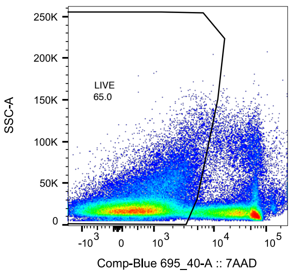

[20180801 Batch3_556_011.fcs]
Singlets2
152377

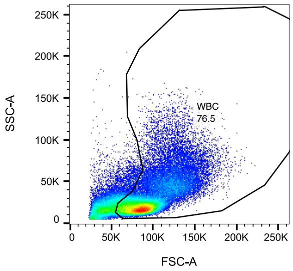

[20180801 Batch3_556_011.fcs]
LIVE
99085

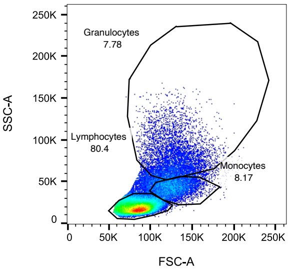

[20180801 Batch3_556_011.fcs]
WBC
75772

Granulocytes

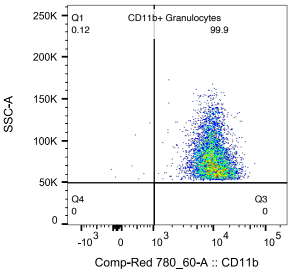

[20180801 Batch3_556_011.fcs]
Granulocytes
5893

Monocytes

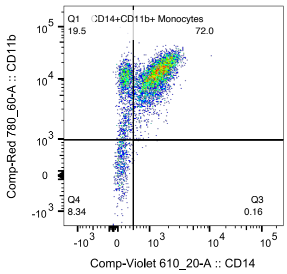

[20180801 Batch3_556_011.fcs]
Monocytes
6187

Lymphocytes

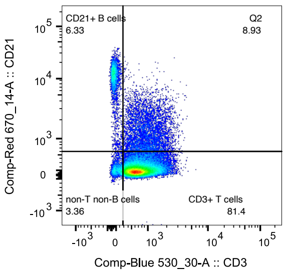

[20180801 Batch3_556_011.fcs]
Lymphocytes
60947

non-T non-B

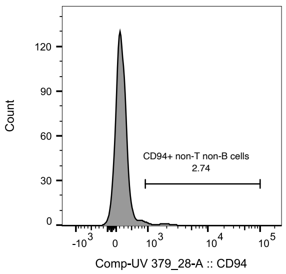

[20180801 Batch3_556_011.fcs]
non-T non-B cells
2046

556
01-AUG-2018

T cells

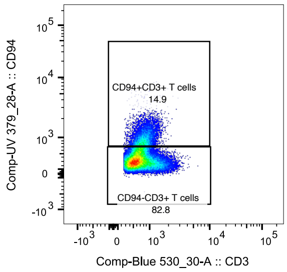

[20180801 Batch3_556_011.fcs]
CD3+ T cells
49605

CD94- T cells

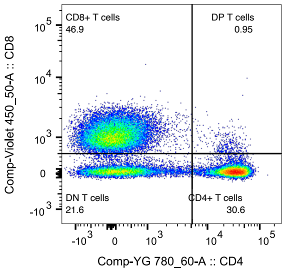

[20180801 Batch3_556_011.fcs]
CD94-CD3+ T cells
41084

CD4 T cells

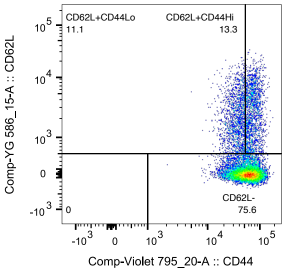

[20180801 Batch3_556_011.fcs]
CD4+ T cells
12574

CD4 T cells

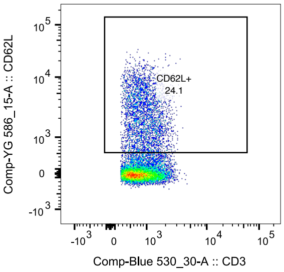

[20180801 Batch3_556_011.fcs]
CD4+ T cells
12574

CD8 T cells

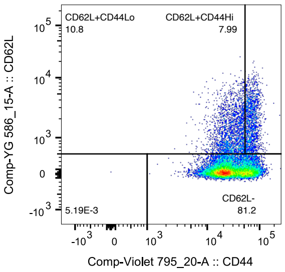

[20180801 Batch3_556_011.fcs]
CD8+ T cells
19264

CD8 T cells

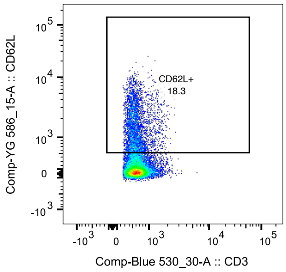

[20180801 Batch3_556_011.fcs]
CD8+ T cells
19264

DN T cells

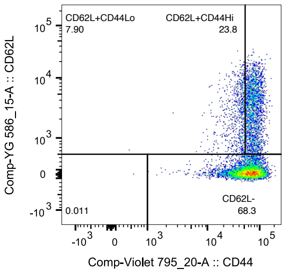

[20180801 Batch3_556_011.fcs]
DN T cells
8857

DN T cells

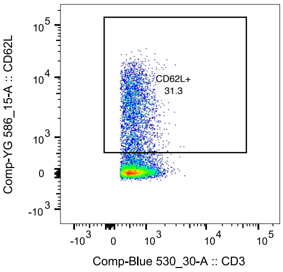

[20180801 Batch3_556_011.fcs]
DN T cells
8857

DP T cells

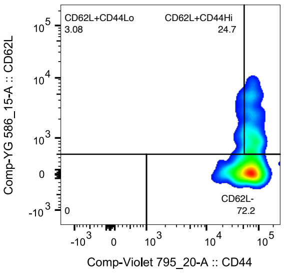

[20180801 Batch3_556_011.fcs]
DP T cells
389

DP T cells

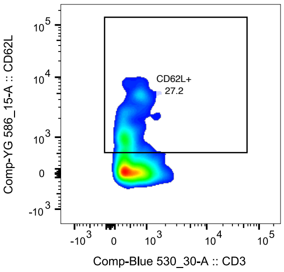

[20180801 Batch3_556_011.fcs]
DP T cells
389

562
01-AUG-2018

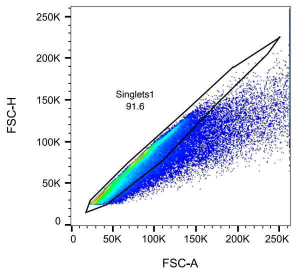

[20180801 Batch3_562_012.fcs]
Ungated
180409

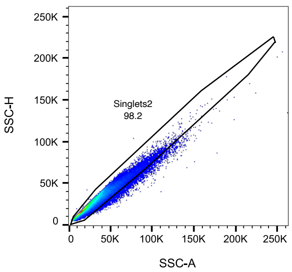

[20180801 Batch3_562_012.fcs]
Singlets1
165288

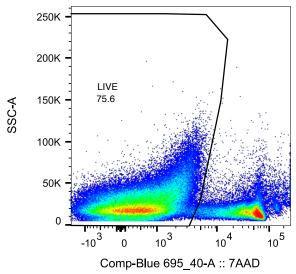

[20180801 Batch3_562_012.fcs]
Singlets2
162360

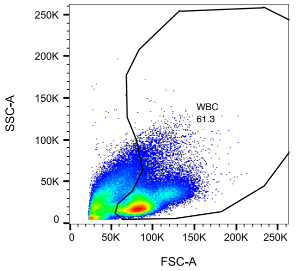

[20180801 Batch3_562_012.fcs]
LIVE
122710

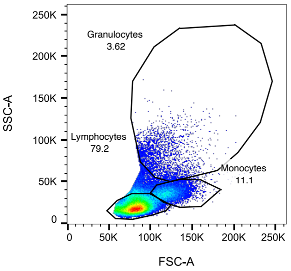

[20180801 Batch3_562_012.fcs]
WBC
75246

Granulocytes

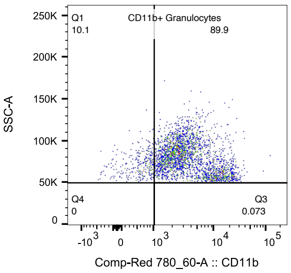

[20180801 Batch3_562_012.fcs]
Granulocytes
2724

Monocytes

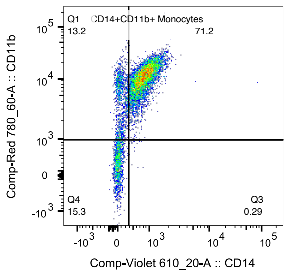

[20180801 Batch3_562_012.fcs]
Monocytes
8373

Lymphocytes

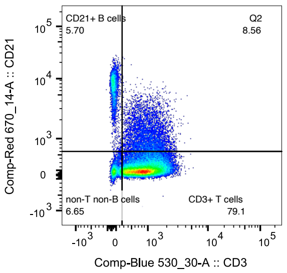

[20180801 Batch3_562_012.fcs]
Lymphocytes
59609

non-T non-B

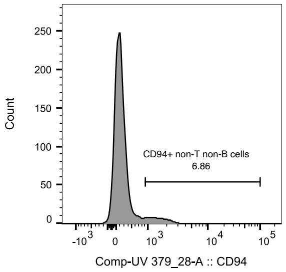

[20180801 Batch3_562_012.fcs]
non-T non-B cells
3963

562
01-AUG-2018

T cells

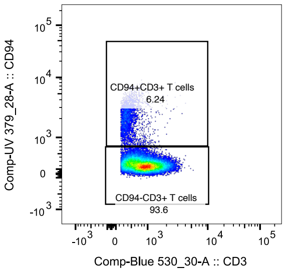

[20180801 Batch3_562_012.fcs]
CD3+ T cells
47148

CD94- T cells

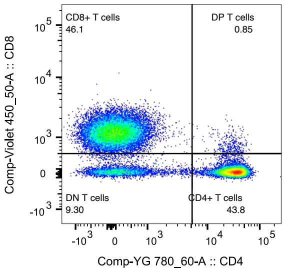

[20180801 Batch3_562_012.fcs]
CD94-CD3+ T cells
44124

CD4 T cells

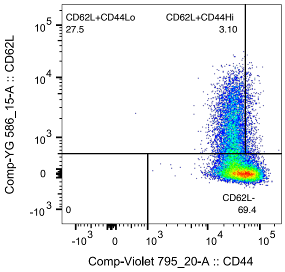

[20180801 Batch3_562_012.fcs]
CD4+ T cells
19320

CD4 T cells

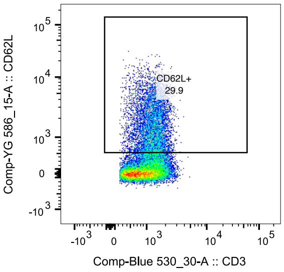

[20180801 Batch3_562_012.fcs]
CD4+ T cells
19320

CD8 T cells

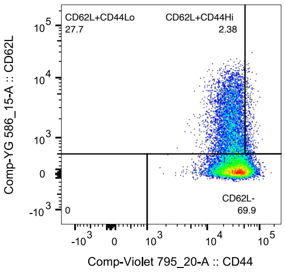

[20180801 Batch3_562_012.fcs]
CD8+ T cells
20322

CD8 T cells

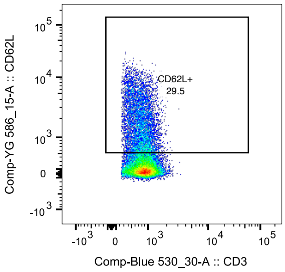

[20180801 Batch3_562_012.fcs]
CD8+ T cells
20322

DN T cells

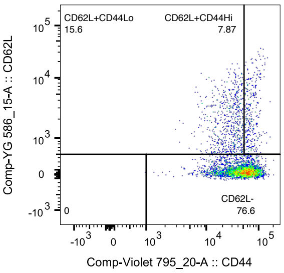

[20180801 Batch3_562_012.fcs]
DN T cells
4105

DN T cells

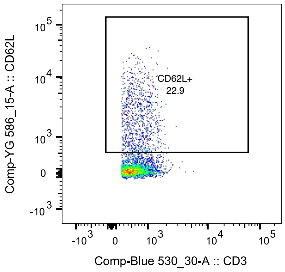

[20180801 Batch3_562_012.fcs]
DN T cells
4105

DP T cells

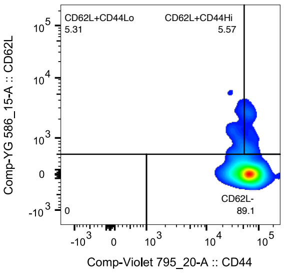

[20180801 Batch3_562_012.fcs]
DP T cells
377

DP T cells

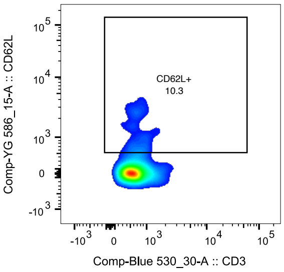

[20180801 Batch3_562_012.fcs]
DP T cells
377

566
01-AUG-2018

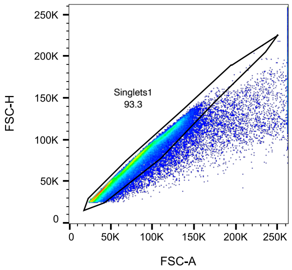

[20180801 Batch3_566_013.fcs]
Ungated
108443

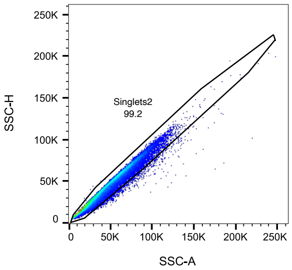

[20180801 Batch3_566_013.fcs]
Singlets1
101205

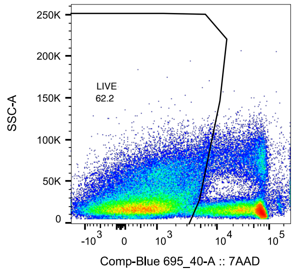

[20180801 Batch3_566_013.fcs]
Singlets2
100427

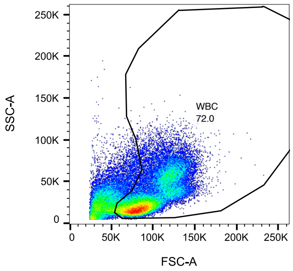

[20180801 Batch3_566_013.fcs]
LIVE
62417

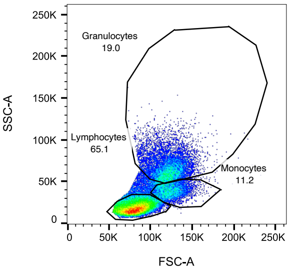

[20180801 Batch3_566_013.fcs]
WBC
44942

Granulocytes

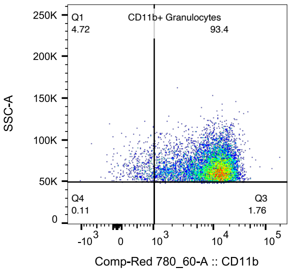

[20180801 Batch3_566_013.fcs]
Granulocytes
8521

Monocytes

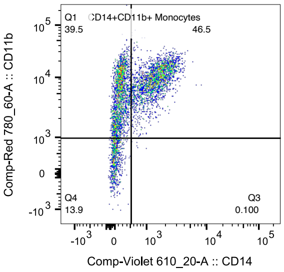

[20180801 Batch3_566_013.fcs]
Monocytes
5012

Lymphocytes

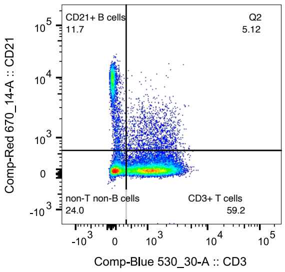

[20180801 Batch3_566_013.fcs]
Lymphocytes
29254

non-T non-B

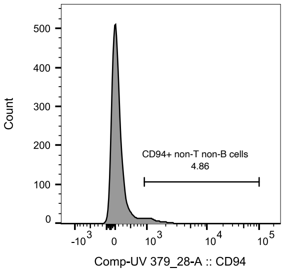

[20180801 Batch3_566_013.fcs]
non-T non-B cells
7014

566
01-AUG-2018

T cells

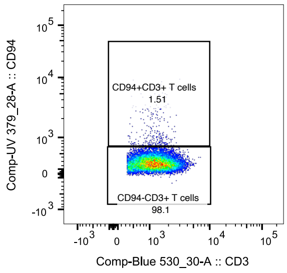

[20180801 Batch3_566_013.fcs]
CD3+ T cells
17330

CD94- T cells

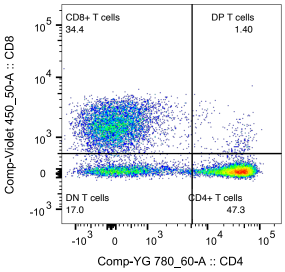

[20180801 Batch3_566_013.fcs]
CD94-CD3+ T cells
17000

CD4 T cells

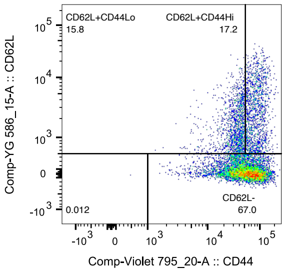

[20180801 Batch3_566_013.fcs]
CD4+ T cells
8036

CD4 T cells

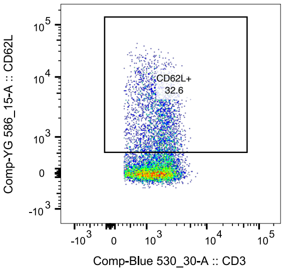

[20180801 Batch3_566_013.fcs]
CD4+ T cells
8036

CD8 T cells

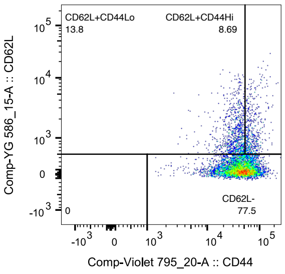

[20180801 Batch3_566_013.fcs]
CD8+ T cells
5843

CD8 T cells

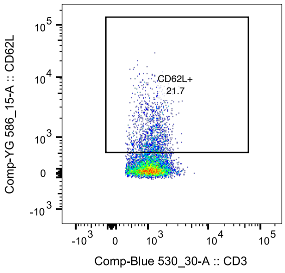

[20180801 Batch3_566_013.fcs]
CD8+ T cells
5843

DN T cells

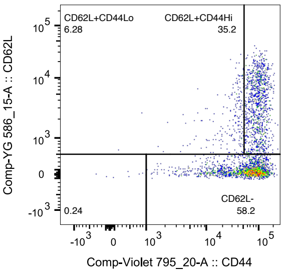

[20180801 Batch3_566_013.fcs]
DN T cells
2883

DN T cells

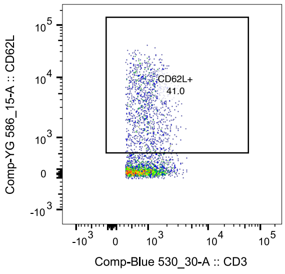

[20180801 Batch3_566_013.fcs]
DN T cells
2883

DP T cells

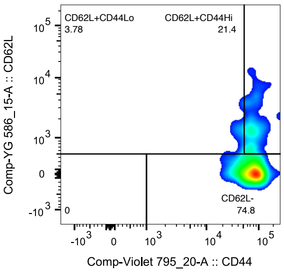

[20180801 Batch3_566_013.fcs]
DP T cells
238

DP T cells

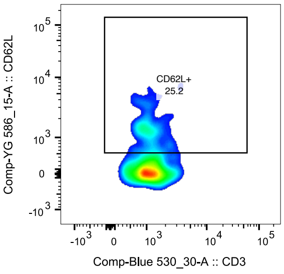

[20180801 Batch3_566_013.fcs]
DP T cells
238

573
01-AUG-2018

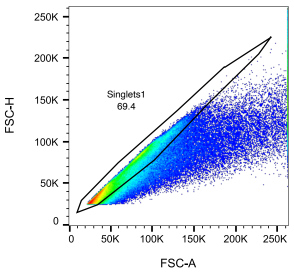

[20180801 Batch3_573_014.fcs]
Ungated
146832

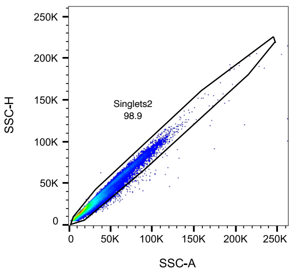

[20180801 Batch3_573_014.fcs]
Singlets1
101966

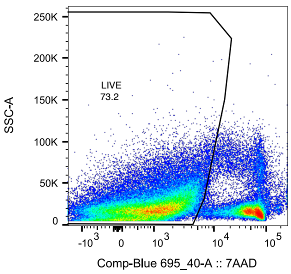

[20180801 Batch3_573_014.fcs]
Singlets2
100891

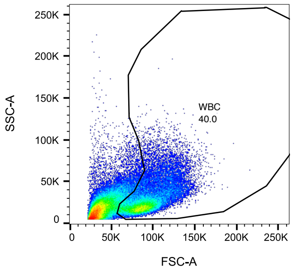

[20180801 Batch3_573_014.fcs]
LIVE
73885

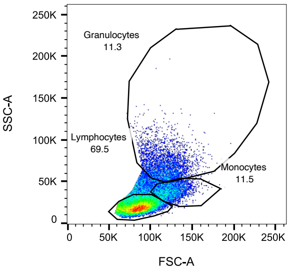

[20180801 Batch3_573_014.fcs]
WBC
29585

Granulocytes

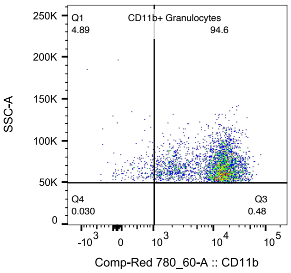

[20180801 Batch3_573_014.fcs]
Granulocytes
3357

Monocytes

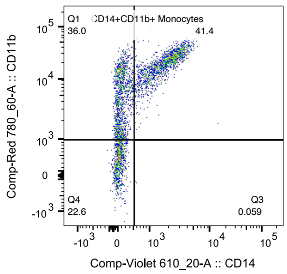

[20180801 Batch3_573_014.fcs]
Monocytes
3395

Lymphocytes

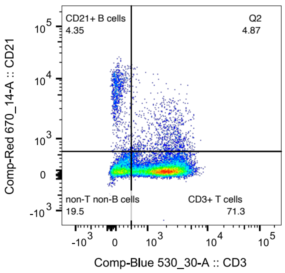

[20180801 Batch3_573_014.fcs]
Lymphocytes
20552

non-T non-B

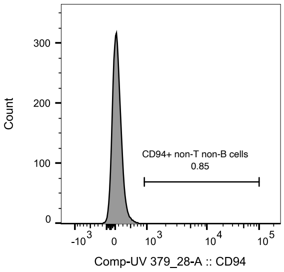

[20180801 Batch3_573_014.fcs]
non-T non-B cells
3999

573
01-AUG-2018

T cells

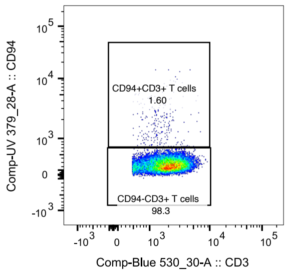

[20180801 Batch3_573_014.fcs]
CD3+ T cells
14659

CD94- T cells

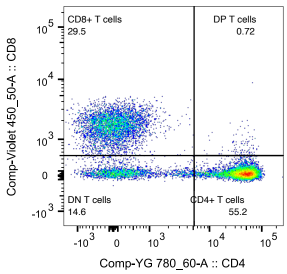

[20180801 Batch3_573_014.fcs]
CD94-CD3+ T cells
14405

CD4 T cells

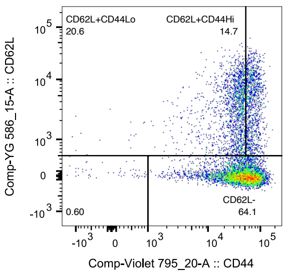

[20180801 Batch3_573_014.fcs]
CD4+ T cells
7948

CD4 T cells

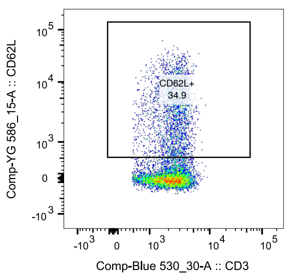

[20180801 Batch3_573_014.fcs]
CD4+ T cells
7948

CD8 T cells

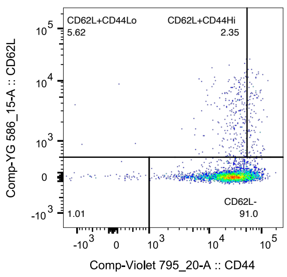

[20180801 Batch3_573_014.fcs]
CD8+ T cells
4249

CD8 T cells

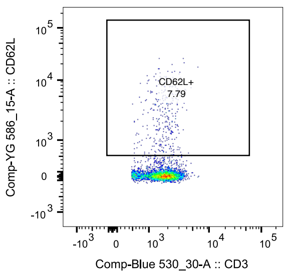

[20180801 Batch3_573_014.fcs]
CD8+ T cells
4249

DN T cells

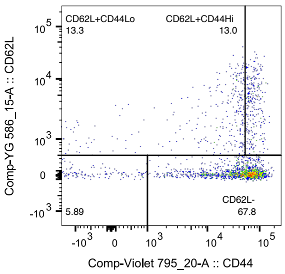

[20180801 Batch3_573_014.fcs]
DN T cells
2104

DN T cells

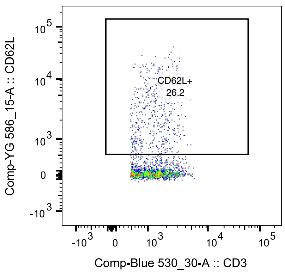

[20180801 Batch3_573_014.fcs]
DN T cells
2104

DP T cells

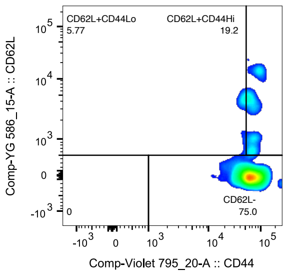

[20180801 Batch3_573_014.fcs]
DP T cells
104

DP T cells

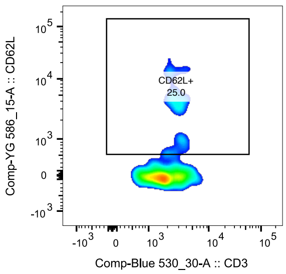

[20180801 Batch3_573_014.fcs]
DP T cells
104

574
01-AUG-2018

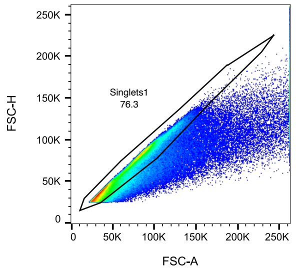

[20180801 Batch3_574_015.fcs]
Ungated
215233

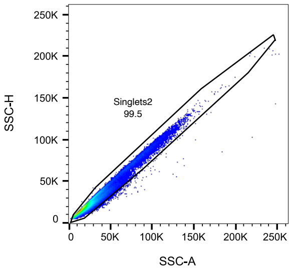

[20180801 Batch3_574_015.fcs]
Singlets1
164146

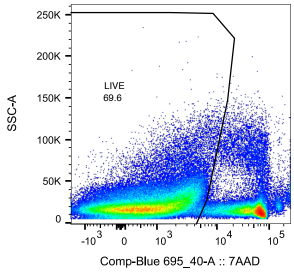

[20180801 Batch3_574_015.fcs]
Singlets2
163282

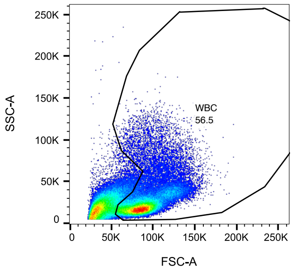

[20180801 Batch3_574_015.fcs]
LIVE
113642

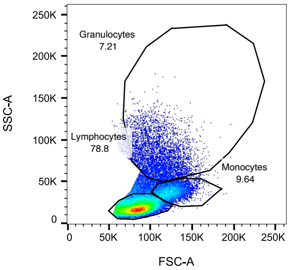

[20180801 Batch3_574_015.fcs]
WBC
64159

Granulocytes

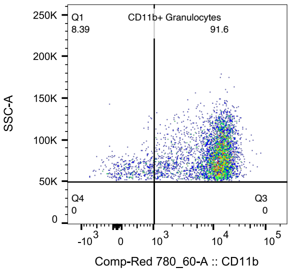

[20180801 Batch3_574_015.fcs]
Granulocytes
4625

Monocytes

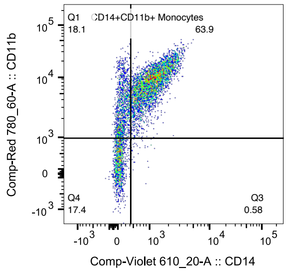

[20180801 Batch3_574_015.fcs]
Monocytes
6185

Lymphocytes

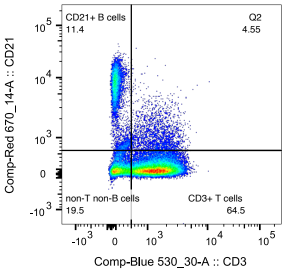

[20180801 Batch3_574_015.fcs]
Lymphocytes
50537

non-T non-B

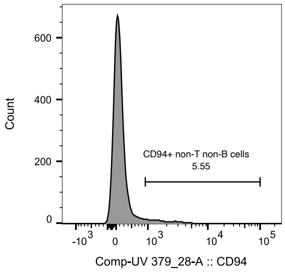

[20180801 Batch3_574_015.fcs]
non-T non-B cells
9861

574
01-AUG-2018

T cells

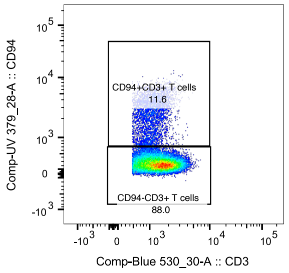

[20180801 Batch3_574_015.fcs]
CD3+ T cells
32616

CD94- T cells

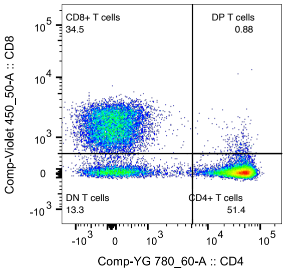

[20180801 Batch3_574_015.fcs]
CD94-CD3+ T cells
28695

CD4 T cells

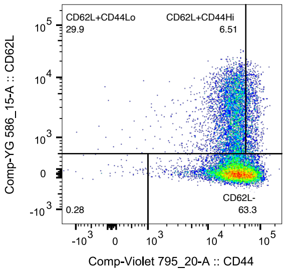

[20180801 Batch3_574_015.fcs]
CD4+ T cells
14740

CD4 T cells

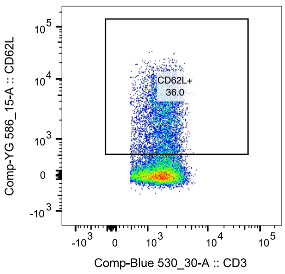

[20180801 Batch3_574_015.fcs]
CD4+ T cells
14740

CD8 T cells

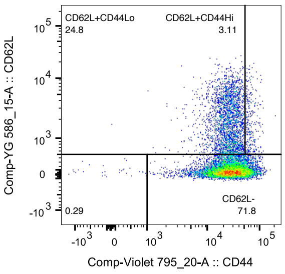

[20180801 Batch3_574_015.fcs]
CD8+ T cells
9888

CD8 T cells

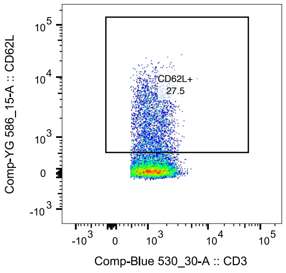

[20180801 Batch3_574_015.fcs]
CD8+ T cells
9888

DN T cells

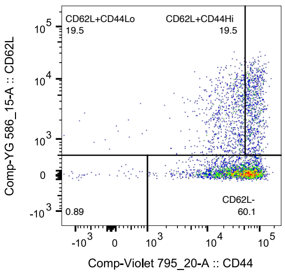

[20180801 Batch3_574_015.fcs]
DN T cells
3815

DN T cells

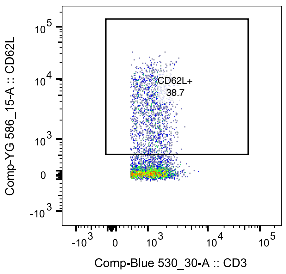

[20180801 Batch3_574_015.fcs]
DN T cells
3815

DP T cells

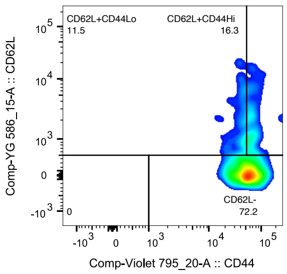

[20180801 Batch3_574_015.fcs]
DP T cells
252

DP T cells

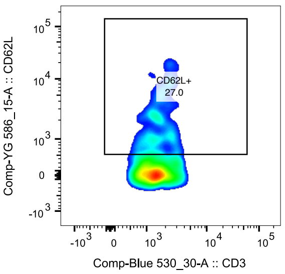

[20180801 Batch3_574_015.fcs]
DP T cells
252

576
01-AUG-2018

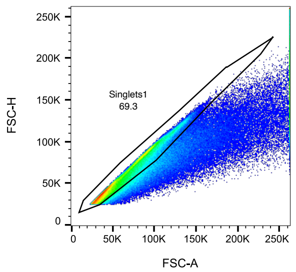

[20180801 Batch3_576_016.fcs]
Ungated
266950

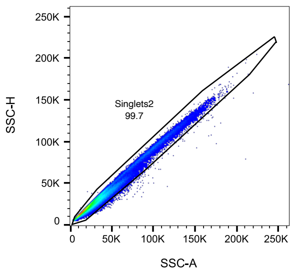

[20180801 Batch3_576_016.fcs]
Singlets1
185089

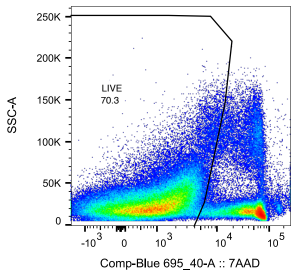

[20180801 Batch3_576_016.fcs]
Singlets2
184482

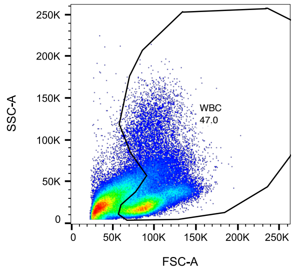

[20180801 Batch3_576_016.fcs]
LIVE
129772

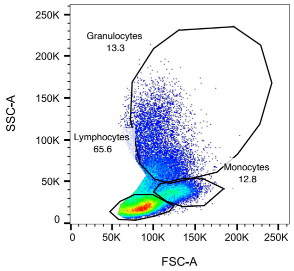

[20180801 Batch3_576_016.fcs]
WBC
60969

Granulocytes

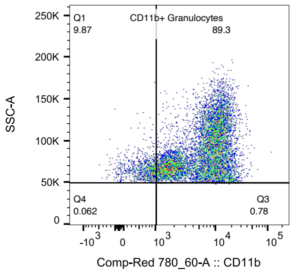

[20180801 Batch3_576_016.fcs]
Granulocytes
8085

Monocytes

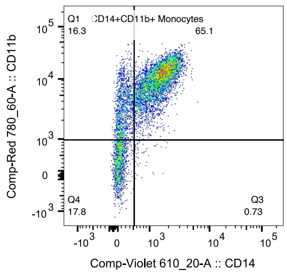

[20180801 Batch3_576_016.fcs]
Monocytes
7834

Lymphocytes

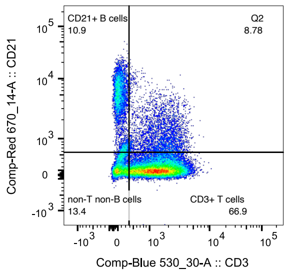

[20180801 Batch3_576_016.fcs]
Lymphocytes
39977

non-T non-B

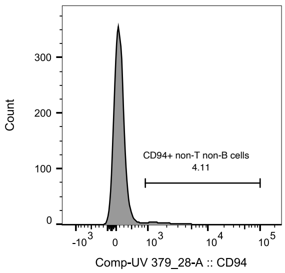

[20180801 Batch3_576_016.fcs]
non-T non-B cells
5357

576
01-AUG-2018

T cells

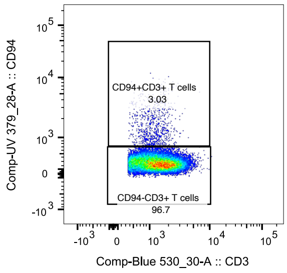

[20180801 Batch3_576_016.fcs]
CD3+ T cells
26760

CD94- T cells

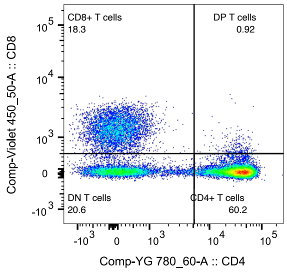

[20180801 Batch3_576_016.fcs]
CD94-CD3+ T cells
25886

CD4 T cells

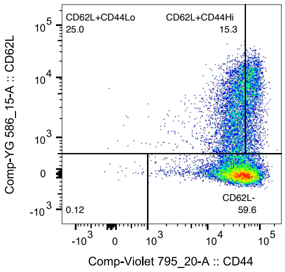

[20180801 Batch3_576_016.fcs]
CD4+ T cells
15585

CD4 T cells

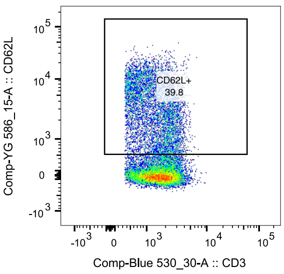

[20180801 Batch3_576_016.fcs]
CD4+ T cells
15585

CD8 T cells

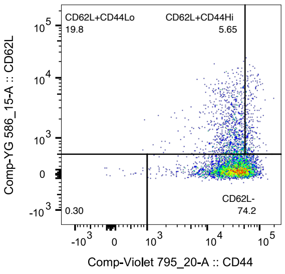

[20180801 Batch3_576_016.fcs]
CD8+ T cells
4726

CD8 T cells

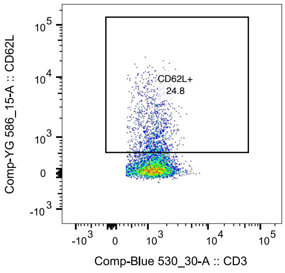

[20180801 Batch3_576_016.fcs]
CD8+ T cells
4726

DN T cells

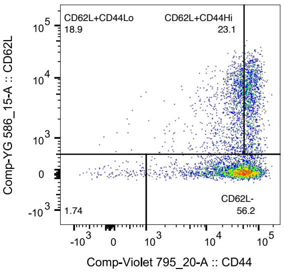

[20180801 Batch3_576_016.fcs]
DN T cells
5338

DN T cells

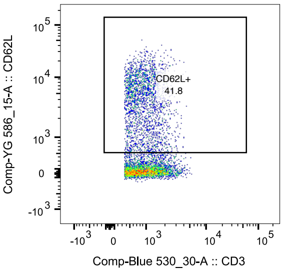

[20180801 Batch3_576_016.fcs]
DN T cells
5338

DP T cells

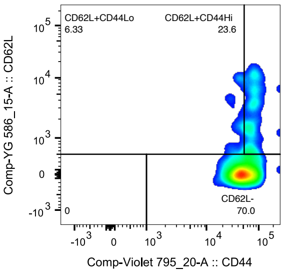

[20180801 Batch3_576_016.fcs]
DP T cells
237

DP T cells

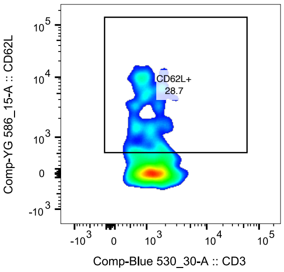

[20180801 Batch3_576_016.fcs]
DP T cells
237

578
01-AUG-2018

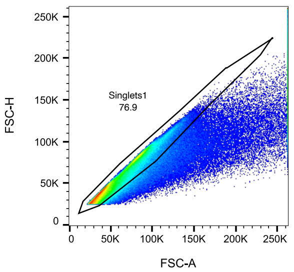

[20180801 Batch3_578_017.fcs]
Ungated
262905

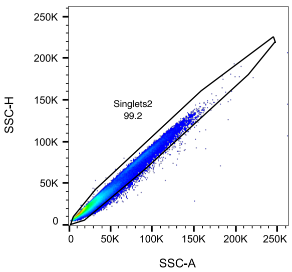

[20180801 Batch3_578_017.fcs]
Singlets1
202249

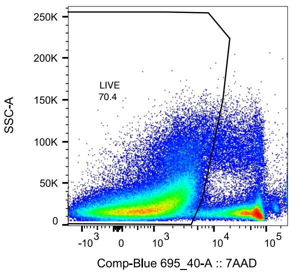

[20180801 Batch3_578_017.fcs]
Singlets2
200713

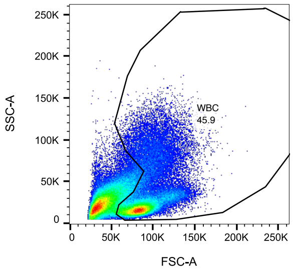

[20180801 Batch3_578_017.fcs]
LIVE
141311

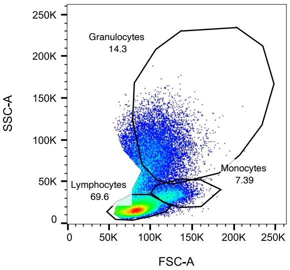

[20180801 Batch3_578_017.fcs]
WBC
64900

Granulocytes

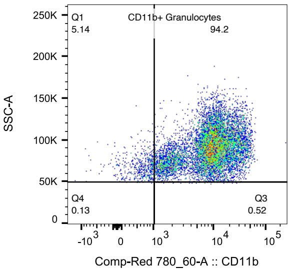

[20180801 Batch3_578_017.fcs]
Granulocytes
9277

Monocytes

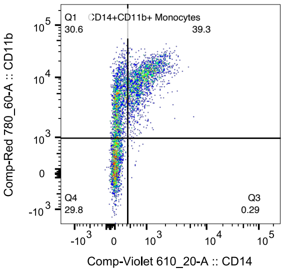

[20180801 Batch3_578_017.fcs]
Monocytes
4793

Lymphocytes

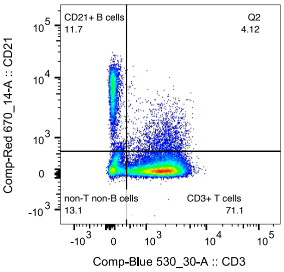

[20180801 Batch3_578_017.fcs]
Lymphocytes
45179

non-T non-B

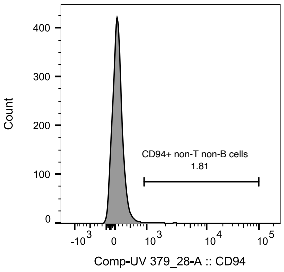

[20180801 Batch3_578_017.fcs]
non-T non-B cells
5912

578
01-AUG-2018

T cells

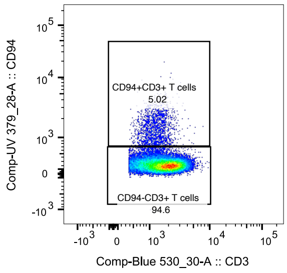

[20180801 Batch3_578_017.fcs]
CD3+ T cells
32128

CD94- T cells

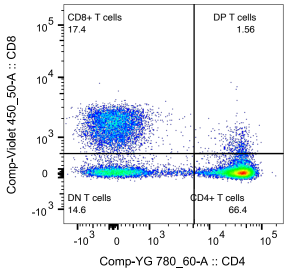

[20180801 Batch3_578_017.fcs]
CD94-CD3+ T cells
30407

CD4 T cells

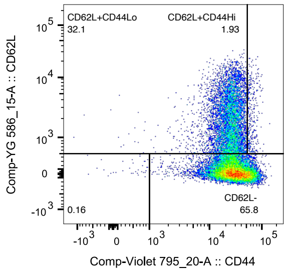

[20180801 Batch3_578_017.fcs]
CD4+ T cells
20199

CD4 T cells

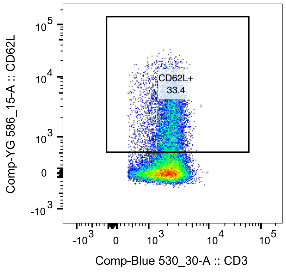

[20180801 Batch3_578_017.fcs]
CD4+ T cells
20199

CD8 T cells

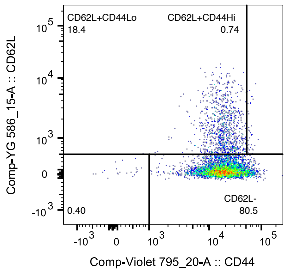

[20180801 Batch3_578_017.fcs]
CD8+ T cells
5300

CD8 T cells

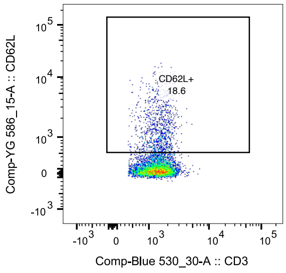

[20180801 Batch3_578_017.fcs]
CD8+ T cells
5300

DN T cells

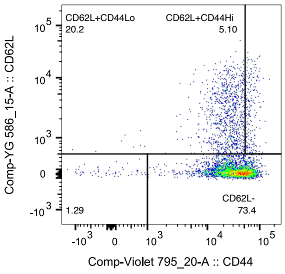

[20180801 Batch3_578_017.fcs]
DN T cells
4433

DN T cells

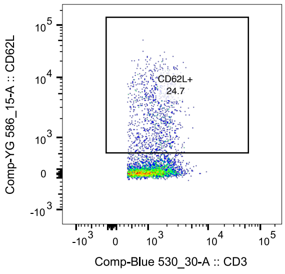

[20180801 Batch3_578_017.fcs]
DN T cells
4433

DP T cells

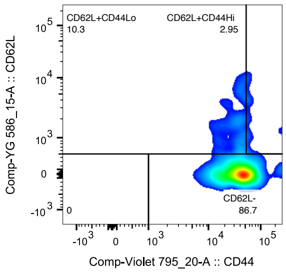

[20180801 Batch3_578_017.fcs]
DP T cells
475

DP T cells

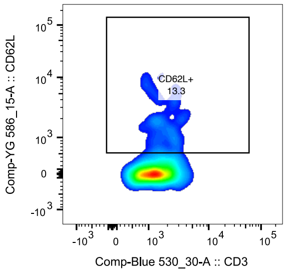

[20180801 Batch3_578_017.fcs]
DP T cells
475

580
01-AUG-2018

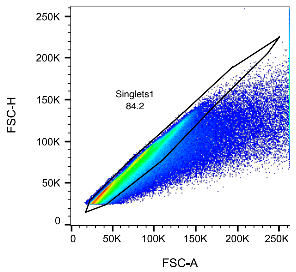

[20180801 Batch3_580_018.fcs]
Ungated
286893

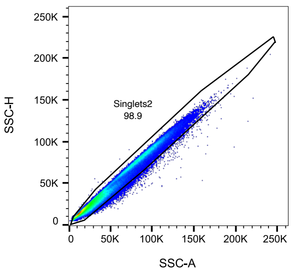

[20180801 Batch3_580_018.fcs]
Singlets1
241540

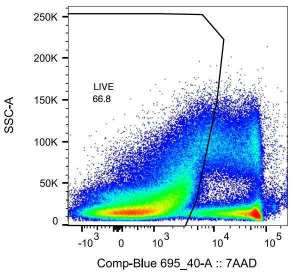

[20180801 Batch3_580_018.fcs]
Singlets2
238896

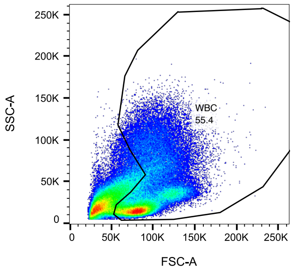

[20180801 Batch3_580_018.fcs]
LIVE
159613

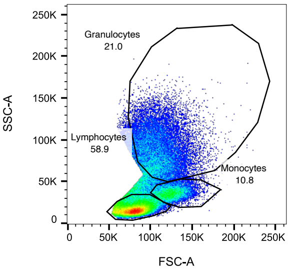

[20180801 Batch3_580_018.fcs]
WBC
88453

Granulocytes

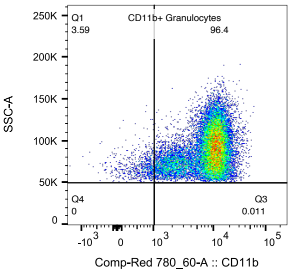

[20180801 Batch3_580_018.fcs]
Granulocytes
18540

Monocytes

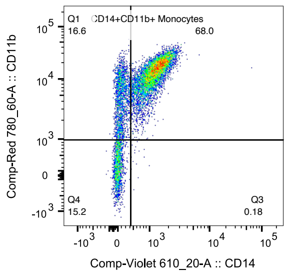

[20180801 Batch3_580_018.fcs]
Monocytes
9554

Lymphocytes

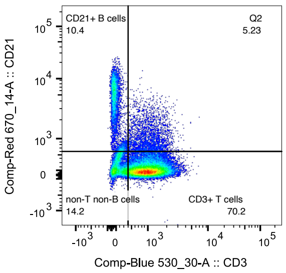

[20180801 Batch3_580_018.fcs]
Lymphocytes
52101

non-T non-B

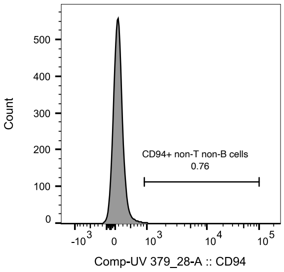

[20180801 Batch3_580_018.fcs]
non-T non-B cells
7378

580
01-AUG-2018

T cells

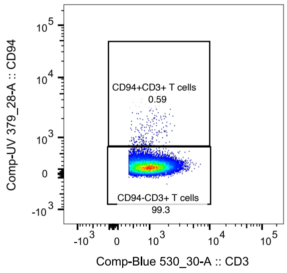

[20180801 Batch3_580_018.fcs]
CD3+ T cells
36566

CD94- T cells

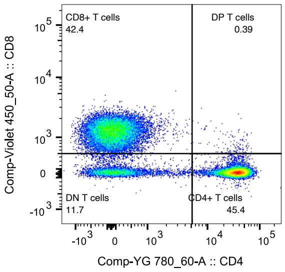

[20180801 Batch3_580_018.fcs]
CD94-CD3+ T cells
36325

CD4 T cells

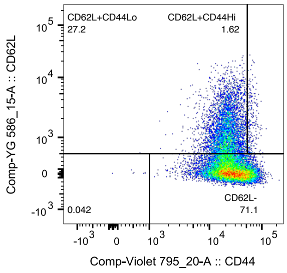

[20180801 Batch3_580_018.fcs]
CD4+ T cells
16505

CD4 T cells

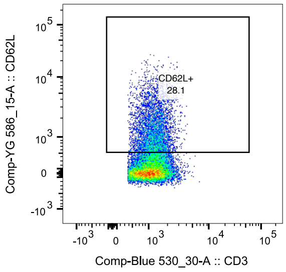

[20180801 Batch3_580_018.fcs]
CD4+ T cells
16505

CD8 T cells

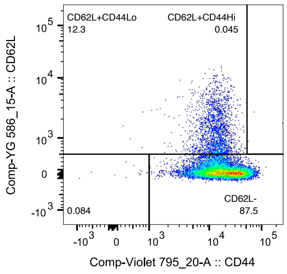

[20180801 Batch3_580_018.fcs]
CD8+ T cells
15410

CD8 T cells

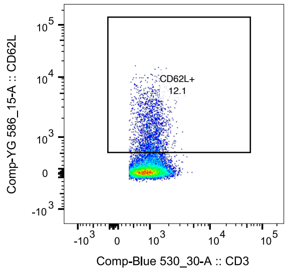

[20180801 Batch3_580_018.fcs]
CD8+ T cells
15410

DN T cells

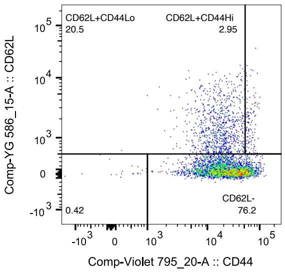

[20180801 Batch3_580_018.fcs]
DN T cells
4268

DN T cells

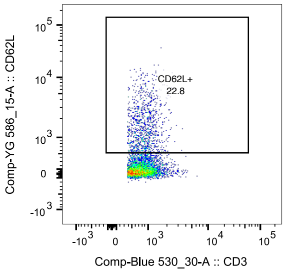

[20180801 Batch3_580_018.fcs]
DN T cells
4268

DP T cells

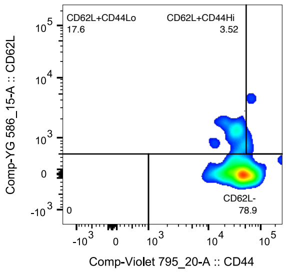

[20180801 Batch3_580_018.fcs]
DP T cells
142

DP T cells

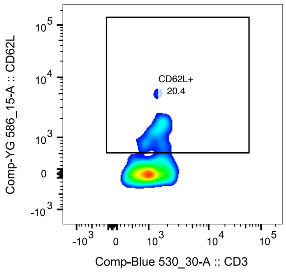

[20180801 Batch3_580_018.fcs]
DP T cells
142

585
01-AUG-2018

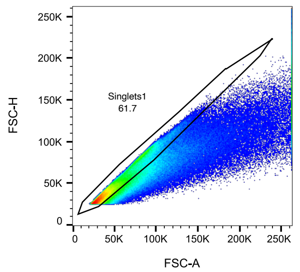

[20180801 Batch3_585_019.fcs]
Ungated
338979

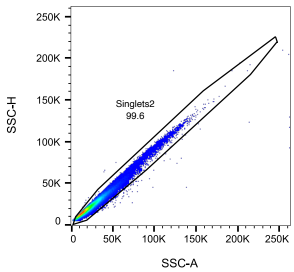

[20180801 Batch3_585_019.fcs]
Singlets1
209079

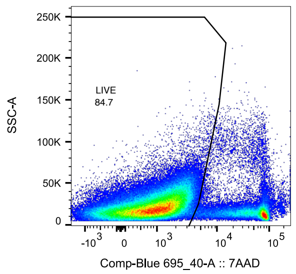

[20180801 Batch3_585_019.fcs]
Singlets2
208148

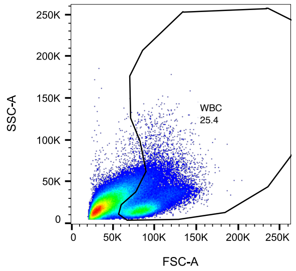

[20180801 Batch3_585_019.fcs]
LIVE
176353

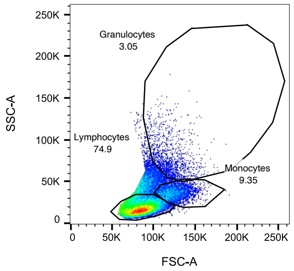

[20180801 Batch3_585_019.fcs]
WBC
44793

Granulocytes

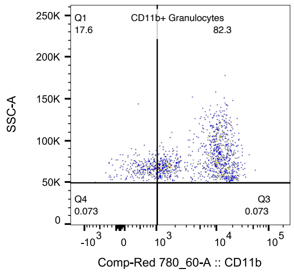

[20180801 Batch3_585_019.fcs]
Granulocytes
1367

Monocytes

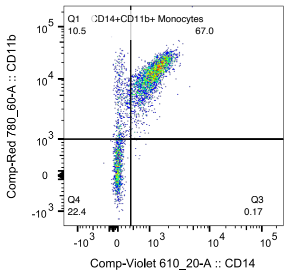

[20180801 Batch3_585_019.fcs]
Monocytes
4188

Lymphocytes

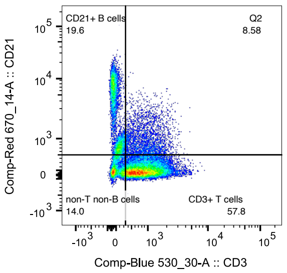

[20180801 Batch3_585_019.fcs]
Lymphocytes
33535

non-T non-B

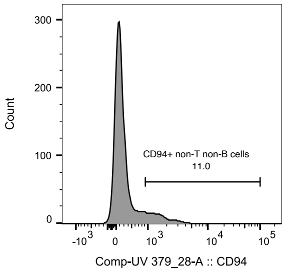

[20180801 Batch3_585_019.fcs]
non-T non-B cells
4709

585
01-AUG-2018

T cells

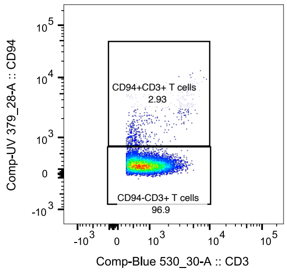

[20180801 Batch3_585_019.fcs]
CD3+ T cells
19391

CD94- T cells

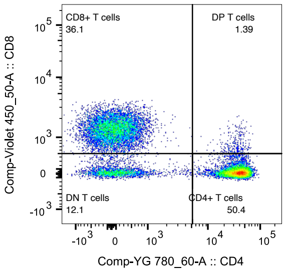

[20180801 Batch3_585_019.fcs]
CD94-CD3+ T cells
18786

CD4 T cells

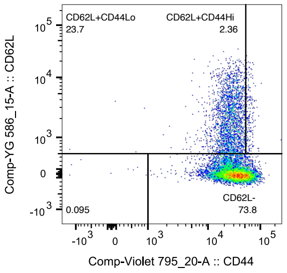

[20180801 Batch3_585_019.fcs]
CD4+ T cells
9461

CD4 T cells

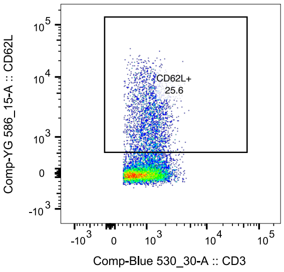

[20180801 Batch3_585_019.fcs]
CD4+ T cells
9461

CD8 T cells

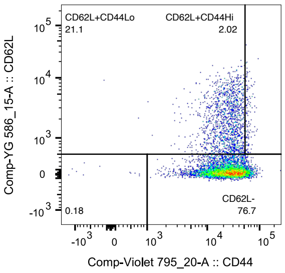

[20180801 Batch3_585_019.fcs]
CD8+ T cells
6787

CD8 T cells

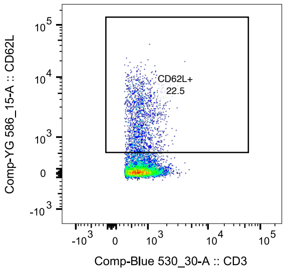

[20180801 Batch3_585_019.fcs]
CD8+ T cells
6787

DN T cells

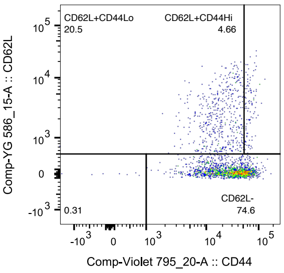

[20180801 Batch3_585_019.fcs]
DN T cells
2277

DN T cells

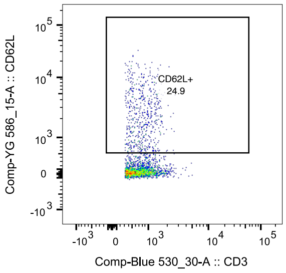

[20180801 Batch3_585_019.fcs]
DN T cells
2277

DP T cells

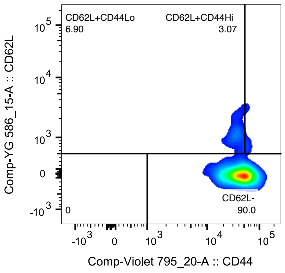

[20180801 Batch3_585_019.fcs]
DP T cells
261

DP T cells

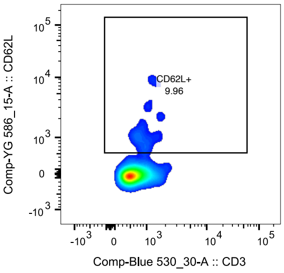

[20180801 Batch3_585_019.fcs]
DP T cells
261

590
01-AUG-2018

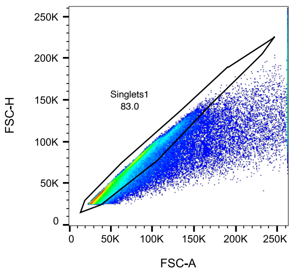

[20180801 Batch3_590_020.fcs]
Ungated
125742

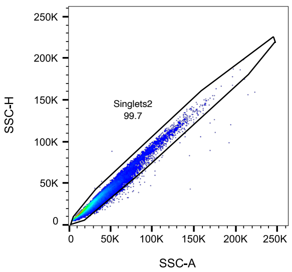

[20180801 Batch3_590_020.fcs]
Singlets1
104425

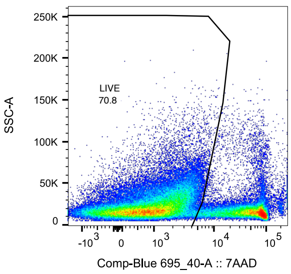

[20180801 Batch3_590_020.fcs]
Singlets2
104110

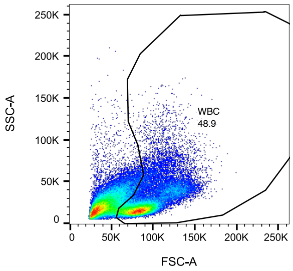

[20180801 Batch3_590_020.fcs]
LIVE
73711

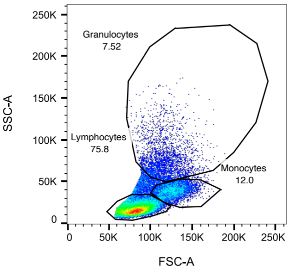

[20180801 Batch3_590_020.fcs]
WBC
36058

Granulocytes

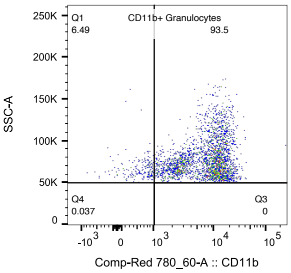

[20180801 Batch3_590_020.fcs]
Granulocytes
2710

Monocytes

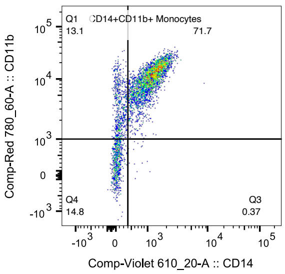

[20180801 Batch3_590_020.fcs]
Monocytes
4314

Lymphocytes

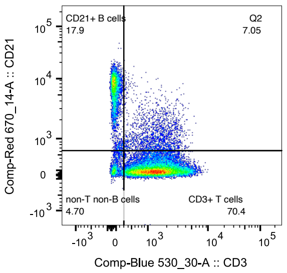

[20180801 Batch3_590_020.fcs]
Lymphocytes
27320

non-T non-B

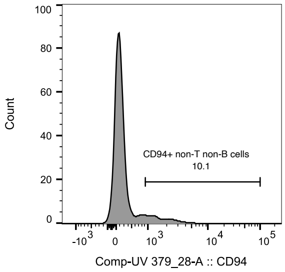

[20180801 Batch3_590_020.fcs]
non-T non-B cells
1283

590
01-AUG-2018

T cells

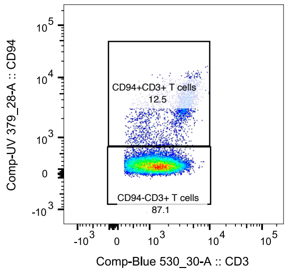

[20180801 Batch3_590_020.fcs]
CD3+ T cells
19233

CD94- T cells

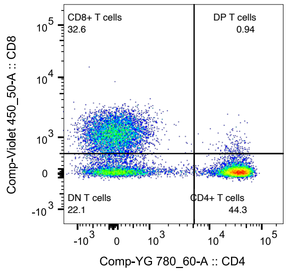

[20180801 Batch3_590_020.fcs]
CD94-CD3+ T cells
16759

CD4 T cells

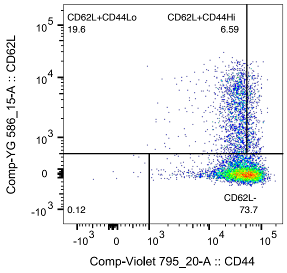

[20180801 Batch3_590_020.fcs]
CD4+ T cells
7425

CD4 T cells

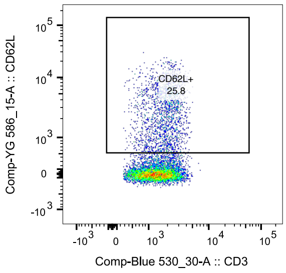

[20180801 Batch3_590_020.fcs]
CD4+ T cells
7425

CD8 T cells

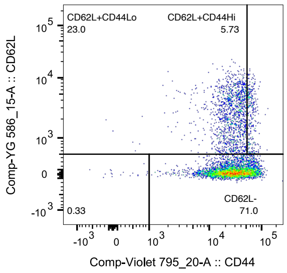

[20180801 Batch3_590_020.fcs]
CD8+ T cells
5467

CD8 T cells

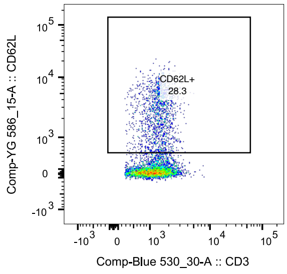

[20180801 Batch3_590_020.fcs]
CD8+ T cells
5467

DN T cells

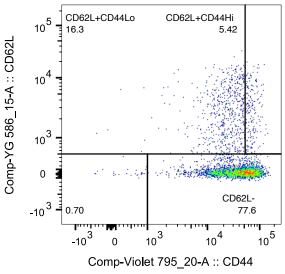

[20180801 Batch3_590_020.fcs]
DN T cells
3709

DN T cells

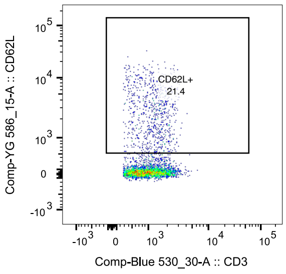

[20180801 Batch3_590_020.fcs]
DN T cells
3709

DP T cells

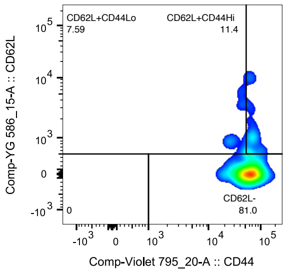

[20180801 Batch3_590_020.fcs]
DP T cells
158

DP T cells

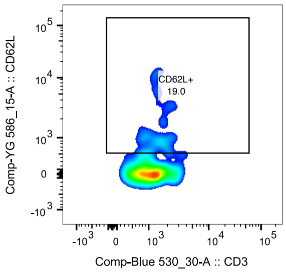

[20180801 Batch3_590_020.fcs]
DP T cells
158

592
01-AUG-2018

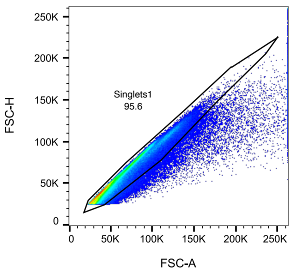

[20180801 Batch3_592_021.fcs]
Ungated
275203

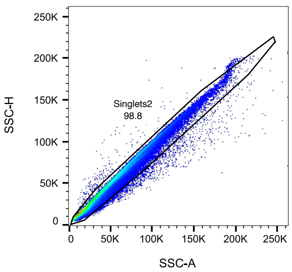

[20180801 Batch3_592_021.fcs]
Singlets1
262960

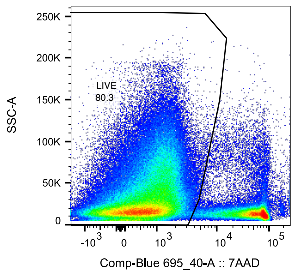

[20180801 Batch3_592_021.fcs]
Singlets2
259678

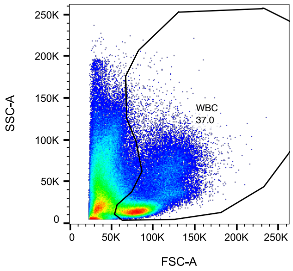

[20180801 Batch3_592_021.fcs]
LIVE
208402

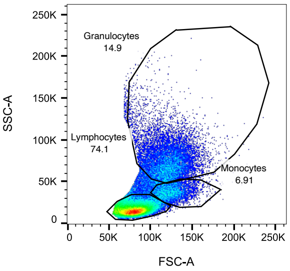

[20180801 Batch3_592_021.fcs]
WBC
77141

Granulocytes

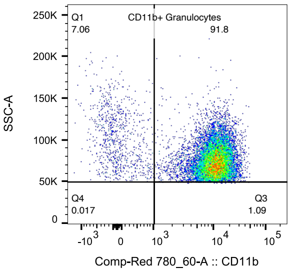

[20180801 Batch3_592_021.fcs]
Granulocytes
11508

Monocytes

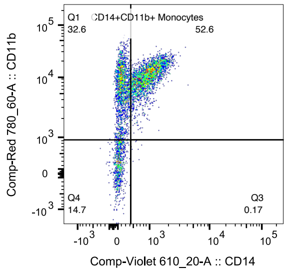

[20180801 Batch3_592_021.fcs]
Monocytes
5330

Lymphocytes

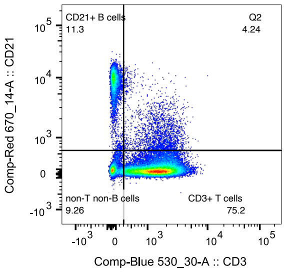

[20180801 Batch3_592_021.fcs]
Lymphocytes
57182

non-T non-B

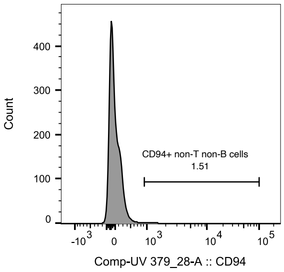

[20180801 Batch3_592_021.fcs]
non-T non-B cells
5295

592
01-AUG-2018

T cells

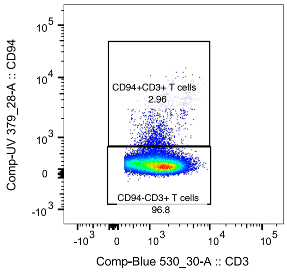

[20180801 Batch3_592_021.fcs]
CD3+ T cells
43016

CD94- T cells

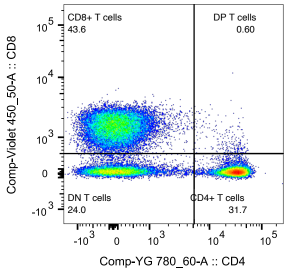

[20180801 Batch3_592_021.fcs]
CD94-CD3+ T cells
41632

CD4 T cells

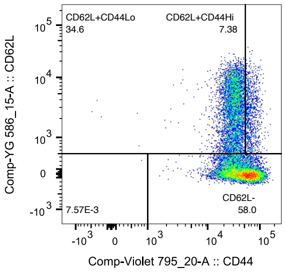

[20180801 Batch3_592_021.fcs]
CD4+ T cells
13218

CD4 T cells

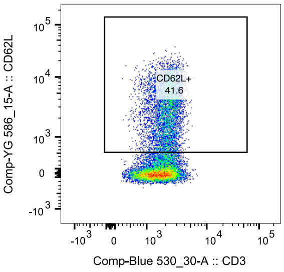

[20180801 Batch3_592_021.fcs]
CD4+ T cells
13218

CD8 T cells

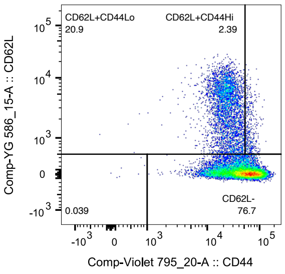

[20180801 Batch3_592_021.fcs]
CD8+ T cells
18162

CD8 T cells

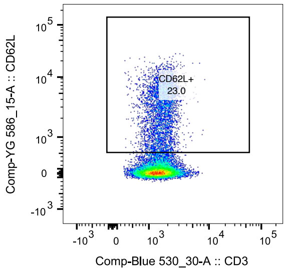

[20180801 Batch3_592_021.fcs]
CD8+ T cells
18162

DN T cells

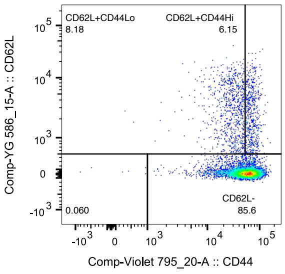

[20180801 Batch3_592_021.fcs]
DN T cells
10001

DN T cells

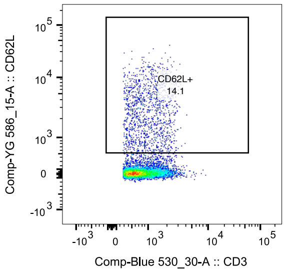

[20180801 Batch3_592_021.fcs]
DN T cells
10001

DP T cells

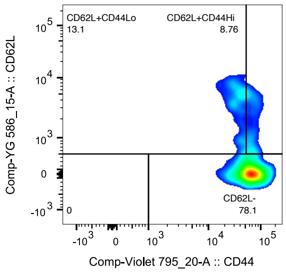

[20180801 Batch3_592_021.fcs]
DP T cells
251

DP T cells

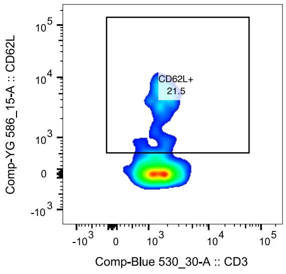

[20180801 Batch3_592_021.fcs]
DP T cells
251

614
01-AUG-2018

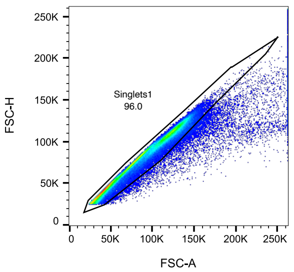

[20180801 Batch3_614_022.fcs]
Ungated
179493

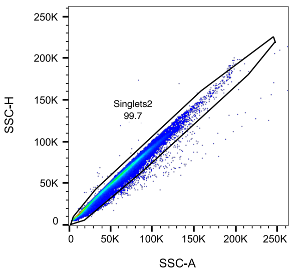

[20180801 Batch3_614_022.fcs]
Singlets1
172259

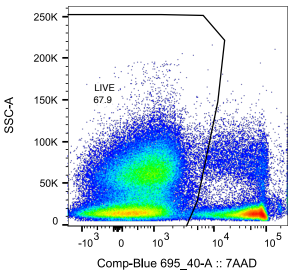

[20180801 Batch3_614_022.fcs]
Singlets2
171671

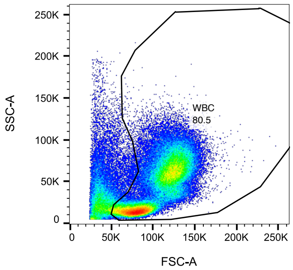

[20180801 Batch3_614_022.fcs]
LIVE
116515

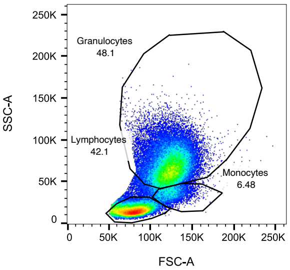

[20180801 Batch3_614_022.fcs]
WBC
93850

Granulocytes

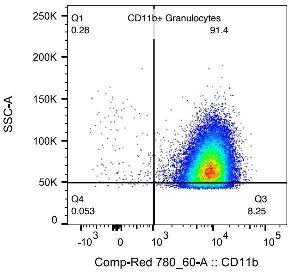

[20180801 Batch3_614_022.fcs]
Granulocytes
45114

Monocytes

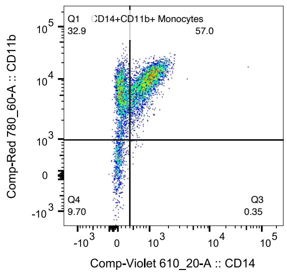

[20180801 Batch3_614_022.fcs]
Monocytes
6083

Lymphocytes

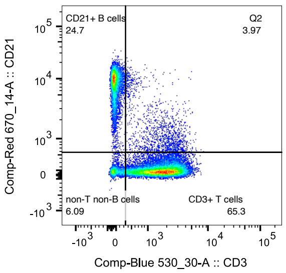

[20180801 Batch3_614_022.fcs]
Lymphocytes
39467

non-T non-B

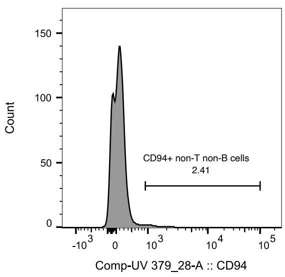

[20180801 Batch3_614_022.fcs]
non-T non-B cells
2403

614
01-AUG-2018

T cells

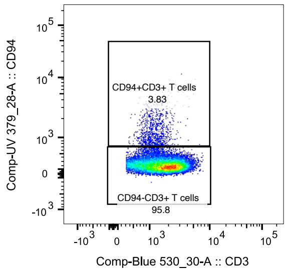

[20180801 Batch3_614_022.fcs]
CD3+ T cells
25769

CD94- T cells

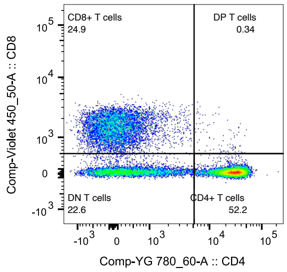

[20180801 Batch3_614_022.fcs]
CD94-CD3+ T cells
24689

CD4 T cells

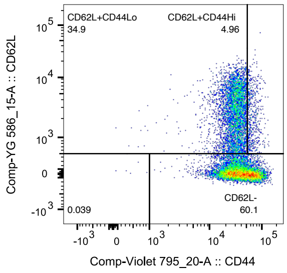

[20180801 Batch3_614_022.fcs]
CD4+ T cells
12899

CD4 T cells

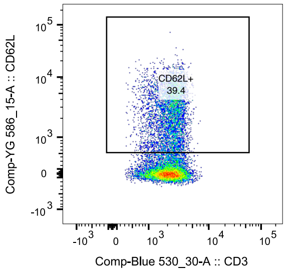

[20180801 Batch3_614_022.fcs]
CD4+ T cells
12899

CD8 T cells

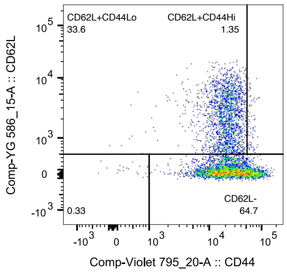

[20180801 Batch3_614_022.fcs]
CD8+ T cells
6136

CD8 T cells

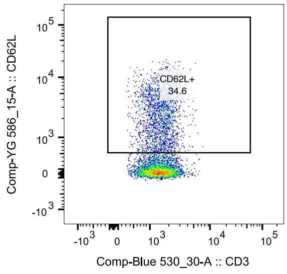

[20180801 Batch3_614_022.fcs]
CD8+ T cells
6136

DN T cells

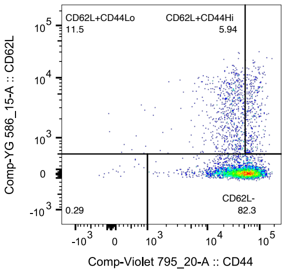

[20180801 Batch3_614_022.fcs]
DN T cells
5569

DN T cells

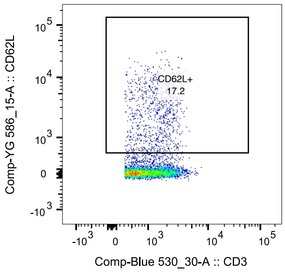

[20180801 Batch3_614_022.fcs]
DN T cells
5569

DP T cells

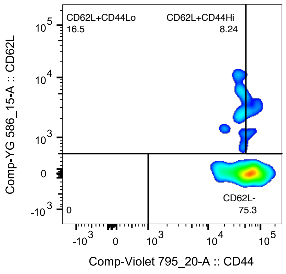

[20180801 Batch3_614_022.fcs]
DP T cells
85.0

DP T cells

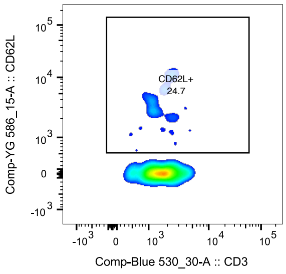

[20180801 Batch3_614_022.fcs]
DP T cells
85.0

617
01-AUG-2018

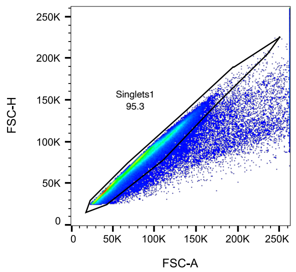

[20180801 Batch3_617_023.fcs]
Ungated
272772

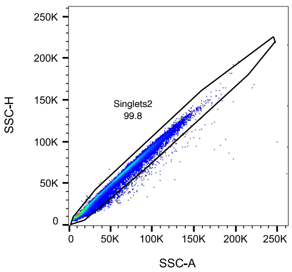

[20180801 Batch3_617_023.fcs]
Singlets1
259871

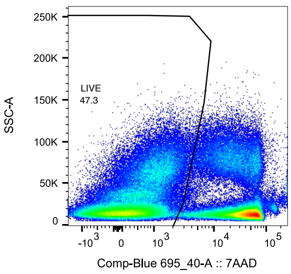

[20180801 Batch3_617_023.fcs]
Singlets2
259307

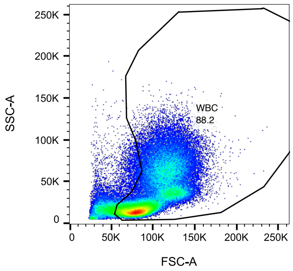

[20180801 Batch3_617_023.fcs]
LIVE
122740

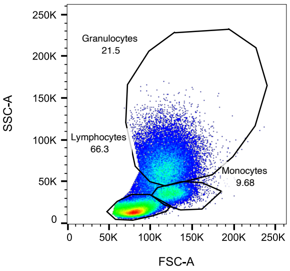

[20180801 Batch3_617_023.fcs]
WBC
108273

Granulocytes

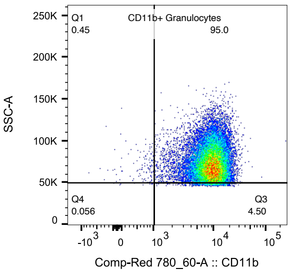

[20180801 Batch3_617_023.fcs]
Granulocytes
23274

Monocytes

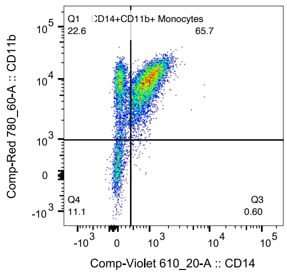

[20180801 Batch3_617_023.fcs]
Monocytes
10485

Lymphocytes

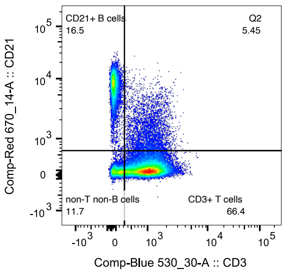

[20180801 Batch3_617_023.fcs]
Lymphocytes
71762

non-T non-B

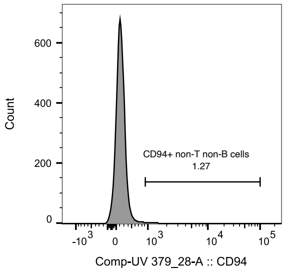

[20180801 Batch3_617_023.fcs]
non-T non-B cells
8400

617
01-AUG-2018

T cells

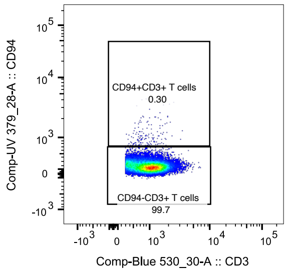

[20180801 Batch3_617_023.fcs]
CD3+ T cells
47629

CD94- T cells

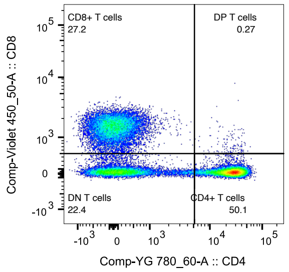

[20180801 Batch3_617_023.fcs]
CD94-CD3+ T cells
47467

CD4 T cells

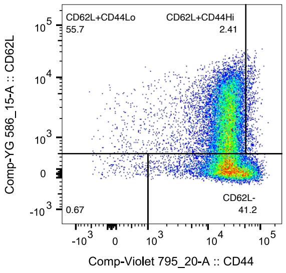

[20180801 Batch3_617_023.fcs]
CD4+ T cells
23800

CD4 T cells

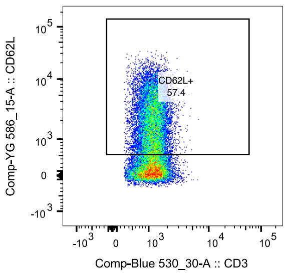

[20180801 Batch3_617_023.fcs]
CD4+ T cells
23800

CD8 T cells

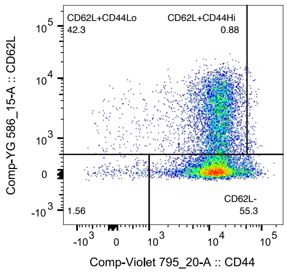

[20180801 Batch3_617_023.fcs]
CD8+ T cells
12911

CD8 T cells

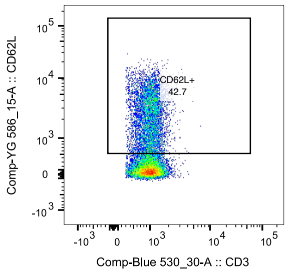

[20180801 Batch3_617_023.fcs]
CD8+ T cells
12911

DN T cells

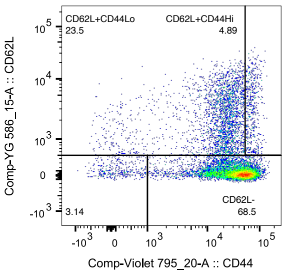

[20180801 Batch3_617_023.fcs]
DN T cells
10627

DN T cells

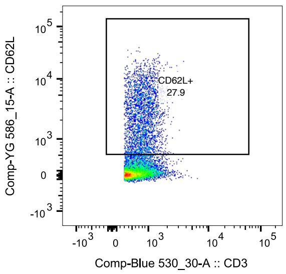

[20180801 Batch3_617_023.fcs]
DN T cells
10627

DP T cells

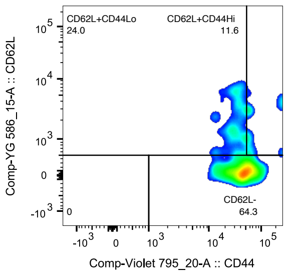

[20180801 Batch3_617_023.fcs]
DP T cells
129

DP T cells

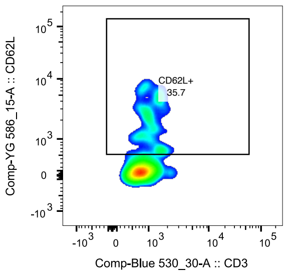

[20180801 Batch3_617_023.fcs]
DP T cells
129

627
01-AUG-2018

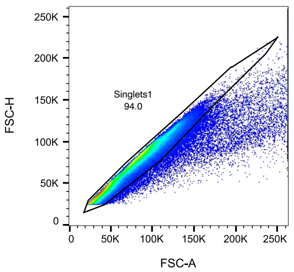

[20180801 Batch3_627_024.fcs]
Ungated
239982

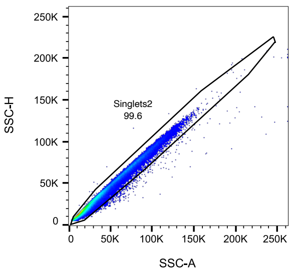

[20180801 Batch3_627_024.fcs]
Singlets1
225618

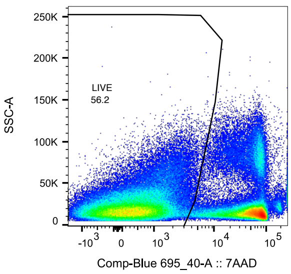

[20180801 Batch3_627_024.fcs]
Singlets2
224772

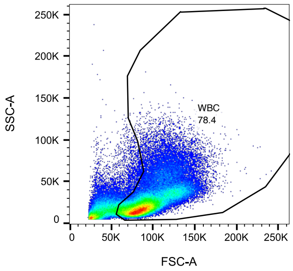

[20180801 Batch3_627_024.fcs]
LIVE
126319

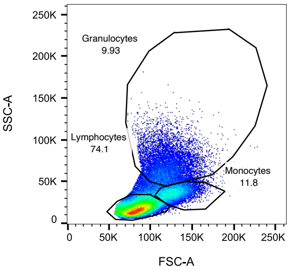

[20180801 Batch3_627_024.fcs]
WBC
99036

Granulocytes

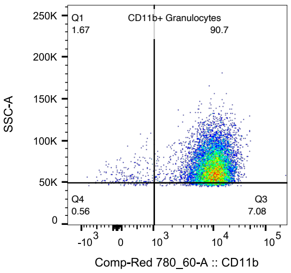

[20180801 Batch3_627_024.fcs]
Granulocytes
9838

Monocytes

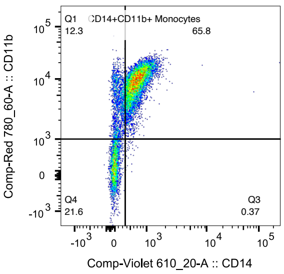

[20180801 Batch3_627_024.fcs]
Monocytes
11712

Lymphocytes

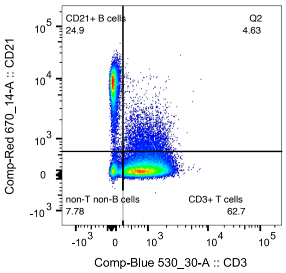

[20180801 Batch3_627_024.fcs]
Lymphocytes
73354

non-T non-B

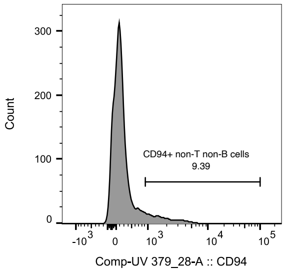

[20180801 Batch3_627_024.fcs]
non-T non-B cells
5707

627
01-AUG-2018

T cells

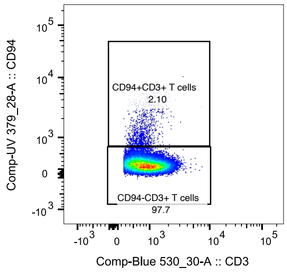

[20180801 Batch3_627_024.fcs]
CD3+ T cells
46003

CD94- T cells

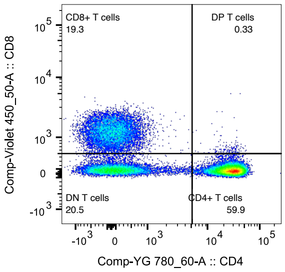

[20180801 Batch3_627_024.fcs]
CD94-CD3+ T cells
44944

CD4 T cells

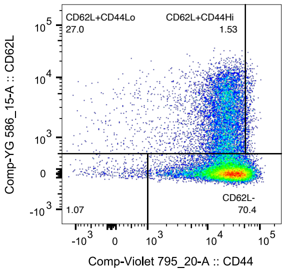

[20180801 Batch3_627_024.fcs]
CD4+ T cells
26912

CD4 T cells

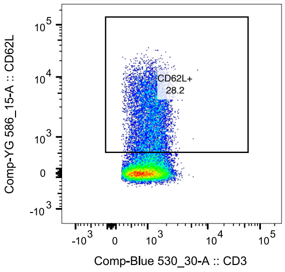

[20180801 Batch3_627_024.fcs]
CD4+ T cells
26912

CD8 T cells

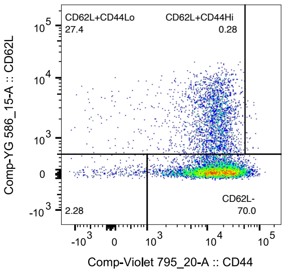

[20180801 Batch3_627_024.fcs]
CD8+ T cells
8669

CD8 T cells

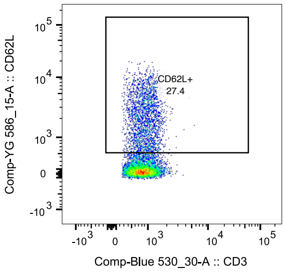

[20180801 Batch3_627_024.fcs]
CD8+ T cells
8669

DN T cells

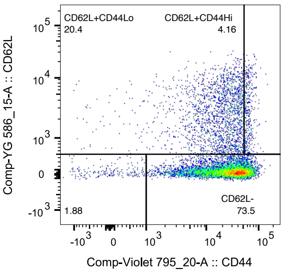

[20180801 Batch3_627_024.fcs]
DN T cells
9215

DN T cells

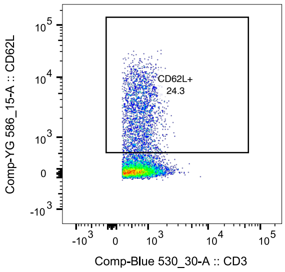

[20180801 Batch3_627_024.fcs]
DN T cells
9215

DP T cells

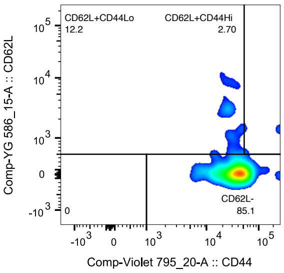

[20180801 Batch3_627_024.fcs]
DP T cells
148

DP T cells

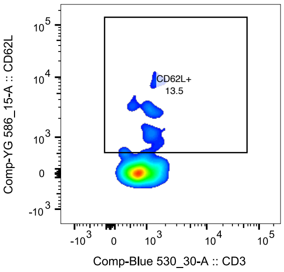

[20180801 Batch3_627_024.fcs]
DP T cells
148

619
06-AUG-2018

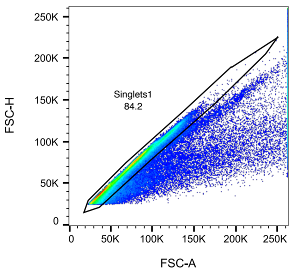

[20180806 Batch4_619_028.fcs]
Ungated
148204

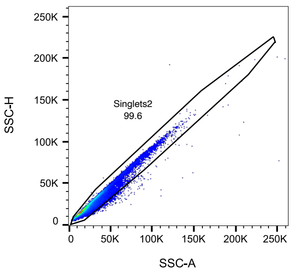

[20180806 Batch4_619_028.fcs]
Singlets1
124788

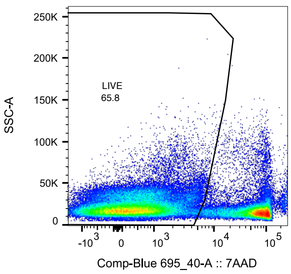

[20180806 Batch4_619_028.fcs]
Singlets2
124294

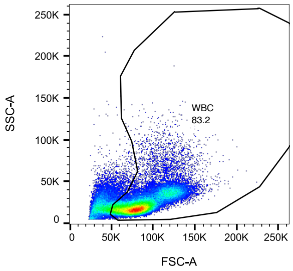

[20180806 Batch4_619_028.fcs]
LIVE
81771

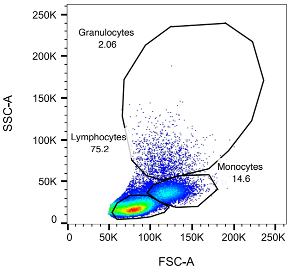

[20180806 Batch4_619_028.fcs]
WBC
68040

Granulocytes

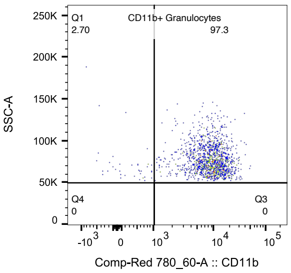

[20180806 Batch4_619_028.fcs]
Granulocytes
1405

Monocytes

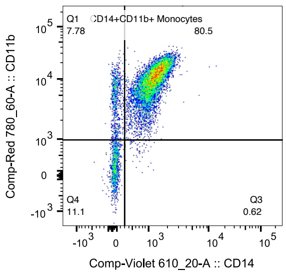

[20180806 Batch4_619_028.fcs]
Monocytes
9966

Lymphocytes

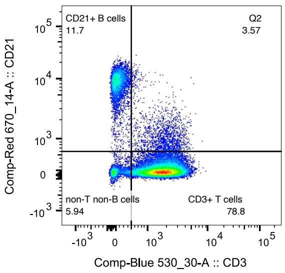

[20180806 Batch4_619_028.fcs]
Lymphocytes
51188

non-T non-B

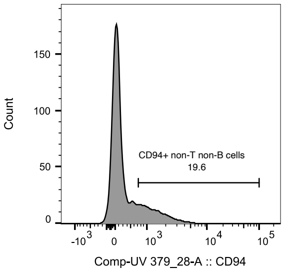

[20180806 Batch4_619_028.fcs]
non-T non-B cells
3039

619
06-AUG-2018

T cells

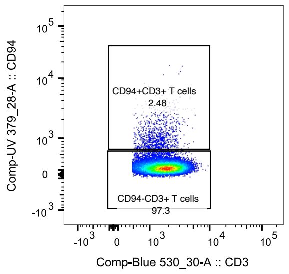

[20180806 Batch4_619_028.fcs]
CD3+ T cells
40333

CD94- T cells

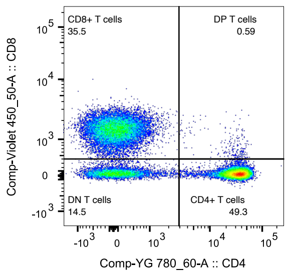

[20180806 Batch4_619_028.fcs]
CD94-CD3+ T cells
39235

CD4 T cells

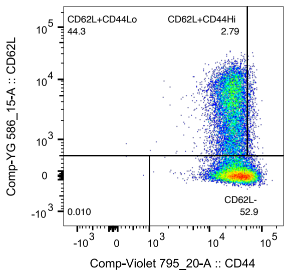

[20180806 Batch4_619_028.fcs]
CD4+ T cells
19359

CD4 T cells

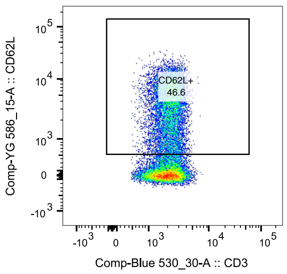

[20180806 Batch4_619_028.fcs]
CD4+ T cells
19359

CD8 T cells

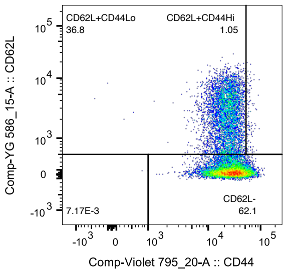

[20180806 Batch4_619_028.fcs]
CD8+ T cells
13942

CD8 T cells

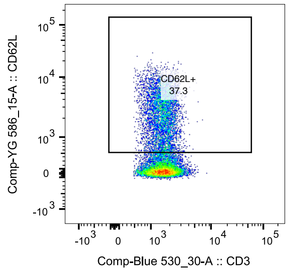

[20180806 Batch4_619_028.fcs]
CD8+ T cells
13942

DN T cells

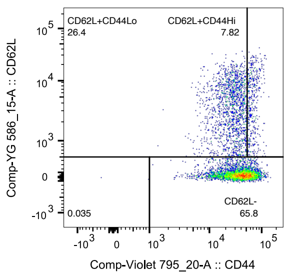

[20180806 Batch4_619_028.fcs]
DN T cells
5704

DN T cells

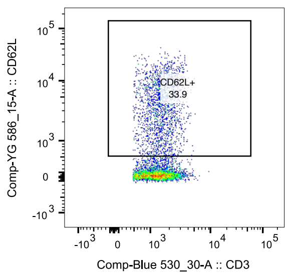

[20180806 Batch4_619_028.fcs]
DN T cells
5704

DP T cells

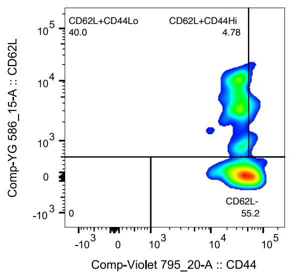

[20180806 Batch4_619_028.fcs]
DP T cells
230

DP T cells

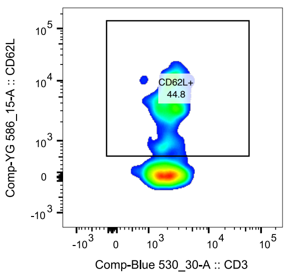

[20180806 Batch4_619_028.fcs]
DP T cells
230

527
06-AUG-2018

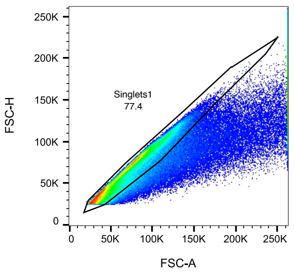

[20180806 Batch4_527_008.fcs]
Ungated
255498

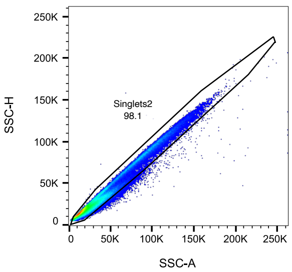

[20180806 Batch4_527_008.fcs]
Singlets1
197716

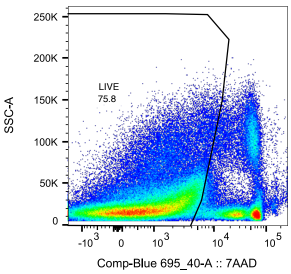

[20180806 Batch4_527_008.fcs]
Singlets2
193942

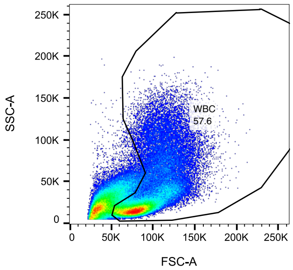

[20180806 Batch4_527_008.fcs]
LIVE
147041

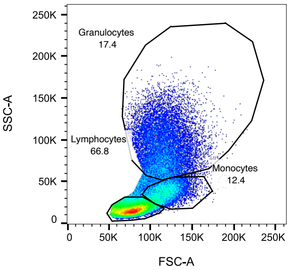

[20180806 Batch4_527_008.fcs]
WBC
84669

Granulocytes

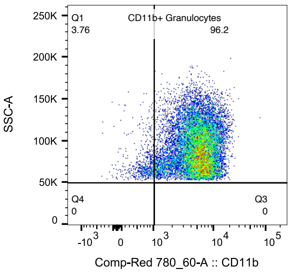

[20180806 Batch4_527_008.fcs]
Granulocytes
14723

Monocytes

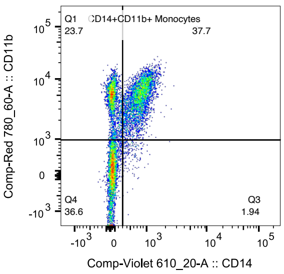

[20180806 Batch4_527_008.fcs]
Monocytes
10487

Lymphocytes

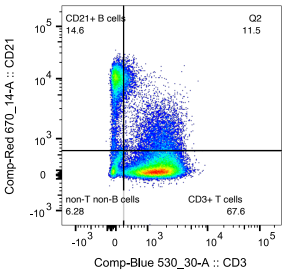

[20180806 Batch4_527_008.fcs]
Lymphocytes
56535

non-T non-B

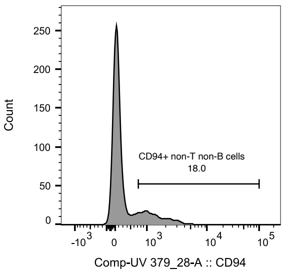

[20180806 Batch4_527_008.fcs]
non-T non-B cells
3550

527
06-AUG-2018

T cells

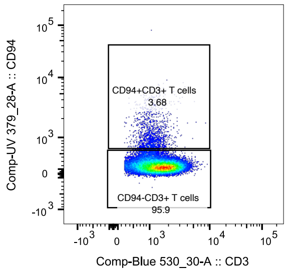

[20180806 Batch4_527_008.fcs]
CD3+ T cells
38196

CD94- T cells

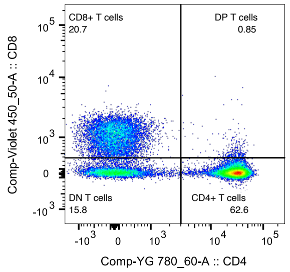

[20180806 Batch4_527_008.fcs]
CD94-CD3+ T cells
36618

CD4 T cells

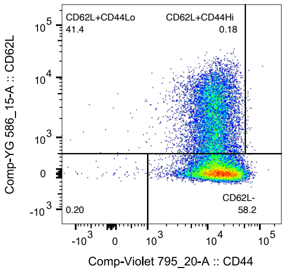

[20180806 Batch4_527_008.fcs]
CD4+ T cells
22941

CD4 T cells

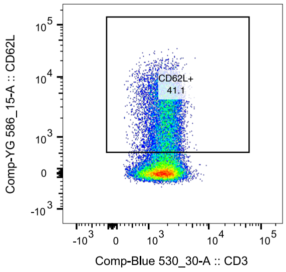

[20180806 Batch4_527_008.fcs]
CD4+ T cells
22941

CD8 T cells

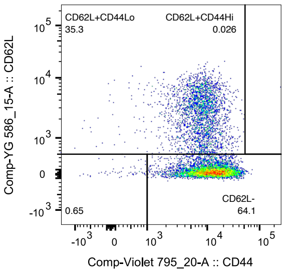

[20180806 Batch4_527_008.fcs]
CD8+ T cells
7584

CD8 T cells

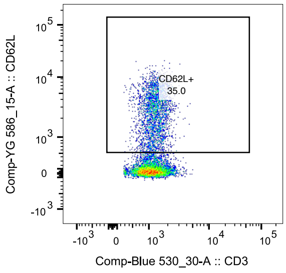

[20180806 Batch4_527_008.fcs]
CD8+ T cells
7584

DN T cells

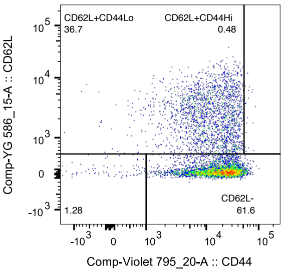

[20180806 Batch4_527_008.fcs]
DN T cells
5781

DN T cells

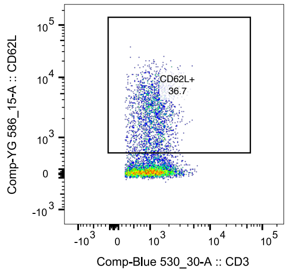

[20180806 Batch4_527_008.fcs]
DN T cells
5781

DP T cells

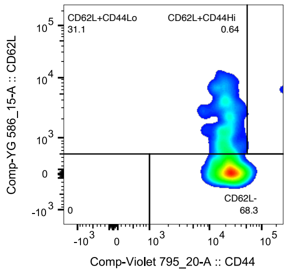

[20180806 Batch4_527_008.fcs]
DP T cells
312

DP T cells

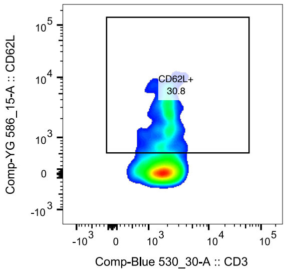

[20180806 Batch4_527_008.fcs]
DP T cells
312

509
06-AUG-2018

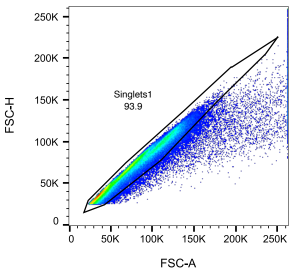

[20180806 Batch4_509_002.fcs]
Ungated
133908

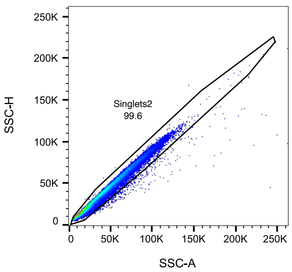

[20180806 Batch4_509_002.fcs]
Singlets1
125764

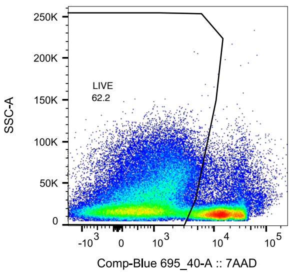

[20180806 Batch4_509_002.fcs]
Singlets2
125281

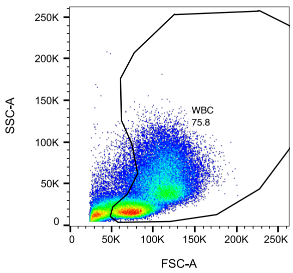

[20180806 Batch4_509_002.fcs]
LIVE
77944

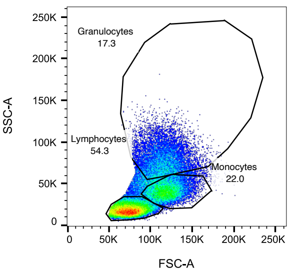

[20180806 Batch4_509_002.fcs]
WBC
59075

Granulocytes

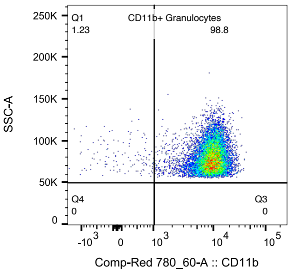

[20180806 Batch4_509_002.fcs]
Granulocytes
10200

Monocytes

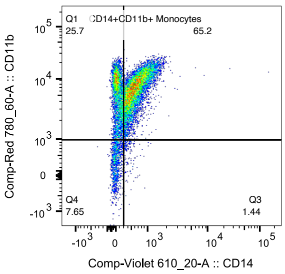

[20180806 Batch4_509_002.fcs]
Monocytes
13012

Lymphocytes

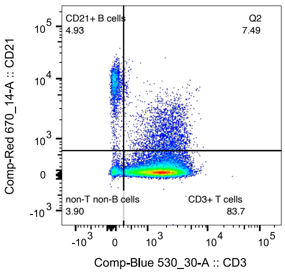

[20180806 Batch4_509_002.fcs]
Lymphocytes
32064

non-T non-B

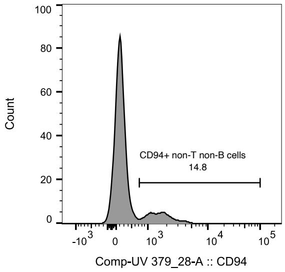

[20180806 Batch4_509_002.fcs]
non-T non-B cells
1250

509
06-AUG-2018

T cells

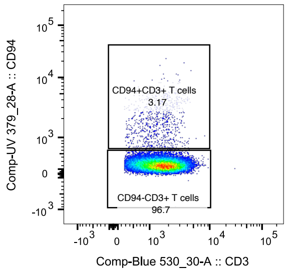

[20180806 Batch4_509_002.fcs]
CD3+ T cells
26831

CD94- T cells

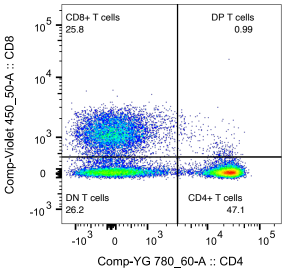

[20180806 Batch4_509_002.fcs]
CD94-CD3+ T cells
25938

CD4 T cells

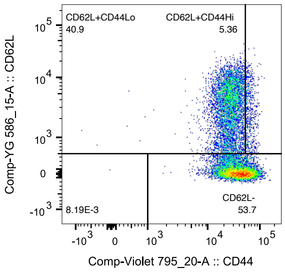

[20180806 Batch4_509_002.fcs]
CD4+ T cells
12212

CD4 T cells

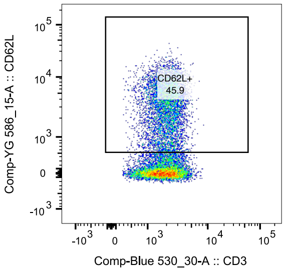

[20180806 Batch4_509_002.fcs]
CD4+ T cells
12212

CD8 T cells

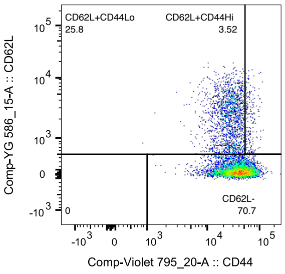

[20180806 Batch4_509_002.fcs]
CD8+ T cells
6681

CD8 T cells

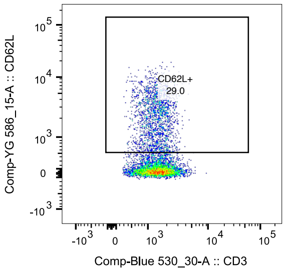

[20180806 Batch4_509_002.fcs]
CD8+ T cells
6681

DN T cells

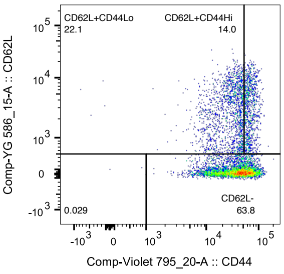

[20180806 Batch4_509_002.fcs]
DN T cells
6787

DN T cells

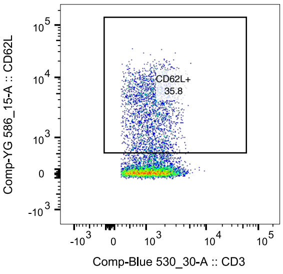

[20180806 Batch4_509_002.fcs]
DN T cells
6787

DP T cells

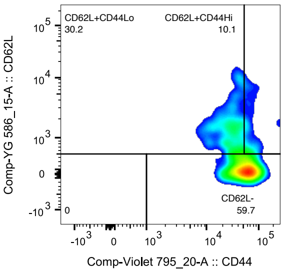

[20180806 Batch4_509_002.fcs]
DP T cells
258

DP T cells

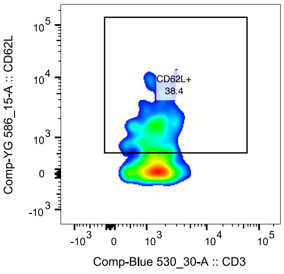

[20180806 Batch4_509_002.fcs]
DP T cells
258

515
06-AUG-2018

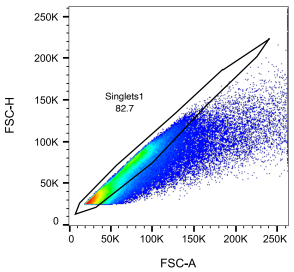

[20180806 Batch4_515_005.fcs]
Ungated
203722

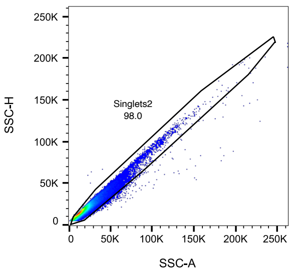

[20180806 Batch4_515_005.fcs]
Singlets1
168529

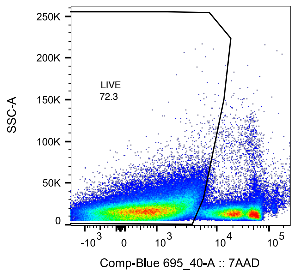

[20180806 Batch4_515_005.fcs]
Singlets2
165098

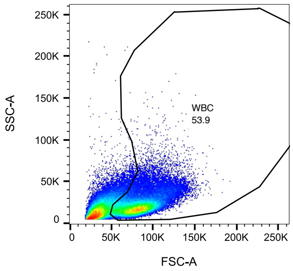

[20180806 Batch4_515_005.fcs]
LIVE
119305

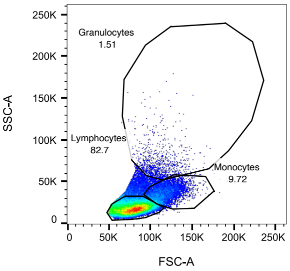

[20180806 Batch4_515_005.fcs]
WBC
64339

Granulocytes

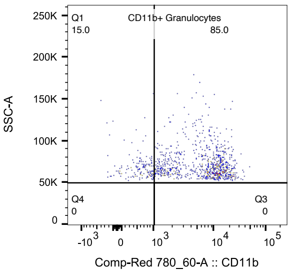

[20180806 Batch4_515_005.fcs]
Granulocytes
974

Monocytes

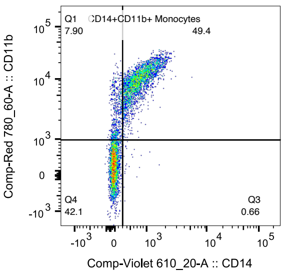

[20180806 Batch4_515_005.fcs]
Monocytes
6251

Lymphocytes

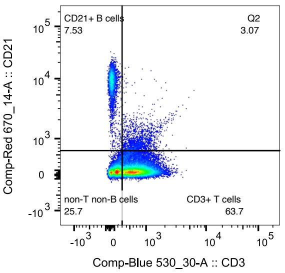

[20180806 Batch4_515_005.fcs]
Lymphocytes
53223

non-T non-B

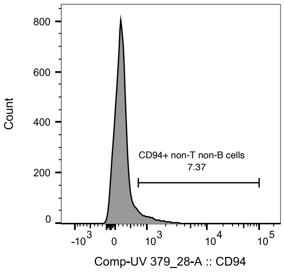

[20180806 Batch4_515_005.fcs]
non-T non-B cells
13681

515

06-AUG-2018

T cells

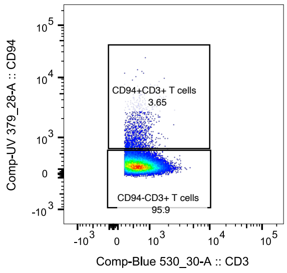

[20180806 Batch4_515_005.fcs]
CD3+ T cells
33902

CD94- T cells

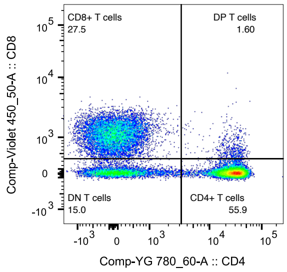

[20180806 Batch4_515_005.fcs]
CD94-CD3+ T cells
32500

CD4 T cells

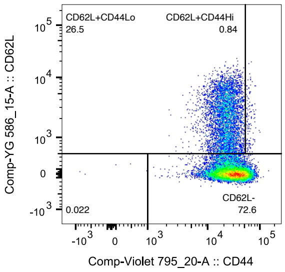

[20180806 Batch4_515_005.fcs]
CD4+ T cells
18172

CD4 T cells

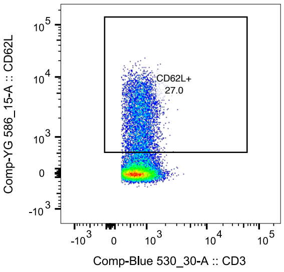

[20180806 Batch4_515_005.fcs]
CD4+ T cells
18172

CD8 T cells

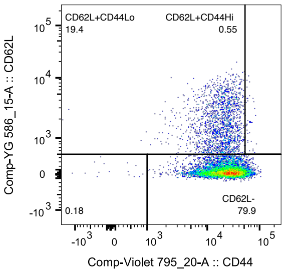

[20180806 Batch4_515_005.fcs]
CD8+ T cells
8932

CD8 T cells

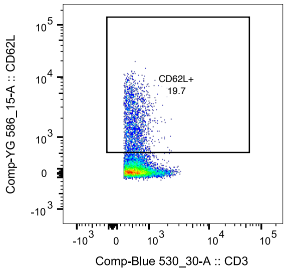

[20180806 Batch4_515_005.fcs]
CD8+ T cells
8932

DN T cells

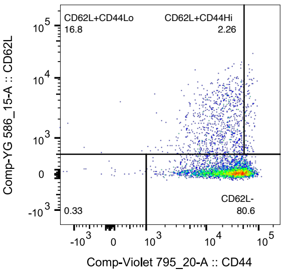

[20180806 Batch4_515_005.fcs]
DN T cells
4877

DN T cells

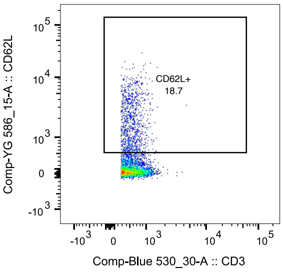

[20180806 Batch4_515_005.fcs]
DN T cells
4877

DP T cells

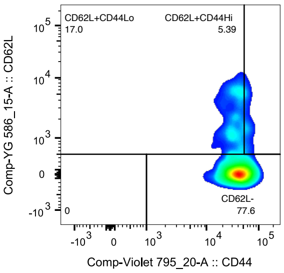

[20180806 Batch4_515_005.fcs]
DP T cells
519

DP T cells

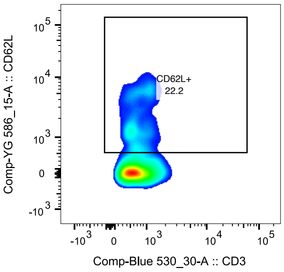

[20180806 Batch4_515_005.fcs]
DP T cells
519

535
06-AUG-2018

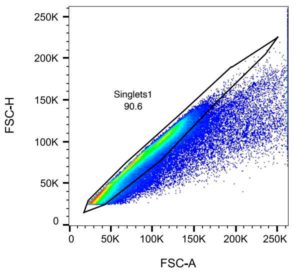

[20180806 Batch4_535_012.fcs]
Ungated
202065

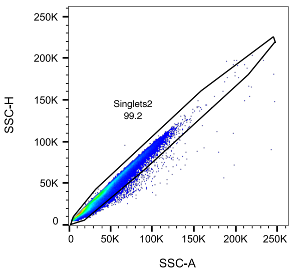

[20180806 Batch4_535_012.fcs]
Singlets1
183085

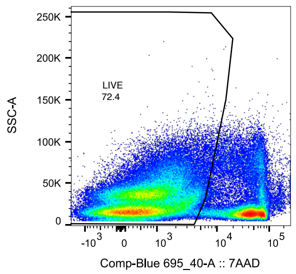

[20180806 Batch4_535_012.fcs]
Singlets2
181591

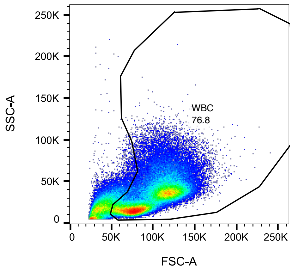

[20180806 Batch4_535_012.fcs]
LIVE
131503

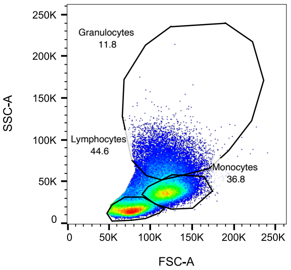

[20180806 Batch4_535_012.fcs]
WBC
100955

Granulocytes

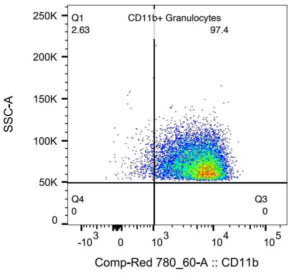

[20180806 Batch4_535_012.fcs]
Granulocytes
11894

Monocytes

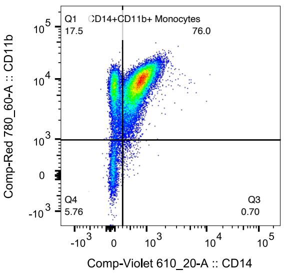

[20180806 Batch4_535_012.fcs]
Monocytes
37113

Lymphocytes

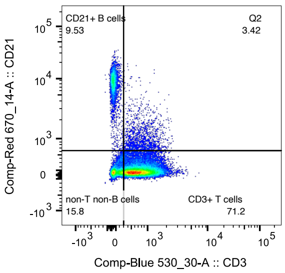

[20180806 Batch4_535_012.fcs]
Lymphocytes
45046

non-T non-B

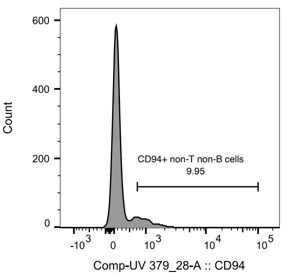

[20180806 Batch4_535_012.fcs]
non-T non-B cells
7127

535
06-AUG-2018

T cells

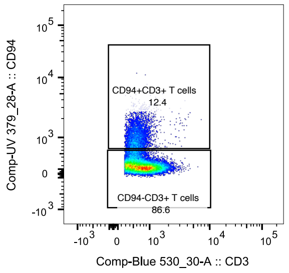

[20180806 Batch4_535_012.fcs]
CD3+ T cells
32085

CD94- T cells

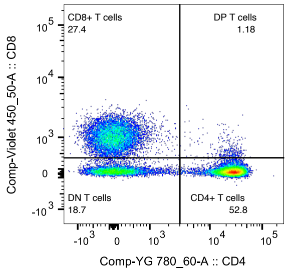

[20180806 Batch4_535_012.fcs]
CD94-CD3+ T cells
27790

CD4 T cells

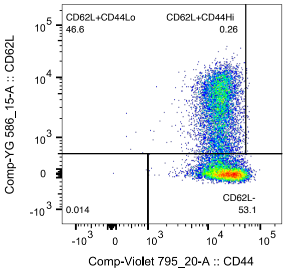

[20180806 Batch4_535_012.fcs]
CD4+ T cells
14663

CD4 T cells

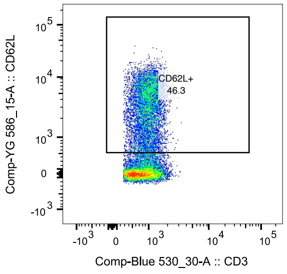

[20180806 Batch4_535_012.fcs]
CD4+ T cells
14663

CD8 T cells

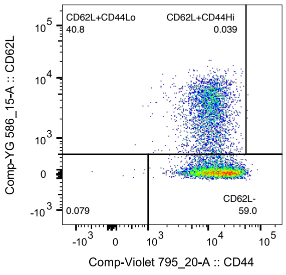

[20180806 Batch4_535_012.fcs]
CD8+ T cells
7606

CD8 T cells

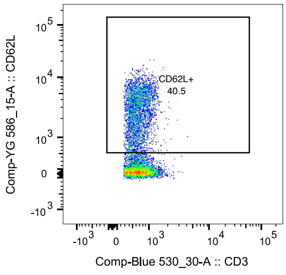

[20180806 Batch4_535_012.fcs]
CD8+ T cells
7606

DN T cells

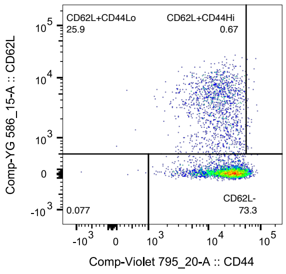

[20180806 Batch4_535_012.fcs]
DN T cells
5194

DN T cells

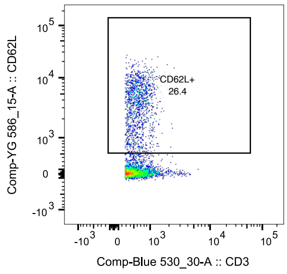

[20180806 Batch4_535_012.fcs]
DN T cells
5194

DP T cells

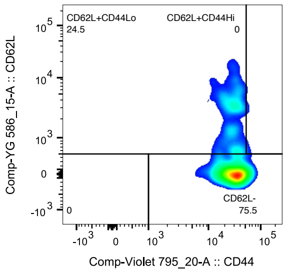

[20180806 Batch4_535_012.fcs]
DP T cells
327

DP T cells

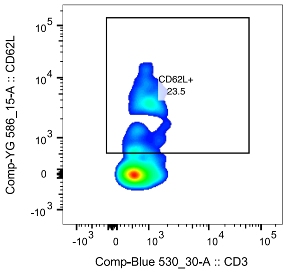

[20180806 Batch4_535_012.fcs]
DP T cells
327

572
06-AUG-2018

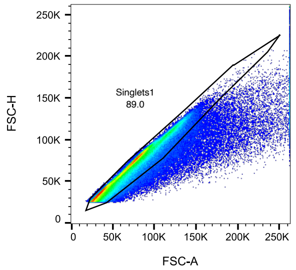

[20180806 Batch4_572_015.fcs]
Ungated
213114

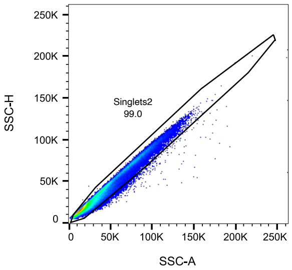

[20180806 Batch4_572_015.fcs]
Singlets1
189636

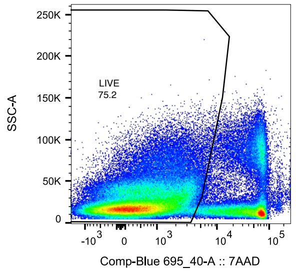

[20180806 Batch4_572_015.fcs]
Singlets2
187787

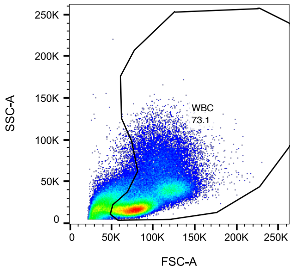

[20180806 Batch4_572_015.fcs]
LIVE
141166

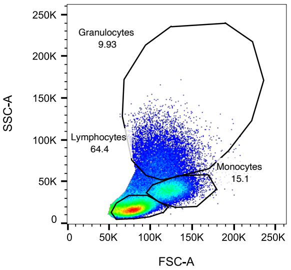

[20180806 Batch4_572_015.fcs]
WBC
103248

Granulocytes

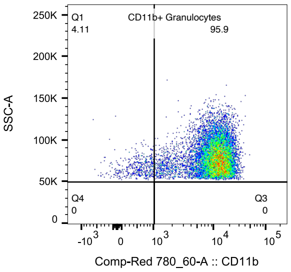

[20180806 Batch4_572_015.fcs]
Granulocytes
10249

Monocytes

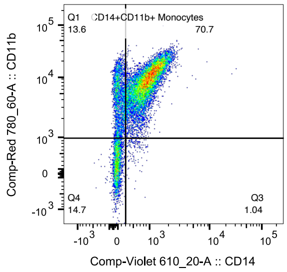

[20180806 Batch4_572_015.fcs]
Monocytes
15614

Lymphocytes

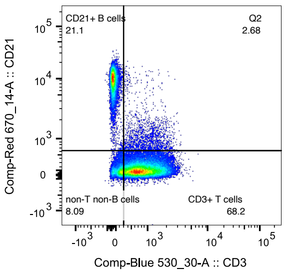

[20180806 Batch4_572_015.fcs]
Lymphocytes
66512

non-T non-B

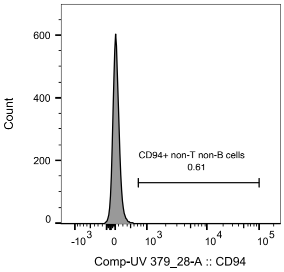

[20180806 Batch4_572_015.fcs]
non-T non-B cells
5381

572
06-AUG-2018

T cells

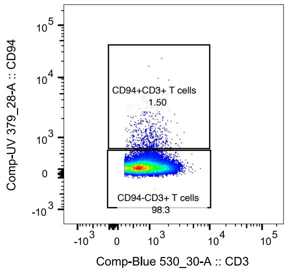

[20180806 Batch4_572_015.fcs]
CD3+ T cells
45333

CD94- T cells

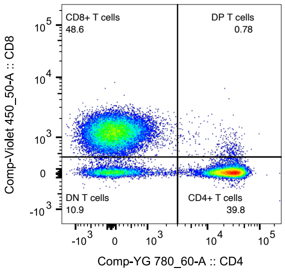

[20180806 Batch4_572_015.fcs]
CD94-CD3+ T cells
44546

CD4 T cells

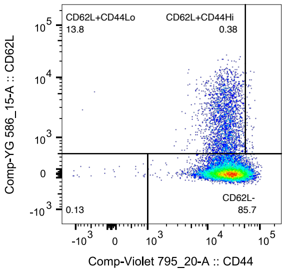

[20180806 Batch4_572_015.fcs]
CD4+ T cells
17721

CD4 T cells

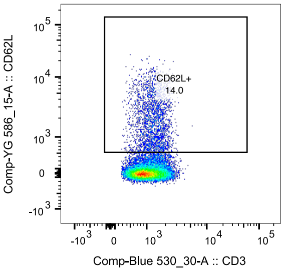

[20180806 Batch4_572_015.fcs]
CD4+ T cells
17721

CD8 T cells

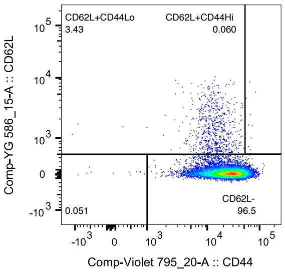

[20180806 Batch4_572_015.fcs]
CD8+ T cells
21634

CD8 T cells

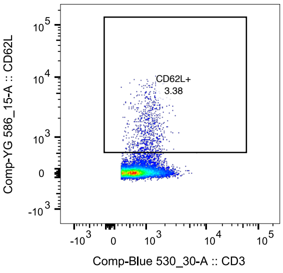

[20180806 Batch4_572_015.fcs]
CD8+ T cells
21634

DN T cells

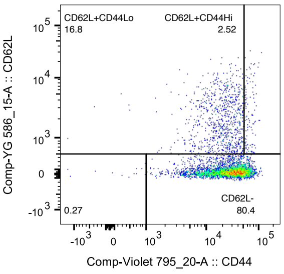

[20180806 Batch4_572_015.fcs]
DN T cells
4842

DN T cells

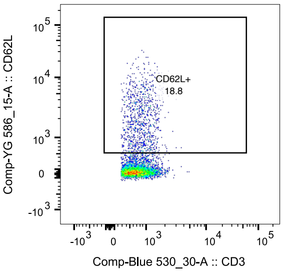

[20180806 Batch4_572_015.fcs]
DN T cells
4842

DP T cells

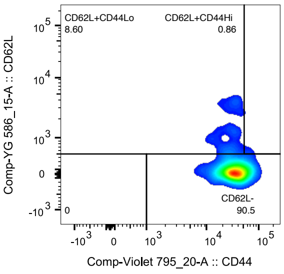

[20180806 Batch4_572_015.fcs]
DP T cells
349

DP T cells

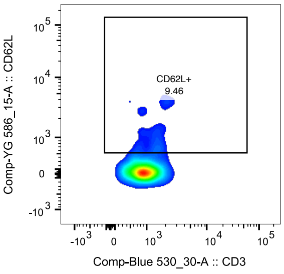

[20180806 Batch4_572_015.fcs]
DP T cells
349

561
06-AUG-2018

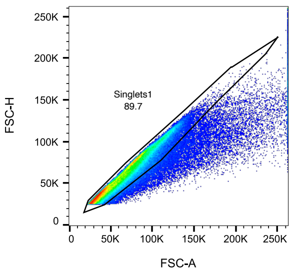

[20180806 Batch4_561_014.fcs]
Ungated
156350

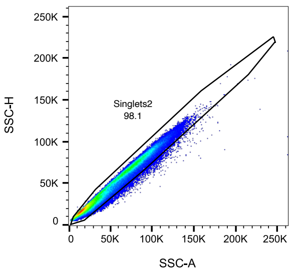

[20180806 Batch4_561_014.fcs]
Singlets1
140253

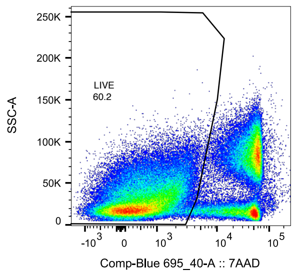

[20180806 Batch4_561_014.fcs]
Singlets2
137649

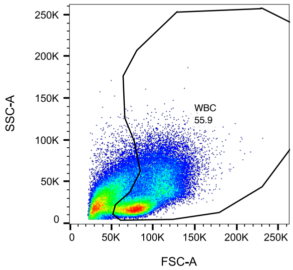

[20180806 Batch4_561_014.fcs]
LIVE
82879

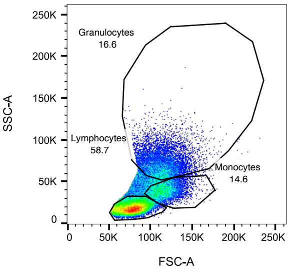

[20180806 Batch4_561_014.fcs]
WBC
46301

Granulocytes

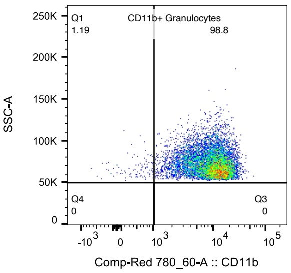

[20180806 Batch4_561_014.fcs]
Granulocytes
7700

Monocytes

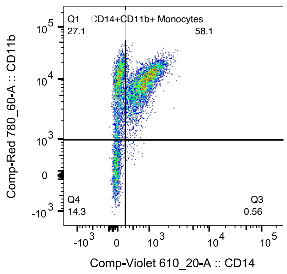

[20180806 Batch4_561_014.fcs]
Monocytes
6759

Lymphocytes

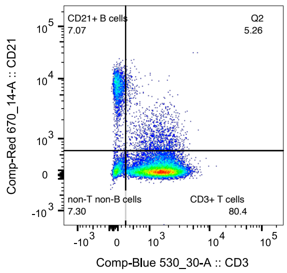

[20180806 Batch4_561_014.fcs]
Lymphocytes
27166

non-T non-B

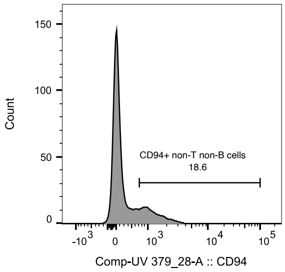

[20180806 Batch4_561_014.fcs]
non-T non-B cells
1984

561
06-AUG-2018

T cells

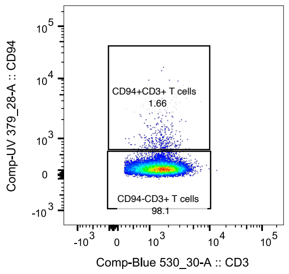

[20180806 Batch4_561_014.fcs]
CD3+ T cells
21834

CD94- T cells

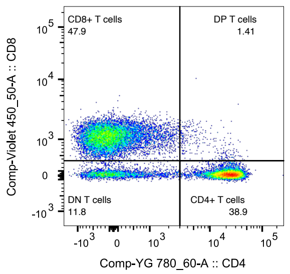

[20180806 Batch4_561_014.fcs]
CD94-CD3+ T cells
21415

CD4 T cells

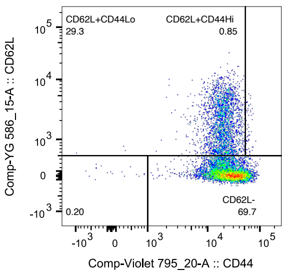

[20180806 Batch4_561_014.fcs]
CD4+ T cells
8321

CD4 T cells

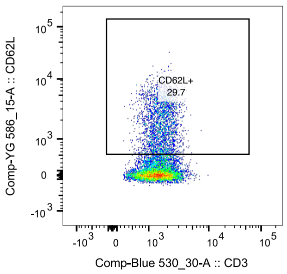

[20180806 Batch4_561_014.fcs]
CD4+ T cells
8321

CD8 T cells

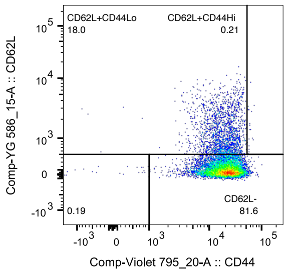

[20180806 Batch4_561_014.fcs]
CD8+ T cells
10267

CD8 T cells

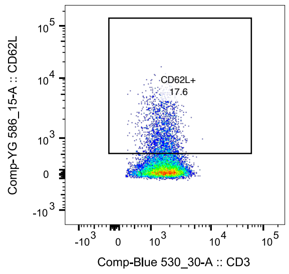

[20180806 Batch4_561_014.fcs]
CD8+ T cells
10267

DN T cells

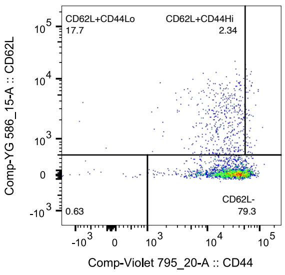

[20180806 Batch4_561_014.fcs]
DN T cells
2525

DN T cells

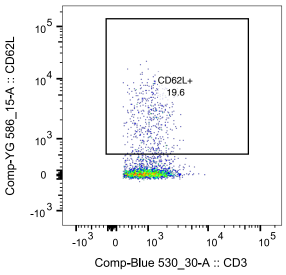

[20180806 Batch4_561_014.fcs]
DN T cells
2525

DP T cells

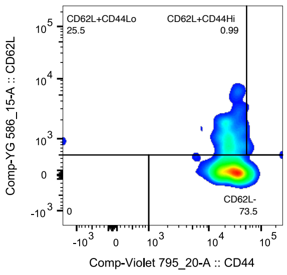

[20180806 Batch4_561_014.fcs]
DP T cells
302

DP T cells

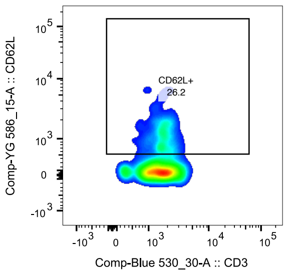

[20180806 Batch4_561_014.fcs]
DP T cells
302

512
06-AUG-2018

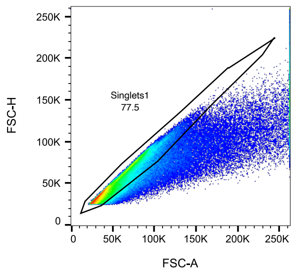

[20180806 Batch4_512_003.fcs]
Ungated
199044

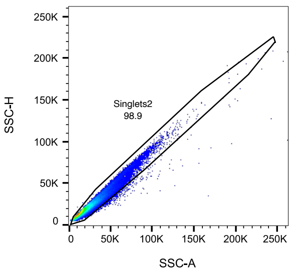

[20180806 Batch4_512_003.fcs]
Singlets1
154272

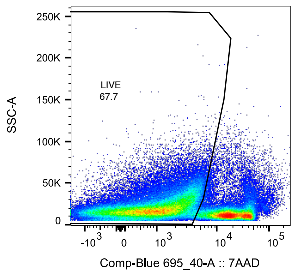

[20180806 Batch4_512_003.fcs]
Singlets2
152540

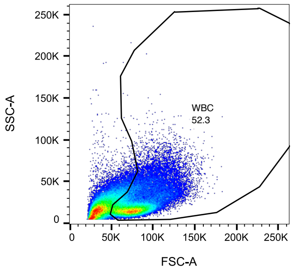

[20180806 Batch4_512_003.fcs]
LIVE
103303

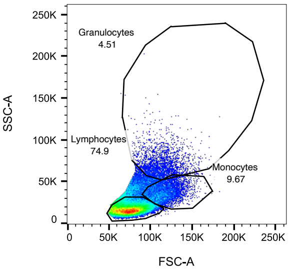

[20180806 Batch4_512_003.fcs]
WBC
53991

Granulocytes

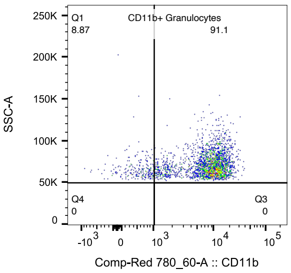

[20180806 Batch4_512_003.fcs]
Granulocytes
2436

Monocytes

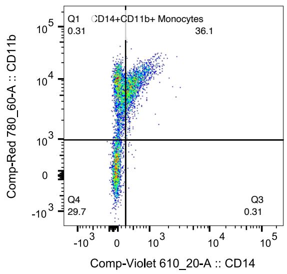

[20180806 Batch4_512_003.fcs]
Monocytes
5220

Lymphocytes

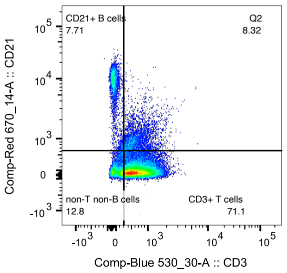

[20180806 Batch4_512_003.fcs]
Lymphocytes
40451

non-T non-B

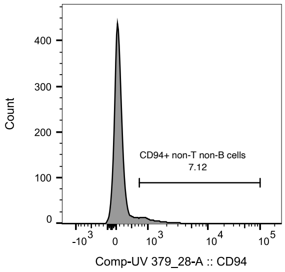

[20180806 Batch4_512_003.fcs]
non-T non-B cells
5197

512
06-AUG-2018

T cells

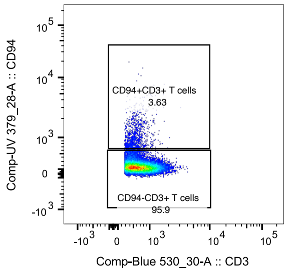

[20180806 Batch4_512_003.fcs]
CD3+ T cells
28769

CD94- T cells

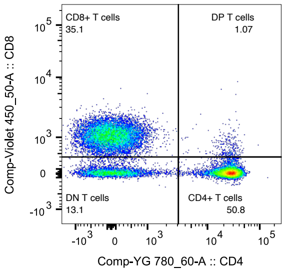

[20180806 Batch4_512_003.fcs]
CD94-CD3+ T cells
27597

CD4 T cells

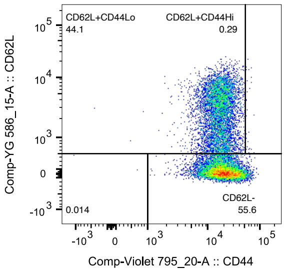

[20180806 Batch4_512_003.fcs]
CD4+ T cells
14024

CD4 T cells

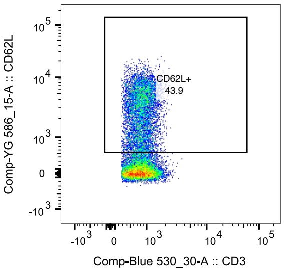

[20180806 Batch4_512_003.fcs]
CD4+ T cells
14024

CD8 T cells

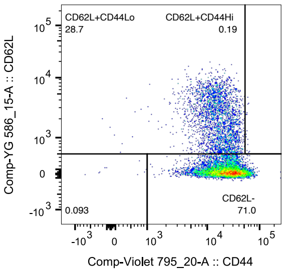

[20180806 Batch4_512_003.fcs]
CD8+ T cells
9674

CD8 T cells

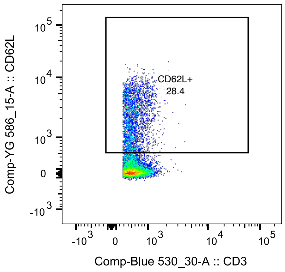

[20180806 Batch4_512_003.fcs]
CD8+ T cells
9674

DN T cells

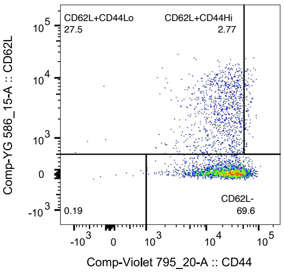

[20180806 Batch4_512_003.fcs]
DN T cells
3604

DN T cells

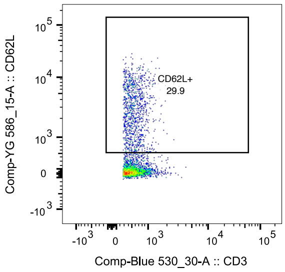

[20180806 Batch4_512_003.fcs]
DN T cells
3604

DP T cells

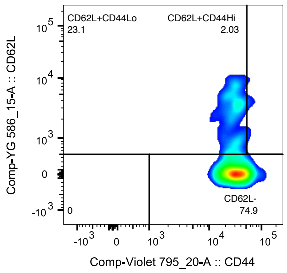

[20180806 Batch4_512_003.fcs]
DP T cells
295

DP T cells

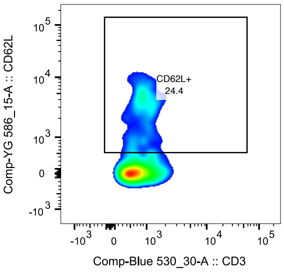

[20180806 Batch4_512_003.fcs]
DP T cells
295

514
06-AUG-2018

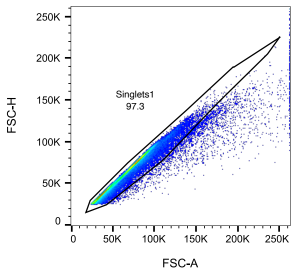

[20180806 Batch4_514_004.fcs]
Ungated
99168

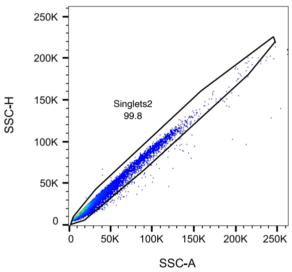

[20180806 Batch4_514_004.fcs]
Singlets1
96528

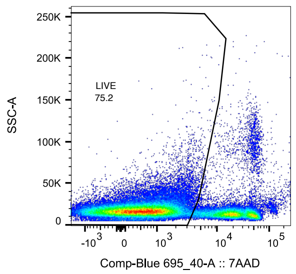

[20180806 Batch4_514_004.fcs]
Singlets2
96362

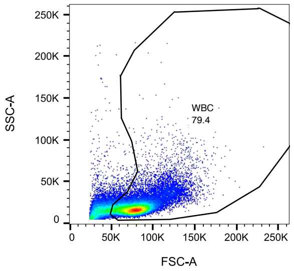

[20180806 Batch4_514_004.fcs]
LIVE
72500

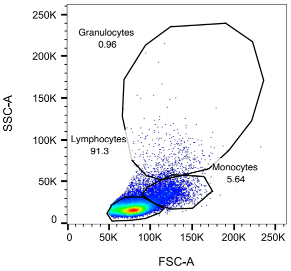

[20180806 Batch4_514_004.fcs]
WBC
57575

Granulocytes

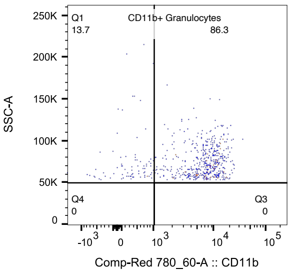

[20180806 Batch4_514_004.fcs]
Granulocytes
555

Monocytes

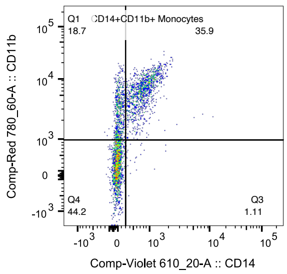

[20180806 Batch4_514_004.fcs]
Monocytes
3250

Lymphocytes

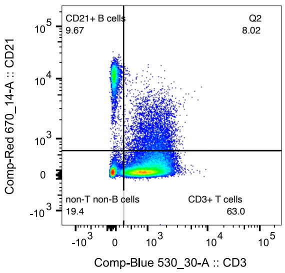

[20180806 Batch4_514_004.fcs]
Lymphocytes
52594

non-T non-B

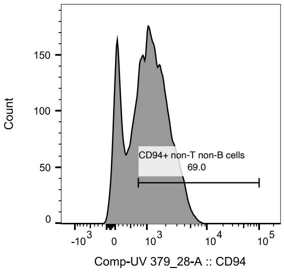

[20180806 Batch4_514_004.fcs]
non-T non-B cells
10177

514
06-AUG-2018

T cells

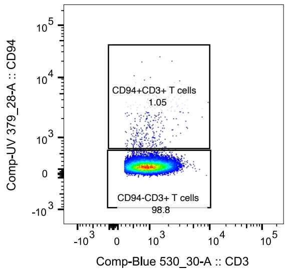

[20180806 Batch4_514_004.fcs]
CD3+ T cells
33116

CD94- T cells

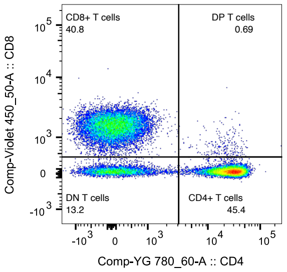

[20180806 Batch4_514_004.fcs]
CD94-CD3+ T cells
32724

CD4 T cells

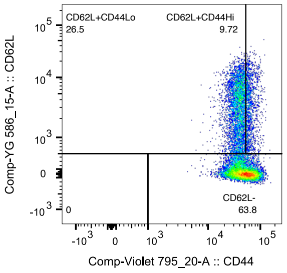

[20180806 Batch4_514_004.fcs]
CD4+ T cells
14847

CD4 T cells

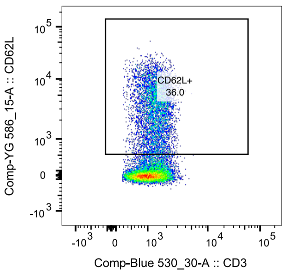

[20180806 Batch4_514_004.fcs]
CD4+ T cells
14847

CD8 T cells

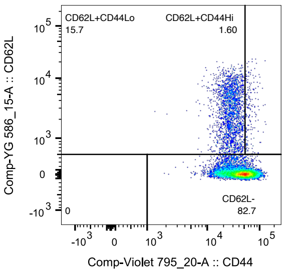

[20180806 Batch4_514_004.fcs]
CD8+ T cells
13345

CD8 T cells

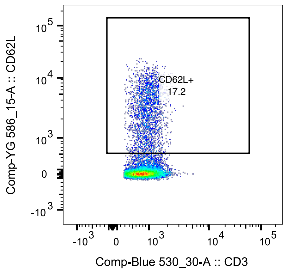

[20180806 Batch4_514_004.fcs]
CD8+ T cells
13345

DN T cells

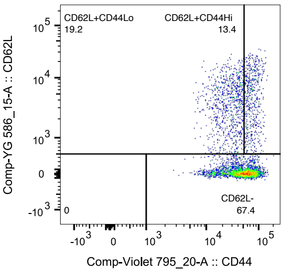

[20180806 Batch4_514_004.fcs]
DN T cells
4305

DN T cells

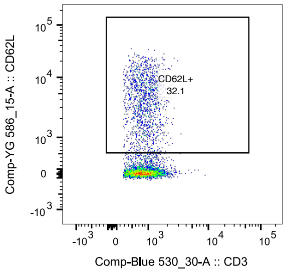

[20180806 Batch4_514_004.fcs]
DN T cells
4305

DP T cells

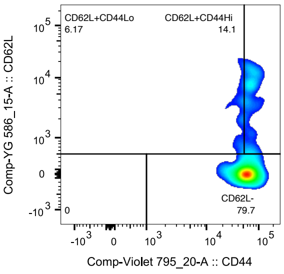

[20180806 Batch4_514_004.fcs]
DP T cells
227

DP T cells

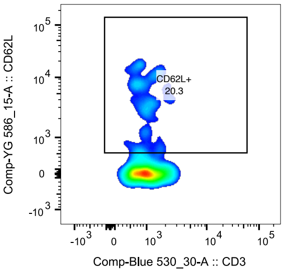

[20180806 Batch4_514_004.fcs]
DP T cells
227

618
06-AUG-2018

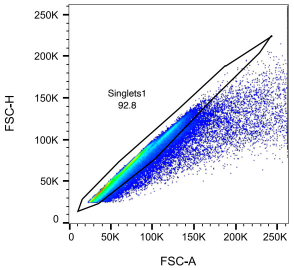

[20180806 Batch4_618_027.fcs]
Ungated
188404

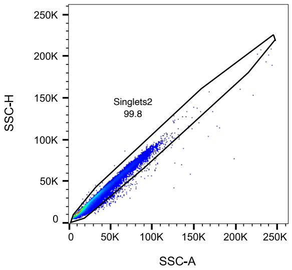

[20180806 Batch4_618_027.fcs]
Singlets1
174919

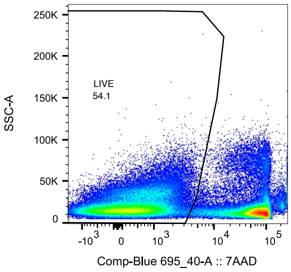

[20180806 Batch4_618_027.fcs]
Singlets2
174541

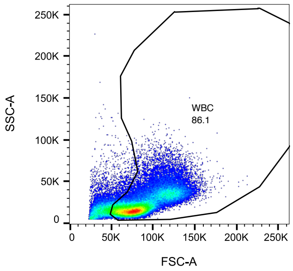

[20180806 Batch4_618_027.fcs]
LIVE
94435

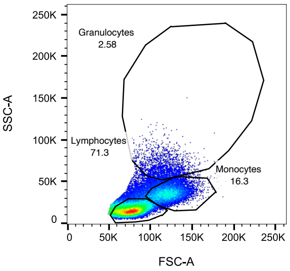

[20180806 Batch4_618_027.fcs]
WBC
81307

Granulocytes

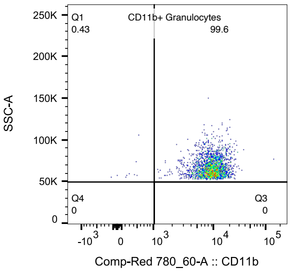

[20180806 Batch4_618_027.fcs]
Granulocytes
2097

Monocytes

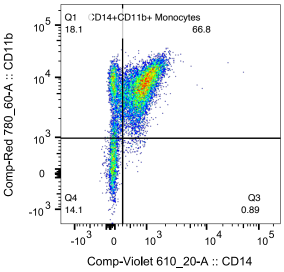

[20180806 Batch4_618_027.fcs]
Monocytes
13273

Lymphocytes

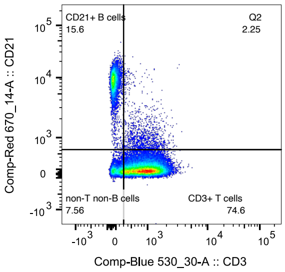

[20180806 Batch4_618_027.fcs]
Lymphocytes
57954

non-T non-B

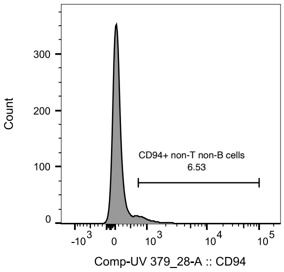

[20180806 Batch4_618_027.fcs]
non-T non-B cells
4381

618
06-AUG-2018

T cells

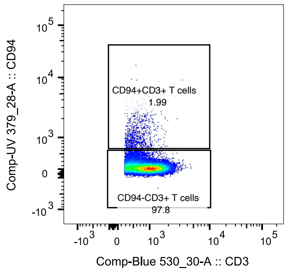

[20180806 Batch4_618_027.fcs]
CD3+ T cells
43237

CD94- T cells

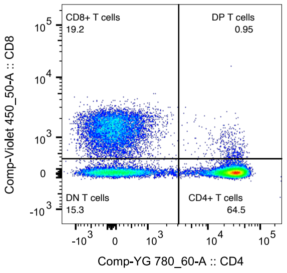

[20180806 Batch4_618_027.fcs]
CD94-CD3+ T cells
42286

CD4 T cells

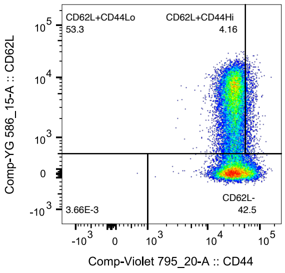

[20180806 Batch4_618_027.fcs]
CD4+ T cells
27288

CD4 T cells

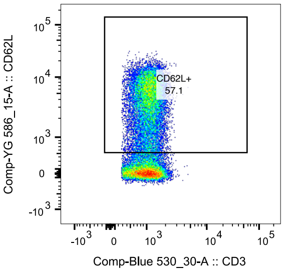

[20180806 Batch4_618_027.fcs]
CD4+ T cells
27288

CD8 T cells

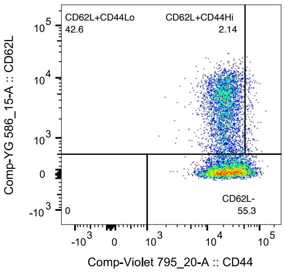

[20180806 Batch4_618_027.fcs]
CD8+ T cells
8114

CD8 T cells

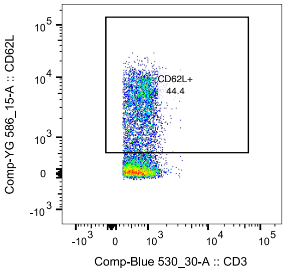

[20180806 Batch4_618_027.fcs]
CD8+ T cells
8114

DN T cells

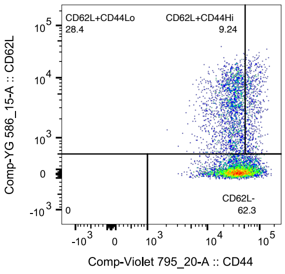

[20180806 Batch4_618_027.fcs]
DN T cells
6483

DN T cells

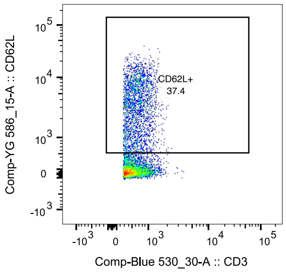

[20180806 Batch4_618_027.fcs]
DN T cells
6483

DP T cells

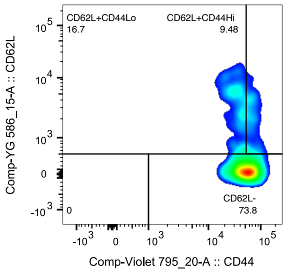

[20180806 Batch4_618_027.fcs]
DP T cells
401

DP T cells

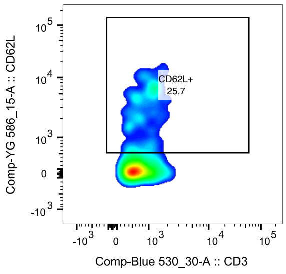

[20180806 Batch4_618_027.fcs]
DP T cells
401

615
06-AUG-2018

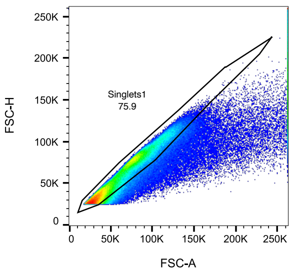

[20180806 Batch4_615_025.fcs]
Ungated
223731

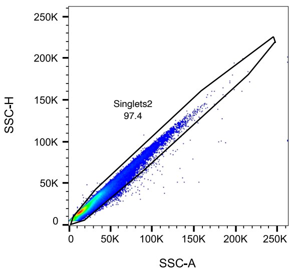

[20180806 Batch4_615_025.fcs]
Singlets1
169880

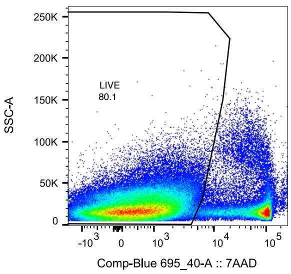

[20180806 Batch4_615_025.fcs]
Singlets2
165533

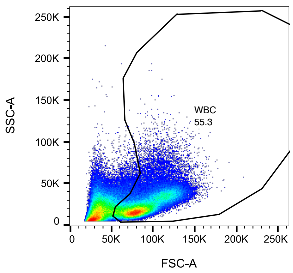

[20180806 Batch4_615_025.fcs]
LIVE
132543

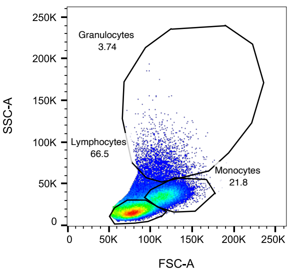

[20180806 Batch4_615_025.fcs]
WBC
73329

Granulocytes

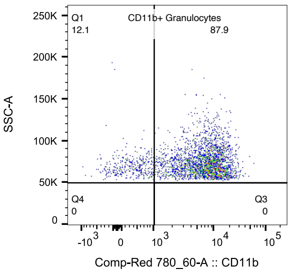

[20180806 Batch4_615_025.fcs]
Granulocytes
2739

Monocytes

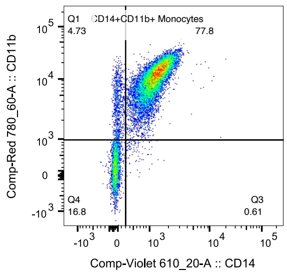

[20180806 Batch4_615_025.fcs]
Monocytes
16020

Lymphocytes

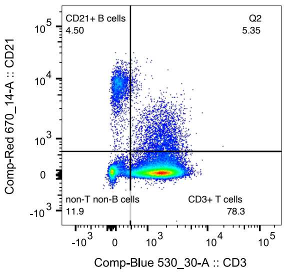

[20180806 Batch4_615_025.fcs]
Lymphocytes
48748

non-T non-B

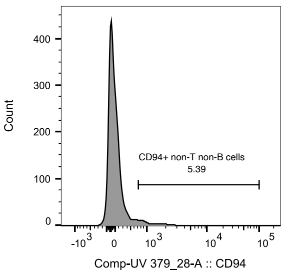

[20180806 Batch4_615_025.fcs]
non-T non-B cells
5784

615
06-AUG-2018

T cells

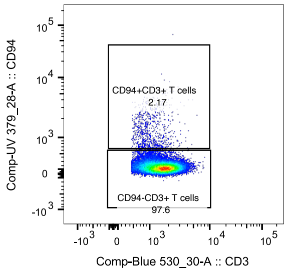

[20180806 Batch4_615_025.fcs]
CD3+ T cells
38159

CD94- T cells

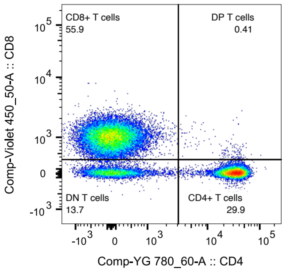

[20180806 Batch4_615_025.fcs]
CD94-CD3+ T cells
37239

CD4 T cells

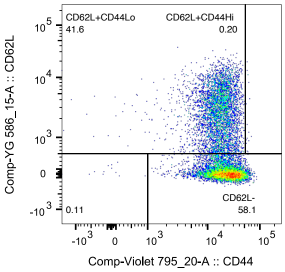

[20180806 Batch4_615_025.fcs]
CD4+ T cells
11146

CD4 T cells

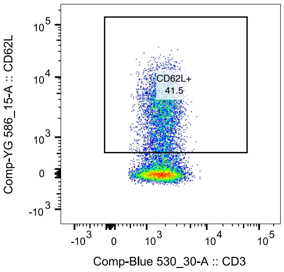

[20180806 Batch4_615_025.fcs]
CD4+ T cells
11146

CD8 T cells

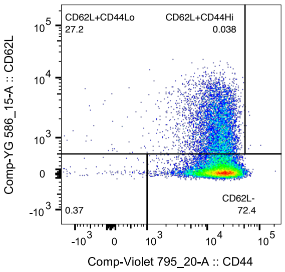

[20180806 Batch4_615_025.fcs]
CD8+ T cells
20831

CD8 T cells

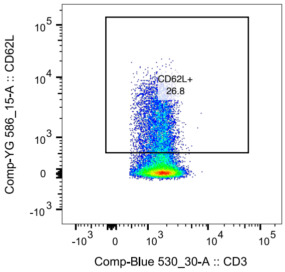

[20180806 Batch4_615_025.fcs]
CD8+ T cells
20831

DN T cells

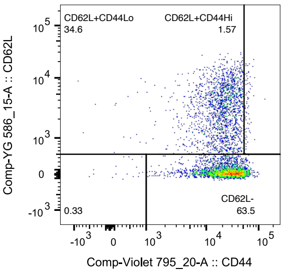

[20180806 Batch4_615_025.fcs]
DN T cells
5111

DN T cells

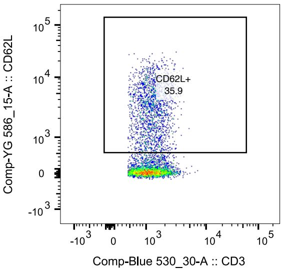

[20180806 Batch4_615_025.fcs]
DN T cells
5111

DP T cells

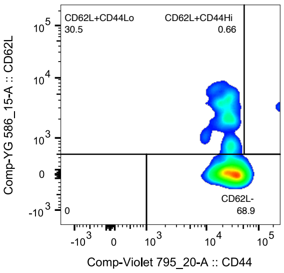

[20180806 Batch4_615_025.fcs]
DP T cells
151

DP T cells

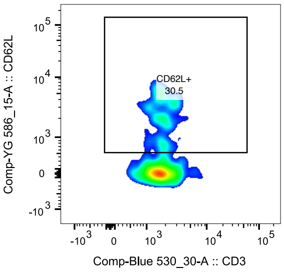

[20180806 Batch4_615_025.fcs]
DP T cells
151

612
06-AUG-2018

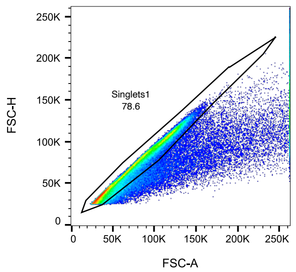

[20180806 Batch4_612_023.fcs]
Ungated
106267

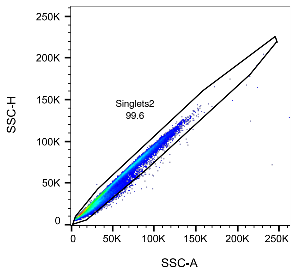

[20180806 Batch4_612_023.fcs]
Singlets1
83536

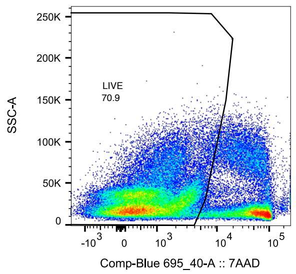

[20180806 Batch4_612_023.fcs]
Singlets2
83237

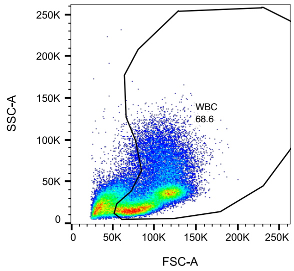

[20180806 Batch4_612_023.fcs]
LIVE
58978

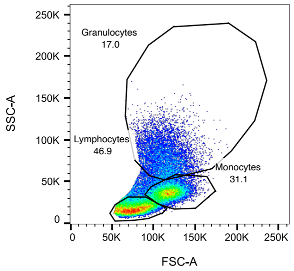

[20180806 Batch4_612_023.fcs]
WBC
40482

Granulocytes

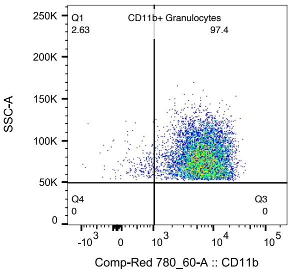

[20180806 Batch4_612_023.fcs]
Granulocytes
6882

Monocytes

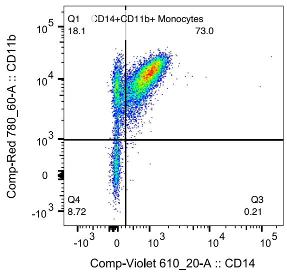

[20180806 Batch4_612_023.fcs]
Monocytes
12595

Lymphocytes

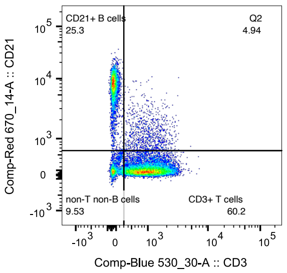

[20180806 Batch4_612_023.fcs]
Lymphocytes
18970

non-T non-B

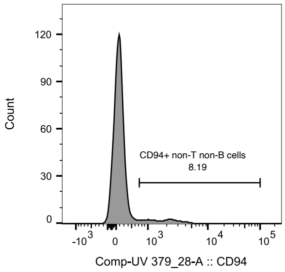

[20180806 Batch4_612_023.fcs]
non-T non-B cells
1807

612
06-AUG-2018

T cells

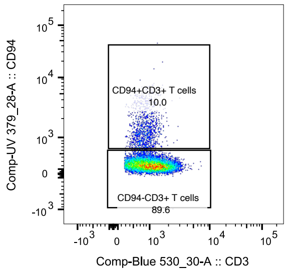

[20180806 Batch4_612_023.fcs]
CD3+ T cells
11426

CD94- T cells

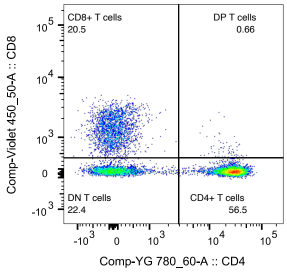

[20180806 Batch4_612_023.fcs]
CD94-CD3+ T cells
10243

CD4 T cells

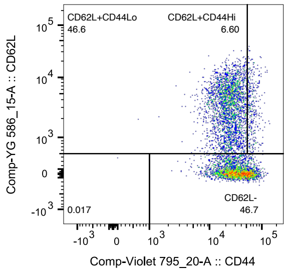

[20180806 Batch4_612_023.fcs]
CD4+ T cells
5790

CD4 T cells

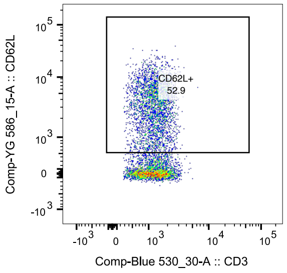

[20180806 Batch4_612_023.fcs]
CD4+ T cells
5790

CD8 T cells

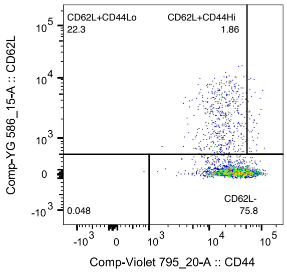

[20180806 Batch4_612_023.fcs]
CD8+ T cells
2095

CD8 T cells

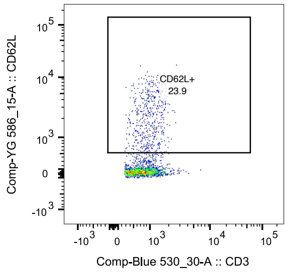

[20180806 Batch4_612_023.fcs]
CD8+ T cells
2095

DN T cells

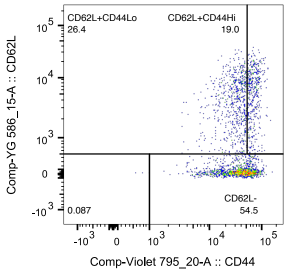

[20180806 Batch4_612_023.fcs]
DN T cells
2290

DN T cells

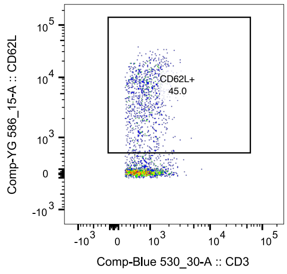

[20180806 Batch4_612_023.fcs]
DN T cells
2290

DP T cells

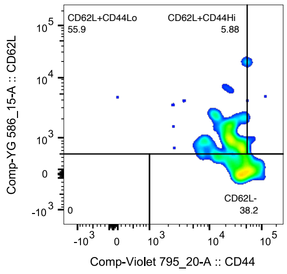

[20180806 Batch4_612_023.fcs]
DP T cells
68.0

DP T cells

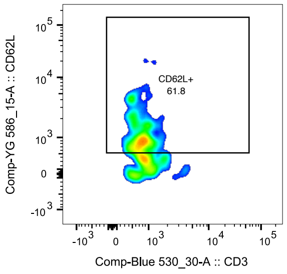

[20180806 Batch4_612_023.fcs]
DP T cells
68.0

506
06-AUG-2018

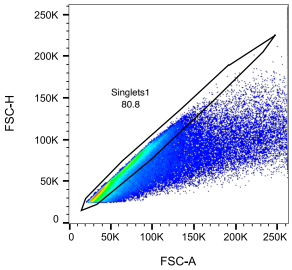

[20180806 Batch4_506_001.fcs]
Ungated
237994

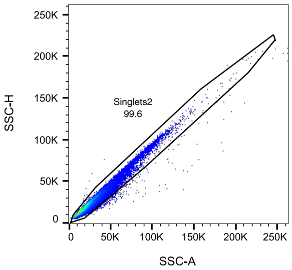

[20180806 Batch4_506_001.fcs]
Singlets1
192248

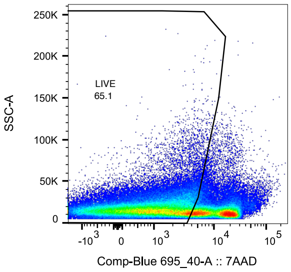

[20180806 Batch4_506_001.fcs]
Singlets2
191499

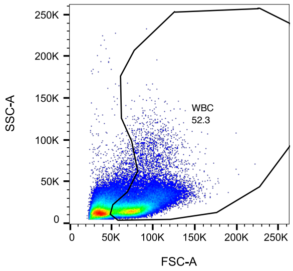

[20180806 Batch4_506_001.fcs]
LIVE
124608

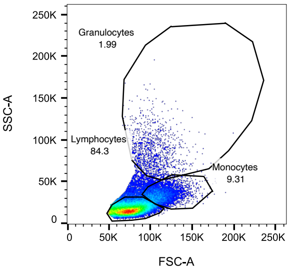

[20180806 Batch4_506_001.fcs]
WBC
65187

Granulocytes

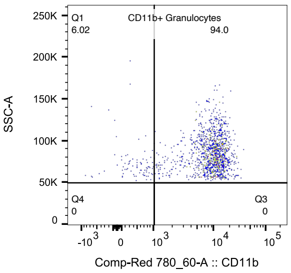

[20180806 Batch4_506_001.fcs]
Granulocytes
1295

Monocytes

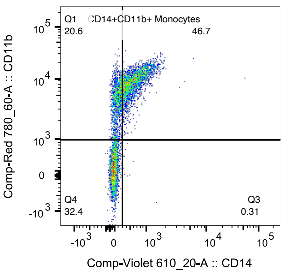

[20180806 Batch4_506_001.fcs]
Monocytes
6068

Lymphocytes

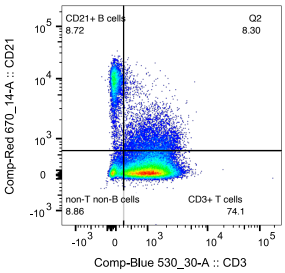

[20180806 Batch4_506_001.fcs]
Lymphocytes
54949

non-T non-B

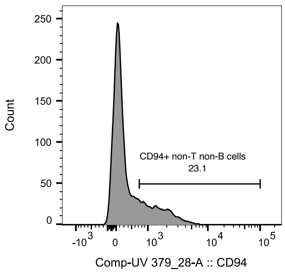

[20180806 Batch4_506_001.fcs]
non-T non-B cells
4866

506
06-AUG-2018

T cells

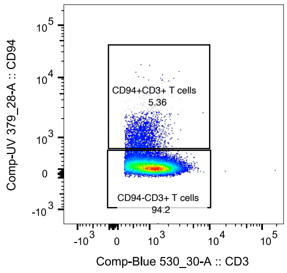

[20180806 Batch4_506_001.fcs]
CD3+ T cells
40731

CD94- T cells

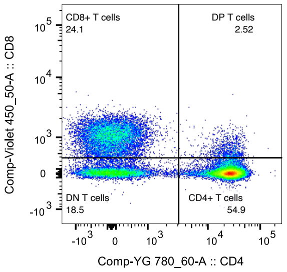

[20180806 Batch4_506_001.fcs]
CD94-CD3+ T cells
38356

CD4 T cells

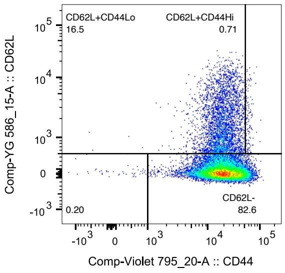

[20180806 Batch4_506_001.fcs]
CD4+ T cells
21063

CD4 T cells

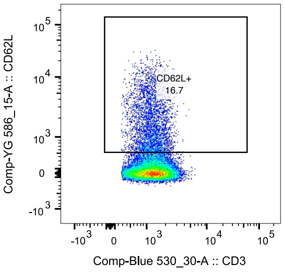

[20180806 Batch4_506_001.fcs]
CD4+ T cells
21063

CD8 T cells

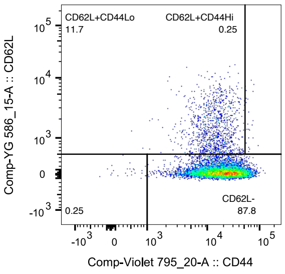

[20180806 Batch4_506_001.fcs]
CD8+ T cells
9230

CD8 T cells

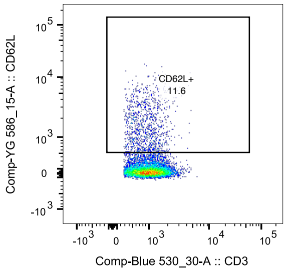

[20180806 Batch4_506_001.fcs]
CD8+ T cells
9230

DN T cells

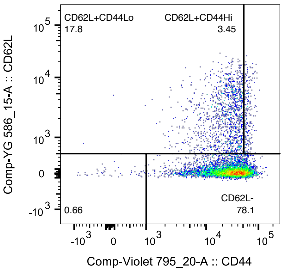

[20180806 Batch4_506_001.fcs]
DN T cells
7097

DN T cells

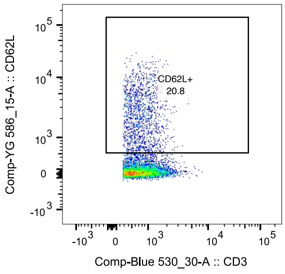

[20180806 Batch4_506_001.fcs]
DN T cells
7097

DP T cells

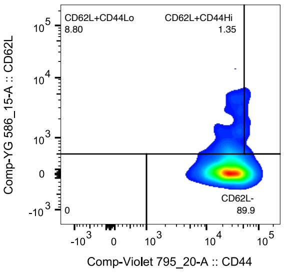

[20180806 Batch4_506_001.fcs]
DP T cells
966

DP T cells

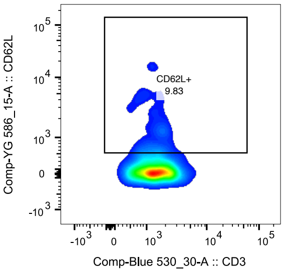

[20180806 Batch4_506_001.fcs]
DP T cells
966

520
06-AUG-2018

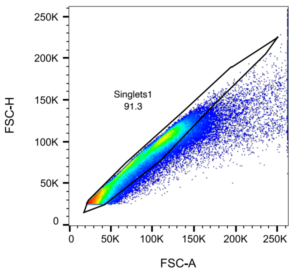

[20180806 Batch4_520_007.fcs]
Ungated
138149

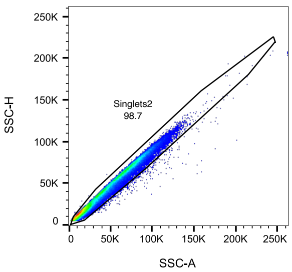

[20180806 Batch4_520_007.fcs]
Singlets1
126123

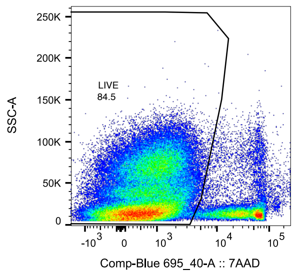

[20180806 Batch4_520_007.fcs]
Singlets2
124443

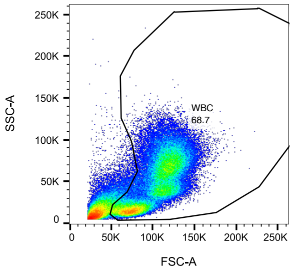

[20180806 Batch4_520_007.fcs]
LIVE
105108

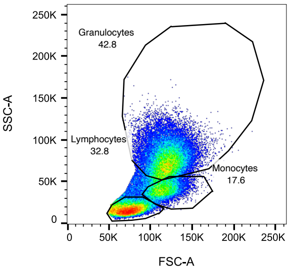

[20180806 Batch4_520_007.fcs]
WBC
72171

Granulocytes

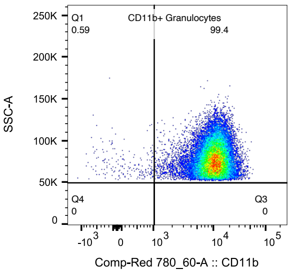

[20180806 Batch4_520_007.fcs]
Granulocytes
30924

Monocytes

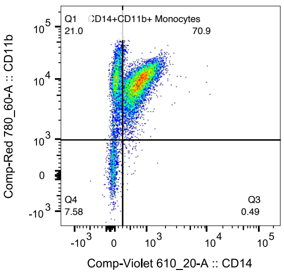

[20180806 Batch4_520_007.fcs]
Monocytes
12721

Lymphocytes

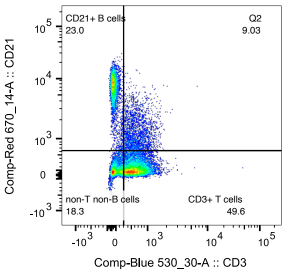

[20180806 Batch4_520_007.fcs]
Lymphocytes
23679

non-T non-B

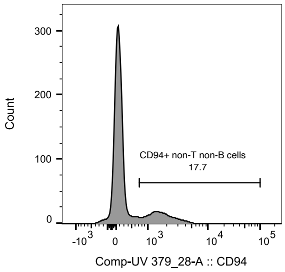

[20180806 Batch4_520_007.fcs]
non-T non-B cells
4344

520
06-AUG-2018

T cells

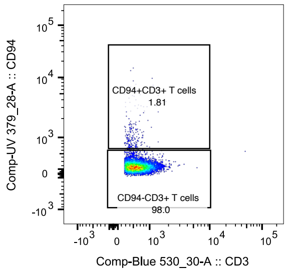

[20180806 Batch4_520_007.fcs]
CD3+ T cells
11752

CD94- T cells

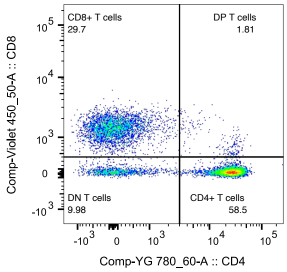

[20180806 Batch4_520_007.fcs]
CD94-CD3+ T cells
11518

CD4 T cells

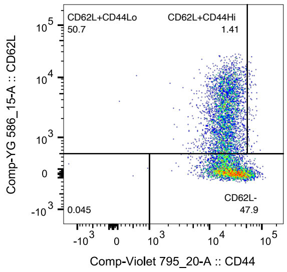

[20180806 Batch4_520_007.fcs]
CD4+ T cells
6734

CD4 T cells

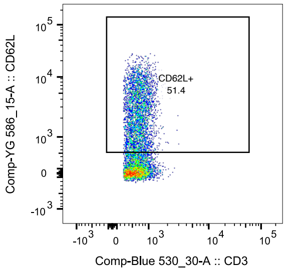

[20180806 Batch4_520_007.fcs]
CD4+ T cells
6734

CD8 T cells

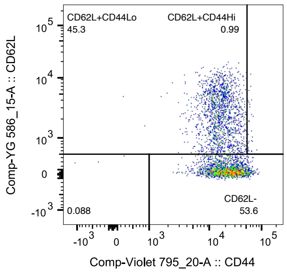

[20180806 Batch4_520_007.fcs]
CD8+ T cells
3426

CD8 T cells

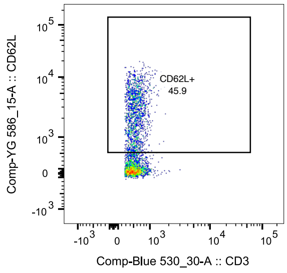

[20180806 Batch4_520_007.fcs]
CD8+ T cells
3426

DN T cells

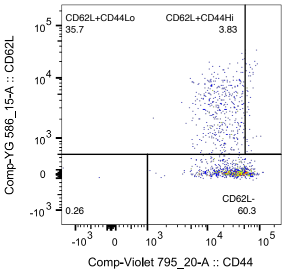

[20180806 Batch4_520_007.fcs]
DN T cells
1150

DN T cells

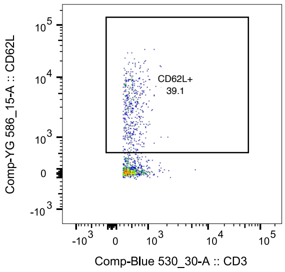

[20180806 Batch4_520_007.fcs]
DN T cells
1150

DP T cells

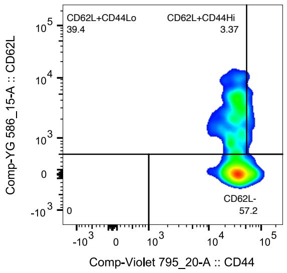

[20180806 Batch4_520_007.fcs]
DP T cells
208

DP T cells

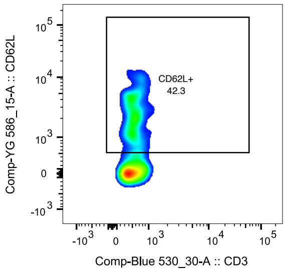

[20180806 Batch4_520_007.fcs]
DP T cells
208

610
06-AUG-2018

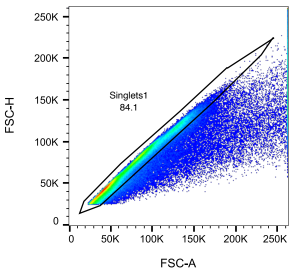

[20180806 Batch4_610_021.fcs]
Ungated
245274

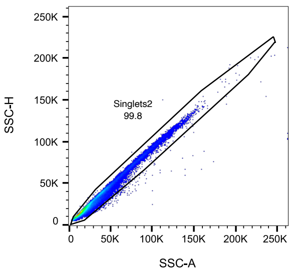

[20180806 Batch4_610_021.fcs]
Singlets1
206292

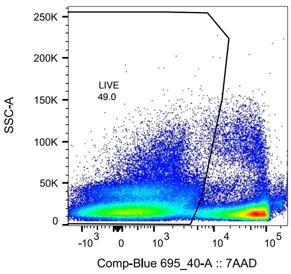

[20180806 Batch4_610_021.fcs]
Singlets2
205950

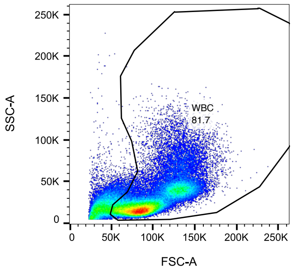

[20180806 Batch4_610_021.fcs]
LIVE
101008

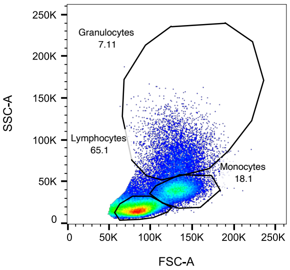

[20180806 Batch4_610_021.fcs]
WBC
82527

Granulocytes

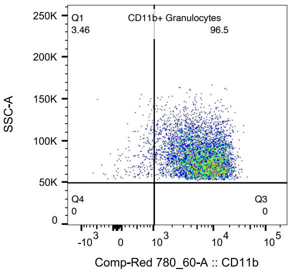

[20180806 Batch4_610_021.fcs]
Granulocytes
5864

Monocytes

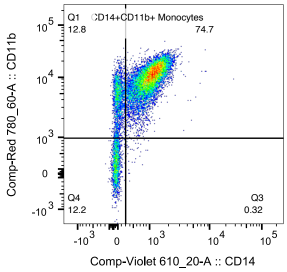

[20180806 Batch4_610_021.fcs]
Monocytes
14945

Lymphocytes

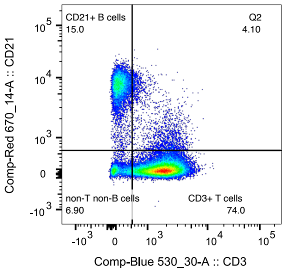

[20180806 Batch4_610_021.fcs]
Lymphocytes
53712

non-T non-B

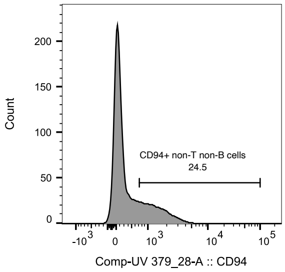

[20180806 Batch4_610_021.fcs]
non-T non-B cells
3705

610
06-AUG-2018

T cells

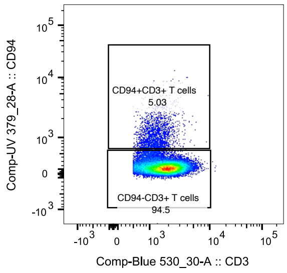

[20180806 Batch4_610_021.fcs]
CD3+ T cells
39734

CD94- T cells

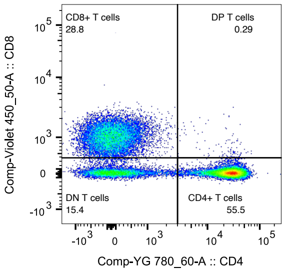

[20180806 Batch4_610_021.fcs]
CD94-CD3+ T cells
37560

CD4 T cells

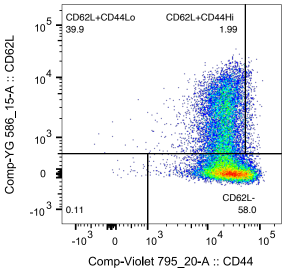

[20180806 Batch4_610_021.fcs]
CD4+ T cells
20839

CD4 T cells

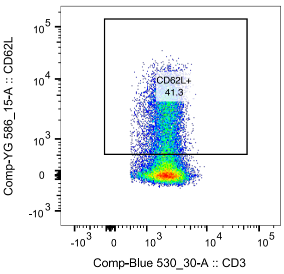

[20180806 Batch4_610_021.fcs]
CD4+ T cells
20839

CD8 T cells

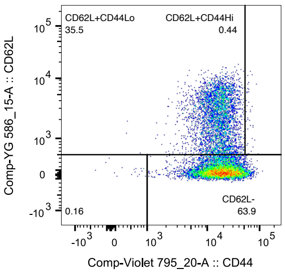

[20180806 Batch4_610_021.fcs]
CD8+ T cells
10834

CD8 T cells

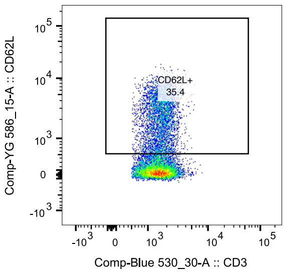

[20180806 Batch4_610_021.fcs]
CD8+ T cells
10834

DN T cells

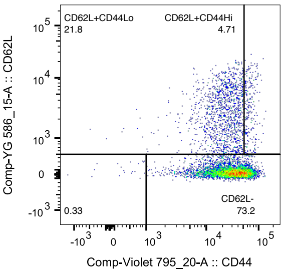

[20180806 Batch4_610_021.fcs]
DN T cells
5779

DN T cells

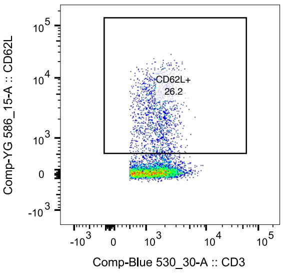

[20180806 Batch4_610_021.fcs]
DN T cells
5779

DP T cells

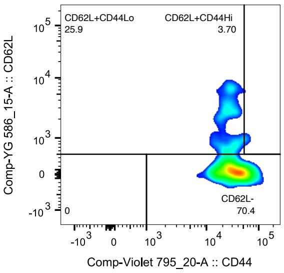

[20180806 Batch4_610_021.fcs]
DP T cells
108

DP T cells

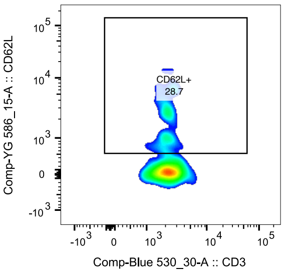

[20180806 Batch4_610_021.fcs]
DP T cells
108

517
06-AUG-2018

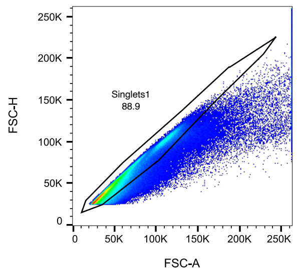

[20180806 Batch4_517_006.fcs]
Ungated
4.96E5

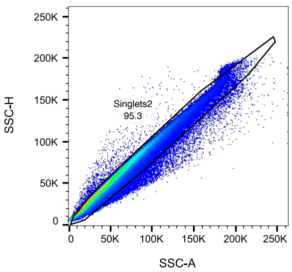

[20180806 Batch4_517_006.fcs]
Singlets1
4.41E5

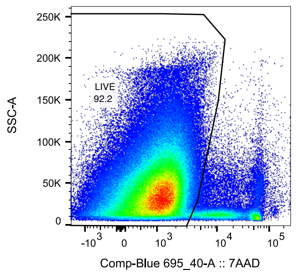

[20180806 Batch4_517_006.fcs]
Singlets2
4.20E5

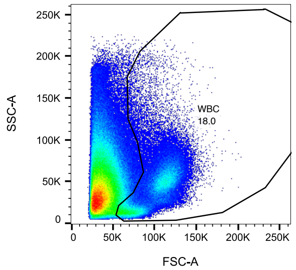

[20180806 Batch4_517_006.fcs]
LIVE
387142

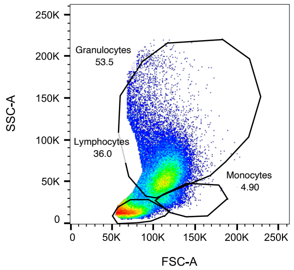

[20180806 Batch4_517_006.fcs]
WBC
69726

Granulocytes

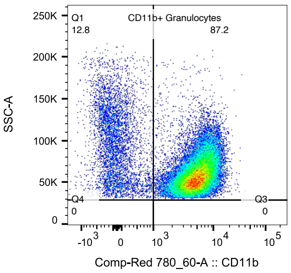

[20180806 Batch4_517_006.fcs]
Granulocytes
37284

Monocytes

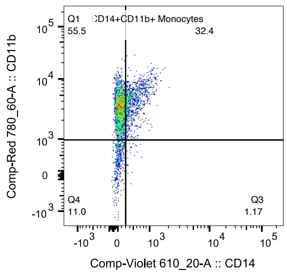

[20180806 Batch4_517_006.fcs]
Monocytes
3418

Lymphocytes

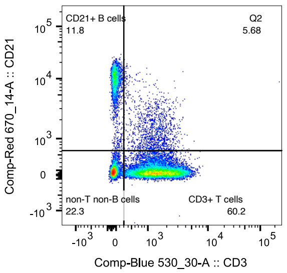

[20180806 Batch4_517_006.fcs]
Lymphocytes
25096

non-T non-B

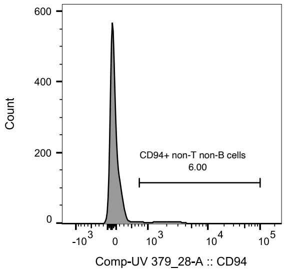

[20180806 Batch4_517_006.fcs]
non-T non-B cells
5601

517
06-AUG-2018

T cells

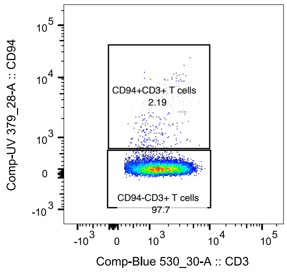

[20180806 Batch4_517_006.fcs]
CD3+ T cells
15101

CD94- T cells

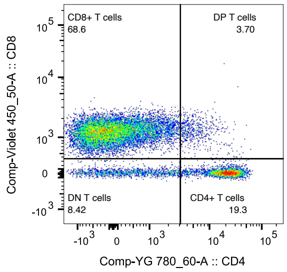

[20180806 Batch4_517_006.fcs]
CD94-CD3+ T cells
14756

CD4 T cells

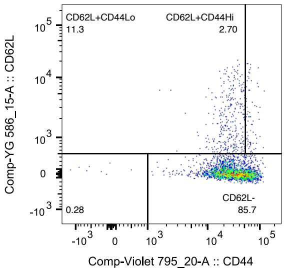

[20180806 Batch4_517_006.fcs]
CD4+ T cells
2850

CD4 T cells

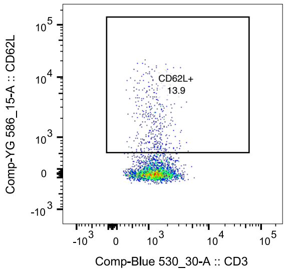

[20180806 Batch4_517_006.fcs]
CD4+ T cells
2850

CD8 T cells

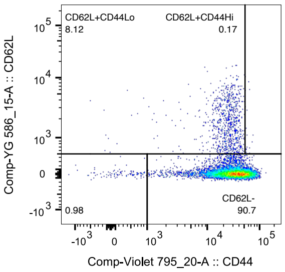

[20180806 Batch4_517_006.fcs]
CD8+ T cells
10117

CD8 T cells

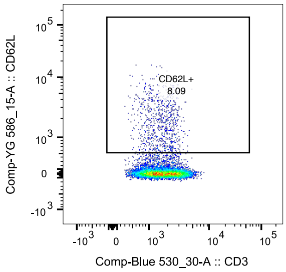

[20180806 Batch4_517_006.fcs]
CD8+ T cells
10117

DN T cells

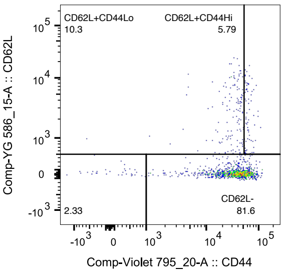

[20180806 Batch4_517_006.fcs]
DN T cells
1243

DN T cells

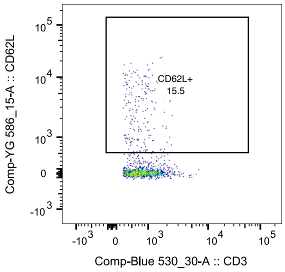

[20180806 Batch4_517_006.fcs]
DN T cells
1243

DP T cells

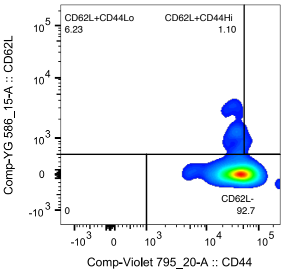

[20180806 Batch4_517_006.fcs]
DP T cells
546

DP T cells

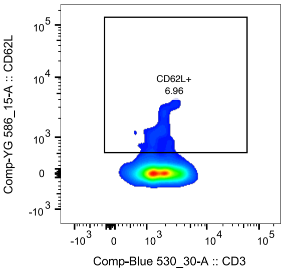

[20180806 Batch4_517_006.fcs]
DP T cells
546

616
06-AUG-2018

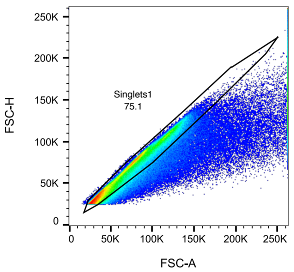

[20180806 Batch4_616_026.fcs]
Ungated
240977

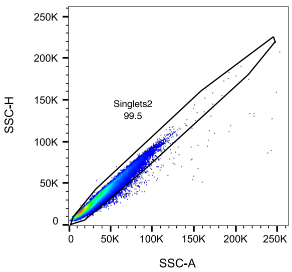

[20180806 Batch4_616_026.fcs]
Singlets1
181003

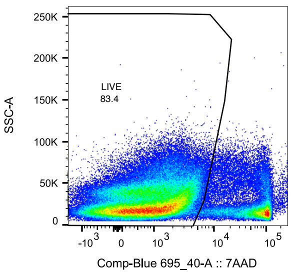

[20180806 Batch4_616_026.fcs]
Singlets2
180105

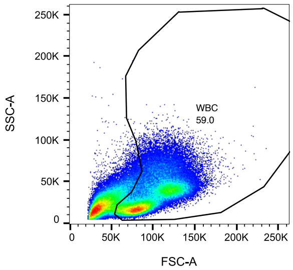

[20180806 Batch4_616_026.fcs]
LIVE
150173

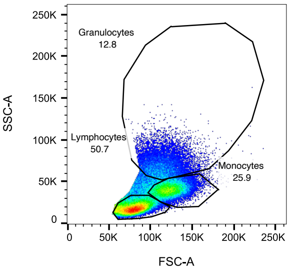

[20180806 Batch4_616_026.fcs]
WBC
88559

Granulocytes

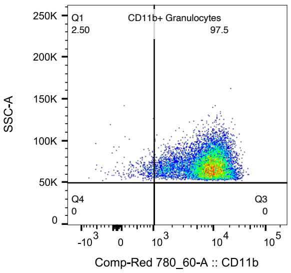

[20180806 Batch4_616_026.fcs]
Granulocytes
11301

Monocytes

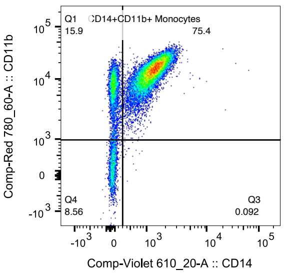

[20180806 Batch4_616_026.fcs]
Monocytes
22932

Lymphocytes

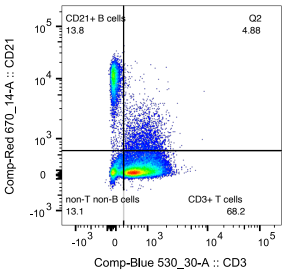

[20180806 Batch4_616_026.fcs]
Lymphocytes
44935

non-T non-B

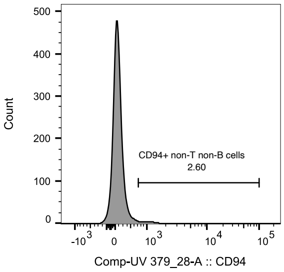

[20180806 Batch4_616_026.fcs]
non-T non-B cells
5892

616
06-AUG-2018

T cells

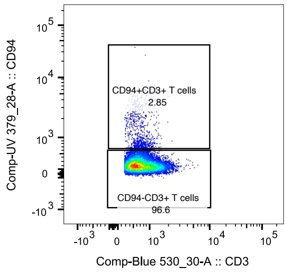

[20180806 Batch4_616_026.fcs]
CD3+ T cells
30653

CD94- T cells

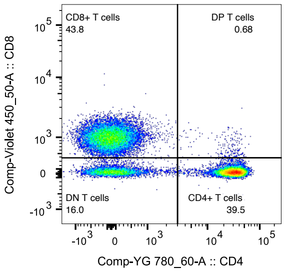

[20180806 Batch4_616_026.fcs]
CD94-CD3+ T cells
29609

CD4 T cells

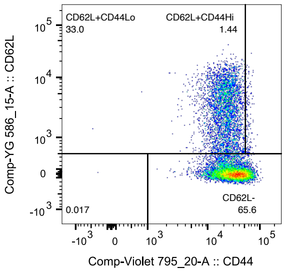

[20180806 Batch4_616_026.fcs]
CD4+ T cells
11701

CD4 T cells

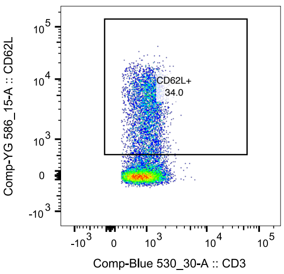

[20180806 Batch4_616_026.fcs]
CD4+ T cells
11701

CD8 T cells

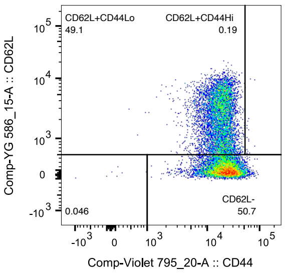

[20180806 Batch4_616_026.fcs]
CD8+ T cells
12967

CD8 T cells

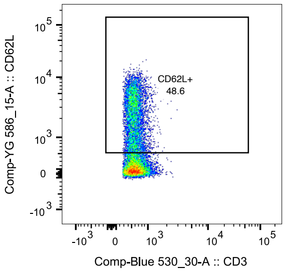

[20180806 Batch4_616_026.fcs]
CD8+ T cells
12967

DN T cells

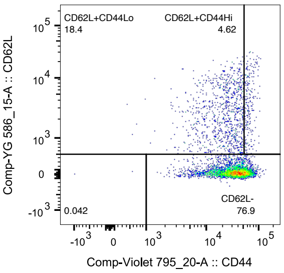

[20180806 Batch4_616_026.fcs]
DN T cells
4739

DN T cells

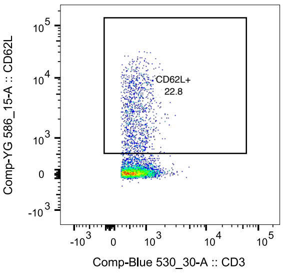

[20180806 Batch4_616_026.fcs]
DN T cells
4739

DP T cells

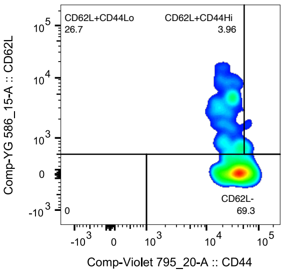

[20180806 Batch4_616_026.fcs]
DP T cells
202

DP T cells

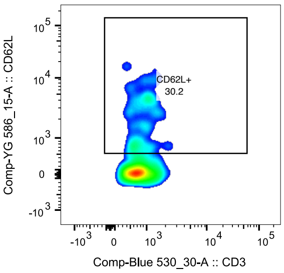

[20180806 Batch4_616_026.fcs]
DP T cells
202

611
06-AUG-2018

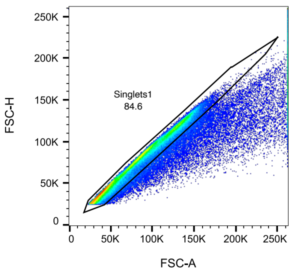

[20180806 Batch4_611_022.fcs]
Ungated
143678

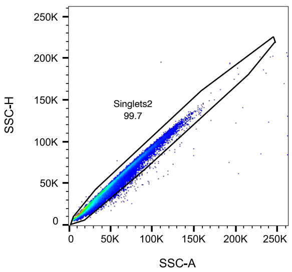

[20180806 Batch4_611_022.fcs]
Singlets1
121509

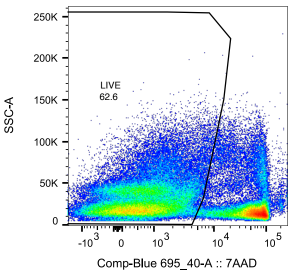

[20180806 Batch4_611_022.fcs]
Singlets2
121096

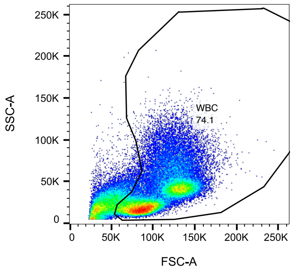

[20180806 Batch4_611_022.fcs]
LIVE
75762

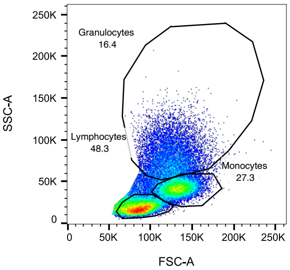

[20180806 Batch4_611_022.fcs]
WBC
56106

Granulocytes

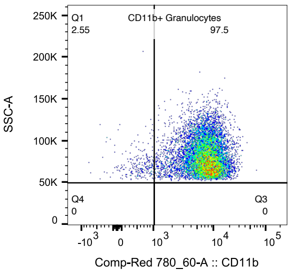

[20180806 Batch4_611_022.fcs]
Granulocytes
9185

Monocytes

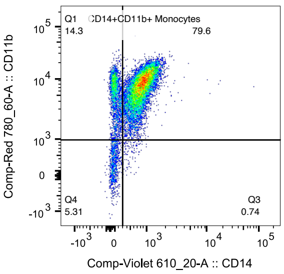

[20180806 Batch4_611_022.fcs]
Monocytes
15342

Lymphocytes

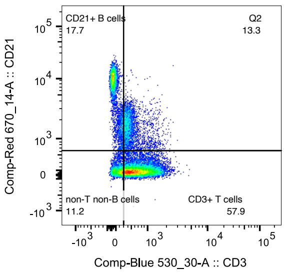

[20180806 Batch4_611_022.fcs]
Lymphocytes
27109

non-T non-B

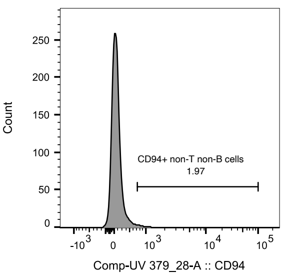

[20180806 Batch4_611_022.fcs]
non-T non-B cells
3041

611
06-AUG-2018

T cells

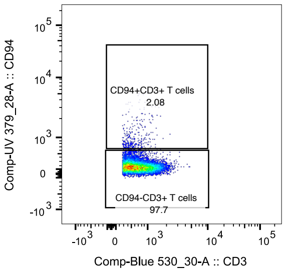

[20180806 Batch4_611_022.fcs]
CD3+ T cells
15683

CD94- T cells

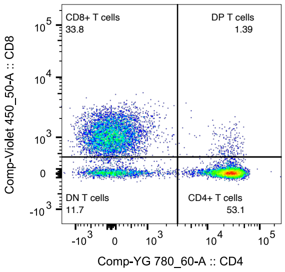

[20180806 Batch4_611_022.fcs]
CD94-CD3+ T cells
15320

CD4 T cells

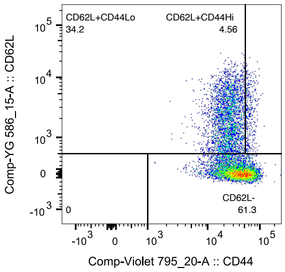

[20180806 Batch4_611_022.fcs]
CD4+ T cells
8136

CD4 T cells

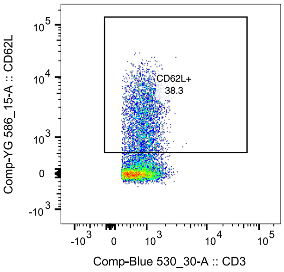

[20180806 Batch4_611_022.fcs]
CD4+ T cells
8136

CD8 T cells

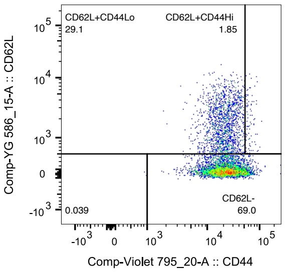

[20180806 Batch4_611_022.fcs]
CD8+ T cells
5178

CD8 T cells

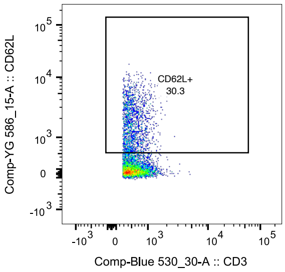

[20180806 Batch4_611_022.fcs]
CD8+ T cells
5178

DN T cells

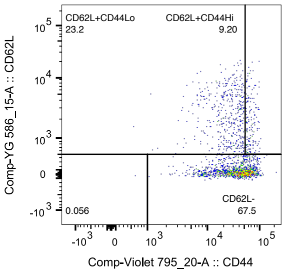

[20180806 Batch4_611_022.fcs]
DN T cells
1793

DN T cells

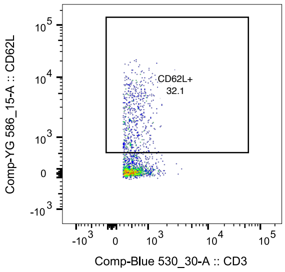

[20180806 Batch4_611_022.fcs]
DN T cells
1793

DP T cells

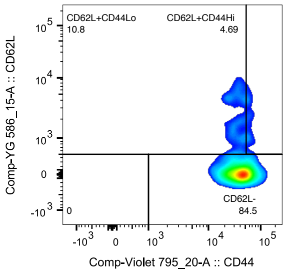

[20180806 Batch4_611_022.fcs]
DP T cells
213

DP T cells

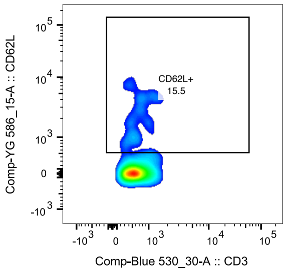

[20180806 Batch4_611_022.fcs]
DP T cells
213

531
06-AUG-2018

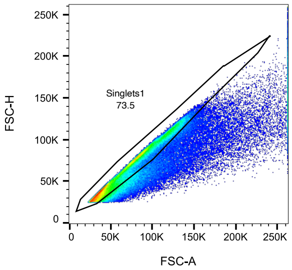

[20180806 Batch4_531_010.fcs]
Ungated
142990

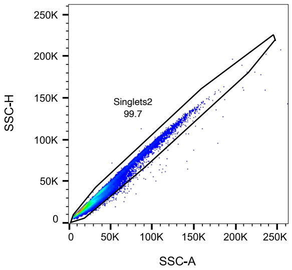

[20180806 Batch4_531_010.fcs]
Singlets1
105102

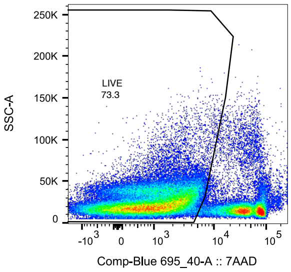

[20180806 Batch4_531_010.fcs]
Singlets2
104825

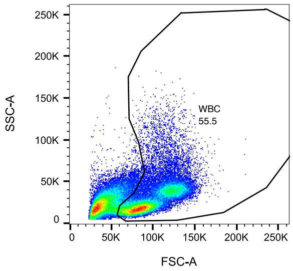

[20180806 Batch4_531_010.fcs]
LIVE
76831

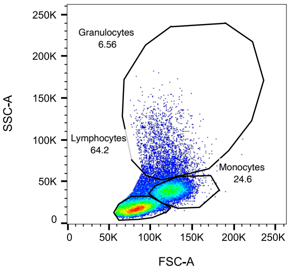

[20180806 Batch4_531_010.fcs]
WBC
42670

Granulocytes

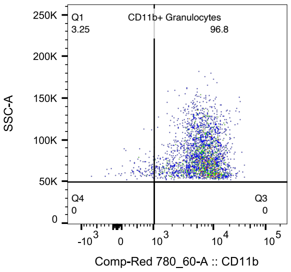

[20180806 Batch4_531_010.fcs]
Granulocytes
2801

Monocytes

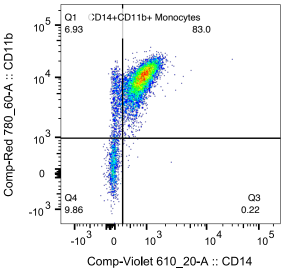

[20180806 Batch4_531_010.fcs]
Monocytes
10513

Lymphocytes

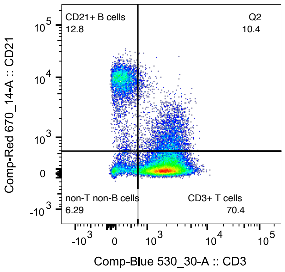

[20180806 Batch4_531_010.fcs]
Lymphocytes
27373

non-T non-B

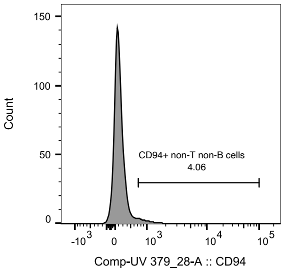

[20180806 Batch4_531_010.fcs]
non-T non-B cells
1723

531
06-AUG-2018

T cells

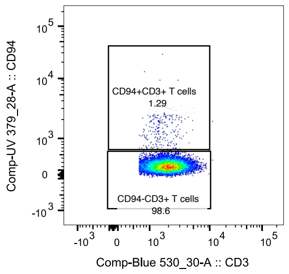

[20180806 Batch4_531_010.fcs]
CD3+ T cells
19283

CD94- T cells

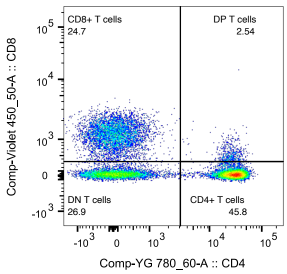

[20180806 Batch4_531_010.fcs]
CD94-CD3+ T cells
19010

CD4 T cells

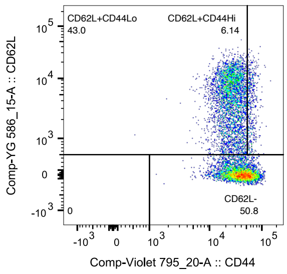

[20180806 Batch4_531_010.fcs]
CD4+ T cells
8708

CD4 T cells

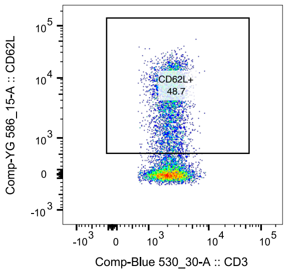

[20180806 Batch4_531_010.fcs]
CD4+ T cells
8708

CD8 T cells

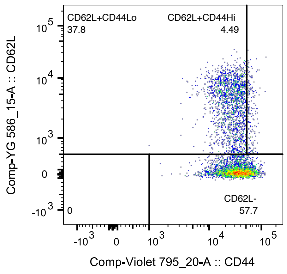

[20180806 Batch4_531_010.fcs]
CD8+ T cells
4697

CD8 T cells

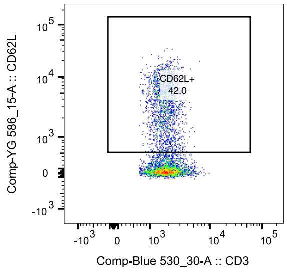

[20180806 Batch4_531_010.fcs]
CD8+ T cells
4697

DN T cells

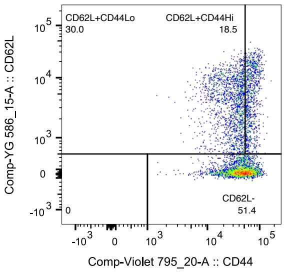

[20180806 Batch4_531_010.fcs]
DN T cells
5123

DN T cells

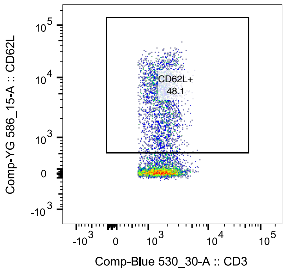

[20180806 Batch4_531_010.fcs]
DN T cells
5123

DP T cells

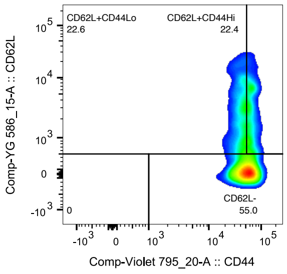

[20180806 Batch4_531_010.fcs]
DP T cells
482

DP T cells

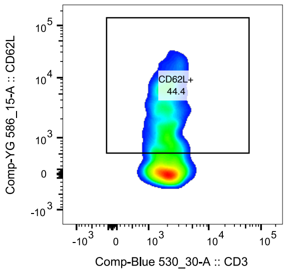

[20180806 Batch4_531_010.fcs]
DP T cells
482

528
06-AUG-2018

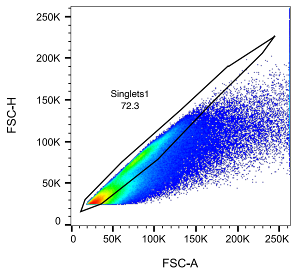

[20180806 Batch4_528_009.fcs]
Ungated
305472

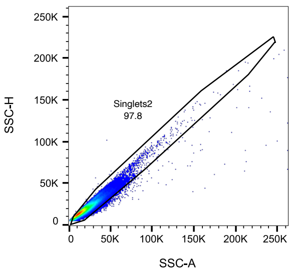

[20180806 Batch4_528_009.fcs]
Singlets1
220826

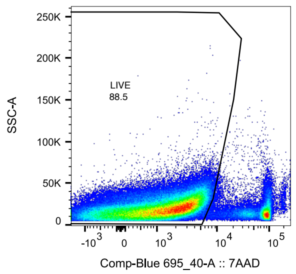

[20180806 Batch4_528_009.fcs]
Singlets2
215907

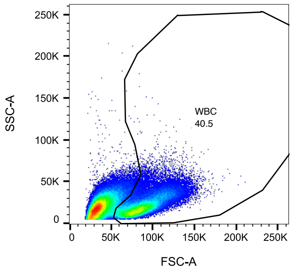

[20180806 Batch4_528_009.fcs]
LIVE
191042

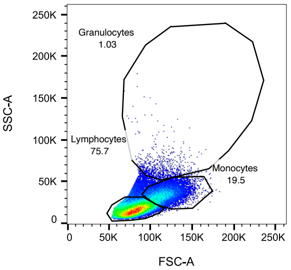

[20180806 Batch4_528_009.fcs]
WBC
77366

Granulocytes

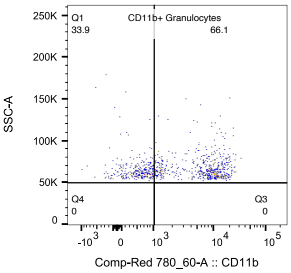

[20180806 Batch4_528_009.fcs]
Granulocytes
800

Monocytes

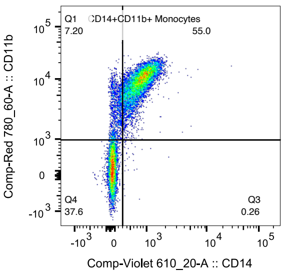

[20180806 Batch4_528_009.fcs]
Monocytes
15049

Lymphocytes

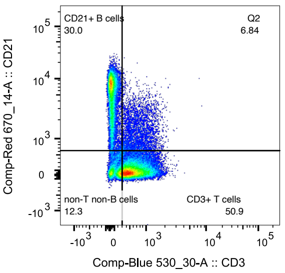

[20180806 Batch4_528_009.fcs]
Lymphocytes
58528

non-T non-B

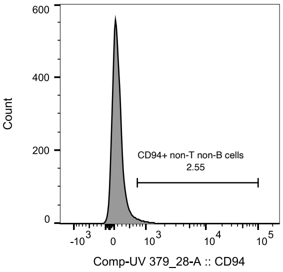

[20180806 Batch4_528_009.fcs]
non-T non-B cells
7204

528
06-AUG-2018

T cells

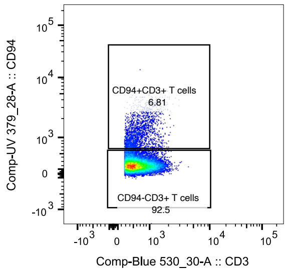

[20180806 Batch4_528_009.fcs]
CD3+ T cells
29762

CD94- T cells

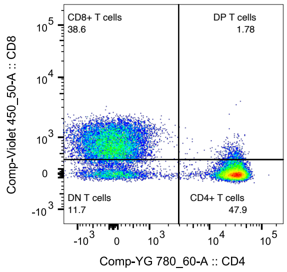

[20180806 Batch4_528_009.fcs]
CD94-CD3+ T cells
27528

CD4 T cells

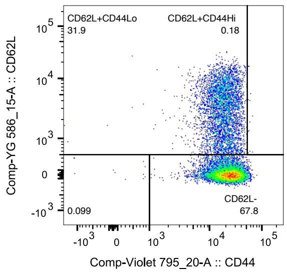

[20180806 Batch4_528_009.fcs]
CD4+ T cells
13191

CD4 T cells

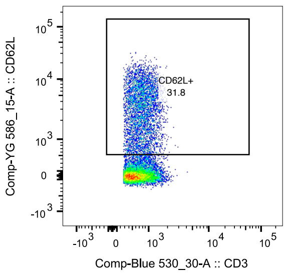

[20180806 Batch4_528_009.fcs]
CD4+ T cells
13191

CD8 T cells

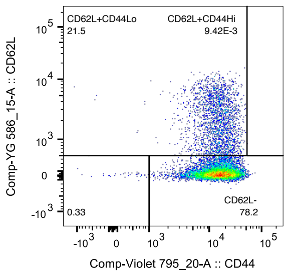

[20180806 Batch4_528_009.fcs]
CD8+ T cells
10618

CD8 T cells

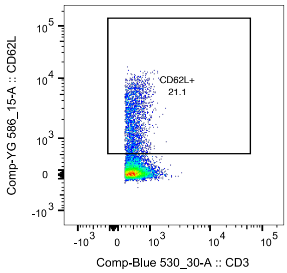

[20180806 Batch4_528_009.fcs]
CD8+ T cells
10618

DN T cells

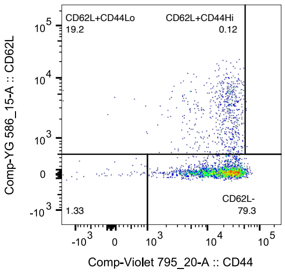

[20180806 Batch4_528_009.fcs]
DN T cells
3229

DN T cells

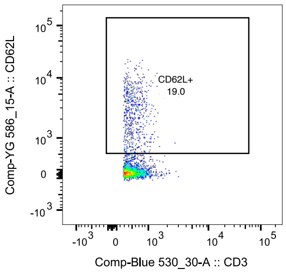

[20180806 Batch4_528_009.fcs]
DN T cells
3229

DP T cells

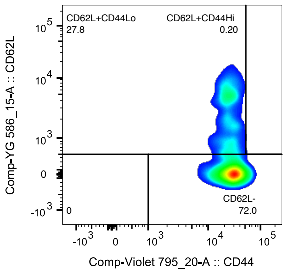

[20180806 Batch4_528_009.fcs]
DP T cells
490

DP T cells

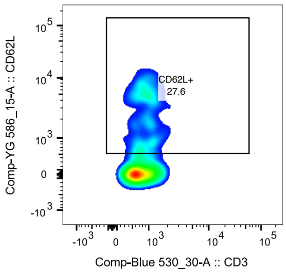

[20180806 Batch4_528_009.fcs]
DP T cells
490

600
06-AUG-2018

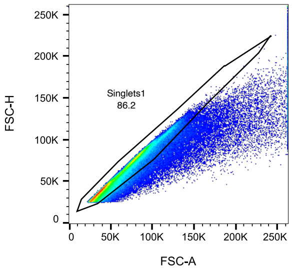

[20180806 Batch4_600_019.fcs]
Ungated
185340

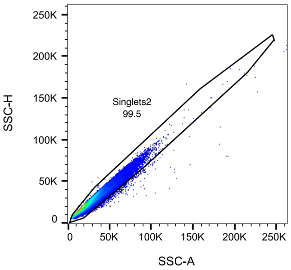

[20180806 Batch4_600_019.fcs]
Singlets1
159855

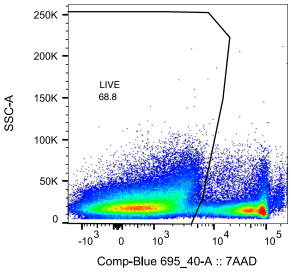

[20180806 Batch4_600_019.fcs]
Singlets2
159132

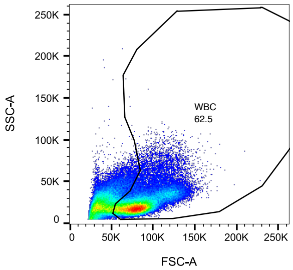

[20180806 Batch4_600_019.fcs]
LIVE
109549

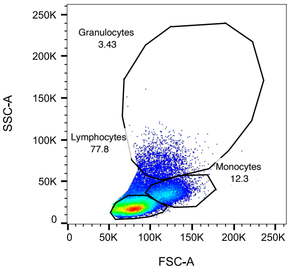

[20180806 Batch4_600_019.fcs]
WBC
68422

Granulocytes

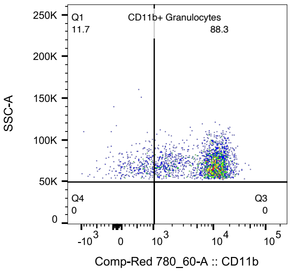

[20180806 Batch4_600_019.fcs]
Granulocytes
2349

Monocytes

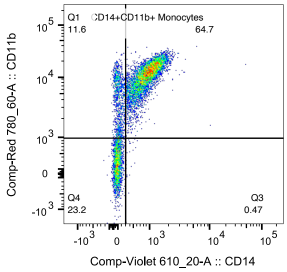

[20180806 Batch4_600_019.fcs]
Monocytes
8445

Lymphocytes

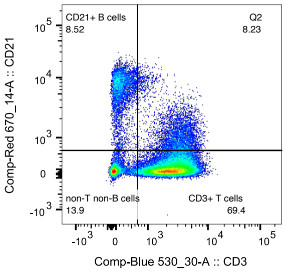

[20180806 Batch4_600_019.fcs]
Lymphocytes
53224

non-T non-B

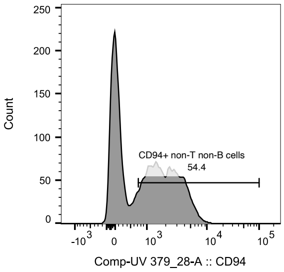

[20180806 Batch4_600_019.fcs]
non-T non-B cells
7378

600
06-AUG-2018

T cells

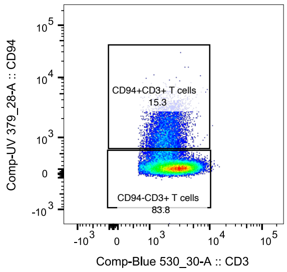

[20180806 Batch4_600_019.fcs]
CD3+ T cells
36930

CD94- T cells

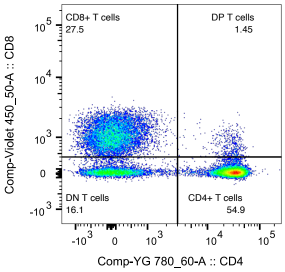

[20180806 Batch4_600_019.fcs]
CD94-CD3+ T cells
30931

CD4 T cells

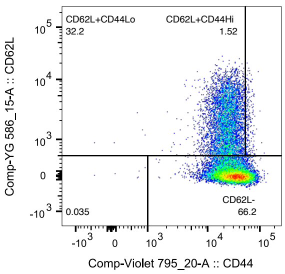

[20180806 Batch4_600_019.fcs]
CD4+ T cells
16993

CD4 T cells

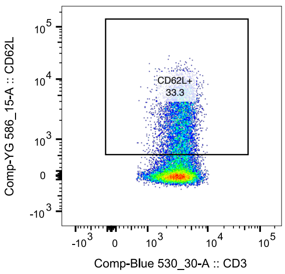

[20180806 Batch4_600_019.fcs]
CD4+ T cells
16993

CD8 T cells

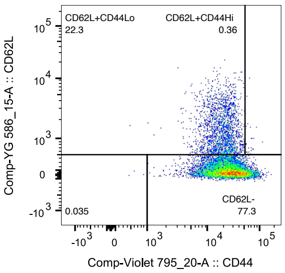

[20180806 Batch4_600_019.fcs]
CD8+ T cells
8515

CD8 T cells

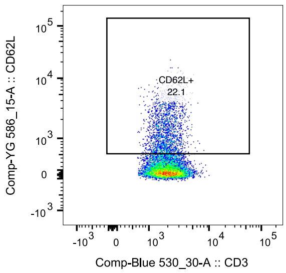

[20180806 Batch4_600_019.fcs]
CD8+ T cells
8515

DN T cells

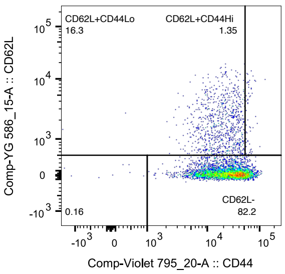

[20180806 Batch4_600_019.fcs]
DN T cells
4973

DN T cells

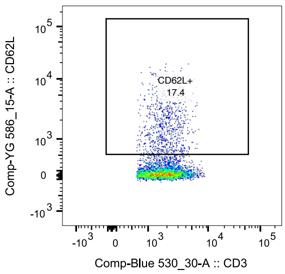

[20180806 Batch4_600_019.fcs]
DN T cells
4973

DP T cells

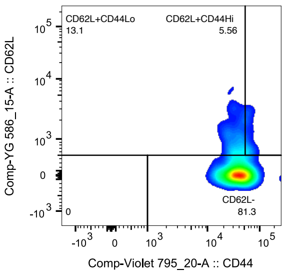

[20180806 Batch4_600_019.fcs]
DP T cells
450

DP T cells

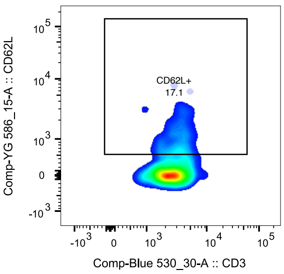

[20180806 Batch4_600_019.fcs]
DP T cells
450

613
06-AUG-2018

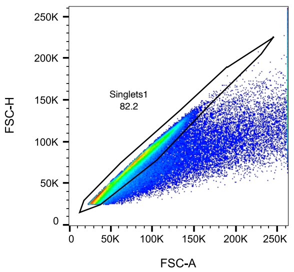

[20180806 Batch4_613_024.fcs]
Ungated
166144

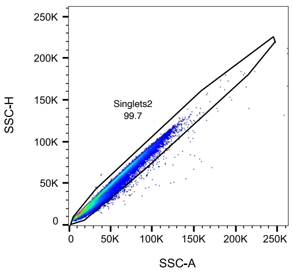

[20180806 Batch4_613_024.fcs]
Singlets1
136512

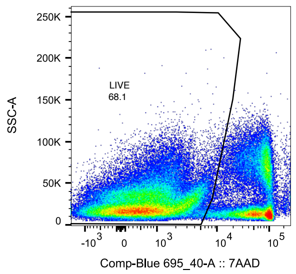

[20180806 Batch4_613_024.fcs]
Singlets2
136133

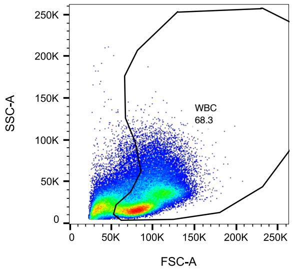

[20180806 Batch4_613_024.fcs]
LIVE
92740

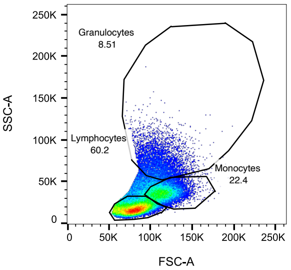

[20180806 Batch4_613_024.fcs]
WBC
63339

Granulocytes

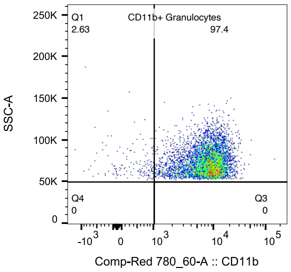

[20180806 Batch4_613_024.fcs]
Granulocytes
5389

Monocytes

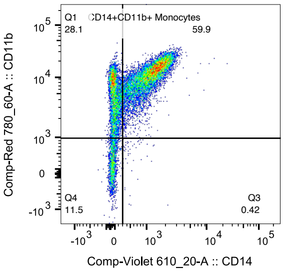

[20180806 Batch4_613_024.fcs]
Monocytes
14173

Lymphocytes

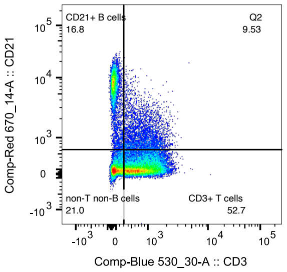

[20180806 Batch4_613_024.fcs]
Lymphocytes
38160

non-T non-B

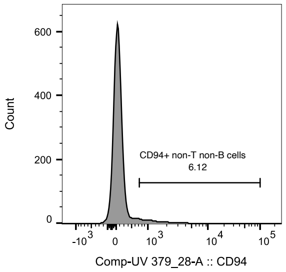

[20180806 Batch4_613_024.fcs]
non-T non-B cells
8020

613
06-AUG-2018

T cells

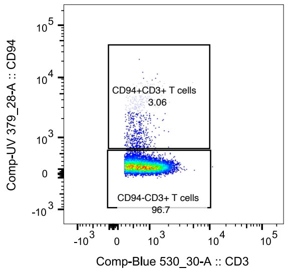

[20180806 Batch4_613_024.fcs]
CD3+ T cells
20106

CD94- T cells

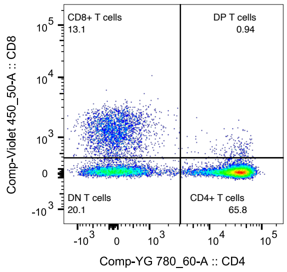

[20180806 Batch4_613_024.fcs]
CD94-CD3+ T cells
19445

CD4 T cells

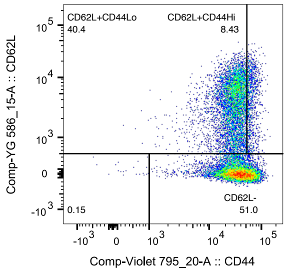

[20180806 Batch4_613_024.fcs]
CD4+ T cells
12794

CD4 T cells

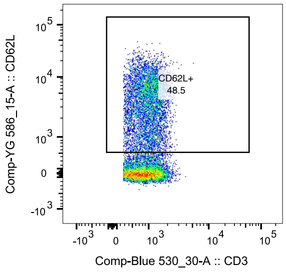

[20180806 Batch4_613_024.fcs]
CD4+ T cells
12794

CD8 T cells

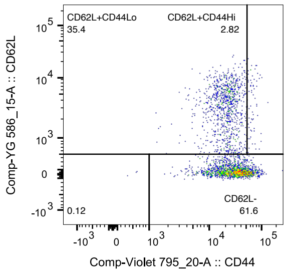

[20180806 Batch4_613_024.fcs]
CD8+ T cells
2556

CD8 T cells

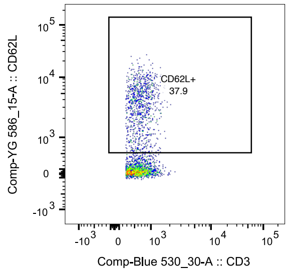

[20180806 Batch4_613_024.fcs]
CD8+ T cells
2556

DN T cells

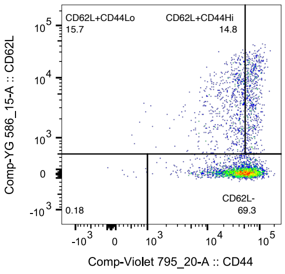

[20180806 Batch4_613_024.fcs]
DN T cells
3913

DN T cells

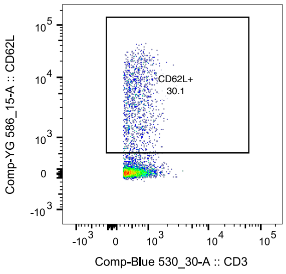

[20180806 Batch4_613_024.fcs]
DN T cells
3913

DP T cells

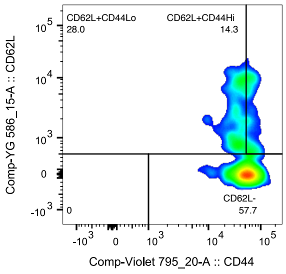

[20180806 Batch4_613_024.fcs]
DP T cells
182

DP T cells

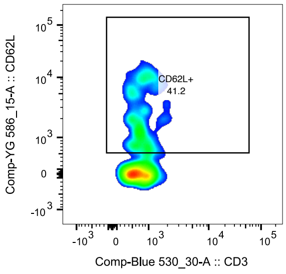

[20180806 Batch4_613_024.fcs]
DP T cells
182

534
06-AUG-2018

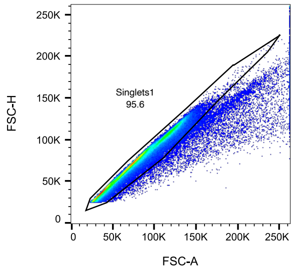

[20180806 Batch4_534_011.fcs]
Ungated
196270

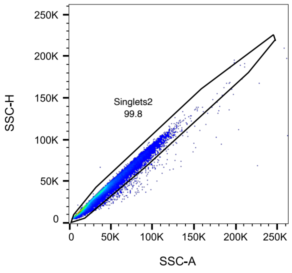

[20180806 Batch4_534_011.fcs]
Singlets1
187721

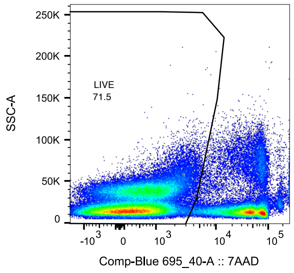

[20180806 Batch4_534_011.fcs]
Singlets2
187371

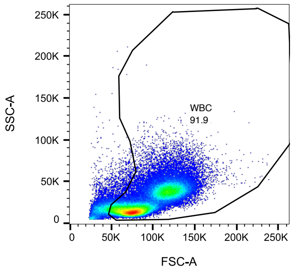

[20180806 Batch4_534_011.fcs]
LIVE
133898

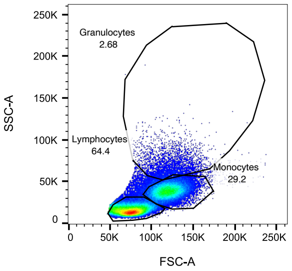

[20180806 Batch4_534_011.fcs]
WBC
123090

Granulocytes

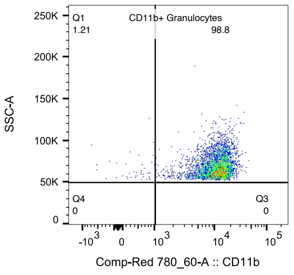

[20180806 Batch4_534_011.fcs]
Granulocytes
3295

Monocytes

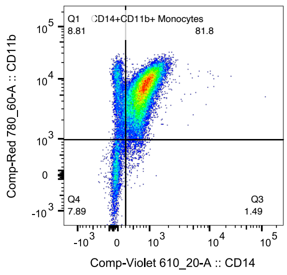

[20180806 Batch4_534_011.fcs]
Monocytes
35932

Lymphocytes

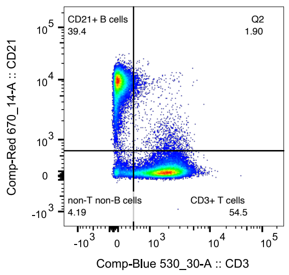

[20180806 Batch4_534_011.fcs]
Lymphocytes
79312

non-T non-B

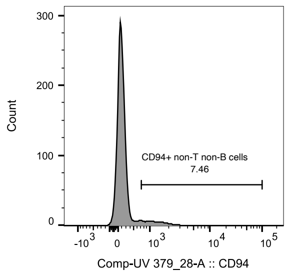

[20180806 Batch4_534_011.fcs]
non-T non-B cells
3324

534
06-AUG-2018

T cells

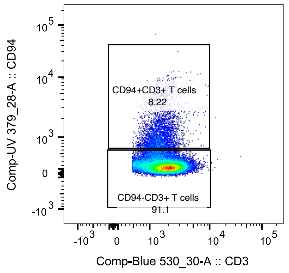

[20180806 Batch4_534_011.fcs]
CD3+ T cells
43208

CD94- T cells

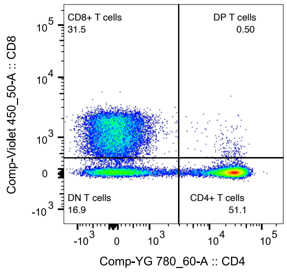

[20180806 Batch4_534_011.fcs]
CD94-CD3+ T cells
39341

CD4 T cells

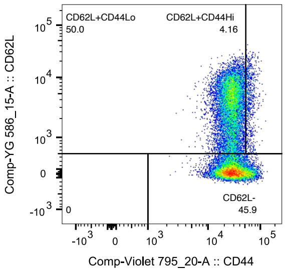

[20180806 Batch4_534_011.fcs]
CD4+ T cells
20096

CD4 T cells

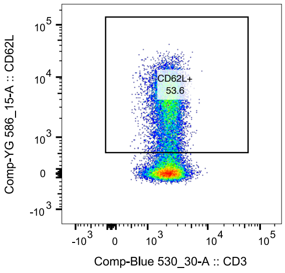

[20180806 Batch4_534_011.fcs]
CD4+ T cells
20096

CD8 T cells

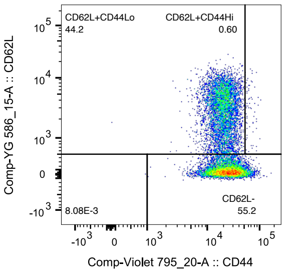

[20180806 Batch4_534_011.fcs]
CD8+ T cells
12381

CD8 T cells

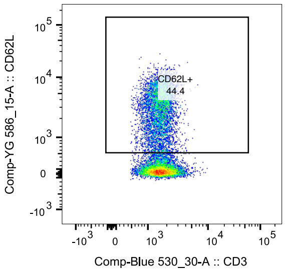

[20180806 Batch4_534_011.fcs]
CD8+ T cells
12381

DN T cells

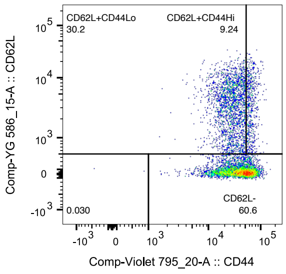

[20180806 Batch4_534_011.fcs]
DN T cells
6668

DN T cells

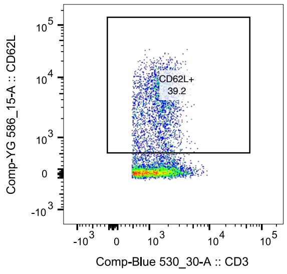

[20180806 Batch4_534_011.fcs]
DN T cells
6668

DP T cells

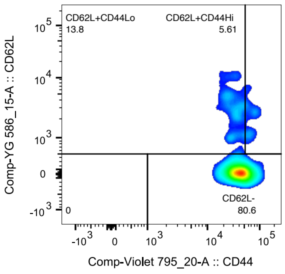

[20180806 Batch4_534_011.fcs]
DP T cells
196

DP T cells

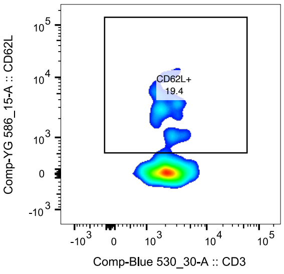

[20180806 Batch4_534_011.fcs]
DP T cells
196

581
06-AUG-2018

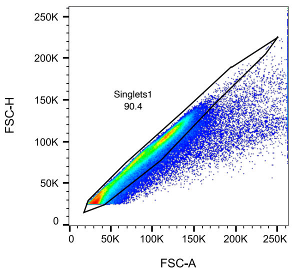

[20180806 Batch4_581_017.fcs]
Ungated
136033

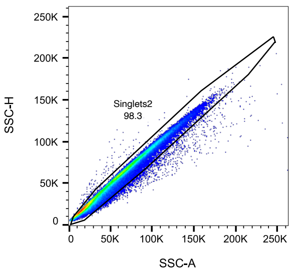

[20180806 Batch4_581_017.fcs]
Singlets1
122990

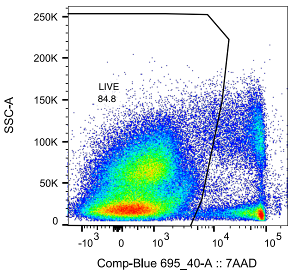

[20180806 Batch4_581_017.fcs]
Singlets2
120843

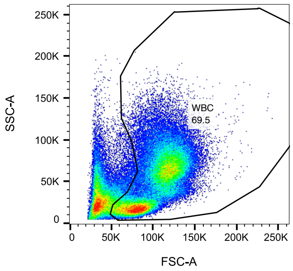

[20180806 Batch4_581_017.fcs]
LIVE
102451

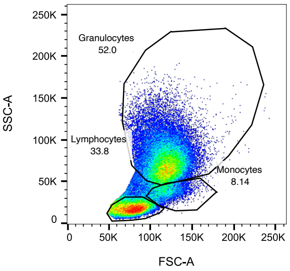

[20180806 Batch4_581_017.fcs]
WBC
71219

Granulocytes

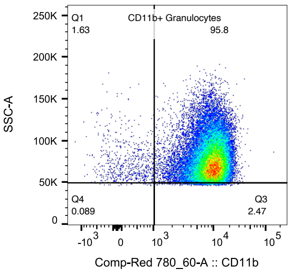

[20180806 Batch4_581_017.fcs]
Granulocytes
37042

Monocytes

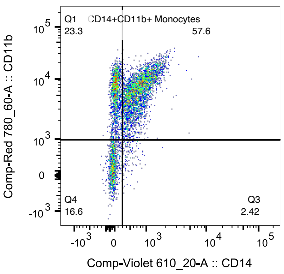

[20180806 Batch4_581_017.fcs]
Monocytes
5794

Lymphocytes

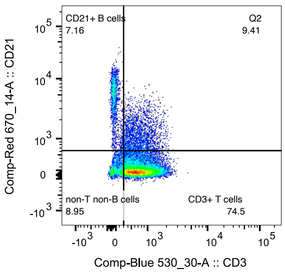

[20180806 Batch4_581_017.fcs]
Lymphocytes
24069

non-T non-B

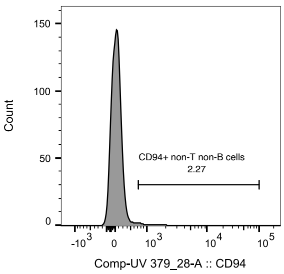

[20180806 Batch4_581_017.fcs]
non-T non-B cells
2155

581
06-AUG-2018

T cells

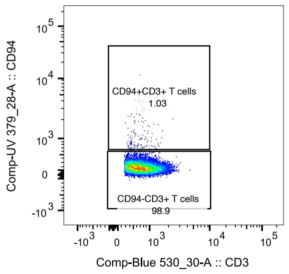

[20180806 Batch4_581_017.fcs]
CD3+ T cells
17924

CD94- T cells

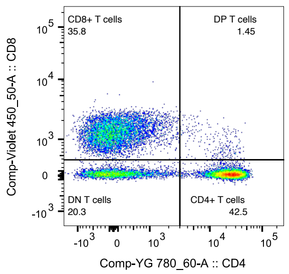

[20180806 Batch4_581_017.fcs]
CD94-CD3+ T cells
17720

CD4 T cells

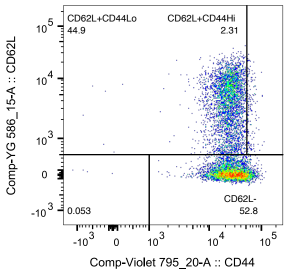

[20180806 Batch4_581_017.fcs]
CD4+ T cells
7532

CD4 T cells

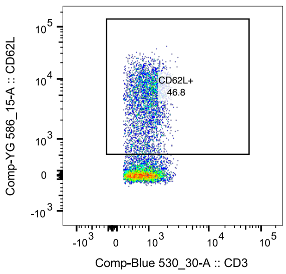

[20180806 Batch4_581_017.fcs]
CD4+ T cells
7532

CD8 T cells

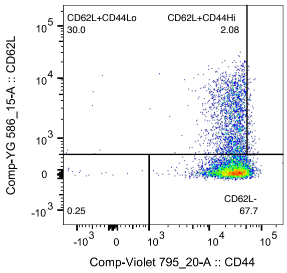

[20180806 Batch4_581_017.fcs]
CD8+ T cells
6341

CD8 T cells

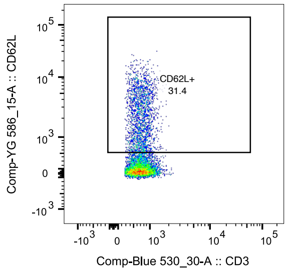

[20180806 Batch4_581_017.fcs]
CD8+ T cells
6341

DN T cells

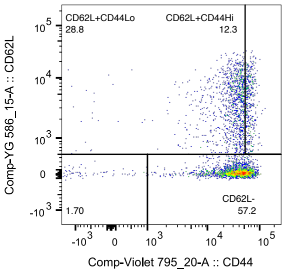

[20180806 Batch4_581_017.fcs]
DN T cells
3590

DN T cells

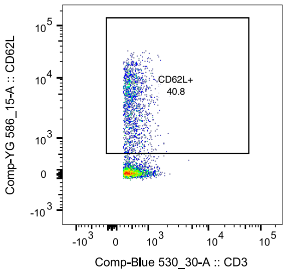

[20180806 Batch4_581_017.fcs]
DN T cells
3590

DP T cells

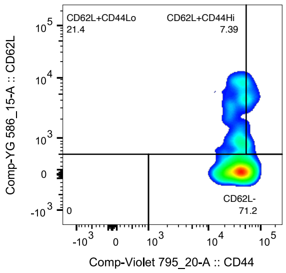

[20180806 Batch4_581_017.fcs]
DP T cells
257

DP T cells

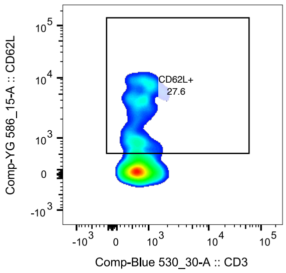

[20180806 Batch4_581_017.fcs]
DP T cells
257

575
06-AUG-2018

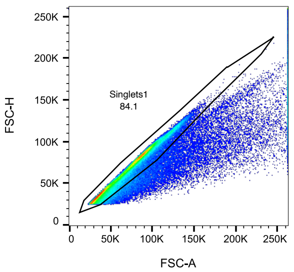

[20180806 Batch4_575_016.fcs]
Ungated
135293

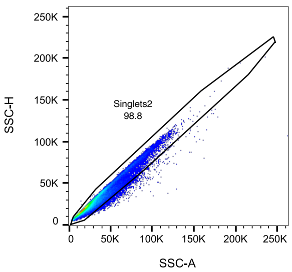

[20180806 Batch4_575_016.fcs]
Singlets1
113789

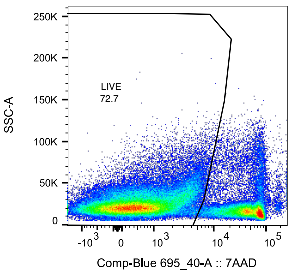

[20180806 Batch4_575_016.fcs]
Singlets2
112461

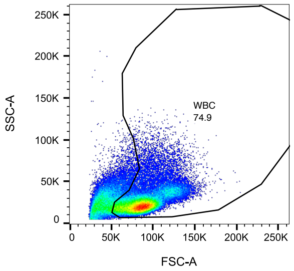

[20180806 Batch4_575_016.fcs]
LIVE
81717

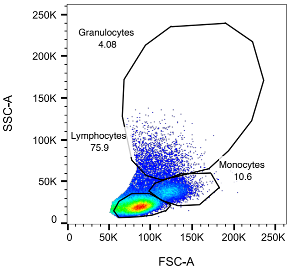

[20180806 Batch4_575_016.fcs]
WBC
61187

Granulocytes

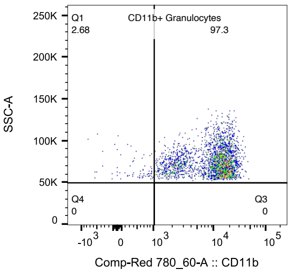

[20180806 Batch4_575_016.fcs]
Granulocytes
2496

Monocytes

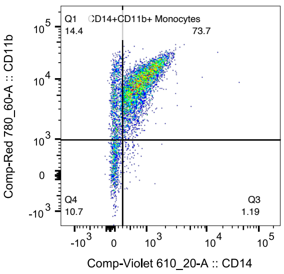

[20180806 Batch4_575_016.fcs]
Monocytes
6495

Lymphocytes

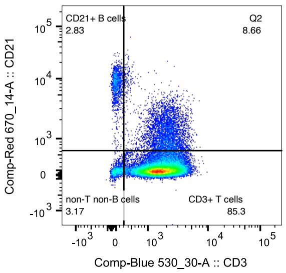

[20180806 Batch4_575_016.fcs]
Lymphocytes
46434

non-T non-B

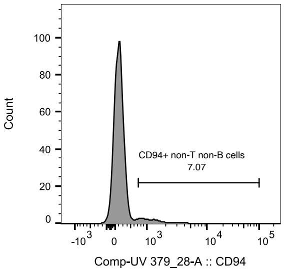

[20180806 Batch4_575_016.fcs]
non-T non-B cells
1471

575
06-AUG-2018

T cells

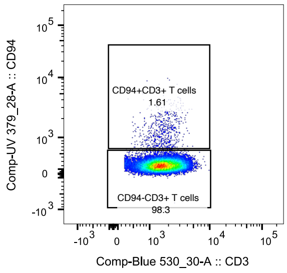

[20180806 Batch4_575_016.fcs]
CD3+ T cells
39629

CD94- T cells

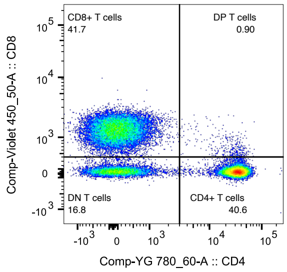

[20180806 Batch4_575_016.fcs]
CD94-CD3+ T cells
38949

CD4 T cells

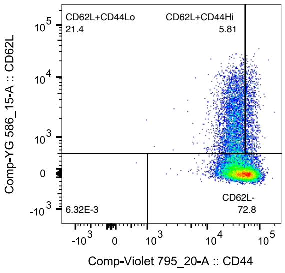

[20180806 Batch4_575_016.fcs]
CD4+ T cells
15830

CD4 T cells

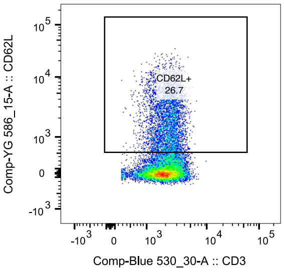

[20180806 Batch4_575_016.fcs]
CD4+ T cells
15830

CD8 T cells

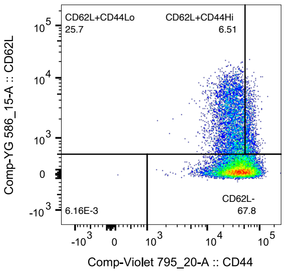

[20180806 Batch4_575_016.fcs]
CD8+ T cells
16237

CD8 T cells

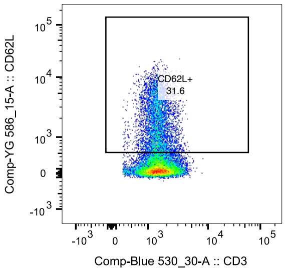

[20180806 Batch4_575_016.fcs]
CD8+ T cells
16237

DN T cells

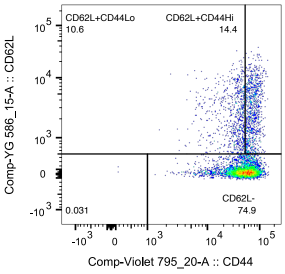

[20180806 Batch4_575_016.fcs]
DN T cells
6531

DN T cells

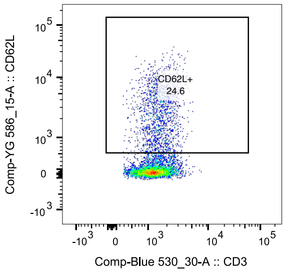

[20180806 Batch4_575_016.fcs]
DN T cells
6531

DP T cells

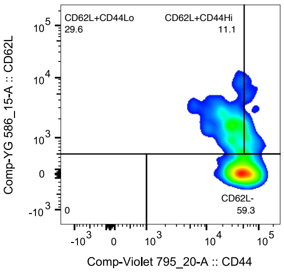

[20180806 Batch4_575_016.fcs]
DP T cells
351

DP T cells

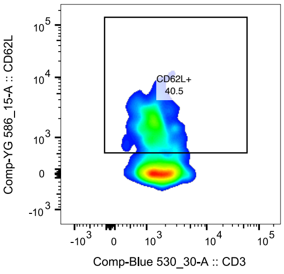

[20180806 Batch4_575_016.fcs]
DP T cells
351

586
06-AUG-2018

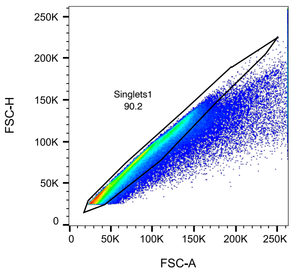

[20180806 Batch4_586_018.fcs]
Ungated
203577

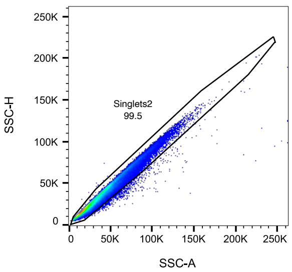

[20180806 Batch4_586_018.fcs]
Singlets1
183538

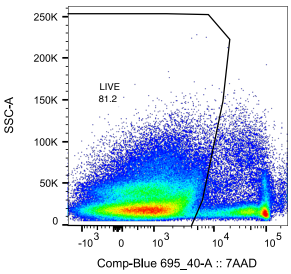

[20180806 Batch4_586_018.fcs]
Singlets2
182594

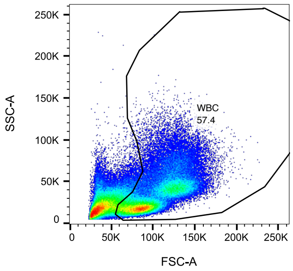

[20180806 Batch4_586_018.fcs]
LIVE
148330

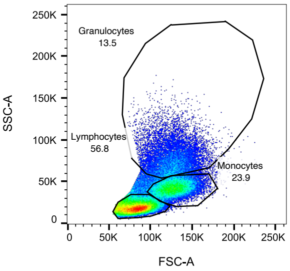

[20180806 Batch4_586_018.fcs]
WBC
85103

Granulocytes

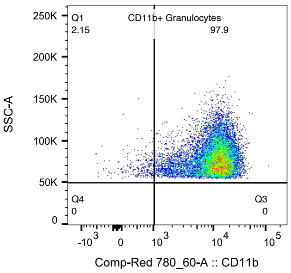

[20180806 Batch4_586_018.fcs]
Granulocytes
11460

Monocytes

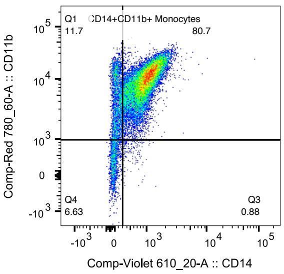

[20180806 Batch4_586_018.fcs]
Monocytes
20325

Lymphocytes

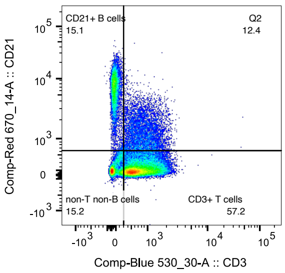

[20180806 Batch4_586_018.fcs]
Lymphocytes
48331

non-T non-B

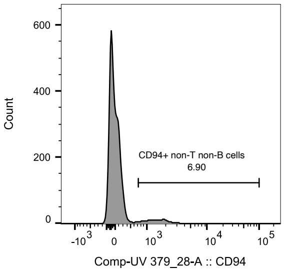

[20180806 Batch4_586_018.fcs]
non-T non-B cells
7351

586
06-AUG-2018

T cells

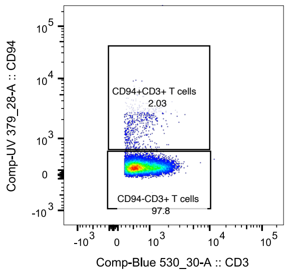

[20180806 Batch4_586_018.fcs]
CD3+ T cells
27647

CD94- T cells

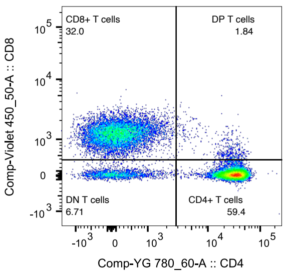

[20180806 Batch4_586_018.fcs]
CD94-CD3+ T cells
27050

CD4 T cells

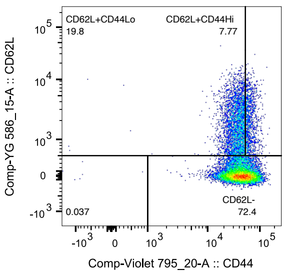

[20180806 Batch4_586_018.fcs]
CD4+ T cells
16069

CD4 T cells

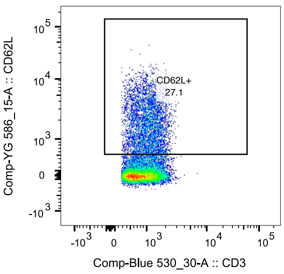

[20180806 Batch4_586_018.fcs]
CD4+ T cells
16069

CD8 T cells

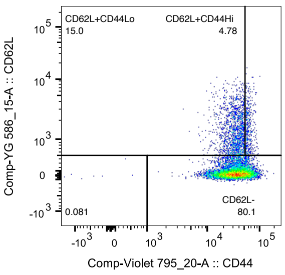

[20180806 Batch4_586_018.fcs]
CD8+ T cells
8668

CD8 T cells

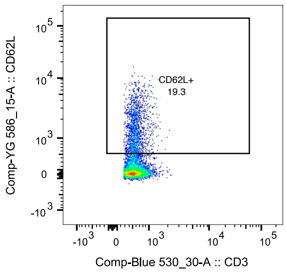

[20180806 Batch4_586_018.fcs]
CD8+ T cells
8668

DN T cells

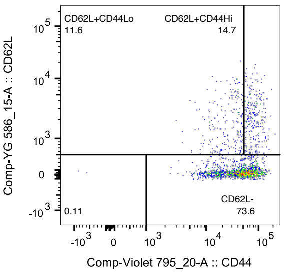

[20180806 Batch4_586_018.fcs]
DN T cells
1815

DN T cells

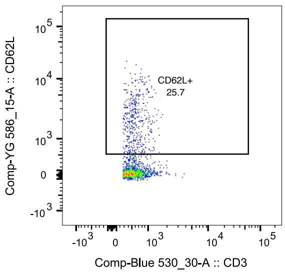

[20180806 Batch4_586_018.fcs]
DN T cells
1815

DP T cells

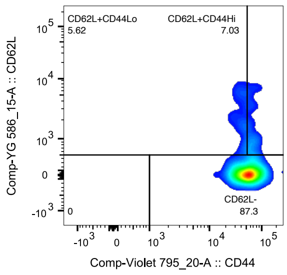

[20180806 Batch4_586_018.fcs]
DP T cells
498

DP T cells

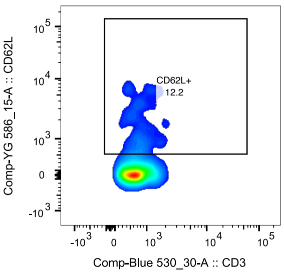

[20180806 Batch4_586_018.fcs]
DP T cells
498

605
06-AUG-2018

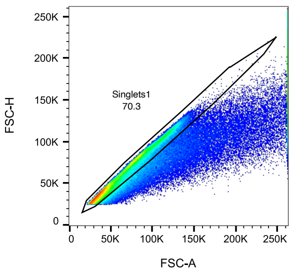

[20180806 Batch4_605_020.fcs]
Ungated
199595

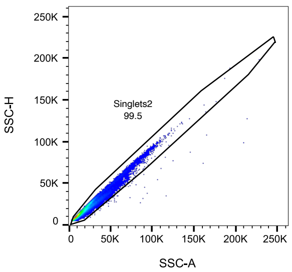

[20180806 Batch4_605_020.fcs]
Singlets1
140370

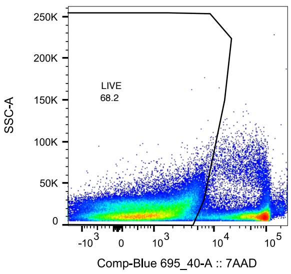

[20180806 Batch4_605_020.fcs]
Singlets2
139729

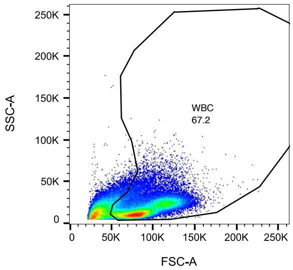

[20180806 Batch4_605_020.fcs]
LIVE
95260

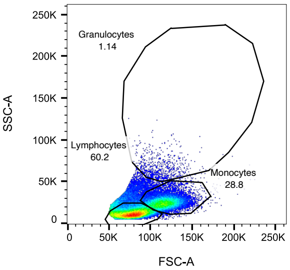

[20180806 Batch4_605_020.fcs]
WBC
63973

Granulocytes

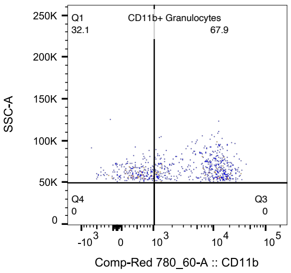

[20180806 Batch4_605_020.fcs]
Granulocytes
730

Monocytes

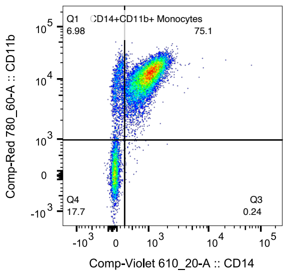

[20180806 Batch4_605_020.fcs]
Monocytes
18445

Lymphocytes

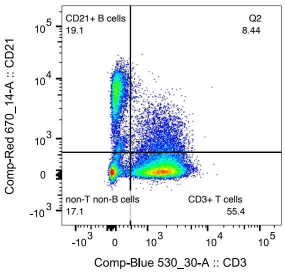

[20180806 Batch4_605_020.fcs]
Lymphocytes
38500

non-T non-B

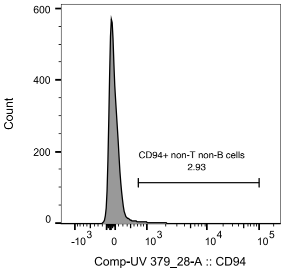

[20180806 Batch4_605_020.fcs]
non-T non-B cells
6592

605
06-AUG-2018

T cells

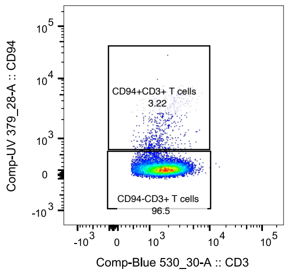

[20180806 Batch4_605_020.fcs]
CD3+ T cells
21312

CD94- T cells

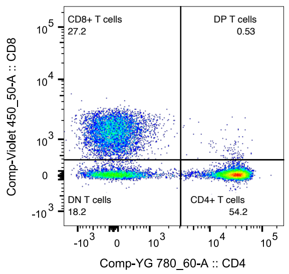

[20180806 Batch4_605_020.fcs]
CD94-CD3+ T cells
20570

CD4 T cells

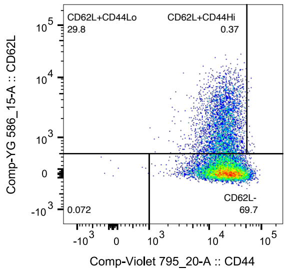

[20180806 Batch4_605_020.fcs]
CD4+ T cells
11140

CD4 T cells

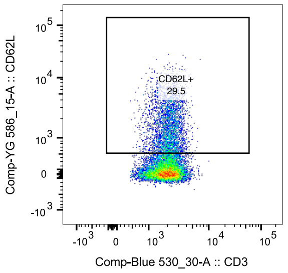

[20180806 Batch4_605_020.fcs]
CD4+ T cells
11140

CD8 T cells

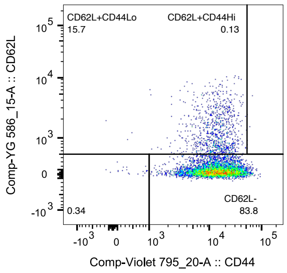

[20180806 Batch4_605_020.fcs]
CD8+ T cells
5585

CD8 T cells

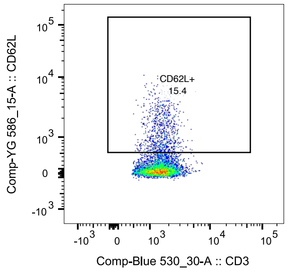

[20180806 Batch4_605_020.fcs]
CD8+ T cells
5585

DN T cells

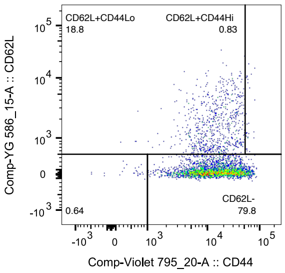

[20180806 Batch4_605_020.fcs]
DN T cells
3735

DN T cells

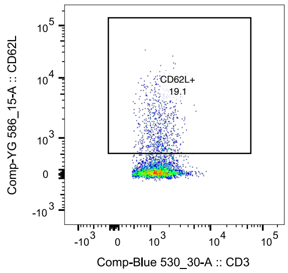

[20180806 Batch4_605_020.fcs]
DN T cells
3735

DP T cells

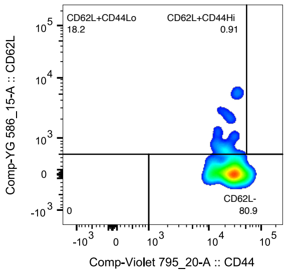

[20180806 Batch4_605_020.fcs]
DP T cells
110

DP T cells

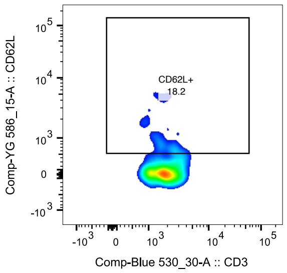

[20180806 Batch4_605_020.fcs]
DP T cells
110

545
06-AUG-2018

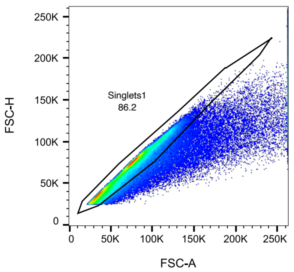

[20180806 Batch4_545_013.fcs]
Ungated
197689

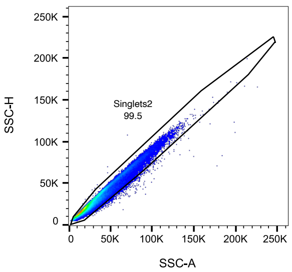

[20180806 Batch4_545_013.fcs]
Singlets1
170346

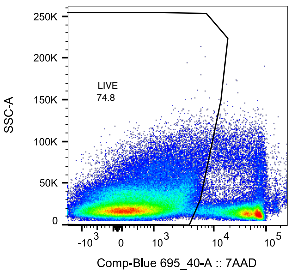

[20180806 Batch4_545_013.fcs]
Singlets2
169484

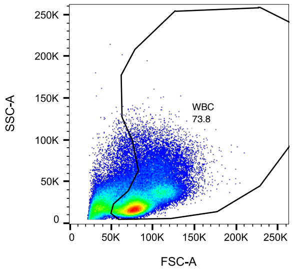

[20180806 Batch4_545_013.fcs]
LIVE
126718

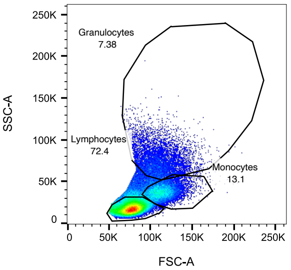

[20180806 Batch4_545_013.fcs]
WBC
93570

Granulocytes

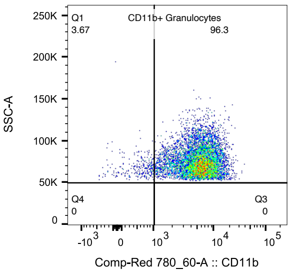

[20180806 Batch4_545_013.fcs]
Granulocytes
6901

Monocytes

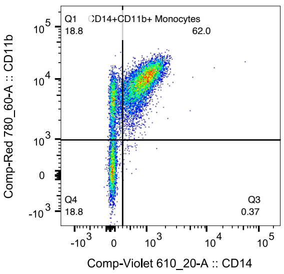

[20180806 Batch4_545_013.fcs]
Monocytes
12225

Lymphocytes

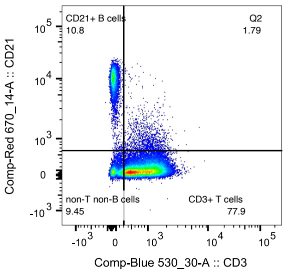

[20180806 Batch4_545_013.fcs]
Lymphocytes
67703

non-T non-B

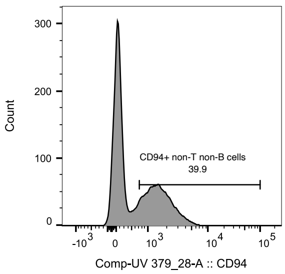

[20180806 Batch4_545_013.fcs]
non-T non-B cells
6401

545
06-AUG-2018

T cells

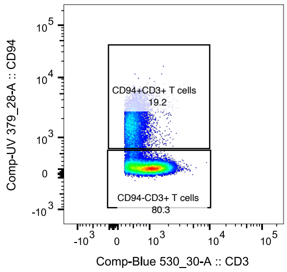

[20180806 Batch4_545_013.fcs]
CD3+ T cells
52758

CD94- T cells

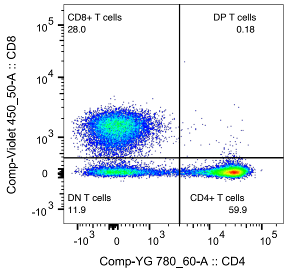

[20180806 Batch4_545_013.fcs]
CD94-CD3+ T cells
42342

CD4 T cells

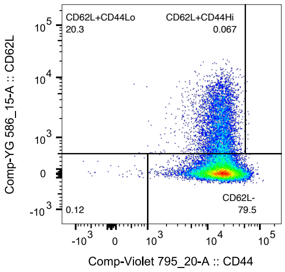

[20180806 Batch4_545_013.fcs]
CD4+ T cells
25363

CD4 T cells

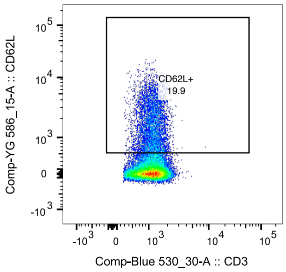

[20180806 Batch4_545_013.fcs]
CD4+ T cells
25363

CD8 T cells

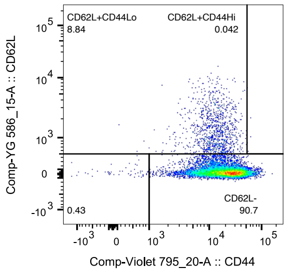

[20180806 Batch4_545_013.fcs]
CD8+ T cells
11867

CD8 T cells

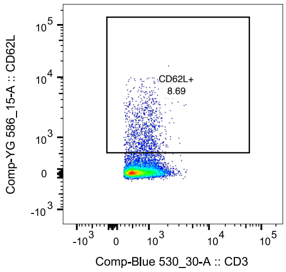

[20180806 Batch4_545_013.fcs]
CD8+ T cells
11867

DN T cells

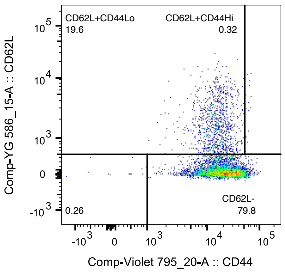

[20180806 Batch4_545_013.fcs]
DN T cells
5034

DN T cells

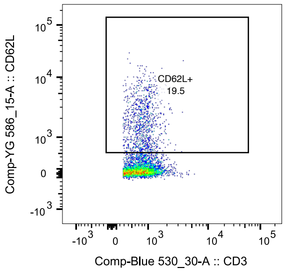

[20180806 Batch4_545_013.fcs]
DN T cells
5034

DP T cells

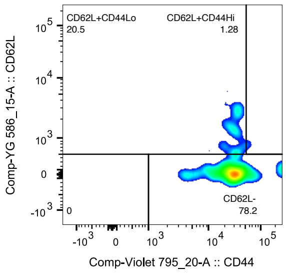

[20180806 Batch4_545_013.fcs]
DP T cells
78.0

DP T cells

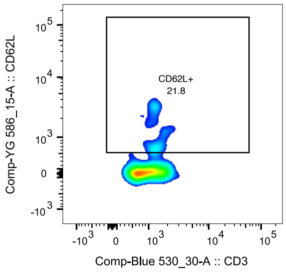

[20180806 Batch4_545_013.fcs]
DP T cells
78.0
