# Supplementary material for: DNA methylation and chromatin accessibility predict age in the domestic dog
Source: Aging Cell. 2024 Jan 23;23(4):e14079. doi: 10.1111/acel.14079 (PMC11019125; doi:10.1111/acel.14079)
Supplement: Supplementary file 4 — Figures S1–S3. [file ACEL-23-e14079-s001.docx]

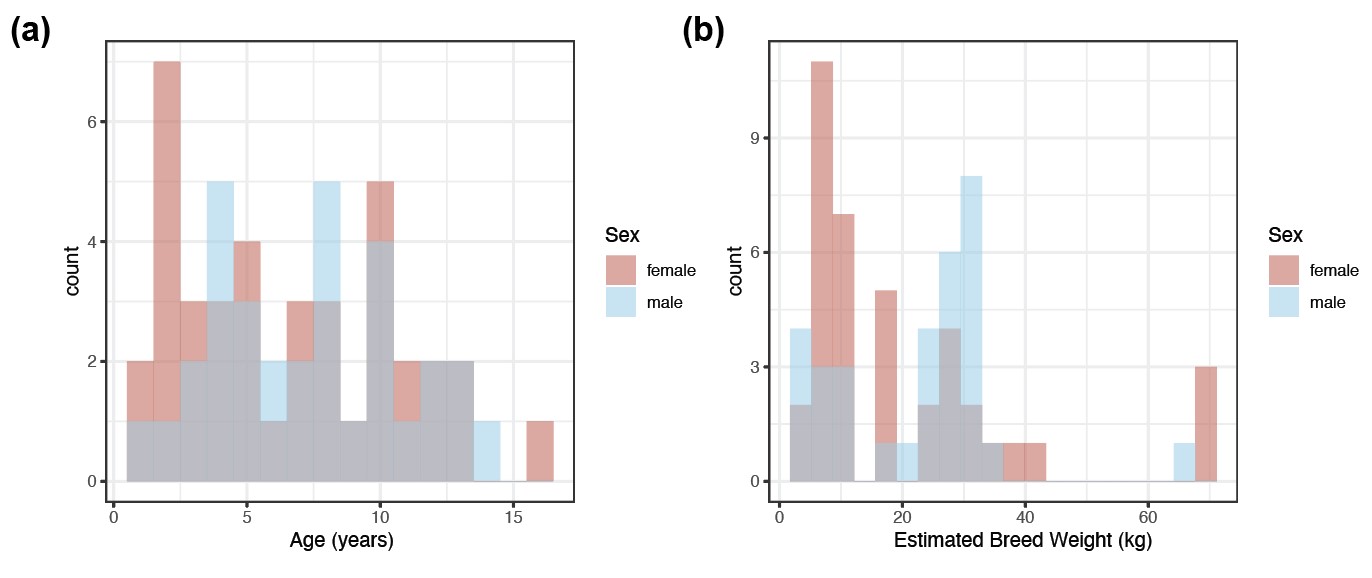


**Supplemental Figure 1.** (a-b) Distribution of ages (a) and estimated breed weights (b) of 71 dogs included in the cohort, colored by sex.


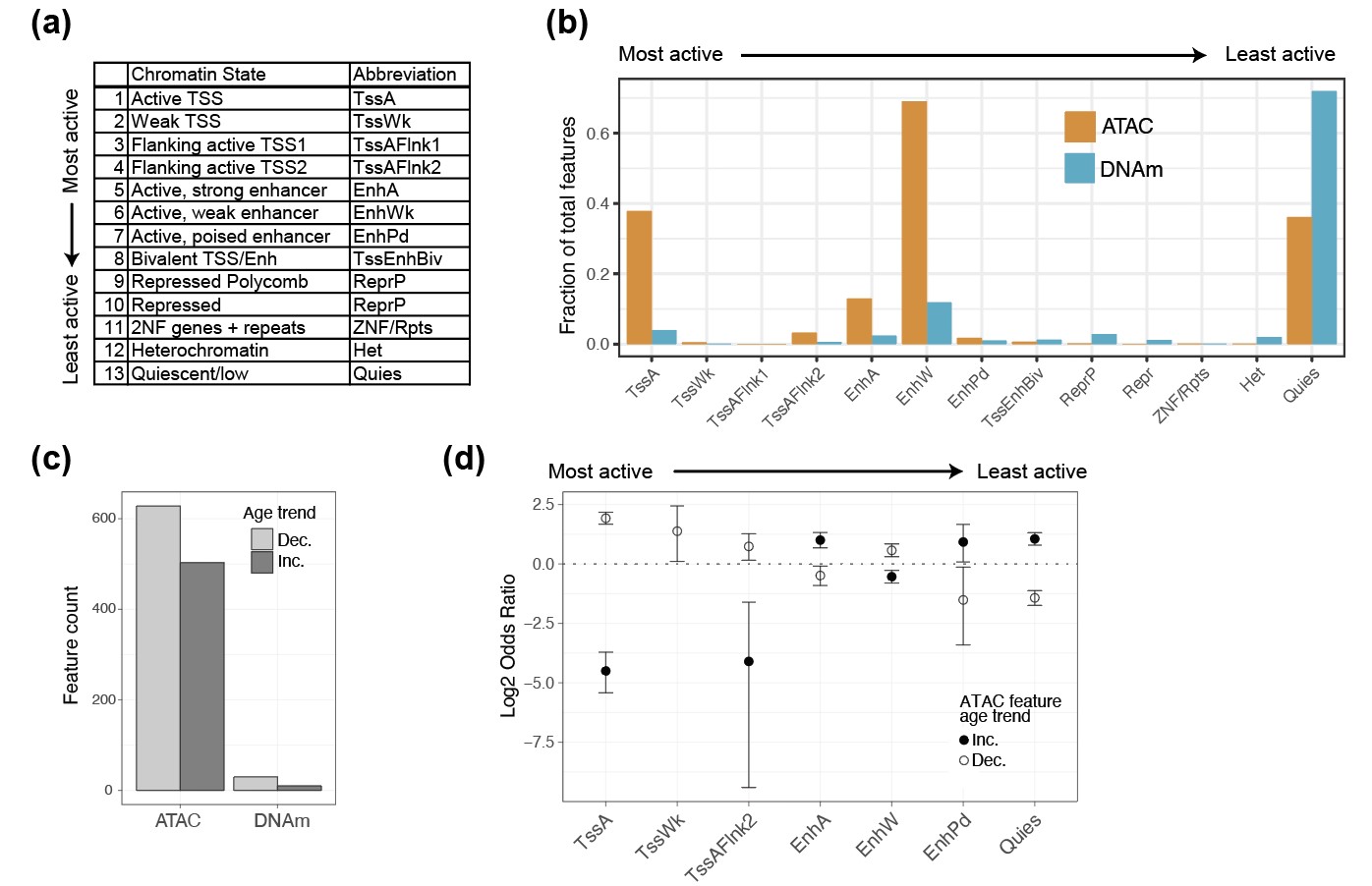


**Supplemental Figure 2. Functional annotation of epigenetic features and their association with age.** (a) Label and description of epigenetic regulatory elements annotated in the EpiC Dog ranked from most to least active (adapted from Son et al. 2023). (b) Breakdown of fraction of total features from ATAC and RRBS datasets that fall into each of the 13 chromatin categories described in (a). (c) Numbers of features significantly associated with age for each data type, broken down by whether or not they are increasing or decreasing with age. (d) Log2 odds ratios from Fisher’s exact test of enrichment of age-associated ATAC features by chromatin state and whether or not they are increasing or decreasing with age. Fisher’s exact test results from groups with p-val < 0.05 are plotted here.


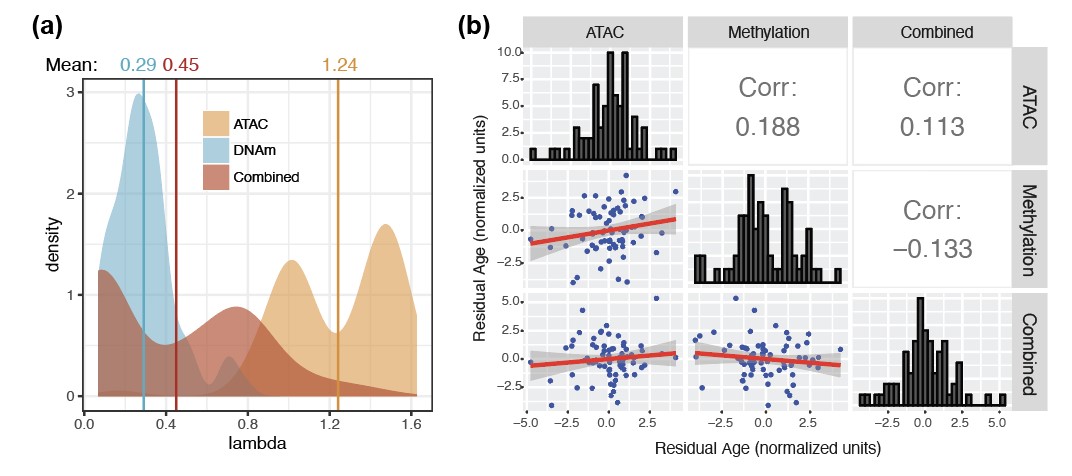


**Supplemental Figure 3. (a)** Distribution of optimized lambda parameters of all models. **(b)** Correlation between residual age predicted from each of the 3 clocks for both all models
